# Oxytocin and arginine vasopressin systems in the domestication process

**DOI:** 10.1590/1678-4685-GMB-2017-0069

**Published:** 2018-03-26

**Authors:** Bibiana S.O. Fam, Pamela Paré, Aline B. Felkl, Pedro Vargas-Pinilla, Vanessa R. Paixão-Côrtes, Lucas Henriques Viscardi, Maria Cátira Bortolini

**Affiliations:** 1 Departamento de Genética, Universidade Federal do Rio Grande do Sul, Porto Alegre, RS, Brazil

**Keywords:** Oxytocin and receptors, vasopressin and receptors, animal domestication, molecular evolution, positive selection

## Abstract

Domestication is of unquestionable importance to the technological revolution that has given rise to modern human societies. In this study, we analyzed the DNA and protein sequences of six genes of the oxytocin and arginine vasopressin systems (*OXT-OXTR; AVP-AVPR1a, AVPR1b and AVPR2)* in 40 placental mammals. These systems play an important role in the control of physiology and behavior. According to our analyses, neutrality does not explain the pattern of molecular evolution found in some of these genes. We observed specific sites under positive selection in *AVPR1b* (ω = 1.429, *p* = 0.001) and *AVPR2* (ω= 1.49, *p* = 0.001), suggesting that they could be involved in behavior and physiological changes, including those related to the domestication process. Furthermore, *AVPR1a*, which plays a role in social behavior, is under relaxed selective constraint in domesticated species. These results provide new insights into the nature of the domestication process and its impact on the OXT-AVP system.

## Introduction

The phenomenon of domestication did not go unnoticed by Charles Darwin. With *The Variation of Animals and Plants under Domestication*, published in January 1868, he devoted a whole book to the mechanisms underlying this intriguing process (Darwin, 1868). It is now well known that the complex process of animal domestication involves systematic selective pressures imposed by humans according to their needs and wishes. In a classic experiment modeling domestication, Russian researchers subjected silver foxes to rigorous artificial selection for tameness and correlated traits over more than 50 years. Although part of the lineages retained their ancestral traits, foxes selected for tamability showed a loss of wild-type behavior within relatively few generations, acquiring several classical morphological attributes of domesticated animals such as white spotting, floppy ears, and curly tails (Trut *et al.*, 2009). The selection of traits, such as tameness and the reduction of innate stress, aggression, fear and anxiety, allowed domesticated animals to coexist and coevolve with humans within their constructed niches. This complex process arguably contributed to the rapid spread of our species across the globe, as well as facilitating the emergence of our civilization (Künzl and Sachser, 1999; Wiener and Wilkinson, 2011; Wright, 2015).

In recent years, a picture of the genetic basis for domestication has started to emerge (Schubert *et al.*, 2014, Park *et al.*, 2015; Marsden *et al.*, 2016). Genes of the immune system, neuronal development and behavior were shown to have been co-opted as part of the domestication process (Albert *et al.*, 2012; Wright, 2015). For instance, gray wolves (*Canis lupus lupus*) and dogs (*Canis lupus familiaris*) are highly differentiated in their expression of serotonin receptor genes, consistent with behavioral changes as part of domestication (Li *et al.*, 2013). Importantly, domestication can lead to both the rapid fixation of alleles associated with phenotypes of interest and a relaxation of selective constraints previously imposed by natural selection, as has been described for dogs, horses (*Equus caballus*) and cows (*Bos taurus*) (Zeder, 2012).

In placental mammals, the paralogous nonapeptides oxytocin (OXT: Cys-Tyr-Ile-Gln-Asn-Cys-Pro-Leu-Gly) and arginine vasopressin (AVP: Cys-Tyr-Phe-Gln-Asn-Cys-Pro-Arg-Gly) play an important role in physiological and behavioral functions, such as water homeostasis, vasoconstriction, lactation, uterine contractions, parental care, control of aggression, anxiety and stress (Gimpl and Fahrenholz, 2001; Cagliani *et al.*, 2009; Young and Flanagan-Cato, 2012; McCall and Singer, 2012). In addition to the respective nonapeptides, the genes *OXT* and *AVP* encode the carrier protein neurophysin (NP) and a signal peptide (SP). While the nonapeptides OXT and AVP are relatively well conserved in placental mammals, neurophysin and the signal peptide are more variable. The patterns of variation found in the portion of the *OXT* and *AVP* genes encoding NP and SP are therefore central to our question of linking genetic variation to domestication. Because both NP and SP play an important role in the export of the nonapeptides to their specific site of action, variation in these proteins might provide a mechanism by which OXT and AVP-mediated responses differ, even in the face of nonapeptide sequence conservation.

OXT and AVP are produced in high quantities in the brain, but their systemic and/or tissue/organ specificity depends on an adequate interaction with NP and SP and their respective paralog G protein-coupled receptors (GPCRs), OXTR, AVPR1a, AVPR1b and AVPR2 (Kimura *et al.*, 1992; Yamashita and Kitano, 2013). Nonetheless, it is known that some level of crosstalk can occur in the interaction of these ligands and their receptors (Zingg and Laporte, 2003; Slusarz *et al.*, 2013). Like other GPCRs, OXTR, AVPR2, AVPR1a and AVPR1b all contain seven transmembrane domains (named TM1-TM7) as well as four extracellular (N-terminal tail-ECL3) and four intracellular (ICL1-C-terminal tail) domains. The N-terminal tail is crucial for the detection of molecules outside the cell, while the C-terminal mediates the activation of internal signal transduction. Extracellular (ECL) and intracellular (ICL) domains are important for the interaction with nonapeptides and G-proteins respectively, while transmembrane domains (TMs) are connected with both functions (Zingg and Laporte, 2003; Koehbach *et al.*, 2013).

AVPR1a, AVPR1b and OXTR play important roles in a range of social behaviors. AVPR1b is produced in the anterior pituitary gland, where it mediates the secretion of the adrenocorticotropic hormone (ACTH) (Koshimizu *et al.*, 2012). ACTH is an important component of the hypothalamic-pituitary-adrenal (HPA) axis, a key system controlling a range of important adaptive behaviors, including circadian rhythms, the stress reaction and responses to fear (Kalsbeek *et al.*, 2012; De Oliveira *et al.*, 2013; Terenina *et al.*, 2013). Through a connection with ACTH, HPA and other neuronal mechanisms, mutations in *AVPR1b* have been associated with altered responses to stress and aggressive behavior in *Sus scrofa* (Muráni *et al.*, 2010; Terenina *et al.*, 2013; Lucion and Bortolini, 2014). *AVPR1b* knockout mice showed normal predatory behavior, while presenting a reduced level of aggression against individuals of their own species (Wersinger *et al.*, 2007). Treatment with AVPR1a antagonists has been shown to result in reduced anxiety and offensive aggression in rats and hamsters, respectively (Liebsch *et al.*, 1996; Keverne and Curley, 2004; Ferris *et al.*, 2006). In addition, variation in the promoter region of *AVPR1a* could explain differences in behavior within and between species, including humans, bonobos, and chimpanzees (Staes *et al.*, 2016). OXTR is associated with a wide range of phenotypes. It is expressed in the uterus, where it controls uterine contraction, and in the mammary glands, where it regulates milk ejection; in the brain, it governs social learning and emotion (Kimura *et al.*, 1992; Guzmán *et al.*, 2013a,b; Yamashita and Kitano, 2013). Finally, AVPR2 stimulates urine concentration and helps maintain water homeostasis (Carter, 2013; Koshimizu *et al.*, 2012; Yamashita and Kitano, 2013). It is the only AVP receptor that is not directly involved with behavior; however, it increases the expression of AVPR1a through the formation of heterodimers with that receptor (Terrillon *et al.*, 2004). In a wider context, the increased availability of resources resulting from the contact with humans may have impacted on the diet of domesticated animals, potentially leading to metabolic changes, which may be reflected in the OXT-AVP system.

Recently, Paré *et al.* (2016) explored the evolutionary forces acting on the four receptors *OXTR*, *AVPR1a, AVPR1b*, and *AVPR2* in a set of 35 placental mammal orthologs. They found evidence for a dynamic scenario, in which *OXTR* was under evolutionary constraint, while *AVPR1a, AVPR1b*, and *AVPR2* exhibited elevated rates of evolution, indicating a relaxation of selective pressures, or even positive selection of novel vasopressin receptor variants in the Placentalia (Paré *et al.*, 2016). In the light of these recent findings of the genetic variability of the OXT-AVP system, we aimed to explore whether amino acid changes at *AVP, OXT, OXTR, AVPR2, AVPR1a*, and *AVPR1b* could be associated with the animal domestication process.

## Material and Methods

Studies of domestication are commonly based on a comparison between domesticated and their putative wild ancestral lineages. Assuming that wild and domesticated species carry different alleles, it is possible to identify the ancestral allele as the most common one in the studied mammals and to thus reconstruct the genetic background on which domestication occurred. This is relatively straightforward where the wild ancestral species is known, *e.g.* in the case of the dog, which we compared to the gray wolf (*Canis lupus;* PRJNA266585; Li *et al.*, 2013). However, extant wild species have followed their own evolutionary path from the moment of divergence to the present time (Freedman *et al.*, 2014). It is therefore ideal to use the ancient genomes of the ancestral species for comparison wherever possible. Ancestral DNA (aDNA) sequences were available for the putative wild ancestors of cattle (auroch, *Bos primigenius;* PRJNA294709; Park *et al.*, 2015) and horse (two Late Pleistocene equines; PRJEB7537; Schubert *et al.*, 2014). Finally, a further challenge lies in the fact that the wild ancestral species of many domesticated animals are either extinct or unknown. Wherever the putative ancestral species were unavailable, we therefore compared the domesticated species with their phylogenetically closest wild relative.

While the literature offers numerous definitions of a domesticated species (Larson and Burger, 2013), here we refer to a domesticated animal as “one whose mate choice is influenced by humans and whose tameness and tolerance of humans are genetically determined” (Driscoll *et al.*, 2009). Based on morphological and behavioral phenotypes shared between humans and a handful of domesticated species, *Homo sapiens* can be referred to as a self-domesticated species (Hare, 2017). Therefore, to avoid methodological bias, we ran evolutionary tests for gene sets with and without data from *Homo sapiens* and its phylogenetically closest (wild) relative, *Pan troglodytes.*

### Data mining

The coding sequences of *AVP, OXT* (including neurophysin and signal peptide sequences), *OXTR, AVPR2, AVPR1a*, and *AVPR1b* of 19 domesticated and 21 wild placental mammals (Table S1) were obtained from the NCBI GenBank genomic database (http://www.ncbi.nlm.nih.gov) and Ensembl (http://www.ensembl.org). Sequences with an identity value ≥ 98%, a minimum score of 200 and a statistically significant E-value (i.e. *Sus scrofa*, *Bos primigenius*, *Felis silvestris*, *Capra aegagrus*, *Ovis orientalis*, *Canis lupus* and ancient horse) were aligned using the MUSCLE algorithm (Edgar, 2004) implemented in Mega 6.0 (Tamura *et al.*, 2011). In order to maximize the number of species-specific sequences per gene, the set of homologous sequences analyzed for each gene varied, ranging from 26 for *AVP* and *OXTR*, to 36 for *AVPR1b* (Supplementary material - Alignment Analyses 1-6).

### Data analysis

To detect positive selection, we applied the NsSites test, implemented in PAML 4.9. This approach is based on an interspecific phylogenetic comparison of nonsynonymous and synonymous substitution rates in protein-coding genes. The type of selection is indicated by the value of the nonsynonymous/synonymous rate ratio ω = dN/dS. We compared neutral models with alternative models allowing positive selection, performing a pairwise comparison of two likelihood ratio tests (LRTs) of the following PAML models: M1a *vs.* M2a and M7 *vs.* M8 (Yang, 2007). Due to the inclusion of ancient genome sequences, such as that of the aurochs (*Bos primigenius;* Park *et al.*, 2015) and two Late Pleistocene equines (CGG10022 and CGG10023; Schubert *et al.*, 2014), all sites in the alignment were used in the analysis (“clean data=0”).

The Bayes Empirical Bayes (BEB) approach implemented in CODEML was used to select sites potentially under positive selection. Phylogenetic trees constructed in accordance with the published phylogenies of primates (Perelman *et al.*, 2011) and mammals (Meredith *et al.*, 2011; Song *et al.*, 2012) were used as input for PAML 4.9. For sites with a high (> 90%) posterior probability of being under positive selection in BEB, we determined the Grantham score (GS), a chemical measure for the fit between an amino acid and the one replacing it in a missense substitution. Based on their GS, amino acid changes were categorized as conservative (GS 0–50), moderately conservative (51–100), moderately radical (101–150), and radical (> 151) (Grantham, 1974; Li *et al.*, 1984).

Importantly, while the NsSites test has been developed to detect natural selection, we assume that signals of positive selection in domesticated animals reflect artificial selection. Furthermore, we applied the posterior Clade D test, a branch-site model implemented in PAML, to compare the evolutionary rates found in a domesticated species either to its putative wild ancestor species or to the most closely-related wild species whose genome is available. The phylogeny was divided into two clades, and an LRT was used to evaluate divergences in selective pressure between them, as indicated by different ω ratios. This model assumes two classes of sites, foreground and background sites, which were compared to the neutral model M1a using an LRT with three degrees of freedom. Importantly, the phylogenetic tree was built in a mirrored way, comparing the domesticated animals on the foreground branches with the wild animals on the background branches, always using the same number of species per branch. Any divergence in the evolutionary rates (ω ratios) of corresponding domesticated and wild branches can be thought to be due to either positive selection or the relaxation of natural selection. Figures S1-S6 show two structurally different trees for each analyzed protein: the tree at the top is a “mirrored tree”, which artificially separates the foreground branches (domesticated animals) from the background branches (wild animals; e.g., Figure S1.1). The tree at the bottom represents the actual phylogenetic tree, as published in the literature (e.g., Figure S1.2). Importantly, the actual phylogenetic trees were used in the NsSites analyses, while the mirrored trees were used in the branch comparisons for Clade D and RELAX (see below).

We searched for signals of relaxed selection using the online tool RELAX (http://test.datamonkey.org/relax; Delport *et al.*, 2010; Wertheim *et al.*, 2015). RELAX allows to infer the variation of evolutionary rates both between sites and across branches. The relaxation of natural selection is indicated by an increase (for ω < 1) or decrease (for ω > 1) of ω toward 1. In the context of models that compared two or more branches in a phylogeny (branch-site models), different proportions of sites fall into each ω category, leading to two different effects: On the one hand, the values inferred for the each selection category can move toward 1. On the other hand, the proportions of sites belonging to the different categories can change in such a way that more sites are assigned to categories with ω values closer to 1 (Wertheim *et al.*, 2015). It should be stressed that RELAX uses the *K*-value, which measures the selection strength of each branch. A *K* > 1 indicates that the respective branch has been subjected to stronger purifying selection in relation to the other branch(es). Alternatively, sites that are under moderate positive selection in the reference branch are subjected to stronger positive selection. *K*-values < 1 for a branch indicate the relaxation of purifying selection (Wertheim *et al.*, 2015).

## Results and Discussion

Domesticated species are subject to severe artificial selection. They can therefore be expected to present genetic changes when compared with their putative wild ancestral species, or with phylogenetically related species in the wild. Here, we used classical approaches to infer positive selection through the interspecific phylogenetic comparison of six candidate genes and the evaluation of different evolutionary rates between wild and domesticated species. In the present context we assume that a signal of positive selection can reflect artificial selection.

### Analysis of positive selection

Initially, we searched for relevant selection signals to answer our original question whether changes in *AVP, OXT, OXTR, AVPR2, AVPR1a*, and *AVPR1b* could be associated with the animal domestication process. Therefore, we only considered sites with significant differences in the amino acids between a domesticated species and its putative wild ancestor or a phylogenetically closely related wild species.

The NSsites analysis of *AVPR1b* and *AVPR2* revealed that the different selective pressures acting in wild and captive environments left molecular footprints at specific sites within these genes in both sets of animals (Table 1).

**Table 1:** Sites within *AVPR1B* and *AVPR2* with a high probability (> 90%) of being under positive selection in domesticated animals (ω value > 1 for Model 8).

AVPR1B						
Domain	AA position	Wild species^1^	AA	Domesticated species	GS	Probability^2^
C-TERMINAL	404	*Camelus bactrianus*	Val-Glu	*Vicugna pacos*	121	0.964
	404	*Octodon degus*	Val-Ala	*Cavia porcellus*	64	0.964
	404	*Marmota marmota*	Ala-Glu	*Chinchilla lanigera*	107	0.964
	404	*Ochotona princeps*	Gly-Gln	*Oryctolagus cuniculus*	98	0.964
**AVPR2**						
N-TERMINAL	3	*Marmota marmota*	Leu-Ser	*Cavia porcellus*	145	0.994
	3	*Camelus ferus*	Leu-Met	*Camelus dromedarius*	15	0.994
	3	*Sus scrofa*	Arg-Ala	*Sus scrofa familiaris*	112	0.994
	3	*Bos primigenius*	Ser-Met	*Bos taurus*	135	0.994
	3	*Ovis orientalis*	Cys-Met	*Ovis aries*	196	0.994
	3	*Capra aegagrus*	Ser-Met	*Capra hircus*	135	0.994
	3	*Ailuropoda melanoleuca*	Met-Thr	*Mustela putorius furo*	81	0.994
ICL3	257	*Peromyscus maniculatus*	Ile-Ser	*Mus musculus*	142	0.922
	257	*Ictiodomys tridecemlineatus*	Arg-Thr	*Mesocricetus auratus*	71	0.922
	257	*Ochotona princeps*	His-Ser	*Oryctolagus cuniculus*	89	0.922
ECL3	302	*Marmota marmota*	Leu-Arg	*Cavia porcellus*	102	0.911
	302	Ancient Horse	Pro-Arg	*Equus caballus*	103	0.911
	302	*Bos primigenius*	Pro-Arg	*Bos taurus*	103	0.911
	302	*Capra aegagrus*	His-Arg	*Capra hircus*	29	0.911
	302	*Felis silvestris*	Pro-Arg	*Felis catus*	103	0.911
	302	*Ailuropoda melanoleuca*	Leu-Val	*Mustela putorius furo*	32	0.911

1 Putative ancestral species or phylogenetically related wild species;

2 probability of being under positive selection in the Bayes Empirical Bayes (BEB) analysis.

**Figure 1:**
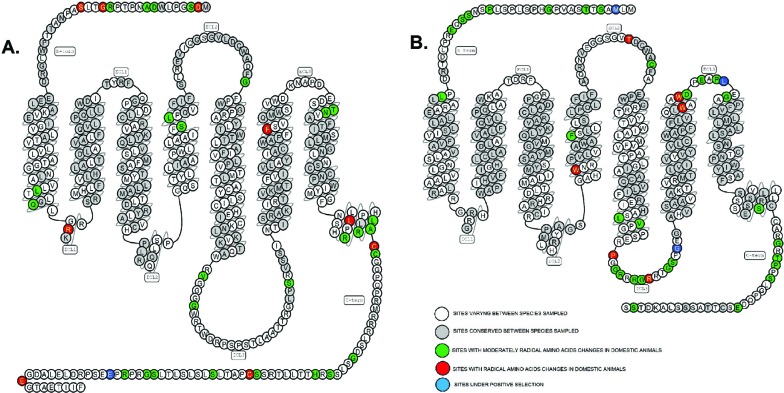
Snake plot of (A) AVPR1b and (B) AVPR2 (Reference: www.gpcrdb.org).

Specifically, *AVPR1b* was found to harbor one site with a posterior probability > 95% of being under positive selection (position 404 of AVPR1b; ω = 1.429, *p* = 0.001). *AVPR2* (ω= 1.49, *p* = 0.001) was found to contain three such sites (positions 3, 257 and 302 of AVPR2; Table S2; Figure 1; see also Material and Methods). The evolutionary model that best fitted both *AVPR1b* and *AVPR2* was M8 (*p* = 0.001 and *p* < 0.001, respectively; Table S2).

AVPR1b position 404 is located within the C-terminal, an important region for G-protein interaction (Zingg and Laporte, 2003). Although this region is highly variable across mammals, all carnivores carry the same amino acid, suggesting high evolutionary conservation within this clade (Table S3).

It is worthy of note that the Alpaca (*Vicugna pacos*) also differs from its closest wild relative at position 404 of AVPR1b, with a moderately radical change (GS = 121). Since AVPR1b is known to play an important role in behavior, this nonsynonymous substitution could have been important for the domestication of Alpaca in Pre-Hispanic South America (Westbury *et al.*, 2016). A role for AVPR1b in the behavioral changes associated with domestication is consistent with findings of a reduced basal plasma levels of ACTH in foxes subjected to rigorous artificial selection for domestication phenotypes over more than 50 years (Trut *et al.*, 2009): ACTH is an important hormone of the hypothalamic-pituitary-adrenal (HPA) axis, and its secretion in this axis is modulated by the AVPR1b receptor (Kalsbeek *et al.*, 2012; Terenina *et al.*, 2013). Interestingly, *Chinchilla lanigera* also carries a moderately radical change at this site. However, the chinchilla was domesticated for its fur (Baranowski *et al.*, 2013), and the impact of the domestication process on its behavior is still uncertain.

Within AVPR2, positions 3, 257 and 302 were found to be under positive selection (Tables 1 and S2). Located in the N-terminal region of the protein, position 3 has a 99% probability of being under positive selection. Seven domesticated species differed from their wild relatives at this position: *Cavia porcellus, Camelus dromedarius, Sus scrofa familiaris, Bos taurus, Ovis aries, Capra hircus and Mustela putorius furo*. Five of these seven modifications led to an at least moderately radical amino acid change. Interestingly, four domesticated Artiodactyla species carry a methionine at this position (*Camelus dromedarius*, *Bos taurus*, *Ovis aries*, and *Capra hircus*) (Tables 1 and S3). Based on its distribution across the species, Met can be considered the ancestral amino acid. It should be stressed that this amino acid is maintained only in domesticated Artiodactyla, while three other amino acids, Leu, Ser and Cys, are found in their respective wild relatives. Assuming that Met is not the ancestral amino acid, this can be suggested as an instigating case of molecular convergence in domesticated animals (Zhang and Kumar, 1997; Melville *et al.*, 2006). Therefore, it is possible that this site might have evolved at a different rate in both sets of animals, at least within the Artiodactyla clade.

Five domesticated animals (*Equus caballus, Bos taurus, Cavia porcellus, Capra hircus*, and *Felis catus*) had an arginine at position 302, which is under positive selection with a probability of > 90% (ECL3, Figure 1; Tables 1, S2 and S3). Like the Met at position 3, this is another case where an ancestral amino acid seems has been retained in the domesticated branch. However, the presence of the same amino acid concomitantly in the different species due to convergence cannot be discarded (Table S3). Finally, position 257 (ICL2, Figure 1) has a probability of 92% of being under positive selection. At this site we found three domestic Glires (rodents and lagomorphs) presenting three different amino acid changes (Tables 1 and S3) compared to their wild relatives.

Changes in AVPR2 are associated with metabolic functions, such as homeostasis, rather than behavior (Carter, 2013; Koshimizu *et al.*, 2012; Yamashita and Kitano, 2013). However, genomic analyses have revealed that genes involved in metabolism are under selection in domesticated animals, including cats (*Felis catus*) (Montague *et al.*, 2014). Our results support the idea that dietary manipulation and an increased availability of resources provided by humans, including abundant water supply, may have led to functional changes in AVPR2 in domesticated animals.

Our analyses have shown that *OXT*, *AVP* and *AVPR1a* are under purifying selection. As expected, *OXTR* seems to be under evolutionary constraint in the set of mammals considered here, corroborating earlier studies (Paré *et al.*, 2016). However, despite this general pattern, there is evidence that *OXTR* has been under positive selection in some New World monkey branches, where it has coevolved with taxon-specific OXT forms (Vargas-Pinilla *et al.*, 2015). Of note, the inclusion of *Homo sapiens* and our wild relative *Pan troglodytes* yielded the same results (data not shown).

### Relaxation of selective constraints

A relaxation of selective pressure has previously been described for some domesticated species, including dogs and horses (Price, 1999; Zeder, 2012; Li *et al.*, 2013; Schubert *et al.*, 2014). Analysis of the Clade D model showed that evolutionary rates differed between the branches carrying domesticated animals and those carrying their respective wild relatives for all genes in the present study. However, ω values were below 1 (Table S4), indicating neutrality or relaxed selective constraint. We therefore applied RELAX, a program specialized in the identification of relaxed constraint, on the same dataset. Results from these analyses showed a relaxation of selective constraints on *AVPR1a* in the domesticated branch (k = 0.47, *p* = 0.003). Since within-group aggression can imply the loss of one or several animals, the control of social behavior is extremely important in the domestication process. Within AVPR1a, the regions with the highest level of variation were the N-terminal and ECL3 domains, which interact with the AVP and OXT nonapeptides (Zingg and Laporte, 2003; Koehbach *et al.*, 2013). AVPR1a is known for its role in behavior, particularly the control of aggression and anxiety (Staes *et al.*, 2016). Nevertheless, the administration of AVPR1a antagonists has been found to reduce anxiety in rats (Liebsch *et al.*, 1996) and offensive aggression in hamsters (Ferris *et al.*, 2006). Thus, a relaxation of selective constraint on the *AVPR1a* gene in domesticated animals may reflect the fact that aggressive and anxiolytic behaviors play a lesser part in fitness and survival in captivity than in the wild.

*AVP* (k = 2.5, *p* < 0.001) and *OXT* (k = 1.4, *p* = 0.04) were found to be under moderate purifying selection in the reference branches (wild animals), as previously suggested by our group (Paré *et al.*, 2016). In the branch test, purifying selection was found to be even stronger in domesticated animals. In other words, it seems that there is a higher constraint to change in domesticated animals than in wild ones. Notwithstanding, *AVP*-coded neurophysin (NP) has a relatively high level of variation, with some amino acid changes in rodents, carnivores and artiodactyls. For example, radical changes with respect to the wild species are observed at position NP-42 in *Canis lupus, Sus scrofa*, and *Capra aegagrus* (GS= 159-192), with an ancestral cysteine present in the ancient horse and the remaining 23 animals. In *OXT*, the most variable region is also that encoding NP. Radical modifications in wolf (Asp35Leu), mouflon sheep (Cys41Gly) and wild goat (Cys41Tyr) were observed compared to their putative domesticated correspondents. The same pattern of change was found in the wild goat (Cys52Leu and Cys116Phe), wild boar (Cys116Arg) and mouflon sheep (Gly116Cys; Table S3; all artiodactyls).

Additionally, despite the conservation of AVP across placental mammals, we identified a lysine (Lys) at position 8, both in the domestic pig (*Sus scrofa familiaris*) and the wild boar (*Sus scrofa*). The preservation of this rare change in *Sus scrofa familiaris*, even after severe artificial selection, may indicate an indispensable functional importance of AVP-8Lys in the *Su*s genus. The other receptor genes, *AVPR1b* (k = 1.04, *p* = 0.6), *AVPR2* (k = 0.99, *p* = 0.9) and *OXTR* (k = 1.1, *p* = 0.1), did not show signals of a relaxation of natural selection. This is expected since we identified positive selection at least in the *AVPR1b* and *AVPR2* genes, demonstrating the contrasting selection pressures in wild and captive environments.

Tameness and the reduction of stress, fear and anxiety are traits associated with domestication (Künzl and Sachser, 2009). Our results indicate that the *OXT* and *AVP* systems likely play an important role in this process. Overall, the sites underlying the evolutionary signals found here could be involved in aggressive and stress control phenotypes (*e.g.* C-terminal changes at position 404 of AVPR1b), but also in physiological traits related to conditions caused by humans (e.g*.* AVPR2-3Met in domesticated Artiodactyla). Importantly, genes underlying behavior that would be selected for in the natural environment, such as aggression, would be subject to a relaxation of selective pressures in the captive environment, as suggested by our results for AVPR1a. Last but not least, the analyses of selective constraint that included humans and chimpanzees did not differ from those excluding them.

## Conclusion

In nature, taxon-specific genetic changes have been associated with taxon-specific phenotypes, including adaptive behaviors. Following this idea, we describe amino acid changes at two GPCR receptors (AVPR1b and AVPR2) that are under positive selection comparing domesticated and wild species. While *AVP* and *OXT* were identified as under strong purifying selection, *AVPR1a* shows a clear signal of relaxed selective pressure in domesticated animals. Based on the known function of these genes, these patterns of selection may be thought to be associated with physiological and behavior modifications needed for domesticated animals to coexist and coevolve with humans in their constructed niches.

## Supplementary material

- The following online material is available for this article:

Alignment analyses - Alignments (1-6) used in the analyses of the different species of wild and domesticated mammals.

Table S1 - Species of wild and domestic placental mammals available in genome data banks and used in the analyses.

Table S2 - Estimated parameters under different codon substitution models for *OXT* and *AVP* system genes.

Table S3 - OXT-AVP system amino acid positions with moderately radical or radical changes according to the Grantham Score (GS > 101).

Table S4 - Results of the Clade D analysis.

Figure S1 - Phylogenetic trees of 26 species of mammals used in *AVP* analysis, 1: mirrored tree, 2: phylogenetic tree.

Figure S2 - Phylogenetic trees of 26 species of mammals used in *AVPR1a* analysis, 1: mirrored tree, 2: phylogenetic tree.

Figure S3 - Phylogenetic trees of 26 species of mammals used in *AVPR1b* analysis, 1: mirrored tree, 2: phylogenetic tree.

Figure S4 - Phylogenetic trees of 26 species of mammals used in *AVPR2 analysis*, 1: mirrored tree, 2: phylogenetic tree.

Figure S5 - Phylogenetic trees of 26 species of mammals used in *OXT* analysis, 1: mirrored tree, 2: phylogenetic tree.

Figure S6 - Phylogenetic trees of 26 species of mammals used in *OXTR* analysis, 1: mirrored tree, 2: phylogenetic tree.
