# Supplementary material for: Oxytocin and arginine vasopressin systems in the domestication process
Source: Genet Mol Biol. 2018 Mar 26;41(1 Suppl 1):235–42. doi: 10.1590/1678-4685-GMB-2017-0069 (PMC5913714; doi:10.1590/1678-4685-GMB-2017-0069)
Supplement: Supplementary file 1 [file 1415-4757-GMB-41-01-2017-0069-s001.pdf]

Supplementary Material to "Oxytocin and Arginine Vasopressin Systems in the Domestication Process"

Table S1 - Species of wild and domestic placental mammals available in genome data banks and used in the analyses

DOMESTIC						
**PLACENTAL MAMMALS**	**OXT**	**OXTR**	**AVP**	**AVPR1A**	**AVPR1B**	**AVPR2**
Homo sapiens	XM_011529238.1	NM_000916.3	NM_000490.4	NM_000706.4	NM_000707.3	XM_006724828.3
Canis lupus familiaris	ENSCAFG00000006437	XM_005632029.1	NM_001197149.1	NM_001198658.1	XM_545695.2	XM_005640983.2
Mustela putorius furo	ENSMPUG00000008974	XM_004738430.2	XM_004772898.2	XM_004766600.2	XM_004756498.1	XM_004780683.2
Felis catus	NOT AVAILABLE	XM_003982446.2	XM_011280939.1	XM_003988975.3	XM_003999425.2	XM_006944113.2
Bubalus bubalis	XM_006042729.1	XM_006070767.1	XM_006042730.1	XM_010841597.1	XM_006065667.1	XM_006043661.1
Bos taurus	ENSBTAG00000008026	NM_174134.2	NM_176854.2	NM_001104990.1	NM_001192142.1	XM_005227662.3
Vicugna pacos	NOT AVAILABLE	NOT AVAILABLE	XM_006207409.2	XM_006202854.2	XM_006215470.2	XM_015251376.1
Camelus dromedarius	NOT AVAILABLE	XM_010985013.1	NOT AVAILABLE	XM_010980297.1	XM_010991414.1	XM_010991454.1
Capra hircus	XM_018057424.1	XM_018038465.1	XM_018057423.1	XM_013963910.1	XM_005690409.1	XM_018045070.1
Ovies aries	NM_001009801.2	HM856597.1	NM_001126341.1	NM_001199792.1	NM_001246237.1	NM_001199793.1
Sus scrofa familiaris	NM_001167589.1	XM_005669742.2	NM_213952.2	XM_003126346.4	XM_003130445.2	NM_214232.1
Equs assinus	NOT AVAILABLE	XM_014857931.1	NOT AVAILABLE	XM_014860956.1	XM_014830295.	XM_014832489.1
Equus caballus	ENSECAG00000007628	XM_014731360.1	ENSECAG00000024857	XM_001917923.4	XM_001489521.2	XM_005614629.1
Chinchilla lanigera	NOT AVAILABLE	XM_013509073.1	NOT AVAILABLE	XM_013520787.1	XM_013523068.1	XM_013509698.1
Cavia porcellus	ENSCPOG00000001717	XM_013146337.1	XM_003476416.2	XM_003475951.2	XM_003474668.3	XM_003462180.3
Mesocricetus auratus	HM357357.1	XM_005066161.2	NOT AVAILABLE	NOT AVAILABLE	XM_005079846.1	XM_005086933.2
Rattus norvegicus	ENSRNOG00000021225	XM_008763182.2	NM_016992.2	NM_053019.2	NM_017205.3	XM_006229549.3
Mus musculus	ENSMUSG00000027301	ENSMUSG00000049112	NM_009732.2	NM_016847.2	NM_011924.2	XM_006527756.3
Oryctolagus cuniculus	ENSOCUG000000083521	XM_008273575.2	XM_002710880.2	XM_002711237.2	XM_002717441.1	XM_008250316.2
**WILD**						
**PLACENTAL MAMMALS**	**OXT**	**OXTR**	**AVP**	**AVPR1A**	**AVPR1B**	**AVPR2**
Pan troglodutes	XM_001160221.5	XM_016940345.1	XM_001160259.5	XM_003952135.2	ENSPTRG00000023708	XM_001145732.4
Canis lupus	PRJNA266585	PRJNA266585	PRJNA266585	PRJNA266585	PRJNA266585	PRJNA266585
Odobenus rosmarus	XM_004398165.2	XM_004412946.1	XM_004398166.1	XM_004416459.1	XM_004392841.1	XM_004409179.1
Ailuropoda melanoleuca	NOT AVAILABLE	XM_002920379.2	XM_002918796.2	XM_002919594.2	XM_011236288.1	XM_002928814.3
Felis Silvestris	NOT AVAILABLE	PRJNA32759	PRJNA32759	PRJNA32759	PRJNA32759	XM_006944113.2
Bos mutus	NOT AVAILABLE	XM_005907085.2	NOT AVAILABLE	XM_005902535.2	XM_005910059.1	XM_005909075.1
Bos primigenius	PRJNA294709	PRJNA294709	PRJNA294709	PRJNA294709	PRJNA294709	PRJNA294709
Camelus bactrianus	NOT AVAILABLE	XM_010953164.1	NOT AVAILABLE	XM_010970522.1	XM_010963266.1	XM_010964086.1
Camelus ferus	NOT AVAILABLE	XM_006195359.2	NOT AVAILABLE	XM_006192369.2	XM_006182275.2	XM_014567101.1
Capra aegagrus	PRJNA261263	PRJNA261263	PRJNA261263	PRJNA261263	PRJNA261263	PRJNA261263
Ovis orientalis	PRJEB3139 and PRJEB3141	PRJEB3139 and PRJEB3141	PRJEB3139 and PRJEB3141	PRJEB3139 and PRJEB3141	PRJEB3139 and PRJEB3141	PRJEB3139 and PRJEB3141
Sus scrofa	PRJNA255085	PRJNA255085	PRJNA255085	PRJNA255085	PRJNA255085	PRJNA255085
Ancient horse	PRJEB7537	PRJEB7537	PRJEB7537	PRJEB7537	PRJEB7537	PRJEB7537
Equus przewalskii	NOT AVAILABLE	XM_008532482.1	NOT AVAILABLE	XM_008522319.1	XM_008524566.1	NOT AVAILABLE
Heterocephalus glaber	XM_004840694.1	XM_004835258.2	XM_004840695.1	XM_004861131.2	XM_004862072.1	NOT AVAILABLE
Marmota martmota	XM_015506540.1	XM_015495124.1	XM_015487891.1	XM_015483063.1	XM_015483385.1	XM_015506937.1
Octodon degus	XM_004634250.1	XM_004625053.1	NOT AVAILABLE	XM_004641645.1	XM_004641134.1	NOT AVAILABLE
Ictidomys tridecemlineatı	XM_005320533.1	XM_005333842.1	NOT AVAILABLE	XM_005328685.2	XM_005329491.1	XM_005340614.2
Peromyscus maniculatus	XM_006984536.2	XM_006993911.2	XM_006984538.2	XM_006973335.1	XM_006988832.1	XM_006992461.2
Microtus ochrogaster	XM_005365608.2	XM_005364985.1	NM_001282253.1	NOT AVAILABLE	XM_005348325.1	XM_005371797.1
Ochotona princeps	XM_004585663.1	XM_004581281.1	XM_004585662.2	XM_004582990.1	XM_004578701.1	XM_004598852.1
