# Supplementary material for: Oxytocin and arginine vasopressin systems in the domestication process
Source: Genet Mol Biol. 2018 Mar 26;41(1 Suppl 1):235–42. doi: 10.1590/1678-4685-GMB-2017-0069 (PMC5913714; doi:10.1590/1678-4685-GMB-2017-0069)
Supplement: Supplementary file 2 [file 1415-4757-GMB-41-01-2017-0069-s005.pdf]

# Supplementary Material to "Oxytocin and Arginine Vasopressin Systems in the Domestication Process"

**Alignment analyses 1**. Alignment of the mammalian species used in the *AVP* analyses.

**Alignment analyses 2**. Alignment of the mammalian species used in the *OXT* analyses.

**Alignment analyses 3**. Alignment of the mammalian species used in the *AVPR1a* analyses.

**Alignment analyses 4**.Alignment of the mammalian species used in the *AVPR1b* analyses.

**Alignment analyses 5**. Alignment of the mammalian species used in the *AVPR2* analyses.

**Alignment analyses 6**. Alignment of the mammalian species used in the *OXTR* analyses.

Alignments (1-6) used in the analyses with the different species of wild and domesticated mammals

1. Alignment of the mammalian species used in the *AVP* analyses.

>Homo_sapiens

ATGCCTGACACCATGCTGCCCGCCTGCTTCCTCGGCCTACTGGCCTTCTCCTCCGCGTGC
TACTTCCAGAACTGCCCGAGGGGCGGCAAGAGGGCCATGTCCGACCTGGAGCTGAGACA
GTGCCTCCCCTGCGGCCCCGGGGGCAAAGGCCGCTGCTTCGGGCCCAGCATCTGCTGCG
CGGACGAGCTGGGCTGCTTCGTGGGCACGGCTGAGGCGCTGCGCTGCCAGGAGGAGAA
CTACCTGCCGTCGCCCTGCCAGTCCGGCCAGAAGGCGTGCGGGAGCGGGGGCCGCTGC
GCCGCCTTCGGCGTTTGCTGCAACGACGAGAGCTGCGTGACCGAGCCCGAGTGCCGCGA
G------
GGCTTTCACCGCCGCGCCCGCGCCAGCGACCGGAGCAACGCCACGCAGCTGGACGGGC
CGGCCGGGGCCTTGCTGCTGCGGCTGGTGCAGCTGGCCGGGGCGCCCGAGCCCTTCGA
GCCCGCCCAGCCCGACGCCTAC

>Pan_troglodytes

ATGCCCGACACCATGCTGCCCGCCTGCTTCCTCGGCCTACTGGCCTTCTCCTCCGCGTGC
TACTTCCAGAACTGCCCGAGGGGCGGCAAGAGGGCCGTGTCCGACCTGGAGCTGAGACA
GTGCCTCCCCTGCGGCCCCGGGGGCAAAGGCCGCTGCTTCGGGCCCAGCATCTGCTGCG
CGGACGAGCTGGGCTGCTTTGTGGGCACGGCTGAGGCGCTGCGCTGCCAGGAGGAGAA
CTACCTGCCGTCGCCCTGCCAGTCCGGCCAGAAGGCGTGCGGGAGCGGGGGCCGCTGC
GCCGCCTTCGGCGTTTGCTGCAACGACGAGAGCTGCACGACCGAGCCCGAGTGCCGCGA
G------
GGCTTTCACCGCCGCGCCCGCGCCAGCGACCGGAGCAACGCCACGCAGCTGGACGGGC
CGGCCGGGGCCTTGCTGCTGCGGCTGGTGCAGCTGGCCGGGGCGCCCGAGCCCTTCGA
GCCCGCCCAGCCCGACGTCTAC

>Cavia_porcellus

ATGCCTGACGCCATGCTGCCCGCCTGCCTCCTCGGCCTGCTGGCCTTCACCTCTGCCTGC
TATTTCCAGAACTGCCCGCGGGGTGGCAAGCGGGCCCTGTCTGATACGGAGCTGAGACA
GTGCCTCCCCTGCGGCCCCGGGGGCCAAGGGCGCTGCTTCGGGCCCAGCATCTGCTGC
GCCGACGCGCTGGGCTGCTTCGTGGGCACGGCCGAGGCGCTGCGCTGCCAGGAGGAGA
ACTACCTTCCGTCGCCCTGCCAGTCCGGCCAGAAGCCCTGCGGCAGCGGGGGGCGCTGC
GCCGCCAACGGCGTCTGCTGCAACGATGAGAGCTGCGTGATCGAGCCCGAGTGCCGAGA
G------
GAGTTCCACCGCCCCGTCCGTGCCGGCGACCGGAGCAACGTCACGCAGCTGGACGGCCC
CGCGGGGGCGCTGCTTCTCCGCCTGATGCAGCTGGCAGGGGCGCCCGAGCCC---
CAGCCCGCCGCGCCCGGCGGCTAC

>Rattus_norvegicus

ATGCTCAACACTACGCTCTCTGCTTGCTTCCTGAGCCTGCTGGCCCTCACCTCTGCCTGCT
ACTTCCAGAACTGCCCAAGAGGAGGCAAGAGGGCCACATCCGACATGGAGCTGAGACAGT
GTCTCCCCTGCGGCCCTGGCGGCAAAGGGCGCTGCTTCGGGCCGAGCATCTGCTGCGCG
GACGAGCTGGGCTGCTTCCTGGGCACCGCCGAGGCGCTGCGCTGCCAGGAGGAGAACTA
CCTGCCCTCGCCCTGCCAGTCTGGCCAGAAGCCTTGCGGAAGCGGAGGCCGCTGCGCTG
CCGCGGGCATCTGCTGCAGCGATGAGAGCTGCGTGGCCGAGCCCGAGTGTCGAGAG------
GGTTTTTTCCGCCTCACCCGCGCTCGGGAGCAGAGCAACGCCACGCAGCTGGACGGGCC
AGCCCGGGAGCTGCTGCTTAGGCTGGTACAGCTGGCTGGGACACAAGAGTCCGTGGATT
CTGCCAAGCCCCGGGTCTAC

>Mus_musculus

ATGCTCAACACTACGCTCTCCGCTTGTTTCCTGAGCCTGCTGGCCTTCTCCTCCGCCTGCT
ACTTCCAGAACTGCCCAAGAGGCGGCAAGAGGGCCATCTCTGACATGGAGCTGAGACAGT
GTCTCCCCTGCGGCCCGGGCGGCAAAGGACGCTGCTTCGGACCAAGCATCTGCTGCGCG
GACGAGCTGGGCTGCTTCGTGGGCACCGCCGAGGCGCTGCGCTGCCAGGAGGAGAACTA
CCTGCCCTCGCCCTGCCAGTCCGGCCAGAAGCCCTGCGGGAGCGGGGGCCGCTGCGCC
GCCGTGGGCATCTGCTGCAGCGACGAGAGCTGCGTGGCCGAGCCCGAGTGCCACGAC----
--
GGTTTTTTCCGCCTCACCCGCGCTCGGGAGCCAAGCAACGCCACACAGCTGGACGGCCC
TGCTCGGGCGCTGCTGCTAAGGCTGGTACAGCTGGCTGGGACACGGGAGTCCGTGGATT
CTGCCAAGCCCCGGGTCTAC

>Peromyscus_maniculatus

ATGCTCAACGCCACGCTGTCTGCTTGCTTCCTGAGCCTGCTGGCCTTCACCTCTGCCTGCT
ACTTCCAGAACTGCCCGAGAGGCGGCAAGAGGGCCATGTCTGACATGGAGCTGAGACAG
TGCCTCCCCTGCGGCCCTGGCGGCAAAGGGCGCTGCTTCGGGCCGAACATCTGCTGCGC
CGACGAGCTGGGCTGCTACGTGGGCACCGCCGAGGCGCTGCGCTGCCAGGAGGAGAAC
TACCTGCCCTCGCCCTGCCAGTCGGGCCAGAAGCCGTGCGGGAGCGGGGGCCGCTGCG
CCGCCGCGGGCGTCTGCTGCAGCCCCGAGAGCTGCGTGACCGAGCCCGAGTGTCGCGA
G------
GGTTTTCTGCGGCTCACCCGCGCCCGCGAGCCGAGCAACGGCACACAGCTGGACGGGCC
CGCCCGGGCGCTGCTGCTCCGGCTGGTGCAGCTGGCTGGGTCGCGGGAGGCCGTGGAT
TCTGCCAAGCCCCGGGGCTAC

>Microtus_ochrogaster

ATGCTCAACTCTACGCTGTCTGCGTGTTTCCTGAGCCTGCTGGCCTTCACCTCTGCCTGCT
ACTTCCAGAACTGCCCGAGAGGCGGCAAGAGGGCCATGCCTGACATGGAGCTGAGACAG
TGCCTCCCCTGTGGCCCCGGCGACCAAGGCCGCTGCTTCGGACCCAACATCTGCTGCGC
AGACGAGCTGGGCTGCTTCGTGGGCACCGCCGAGGCGCTGCGCTGCCAGGAGGAGAACT
ACCTGCCCTCGCCCTGCCAGTCGGGCCAGAAGCCGTGCGGGAGCGGGGGCCGCTGCGC
CGCCGCGGGCATCTGCTGCAGCGATGAGAGCTGCGTGACCGATCCCGAGTGCCGCGAG--
----
GATTTTCTCCGCCTCACCCGCGCTCGCGAACCGAACAACGGCACGCAGCTGGACGGGCC
CGCCCGAGCCCTGCTGCTCCGCCTGCTGCAGATGGCCGGGACGCGGGAGTCCGTGGATT
CTGCCAAGCCCCGGGTCTAC

>Ochotona_princeps

ATGCCTGACACTGTGCTACCCGTCTGCTTCCTGGGCCTGTTGGCCCTCACTTCTGCTTGCT
ACTTCCAGAACTGCCCGCGGGGTGGCAAGAGGGCCACATCTGACATGGAGCTGCGACAG
TGCCTCCCCTGCGGCCCCGGGGGCAAGGGCCGCTGCTTCGGGCCCAGCATCTGCTGCG
CCGACGAGCTGGGCTGCTTCGTGGGCACGGCCGAGGCGCTGCGCTGCCAGGAGGAGAA
CTTCCTGCCGTCGCCCTGCCAGTCGGGCCAGAAGCCGTGCGGGAGCGGGGGCCGCTGC
GCCGCCATCGGCATCTGCTGCAACGACGAGAGCTGCGTGACCGAGCCCGAGTGCCGCGA
G------
AGCTTTCCCCGCCGCGCCCGCGCCGGCGACCGCAGCAACGCCACGCGGCTGGACGCAC
CTGCAGGGGCCCTGCTGCTGCGGCTGGTGCAGCTGGCCGGGGCGCCCGAGCCCGCCCA
GCCCGCCCAGCCCGGCGGCTAC

>Oryctolagus_cuniculus

ATGCCCGACACCATGCTGCCTGTCTGCTTCCTCGGCCTGCTGGCCTGCGCCTCCGCTTGC
TACTTCCAGAACTGCCCAAGGGGCGGCAAGAGGGCCATGTCCGACCTGGAGCTGCGACA
GTGCCTCCCCTGCGGCCCCGCGGGCAAGGGCCGCTGCTTCGGGCCCAGCATCTGCTGC
GCCGACGAGCTGGGCTGCTTCGTGGGCACCGCCGAGGCGCTGCGCTGCCAGGAGGAGA
ACTTCCTGCCTTCGCCCTGCCAGTCGGGCCAGAAGCCGTGCGGGAGCGGGGGCCGCTGT
GCCGCCGCCGGCGTCTGCTGCAACGACGAGAGCTGCGTGACCGACCCCGAGTGCCGCG
AG------
GCCTTCCCCCGCCGCGCCCGCGCCAGCGACCGCAGCAACGCCACGCAGCTGGACGCGC
CCGCCGGGGCTCTGCTGCTGCGGCTGGTGCAGCTGGCGGGGGCGCCTGAGCCCGCAGA
GCCCGCCAAGCCCGGCGGCTAC

>Octodon_degus

ATGCCTGACACCATGCTGCCTGCCTGCTTCCTCGGCCTGCTGGCCTTCACCTCTGCCTGC
TACTTCCAGAATTGCCCGAGGGGTGGCAAGCGGGCCCTGGTGGACATGGAGCTGAGAGA
GTGCCTCCCCTGCGGCCCCGGGGGCCAAGGCCGCTGCTTCGGACCCAGCATCTGCTGCG
CCGACGCCCTGGGCTGCTTCGTGGGCACTGCCGAGGCGCTGCGCTGCCGCGAGGAGAA
CTTCCTGCCGTCGCCCTGCCAGTCGGGCCGGAAGCCCTGTGCCGGCGGGGGGCGCTGT
GCGGCCGACGGGGTCTGCTGCAACGATGAGAGCTGCGCGATGGAGCCCGAGTGCCGCG
AG------
AGCCTCCACCGCCGCGCCCGCGCCGGCGACCGGAGCAACAACGCGCAGCTGGACGGCC
CCGCGGGGGCGCTGTTGTACCGCCTGGTGCAGCTGGCGGGGGCGCCTGAGCCC---
GATCCCGCCTCTCCCGACGGCTAT

>Capra_aegagrus

ATGCCCGACGCCACACTGCCCGCCTGCTTCCTCGGCCTGCTGGCCTTCACCTCCGCTTGC
TACTTCCAGAACTGCCCAAGGGGCGGCAAGAGGGCCATGTCCGACCTGGAGCTGAGACA
GGGTATCCCCTGCGGCCCCGGGACCACCGGCCGCTGCTTCGGGCTCAGAATCTGCTGCA
AGGAAGGGGCAGACTGCTTCGTGGGCACGGCCCAGGGACGGCACCACCGTGAAGGGAA
CTACCTGCCGTCGCCCTGCCAATCCGGGAAGAAGCCCTGCGGGAGCGGAGCCCGCCCAG
CCGCCGCCGGCGTCTGCTACGGCGACGAGAGCTGCGTGACCGAGCCCGAGTGCCGGGA
AGGTATCGGCGCCCCCCACCCCACCCGCGCCAGCGACCGGAGCACCCCGCCCTCGCAG
CACGAAAAAAC---
GGTTTCAAAGGCACTGCTAGCGTACTTCTCTGCGGGGCGGAAGCCCGGGGAGCCCGCCC
GGCGGGGGATCTAC

>Capra_hircus

ATGCCCGACGCCACACTGCCCGCCTGCTTCCTCGGCCTGCTGGCCTTCACCTCCGCTTGC
TACTTCCAGAACTGCCCAAGGGGCGGCAAGAGGGCCATGTCCGACCTGGAGCTGAGACA
GTGTCTCCCCTGCGGCCCCGGGGGCAAAGGCCGCTGCTTCGGGCCCAGCATCTGCTGCG
GGGACGAGCTGGGCTGCTTCGTGGGCACGGCCGAGGCGCTGCGCTGCCAAGAGGAGAA
CTACCTGCCGTCGCCCTGCCAGTCCGGCCAGAAGCCCTGCGGGAGCGGGGGCCGCTGC
GCCGCCGCCGGCATCTGCTGTAACGACGAGAGCTGCGTGACCGAGCCCGAGTGCCGGGA
AGGTATCGGCTTCCCCCGCCGCGTCCGCGCCAGCGACCGGAGCAACGCGACCCTGCTGG
ACGGGCCGAGCGGGGCCTTGTTGCTGCGGCTGGTGCAGCTGGCGGCGGCGCCGGAGCC
CGCGGAGCCCGCCCAGCCCGGCGTCTAC

>Ovis_aries

ATGCCCGACGCCACACTGCCCGCCTGCTTCCTCGGCCTGCTGGCCTTCACCTCCGCTTGC
TACTTCCAGAACTGCCCAAGGGGCGGCAAGAGGGCCATGTCCGACCTGGAGCTGAGACA
GTGTCTCCCCTGCGGCCCCGGGGGCAAAGGCCGCTGCTTCGGGCCCAGCATCTGCTGCG
GGGACGAGCTGGGCTGCTTCGTGGGCACGGCCGAGGCGCTGCGCTGCCAAGAGGAGAT
CTACCTGCCGTCGCCCTGCCAGTCCGGCCAGAAGCCCTGCGGGAGCGGGGGCCGCTGC
GCCGCCGCCGGGATCTGCTGCAACGACGAGAGCTGCGTGACCGAGCCCGAGTGTCGGG
AAGGTATCGGCTTCCCCCGCCGCGTCCGCGCCAGCGACCGGAGCAACGCGACCCTGCTG
GACGGGCCGAGCGGGGCCTTGTTGCTGCGGCTGGTGCAGCTGGCGGCGGCGCCGGAGC
CCGCGGAGCCCGCCCAGCCCGGCGTCTAC

>Ovis_orientalis

ATGCCCGACGCCACACTGCCCGCCTGCTTCCTCGGCCTGCTGGCCTTCACCTCCGCTTGC
TACTTCCAGAACTGCCCAAGGGGCGGCAAGAGGGCCATGTCCGACCTGGAGCTGAGACA
GTGTCTCCCCTGCGGCCCCGGGGGCAAAGGCCGCTGCTTCGGGCCCAGCATCTGCTGCG
GGGACGAGCTGGGCTGCTTCGTGGGCACGGCCGAGGCGCTGCGCTGCCGAGAGGAGAA
CTACCTGCCGTCGCCCTGCCAGTCCGGCCAGAAGCCCTGCGGGAGCGGGGGCCGCTGC
GCCGCCGCCGGGATCTGCTGCAGCGCCGGGAGCTCGGCCACGGAGCCCGAGTGCCGGG
AAGGTATCGGCTTCCCCCGCCGCGTCCGCGCCAGCGACCGGAGCAACGCGACCCTGCTG
GACGGGCCGAGCGGGGCCTTGTTGCTGCGGCTGGTGCAGCTGGCGGCGGCGCCGGAGC
CCGCGGAGCCCGCCCAGCCCGGCGTCTAC

>Bos_primigenius

ATGCCCGACGCCACACTGCCCGCCTGCTTCCTCAGCCTGCTGGCCTTCACCTCTGCTTGC
TACTTCCAGAACTGCCCAAGGGGCGGCAAGAGGGCCATGTCCGACCTGGAGCTGAGA--
GTGTCTCCCCTGCGGCCCCGGGGGCAAAGGCCGCTGCTTCGGGCCCAGCATCTGCTGCG
GGGACGAGCTGGGCTGCTTCGTGGGCACGGCCGAGGCGCTGCGCTGCCAAGAGGAGAA
CTACCTGCCGTCGCCCTGCCAGTCCGGCCAGAAGCCCTGCGGGAGCGGGGGCCGCTGC
GCCGCCGCCGGCATCTGCTGCAACGATGAGAGCTGCGTGACCGAGCCCGAGTGCCGGGA
A------
GGCTTCCCCCGCCGCGTTCGCGCCAACGACCGGAGCAACGCGACCCTGCTGGACGGGC
CGAGCGGGGCCTTGTTGCTGCGGCTGGTGCAGCTGGCGGGGGCGCCGGAGCCCGCGGA
GCCCGCCCAGCCCGGCGTCTAC

>Bos_taurus

ATGCCCGACGCCACACTGCCCGCCTGCTTCCTCAGCCTGCTGGCCTTCACCTCTGCTTGC
TACTTCCAGAACTGCCCAAGGGGCGGCAAGAGGGCCATGTCCGACCTGGAGCTGAGACA
GTGTCTCCCCTGCGGCCCCGGGGGCAAAGGCCGCTGCTTCGGGCCCAGCATCTGCTGCG
GGGACGAGCTGGGCTGCTTCGTGGGCACGGCCGAGGCGCTGCGCTGCCAAGAGGAGAA
CTACCTGCCGTCGCCCTGCCAGTCCGGCCAGAAGCCCTGCGGGAGCGGGGGCCGCTGC
GCCGCCGCCGGCATCTGCTGCAACGATGAGAGCTGCGTGACCGAGCCCGAGTGCCGGGA
A------
GGCTTCCCCCGCCGCGTTCGCGCCAACGACCGGAGCAACGCGACCCTGCTGGACGGGC
CGAGCGGGGCCTTGTTGCTGCGGCTGGTGCAGCTGGCGGGGGCGCCGGAGCCCGCGGA
GCCCGCCCAGCCCGGCGTCTAC

>Sus_scrofa

ATGCCTGACGCCACTCTGCCCGCCTGCTTCCTTGGCCTGCTGGCCCTCACCTCCGCTTGC
TACTTCCAGAACTGCCCGAAGGGAGGCAAGAGGGCCATGTCCGACTTGGAGCTGAGACA
GGTAAGAACCTGTGGCCCCAGGGCTGCCGGGCACTGCTGCGGGCCGAGTATCTGCTGCG
GGGATGAGCTGGGCTGCTTCGTGGGCACAGCCGAGGCGCTGCGCTGCCAGGAGGAGAA
CTACCTGCCGTCGCCCTGCCAGTCCGGTCAGAAACCTTGCGGGAGCGAGGGCCGCTGCG
CCGCCGCCGGCATCTGCTGCAACCCTGGTGAGT-
CGGGACGGGAAGGGACCGAGGGGAGGGCGCCAGCTTTCCCCGCCGCGCCCGCGCCAGC
GACCGGAGCAACGCGACCCTGCTGGACGGGCCGAGCGGGGCTCTGCTGCTGCGGCTGG
TGCAACTGGCGGGGGCGCCCGAGCCCGCGGAGCCCGCCCAGCCCGGCGTTTAC

>Sus_scrofa_familiaris

ATGCCTGACGCCACTCTGCCCGCCTGCTTCCTCGGCCTGCTGGCCCTCACCTCCGCTTGC
TACTTCCAGAACTGCCCGAAGGGAGGCAAGAGGGCCATGTCCGACTTGGAGCTGAGACA
GTGCCTCCCCTGCGGCCCCGGGGGTAAAGGTCGCTGCTTCGGGCCCAGTATCTGCTGCG
GGGATGAGCTGGGCTGCTTCGTGGGCACGGCCGAGGCGCTGCGCTGCCAGGAGGAGAA
CTACCTGCCGTCTCCCTGCCAGTCGGGCCAGAAGCCGTGCGGGAGCGGGGGCCGCTGC
GCCGCCGCCGGCATCTGCTGCAACGACGAGAGCTGCGTGACCGAGCCCGAGTGCCGGG
AG------
GGCTTTCTCCGCCGCGCCCGCGCCAGCGACCGGAGCAACGCGACCCTGCTGGACGGGC
CGAGCGGGGCTCTGCTGCTGCGGCTGGTGCAACTGGCGGGGGCGCCCGAGCCCGCGGA
GCCCGCCCAGCCCGGCGTTTAC

>Equus_caballus

ATGCCTGACACCATGCTGCCCGCCTGCTTTCTCGGCCTGCTGGCCTTTACCTCCGCTTGCT
ACTTCCAGAACTGCCCAAGGGGTGGCAAGAGGGCCATGTCCGACCTGGAGCTGAGACAG
TGCCTCCCCTGCGGCCCCGGGGGCAAAGGGCGCTGCTTCGGGCCCAGCATCTGCTGCG
GGGACGAGCTGGGCTGCTTCGTGGGCACAGCCGAGGCGCTGCGCTGCCAGGAGGAGAA
CTACCTGCCGTCGCCCTGCCAGTCGGGCCAGAAGCCTTGCGGGAGCGGGGGCCGCTGC
GCCGCCGCCGGCATCTGCTGCAACGACGAGAGCTGCGTGACAGAACCCAATTGCCGA-----
------------------------------------------------------------------------------------------------------------------------------ -------
---------------

>Ancient_horse

CTGCCCGACACCATGCTGCCCGCCTGCTTTCTCGGCCTGCTGGCCTTTACCTCCGCTTGC
TACTTCCAGAACTGCCCAAGGGGTGGCAAGAGGGCCATGTCCGACCTAGACCCTCGCCAG
TGCCTCCCCTGCGGCCCCGGGGGCAAAGGGCGCTGCTTCGGGCCCAGCATCTGCTGCG
GGGACGAGCTGGGCTGCTTCGTGGGCACGGCCGAGGCGCTGCGCTGCCAGGAGGAGAA
CTACCTGCCGTCGCCCTGCCAGTCGGGCCAGAAGCCCTGCGGGAGCGGGGGCCGCTGC
GCCGCCGCCGGCATCTGCTGCAACGACGCGAGTTGGGGCGTCGGGAAAGAGTGGAGAG
A-------------------------------------------------------------------------------------------------------------------------------------
--------------------

>Felis_catus

ATGCCGGACACCATGCTACCCGCCTGCTTCCTGGGCCTGCTGGCCTTCACCTCCGCCTGC
TACTTCCAGAACTGCCCGAGGGGTGGCAAGAGGGCCATGTCTGACCTGGAGCTGAGACA
GTGCCTCCCCTGCGGCCCCGAGGGCAAAGGGCGCTGCTTCGGGCCCAGCATCTGCTGCG
GCGACGAGCTCGGCTGCTTCGTGGGCACGGCCGAGGCGCTGCGCTGCCAGGAGGAGAA
CTACCTGCCGTCGCCCTGCCAGTCGGGCCACAAGCCGTGCGGGAGCGGGGGCCGCTGC
GCCGCCGCGGGGATCTGCTGCAACGACGGGGGATGTGTGGCCGTGGACCTTC--------------
------------------------------------------------------------------------------------------------------------------------ --------------
----------

>Felis_silvestris

ATGCCGGACCCCATGCTACCCGCCTGCTTCCTGGGCCTGCTGGCCTTCACCTCCGCCTGC
TACTTCCAGAACTGCCCGAGGGGTGGCAAGAGGGCCATGTCTGACCTGGAGCTGAGACA
GG--
CTCCCCTGTGGCCCCGAGGGCAAAGGGCGCTGCTTCGGGCCCAGCAGCTGCTGCGGCG
ACGAGCTCGGCTGCTTCGTGGGCACGGCAGACGCGCCGCGCGGGCAGGGGGAGCACCG
CCCGCCGCCGCCCTGCCACTCGGGCCACAAGCCGTGCGGGAGCGGGGGCCGCTGCGCC
GCCGCGGGGATCTGCTGCAACGACGGGAGCTGCGCTGCCGGGGCGGCCCGGGGGC------
------------------------------------------------------------------------------------------------------------------------------------ --
-------------

>Mustela_putorius_furo

ATGCCGGACGCCATGCTCCCTGCCTGCTTCCTGGGCCTGCTGGCCTTCACCTCCGCCTGC
TACTTCCAGAACTGCCCTCGGGGCGGCAAGAGGGCCCTGTCTGACTTGGAGCTGAGACA
GTGTCTCCCCCTAGGCCCCGGGGGCAAATGCCGCTGCTTCGGGCCCAGCATCTGCTGCG
GCGACGAGCTGGGCTGCTTCGTGGGCACGGCCGAGGCGCTGCGCTGCCAGGAAGAGAA
CTACCTGCCGTCGCCTTGCCAGTCCGGCCACCAGCCATGCGGGAGCGGGGGCCGCTGC
GCCGCCGCCGGCATCTGCTGCAACGATGAGAGCTGCGTGACCGACCCCGAGTGCCGGG
GG------
GGCTTCCACCGCCGTGCCCGTGCCAGCGACCGAAGCAATGCGACCGAGCTGGACGGGCC
GACCGGCGCCTTGCTGCTGCGGCTGGTGCAGCTGGCGGGGGCGCCCGAGCCCGCGGAG
CCCGCCCGGCCCGGCGTCTAC

>Odobenus_rosmarus

ATGCCGGACACCATGCTCCCTGCCTGCTTGCTGGGCCTGCTGGCCTTCACCTCCGCCTGC
TACTTCCAGAACTGCCCGCGGGGCGGCAAGAGGGACTTGTCTGACCTGGAGCTGAGACA
GTGTCTCCCCTGCGGCCCCGGGGGCAAAGGGCGCTGCTTCGGGCCCAGCATCTGCTGCG
GCGACGAGCTGGGCTGCTTCGTGGGCACGGCCGAGGCGCTGCGCTGCCAGGAGGAGAA

CTACCTGCCGTCGCCCTGCCAGTCGGGCCACCAGCCGTGCGGGAGCGGGGGCCGCTGC
GCCGCCGCCGGCATCTGCTGCAGCCCGGAGAGCTGCGTGACCGAGCCCGAGTGCCGGG
GG------
GGCTTCCACCGCCGCGCCCGCGCCAGCGACCGGAGCAACGCGACCCAGCTGGACGGGC
CGACCGGCGCCTTGCTGCTGCGGCTGGTGCAGCTGGCGGGGGCGCCCGAGCCCGCGGA
GCCCGCCCCGCCCGGCGTCTAC

>Canis_lupus

ATGCCGGACACCATGCTGCCCGCCTGCTTCCTGGGCCTGCTGGCCTTCACCTCCGCCTGC
TACTTCCAGAACTGTCCTAGGGGTGGCAAGAGGGCCATGTCTGATCTGGAGCTGAGACAG
GTACGACCCTGTGGCCCCGAGGGCAAAGGGCGCTGCTTCGGGCCCAGCATCTGCTGCGG
CGACGAGCTGGGCTGCTTCCTGGGCACCGCCGAGGCGCTGCGCTGCCAGGAGGAGAACT
ACCTGCCTTCGCCCTGCCAGTCGGGCCGCACGCCGTGCGGGAGCGGGGGTCGCTGCGC
CGCCGCCGGCATCTGCTGCAACGACGGGAGCTGCGTGACCGAGCCCGAGTCCCGGGAG-
-----
GGTTTGCACCGCGGCGCCCGCGCCAGCGACCGGAGCAACGCGACCCAGTTGGACGGGC
CGACGGGAGCCTTGCTGCTGCGGCTGGTGCAGCTGGCGGGGGCGCCCGAGCCCGCGGA
GCCCGCGCCGCCCGGGGTTTAT

>Canis_lupus_familiaris

ATGCCGGACACCATGCTGCCCGCCTGCTTCCTGGGCCTGCTGGCCTTCACCTCCGCCTGC
TACTTCCAGAACTGTCCTAGGGGTGGCAAGAGGGCCATGTCTGATCTGGAGCTGAGACAG
TGCCTCCCCTGCGGCCCCGGGGGCAAAGGGCGCTGCTTCGGGCCCAGCATCTGCTGCG
GCGACGAGCTGGGCTGCTTCGTGGGCACGGCCGAGGCGCTGCGCTGCCAGGAGGAGAA
CTACCTGCCGTCGCCCTGCCAGTCGGGCCGCACGCCGTGCGGGAGCGGGGGTCGCTGC
GCCGCCGCCGGCATCTGCTGCAACGACGAGAGCTGCGTGACCGAGCCCGAGTCCCGGG
AG------
GGTTTGCACCGCGGCGCCCGCGCCAGCGACCGGAGCAACGCGACCCAGTTGGACGGGC
CGACGGGAGCCTTGCTGCTGCGGCTGGTGCAGCTGGCGGGGGCGCCCGAGCCCGCGGA
GCCCGCGCCGCCCGGGGTTTAT

2. Alignment of the mammalian species used in the *OXT* analyses.

>Homo_sapiens

ATGGCCGGCCCCAGCCTCGCTTGCTGTCTGCTCGGCCTCCTGGCGCTGACCTCCGCCTG
CTACATCCAGAACTGCCCCCTGGGAGGCAAGAGGGCCGCGCCGGACCTCGACGTGCGCA
AGTGCCTCCCCTGCGGCCCCGGGGGCAAAGGCCGCTGCTTCGGGCCCAATATCTGCTGC
GCGGAAGAGCTGGGCTGCTTCGTGGGCACCGCCGAAGCGCTGCGCTGCCAGGAGGAGA
ACTACCTGCCGTCGCCCTGCCAGTCCGGCCAGAAGGCGTGCGGGAGCGGGGGCCGCTG
CGCGGTCTTGGGCCTCTGCTGCAGCCCGGACGGCTGCCACGCCGACCCTGCCTGCGACG
CGGAAGCCACCTTCTCCCAGCGC

>Pan_troglodytes

ATGGCCGGCCCCAGCCTCGCTTGCTGTCTGCTCGGCCTCCTGGCGCTGACCTCCGCCTG
CTACATCCAGAACTGCCCCCTGGGAGGCAAGAGGGCCGCGCCGGACCTCGACGTGCGCA
AGTGCCTCCCCTGCGGCCCCGGGGGCAAAGGCCGCTGCTTCGGGCCCAATATCTGCTGC
GCAGAAGAGCTGGGCTGCTTCGTGGGCACCGCCGAAGCGCTGCGCTGCCAGGAGGAGAA
CTACCTGCCGTCGCCCTGCCAGTCCGGCCAGAAGGCGTGCGGGAGCGGGGGCCGCTGC
GCGGTCTTGGGCCTCTGCTGCAGCCCGGACGGCTGCCACGCCGACCCTGCCTGCGACAT
GGAAGCTACCTTCTCCCAGCAC

>Mus_musculus

ATGGCCTGCCCCAGTCTCGCTTGCTGCCTGCTTGGCTTACTGGCTCTGACCTCGGCCTGC
TACATCCAGAACTGCCCCCTGGGCGGCAAGAGGGCTGTGCTGGACCTGGATATGCGCAA
GTGTCTCCCCTGCGGCCCGGGCGGCAAAGGACGCTGCTTCGGACCAAGCATCTGCTGCG
CGGACGAGCTGGGCTGCTTCGTGGGCACCGCCGAGGCGCTGCGCTGCCAGGAGGAGAA
CTACCTGCCTTCGCCCTGCCAGTCTGGCCAGAAGCCCTGCGGGAGCGGAGGCCGCTGCG

CCGCCACAGGCATCTGCTGCAGCCCGGATGGCTGCCGCACAGACCCCGCCTGCGACCCT
GAGTCTGCCTTCTCGGAGCGC

>Rattus_norvegicus

ATGGCCTGCCCCAGTCTTGCTTGCTGCCTGCTTGGCCTACTGGCTCTGACCTCCGCCTGC
TACATCCAGAACTGCCCCCTGGGCGGCAAGAGGGCTGCGCTAGACCTGGATATGCGCAA
GTGTCTTCCCTGCGGACCCGGCGGCAAAGGGCGCTGCTTCGGGCCGAGCATCTGCTGCG
CGGACGAGCTGGGCTGCTTCGTGGGCACCGCCGAGGCGCTGCGCTGCCAGGAGGAGAA
CTACCTGCCCTCGCCCTGCCAGTCTGGCCAGAAGCCTTGCGGAAGCGGAGGCCGCTGCG
CCACCGCGGGCATCTGCTGTAGCCCGGATGGCTGCCGCACCGACCCCGCCTGCGACCCT
GAGTCTGCCTTCTCCGAGCGC

>Microtus_ochrogaster

ATGGCCTGCCCCAGTCTCGCCTGCTGCCTACTTGGCCTCCTGGCTCTGACCTCCGCCTGC
TACATCCAGAACTGTCCCCTGGGCGGCAAGAGGGCTGCGCTGGACTTGGACACGCGCAA
GTGCCTCCCCTGCGGTCCTGGCGGCAAAGGCCGCTGCTTCGGACCCAACATCTGCTGCG
CCGATGAGCTGGGCTGTTTCGTGGGCACCGCCGAGGCGCTGCGCTGCCAGGAGGAGAAC
TACCTGCCCTCGCCCTGCCAGTCGGGCCAGAAGCCGTGCGGGAGCGGGGGCCGCTGCG
CCGCCGCGGGTGTCTGCTGCAACCCGGATGGCTGCCGCATGGACCCCGCCTGCGACCCT
GAGTCTGCTTTCTCCGAGCGC

>Mesocricetus_auratus

ATGGCCTGCCCCAGTCTCGCTTGCTGCCTGCTTGGCTTACTGGCTCTGACCTCGGCCTGC
TACATCCAGAACTGTCCCCTGGGCGGCAAGAGGGCTGTGCTGGACGTGGACATTCGCAAG
TGCCTTCCCTGCGGCCCCAATGGGAGAGGGAGATGCTTCGGGCCTAACATATGCTGCTCT
GAGGAGCTGGGGTGCCTCATCAGCACCTTGGAGGCACTGCCCTGCCTGGAGGAGAACAG
GCTGCCCACACCCTGCCAGTCAGGTTGGAAGCCCTGCAGGAACTCGGGCTACTGTGCCA
CCCAGGACTTGTGCTGCACTACAGAGGATTGCTTCATGGATTCCACCTGCAACCCTGGGT
CTGCCTTCTCCAAG---

>Peromyscus_maniculatus

ATGGCCTGCTCCAGTCTCGCTTGCTGCCTCCTTGGCCTGCTGGCTCTGACCTCCGCCTGC
TACATCCAGAACTGTCCCCTGGGCGGCAAGAGGGCTGTGCTGGACCTGGACATGCGCAA
GTGCCTCCCCTGCGGCCCCGGCGGCAAAGGGCGCTGCTTCGGGCCGAACATCTGCTGCG
CCGACGAGCTGGGCTGCTACGTGGGCACCGCCGAGGCGCTGCGCTGCCAGGAGGAGAA
CTACCTGCCCTCGCCCTGCCAGTCGGGCCAGAAGCCGTGCGGGAGCGGGGGCCGCTGC
GCCGCCGCGGGCGTCTGCTGCAGCCCCGATGGCTGCCGCATGGACCCCGCCTGCGACC
CTGAGTCTGCCTTCTCCGAGCAC

>Ictiodomys_tridecemlineatus

ATGGCCAGCCTCAGCCTCCCCTGCTGCCTGCTGGGCCTCCTGGCTCTGACCTCTGCCTGC
TACATCCAGAACTGCCCCCTGGGAGGCAAGAGGGCCGTGCTGGAGCTGGACGTGCGCAA
GTGCCTCCCCTGCGGCCCCGGTGGCCAAGGGCGCTGCTTCGGGCCCAGCATCTGCTGCG
GGGACGAGCTGGGCTGCTTCGTGGGCACAGCTGAGGCGCTGCGCTGCCAGGAGGAGAA
CTACCTGCCATCGCCCTGCCAGTCGGGCCAGAAGCCCTGCGGGAGCGGGGGCCGGTGC
GCGGCCGCCGGCTTCTGCTGCAGCTCAGATGGCTGCCGCACCGACCCCGCTTGCGACCC
CGAGACCAACTTTTCTGAA---

>Octodon_degus

ATGACCGGGCCCAGCCTCACTTGCTGCCTGCTCGGCCTCCTATCGCTGACCTCGGCCTGC
TACATCCAGAACTGTCCCCTGGGAGGCAAGCGGGCCACGCTGGACCTCGATGTGCGCAA
GTGCCTCCCCTGCGGCCCCGGGGGCCAAGGCCGCTGCTTCGGACCCAGCATCTGCTGCG
CCGACGCCCTGGGCTGCTTCGTGGGCACCGCCGAGGCGCTGCGCTGCCGCGAGGAGAA
CTTCCTGCCGTCGCCCTGCCAGTCGGGCCGGAAGCCCTGTGCCGGCGGGGGGCGCTGC
GCGGCCGACGGGGTCTGCTGCAGTTCGGACGGCTGCCGCACCGACCCCGCCTGCGACG
CGGAGGCCGCCTTCTCGGAGCGC

>Cavia_porcellus

ATGGCCGGCCCCAGCCTCGCCTGCTGCCTGCTCGGCCTCCTGGCGCTGACCTCTGCCTG
CTACATCCAGAACTGTCCCCTGGGCGGCAAGAGGGCCGCGCTGGACCTCGATGTGCGCA
AGTGCCTCCCCTGCGGCCCCGGGGGCCAAGGGCGCTGCTTCGGGCCCAGCATCTGCTGC
GCCGACGCGCTGGGCTGCTTCGTGGGCACGGCCGAGGCGCTGCGCTGCCAGGAGGAGA
ACTACCTTCCGTCGCCCTGCCAGTCCGGCCAGAAGCCCTGCGGCAGCGGGGGGCGCTGC
GCCGCCAACGGCGTCTGCTGCAACGATGACGGCTGCCGCATCGACCCTGCCTGCGACTC
TGAGGCCGCCTTCGCCGAGCGC

>Ochotona_princeps

ATGGCCGGCCCCAGCCTCGCCTGCTGCCTGCTCGGCCTCCTGGCCCTGACTTCGGCCTG
CTACATCCAGAACTGTCCCCTGGGCGGCAAGAGGGCTGCGCTGGACCGCGACGTGCGCA
AGTGCCTCCCCTGCGGCCCCGGGGGCAAGGGCCGCTGCTTCGGGCCCAGCATCTGCTGC
GCCGACGAGCTGGGCTGCTTCGTGGGCACGGCCGAGGCGCTGCGCTGCCAGGAGGAGA
ACTTCCTGCCGTCGCCCTGCCAGTCGGGCCAGAAGCCGTGCGGGAGCGGGGGCCGCTG
CGGGGCCGCCGGCGTCTGCTGCAACGCAGATGGCTGCCGCGCCGACCCCACTTGTGATC
TCGAGGTCGCCTTCCCCGAGCGC

>Oryctolagus_cuniculus

ATGGCCGGCCCCAGCCTCGCCTGCTGCCTGCTCGGCCTCCTGGCGCTGACCTCGGCCTG
CTACATCCAGAACTGCCCTCTGGGCGGCAAGAGGGCCGCGCTGGACCGCGACGTGCGCA
AGTGCCTCCCCTGCGGCCCCGCGGGCAAGGGCCGCTGCTTCGGGCCCAGCATCTGCTGC
GCCGACGAGCTGGGCTGCTTCGTGGGCACCGCCGAGGCGCTGCGCTGCCAGGAGGAGA
ACTTCCTGCCTTCGCCCTGCCAGTCGGGCCAGAAGCCGTGCGGGAGCGGGGGCCGCTGT
GCCGCCGCCGGCGTCTGCTGCAGCGCCGACGGCTGCCGCACCGATCCCACCTGCGACC
CCGAGGCCGCCTTCTCCGAGCGC

>Capra_hircus

ATGGCAGGTTCCAGCCTCGCCTGCTGCCTGCTCGGCCTCCTGGCGTTGACCTCCGCCTGC
TACATTCAGAACTGCCCCCTGGGCGGCAAGCGCGCGGTGCTGGACCTCGACGTGCGCAC
GTGTCTCCCCTGCGGCCCCGGGGGCAAAGGCCGCTGCTTCGGGCCCAGCATCTGCTGCG
GGGACGAGCTGGGCTGCTTCGTGGGCACGGCCGAGGCGCTGCGCTGCCAAGAGGAGAA
CTACCTGCCGTCGCCCTGCCAGTCCGGCCAGAAGCCCTGCGGGAGCGGGGGCCGCTGC
GCCGCCGCCGGCATCTGCTGCAGCCCGGACGGCTGCCACGCGGATCCCGCCTGCGACC
CCGAGGCCGCCTTCTCCCAGCAC

>Capra_aegagrus

ATGCCGGATTCCAGGCGGGCCCG-
GGCCAGGGTGGCCCCTCGGCGTTGACCTCTGCCTGCTACATGCAGAACTGCCCCCTGGG
CGGCTGCCACGCGGTGCTGGACCTCGACGTGCGCACGTATCCCGCCTGCGACCCCGGG
GGCAAAGGCCGCCTTCTCGGGCCCAGCATCTGCTGC---
GACCGGCCGGCCTGCTTCGTGGGCACGGCCGAGGCGCTGCGCTGCCAAGAGGAGAACT
ACCTGACACCATCCTGCCAGTCCGGCCAGAAGCCCTGCGGGAGCGCAACCCTCACTCCCT
CTGTAATCATCCGCTGCAGCCAGGACTTA---CAA---
AATAAAGCCTTTTTTTCCGAGGCCGCCTCCAACAAGCGC

>Ovis_orientalis

ATGGCAGGTTCCAGCCTCGCCTGCTGCCTGCTCGGCCTCCTGGCGTTGACCTCCGCCTGC
TACATTCAGAACTGCCCCCTGGGCGGCAAGCGTGCGGTGCTGGACCTCGACGTGCGCAC
GGGGCGCCCCTGCGGCCCCGGGGGCAAAGGCCGCTGCTTCGGGCCCAGCATCTGCTGC
GGGGACGAGCTGGGCTGCTTCGTGGGCACGGCCGAGGCGCTGCGCTGCCGAGAGGAGA
ACTACCTGCCGTCGCCCTGCCAGTCCGGCCAGAAGCCCTGCGGGAGCGGGGGCCGCTG
CGCCGCCGCCGGGATCTGCTGCAGCCCGGGTGAGTCCCACGGGGGCCGAGACGGGGCC
GGGGAGGCCGCCTTCTCCCAGCAC

>Ovis_aries

ATGGCAGGTTCCAGCCTCGCCTGCTGCCTGCTCGGCCTCCTGGCGTTGACCTCCGCCTGC
TACATTCAGAACTGCCCCCTGGGCGGCAAGCGTGCGGTGCTGGACCTCGACGTGCGCAC
GTGTCTCCCCTGCGGCCCCGGGGCGAAAGGCCGCTGCTTCGGGCCCAGCATCTGCTGCG

GGGACGAGCTGGGCTGCTTCGTGGGCACGGCCGAGGCGCTGCGCTGCCGAGAGGAGAA
CTACCTGCCGTCGCCCTGCCAGTCCGGCCAGAAGCCCTGCGGGAGCGGGGGCCGCTGC
GCCGCCGCCGGGATCTGCTGCAGCCCGGACGGCTCGCACGCGGATCCTGCCTGCGACC
CCGAGGCCGCCTTCTCCCAGCAC

>Bos_taurus

ATGGCAGGTTCCAGCCTCGCCTGCTGCCTGCTCGGCCTCCTGGCGTTGACCTCCGCCTGC
TACATTCAGAACTGCCCCCTGGGCGGCAAACGCGCGGTGCTGGACCTCGACGTGCGCAC
GTGTCTCCCCTGCGGCCCCGGGGGCAAAGGCCGCTGCTTCGGGCCCAGCATCTGCTGCG
GGGACGAGCTGGGCTGCTTCGTGGGCACGGCCGAGGCGCTGCGCTGCCAAGAGGAGAA
CTACCTGCCGTCGCCCTGCCAGTCCGGCCAGAAGCCCTGCGGGAGCGGGGGCCGCTGC
GCCGCCGCCGGCATCTGCTGCAGCCCGGACGGCTGCCACGAGGACCCCGCCTGCGACC
CTGAGGCCGCCTTCTCCCAGCAC

>Bos_primigenius

ATGGCAGGTTCCAGCCTCGCCT-
CTGCCTGCTCGGCCTCCTGGCGTTGACCTCCGCCTGCTACATTCAGAACTGCCCCCTGGG
CGGCAAACGCGCGGTGCTGGACCTCGACGTGCGCAAGTGTCTCCCCTGCGGCCCCGGG
GGCAAAGGCCGCTGCTTCGGGCCCAGCATCTGCTGCGGGGACGAGCTGGGCTGCTTCGT
GGGCACGGCCGAGGCGCTGCGCTGCCAAGAGGAGAACTACCTGCCGTCGCCCTGCCAGT
CCGGCCAGAAGCCCTGCGGGAGCGGGGGCCGCTGCGCCGCCGCCGGCATCTGCTGCAG
CCCGGACGGCTGCCACGAGGACCCCGCCTGCGACCCTGAGGCCGCCTTCTCCCAGCAC

>Sus_scrofa

ATGGCCGGACCCAGCCTTGCCTGCTGCCTGCTCGGCCTCCTGGCGCTGACCTCCGCCTG
CTACATCCAGAACTGCCCCCTGGGCGGCAAGAGGGCCGTGCTGGACCTCGACGTCCGCC
AGTGCCTCCCCTGCGGCCCCGGGGGCAAAGGCCGCTGCTTCGGGCCCAGCATCTGCTGC
GGGGATGAGCTGGGCTGCTTCGTGGGCACAGCCGAGGCGCTGCGCTGCCAGGAGGAGA
ACTACCTGCCGTCGCCCTGCCAGTCCGGTCAGAAACCTTGCGGGAGCGAGGGCCGCTGC
GCCGCCGCCGGCATCTGCTGCAACCCTGGTGACTGCCGCGTCGGGACGGGAAGGGACC
GAGGGATCACCTTTTCCCAGCGC

>Sus_scrofa_familiaris

ATGGCCGGACCCAGCCTTGCCTGCTGCCTGCTCGGCCTCCTGGCGCTGACCTCCGCCTG
CTACATCCAGAACTGCCCCCTGGGCGGCAAGAGGGCCGTGCTGGACCTCGACGTGCGCA
AGTGCCTCCCCTGCGGCCCCGGGGGCAAAGGCCGCTGCTTCGGGCCCAGCATCTGCTGC
GGGGATGAGCTGGGCTGCTTCGTGGGCACAGCCGAGGCGCTGCGCTGCCAGGAGGAGA
ACTACCTGCCGTCGCCCTGCCAGTCCGGTCAGAAACCTTGCGGGAGCGAGGGCCGCTGC
GCCGCCGCCGGCATCTGCTGCAACCCTGACGGCTGCCGCTTCGACCCCGCCTGCGACCC
CGAAGCCACCTTTTCCCAGCGC

>Equus_caballus

-----------------------------------------------------------------
TGCTACATCCAGAACTGCCCCCTGGGCGGCAAGAGGGCCGCGCTGGACCTCGACGTGCG
CAAGTGCCTCCCCTGCG-CCCCGGGGGCAAAGGGCTCTGTTACGGGCCCAGTATCTGC----
-------------------------------------------------------------------------------------------------------------------------------------------- --
--------------------------------------------------------------------------------

>Ancient_horse

---------------------------------------------------------------------------------
TGCCCCCTGGGCGGCAAGAGGGCCGCGCTGGACCTCCCCTCCCGCCAGTGCCTCCCCTG
CGGCCCCGGGGGCAAAGGGCGCTGCTTCGGGCCCAGCATCTGCTGCGGCGACGAGCTG
GGCTGCTTCGTGGGCACGGCCGAGGCGCTGCGCTGCCAGGAGGAGAACTACCTGCCGTC
GCCCTGCCAGTCGGA-----------------------------------------------------------------------------------------------------------
-------------------

>Mustela_putorius_furo

ATGGCTGGCCCCAGCCTCGCCTGCTGCCTGCTGGGCCTCCTCGCCCTGACCTCCGCCTG
CTACATCCAGAACTGCCCCCTGGGCGGCAAGAGGGCTGCGCTGGACCTCGATGTGCGCC
AGTGTCTCCCTTGCGGCCCTGGGGGCAAAGGGCGCTGCTTCGGGCCCAGCATCTGCTGC
GGCGACGAGCTGGGCTGCTTCGTGGGCACCAACGAGGCGCTGCGCTGCCAGGAGGAGA
ACTACCTGCCGTCGCCCTGCCAGTCGGGCCACCAGCCGTGCGGGAGCGGGGGCCGCTG
CGCCGCCGCCGGCATCTGCTGCAGCCCGGACGGCTGCCGCGCCGACCCCGCCTGCGAC
CCCGAGGCCGCCTTTTCCCAGCGC

>Odobenus_rosmarus

ATGGCCGGCCCCGGCCTCGCCTGCTGCCTGCTGGGCCTCCTGGCGCTCACCTCCGCCTG
CTACATCCAGAACTGCCCCCTGGGCGGCAAGAGGGCCGCGCTGGACCTCGACGTGCGCC
AGTGTCTCCCCTGCGGCCCCGGGGGCAAAGGGCGCTGCTTCGGGCCCAGCATCTGCTGC
GGCGACGAGCTGGGCTGCTTCGTGGGCACGGCCGAGGCGCTGCGCTGCCAGGAGGAGA
ACTACCTGCCGTCGCCCTGCCAGTCGGGCCACCAGCCGTGCGGGAGCGGGGGCCGCTG
CGCCGCCGCCGGCATCTGCTGCAGCCCGGACGGCTGCCGCGCCGAGCCCGCCTGCGAC
CCCGAGGCCGCCTTCTCCCAGCGC

>Canis_lupus

ATGGCCGGGCCCGGCCTCGCCTGCTGCCTGCTCGGCCTCCTGGCGCTGACCTCCGCCTG
CTACATCCAGAACTGCCCCCTGGGCGGCAAGAGGGCCGCGCTGCTCCCGCCCTCCCGCC
AGTGCCTCCCCTGCGGCCCCGGGGGCAAAGGGCGCTGCTTCGGGCCCAGCATCTGCTGC
GGCGACGAGCTGGGCTGCTTCGTGGGCACGGCCGAGGCGCTGCGCTGCCAGGAGGAGA
ACTACCTGCCGTCGCCCTGCCAGTCGGGCCGCACGCCGTGCGGGAGCGGGGGTCGCTG
CGCCGCCGCCGGCATCTGCTGCATCCCAGACGGCTGCCGCGCCGACCCCGCCTGCGAC
CCCGACGCCGCCTTCTCCCAGCGC

>Canis_lupus_familiaris

ATGGCCGGCCCCGGCCTCGCCTGCTGCCTGCTCGGCCTCCTGGCGCTGACCTCCGCCTG
CTACATCCAGAACTGCCCCCTGGGCGGCAAGAGGGCCGCGCTGGACCTCGACGTGCGCC
AGTGCCTCCCCTGCGGCCCCGGGGGCCAAGGGCGCTGCTTCGGGCCCAGCATCTGCTGC
GGCGACGAGCTGGGCTGCTTCGTGGGCACGGCCGAGGCGCTGCGCTGCCAGGAGGAGA
ACTACCTGCCGTCGCCCTGCCAGTCGGGCCGCACGCCGTGCGGGAGCGGGGGTCGCTG
CGCCGCCGCCGGCATCTGCTGCAGCCCGGACGGCTGCCGCGCCGACCCCGCCTGCGAC
CCCGACGCCGCCTTCTCCCAGCGC

3. Alignment of the mammalian species used in the *AVPR1a* analyses.

>Homo_sapiens

ATGCGTCTCTCCGCCGGTCCCGACGCGGGGCCCTCGGGCAACTCCAGCCCATGGTGGCC
TCTGGCCACCGGCGCTGGCAACACAAGCCGGGAGGCCGAAGCCCTCGGGGAGGGCAAC
GGCCCACCGAGGGACGTGCGCAACGAGGAGCTGGCCAAACTGGAGATCGCCGTGCTGG
CGGTGACTTTCGCGGTGGCCGTGCTGGGCAACAGCAGCGTACTGCTGGCTCTGCACCGG
ACGCCGCGCAAGACGTCCCGCATGCACCTCTTCATCCGACACCTCAGCCTGGCCGACCTG
GCCGTGGCATTCTTCCAGGTGCTGCCGCAAATGTGCTGGGACATCACCTACCGCTTCCGC
GGCCCCGACTGGCTGTGCCGCGTGGTGAAGCACCTGCAGGTGTTCGGCATGTTTGCGTC
GGCCTACATGCTGGTAGTCATGACAGCCGACCGCTACATCGCGGTGTGCCACCCGCTCAA
GACTCTGCAACAGCCCGCGCGCCGCTCGCGCCTCATGATCGCGGCCGCCTGGGTGCTGA
GCTTCGTGCTGAGCACGCCGCAGTACTTCGTCTTCTCCATGATCGAGGTGAACAATGTCAC
CAAGGCCCGCGACTGCTGGGCCACCTTCATCCAGCCCTGGGGTTCTCGTGCCTACGTGAC
CTGGATGACGGGCGGCATCTTTGTGGCGCCCGTGGTCATCTTGGGTACCTGCTACGGCTT
CATCTGCTACAACATCTGGTGCAACGTCCGCGGGAAGACGGCGTCGCGCCAGAGCAAGG
GTGCAGAGCAAGCGGGTGTGGCCTTCCAAAAGGGGTTCCTGCTCGCACCCTGTGTCAGCA
GCGTGAAGTCCATTTCCCGGGCCAAGATCCGCACGGTGAAGATGACTTTTGTGATCGTGA
CGGCTTACATCGTCTGCTGGGCGCCTTTCTTCATCATCCAGATGTGGTCTGTCTGGGATCC
CATGTCCGTCTGGACCGAATCGGAAAACCCTACCATCACCATCACTGCATTACTGGGTTCC
TTGAATAGCTGCTGTAATCCCTGGATATACATGTTTTTTAGTGGCCATCTCCTTCAAGACTG
TGTTCAAAGCTTCCCATGCTGCCAAAACATGAAGGAAAAATTCAACAAAGAAGATACTGACA
GTATGAGCAGAAGACAGACTTTTTATTCTAACAATCGAAGCCCAACAAACAGTACGGGTAT
GTGGAAGGACTCGCCTAAATCTTCCAAGTCCATCAAATTCATTCCTGTTTCAACT

>Pan_troglodytes

ATGCGTCTCTCCGCCGGTCCCGACGCGGGGCCCTCGGGCAACTCCAGCCCATGGTGGCC
CCTGGCCACCGGCGCTGGCAACACAAGCCGGGAGGCCGAAGCCCTCGGGGAAGGCAAC
GGCTCACCGAGGGACGTGCGCAACGAGGAGTTGGCCAAACTGGAGATCGCCGTGCTGGC
GGTGACTTTCGCGGTGGCCGTGCTGGGCAACAGCAGCGTACTGCTGGTTCTGCACCGGA
CGCCGCGCAAGACGTCCCGCATGCATCTCTTCATCCGACACCTCAGCCTGGCCGACCTGG
CCGTGGCATTCTTCCAGGTGCTGCCGCAAATGTGCTGGGACATCACCTACCGCTTCCGCG
GCCCCGACTGGCTGTGCCGCGTGGTGAAGCACCTGCAGGTGTTCGGCATGTTCGCGTCG
GCCTACATGCTGGTAGTCATGACAGCCGACCGCTACATCGCCGTGTGCCACCCGCTCAAG
ACTCTGCAACAGCCCGCGCGCCGCTCGCGCCTCATGATCGCGGCCGCCTGGGTGCTGAG
CTTCGTGCTGAGCACGCCGCAGTACTTCGTCTTCTCCATGATCGAGGTGAACAATGTCACC
AAGGCCCGCGACTGCTGGGCCACCTTCATCCAGCCCTGGGGTTCTCGTGCCTACGTGACC
TGGATGACGGGCGGCATCTTTGTGGCGCCCGTGGTCATCTTGGGTACCTGCTACGGCTTC
ATCTGCTACAACATCTGGCGCAACGTCCGCGGGAAGACGGCGTCGCGCCAGAGCAAGGG
TGCAGAGCAAGCGGGTGTGGCCTTCCAAAAGGGGTTCCTGCTCGCACCCTGTGTCAGCAG
CGTGAAGTCCATTTCCCGGGCCAAGATCCGCACGGTGAAGATGACTTTTGTGATCGTGAC
GGCTTACATCGTCTGCTGGGCGCCTTTCTTCATCATCCAGATGTGGTCTGTCTGGGATCCC
AAGTCCGTCTGGACCGAATCGGAAAACCCTACCATCACCATCACTGCATTACTGGGTTCCT
TGAATAGCTGCTGTAATCCCTGGATATACATGTTTTTTAGTGGCCATCTCCTTCAGGACTGT
GTTCAAAGCTTCCCATGCTGCCAAAACATGAAGGAAAAATTCAACAAAGAAGATACTGACA
GTATGAGCAGAAGACAGACTTTTTATTCTAACAATCGAAGCCCAACAAACAGCACGGGTAT
GTGGAAGGACTCACCTAAATCTTCCAAGTCCATCAAATTCATTCCTGTTTCAACT

>Mus_musculus

ATGAGTTTCCCGCGAGGCTCCCACGATCTGCCCGCGGGCAACTCCAGCCCGTGGTGGCC
TCTGACCACCGAGGGCGCCAACAGCAGCCGGGAAGCAGCTGGGCTCGGGGAAGGCGGC
AGCCCGCCGGGGGACGTACGCAACGAGGAGCTGGCGAAGCTGGAGGTTACCGTGCTGG
CGGTGATTTTCGTGGTGGCCGTGCTGGGTAATAGCAGTGTGCTGCTGGCGCTGCATCGCA
CGCCACGCAAGACATCCCGCATGCATCTCTTCATCCGACACCTCAGCCTGGCAGACCTGG
CGGTCGCCTTCTTCCAAGTGTTACCACAGCTGTGCTGGGACATCACCTACCGCTTCCGCG
GGCCGGACTGGCTGTGCCGGGTGGTGAAGCACTTGCAGGTGTTTGCCATGTTCGCATCTT
CCTACATGCTGGTGGTGATGACGGCTGACCGCTACATCGCCGTGTGCCACCCGCTCAAGA
CCCTGCAGCAGCCCGCGCGCCGCTCGCGCCTCATGATCGCCGCCTCTTGGGGGCTGAGT
TTCGTTCTGAGCATACCACAGTACTTTATCTTCTCTGTGTTTGAGGTGAACAATGGCACCAA
AGCCCAAGATTGCTGGGCTACCTTCATCCCGCCCTGGGGTACCCGTGCCTACGTGACCTG
GATGACCAGCGGGGTCTTCGTGGTACCCGTGATCATCTTGGGTACCTGCTATGGCTTCAT
CTGTTACCACATCTGGCGCAATGTCCGAGGGAAGACAGCATCGCGACAGGGCAAGGGCT
CTGGGGAAGCGGG---
TCCCTTCCACAAGGGGCTTCTGGTCACGCCTTGTGTCAGCAGCGTGAAGAGCATTTCCCG
TGCCAAGATCCGCACAGTGAAGATGACCTTTGTGATTGTAAGCGCCTACATCCTCTGCTGG
ACACCTTTCTTCATCGTCCAGATGTGGTCAGTCTGGGATACCAATTTCGTTTGGACCGATTC
CGAAAACCCTTCCACCACGATCACGGCGTTACTGGCTTCCTTGAACAGCTGCTGCAACCC
GTGGATCTACATGTTTTTTAGTGGTCATCTCCTACAAGATTGTGTCCAGAGCTTCCCATGCT
GCCAAAGCATAGCGCAGAAATTCGCCAAGGATGACTCGGATAGCATGAGCCGGAGACAGA
CATCTTATTCTAACAACCGAAGCCCAACAAACAGCACTGGGACGTGGAAGGATTCACCTAA
ATCTTCAAAGTCCATCAGATTTATCCCTGTCTCCACT

>Rattus_norvegicus

ATGAGTTTCCCGCGAGGCTCCCAGGATCGGTCCGTGGGCAACTCCAGCCCGTGGTGGCC
TCTAACCACCGAGGGCTCCAACGGCAGTCAGGAGGCAGCCAGGCTTGGGGAAGGTGACA
GCCCGCTGGGGGACGTACGCAATGAGGAGCTGGCCAAACTGGAAATCGCTGTGCTGGCA
GTGATTTTTGTGGTGGCTGTGCTGGGCAATAGCAGTGTGCTGCTGGCGCTGCATCGCACG
CCACGCAAGACATCCCGCATGCACCTCTTTATCCGACACCTCAGCCTGGCAGACCTGGCG
GTCGCCTTCTTCCAAGTATTACCGCAGCTATGCTGGGACATCACCTACCGCTTCCGCGGG
CCGGACTGGCTGTGCCGCGTGGTGAAGCACCTGCAGGTGTTTGCCATGTTCGCGTCTGCC
TATATGCTGGTGGTGATGACAGCCGACCGCTACATCGCCGTGTGCCACCCGCTCAAGACC
CTGCAGCAGCCGGCGCGCCGCTCGCGCCTCATGATCGCCACCTCTTGGGTGCTGAGTTT
CATACTAAGCACGCCACAGTACTTTATATTCTCTGTGATCGAGGTGAACAATGGCACTAAAA
CCCAAGACTGCTGGGCTACCTTCATCCAGCCCTGGGGTACCCGCGCCTACGTGACCTGGA

TGACCAGCGGTGTCTTCGTGGCACCTGTGGTCGTCTTGGGTACATGCTATGGCTTCATCTG
CTACCACATCTGGCGCAACATCCGCGGAAAGACAGCGTCGCGACACGACAAGGGCTCTG
GGGAGGCCGTGGGTCCCTTTCATAAGGGGCTTTTGGTTACACCTTGTGTCAGCAGCGTGA
AGAGCATTTCCCGCGCCAAGATCCGCACTGTGAAGATGACCTTTGTGATTGTAAGCGCCTA
CATCCTTTGCTGGGCGCCTTTCTTCATTGTCCAGATGTGGTCAGTCTGGGATGAGAATTTC
ATCTGGACCGATTCAGAAAACCCTTCCATCACAATCACGGCGTTGCTGGCTTCCTTGAACA
GCTGCTGCAACCCGTGGATATACATGTTTTTCAGTGGCCATCTCCTGCAAGACTGCGTCCA
AAGTTTCCCATGCTGCCACAGCATGGCGCAGAAATTCGCCAAGGATGACTCCGATAGCAT
GAGCCGAAGACAGACTTCTTATTCTAACAACCGGAGCCCAACGAACAGCACTGGGATGTG
GAAGGACTCGCCCAAATCTTCCAAATCCATCAGATTCATTCCTGTCTCCACT

>Peromyscus_maniculatus

ATGAGTTTCCCGCGAGGTTCCCACGATCGGGCGGCCAACAACTCCAGCCGGTGGTGGCC
TCTGAGCGCCGAGGATGCCAACAGCAGCCGGGAGGCGGGGCTGCTCCAGGAAGGTAAC
GACCCTCCCGGGGATGTGCGCAACGAGGAGCTGGCCAAGCTGGAGATCGCCGTGCTGGC
GGTGATTTTCGTGGTGGCCGTGCTGGGCAATAGCAGTGTGCTGCTGGCGTTGCATCGCAC
GCCACGCAAGACATCCCGCATGCACCTCTTTATCCGACACCTCAGCCTGGCAGACTTGGC
GGTCGCCTTCTTCCAAGTGTTGCCACAGCTGTGCTGGGACATCACCTACCGCTTCCGCGG
GCCGGACTGGCTGTGCCGCGTGGTGAAGCACCTGCAGGTGTTTGCCATGTTCGCGTCCG
CCTACATGCTGGTGGTCATGACCGCCGACCGCTACATCGCGGTGTGCCACCCGCTCAAGA
CCCTGCAGCAGCCCGCGCGCCGCTCGCGCCTCATGATCGCTGCCTCCTGGGTGCTGAGT
TTCCTACTAAGCACGCCGCAGTACTTCATCTTCTCTATGATCGAGGTGAACAACGGTACCA
AAACCCAAGACTGCTGGGCTACCTTCATCCAGCCCTGGGGTACCCGTGCCTATGTGACCT
GGATGACCAGTGGTGTCTTTGTGGTACCTGTGGTCATCTTGGGTACCTGCTACGGCTTCAT
CTGCTACCACATCTGGCGCAACGTCCGTGGGAAGACGGCGTCGCGGCAGAGCAAGGACT
CTGGGGACACCCCAGGTCCCTTCCACAAGGGGCTTCTGGTCACGCCTTGTGTCAGCAGCG
TGAAGACCATTTCCCGTGCCAAGATCCGCACAGTGAAGATGACCTTTGTGATCGTGACTGC
CTACATCCTCTGCTGGGCGCCTTTCTTCATCGTCCAGATGTGGTCAGTCTGGGATGACAAC
TTCATCTGGACCGATTCAGAAAACCCCTCCATCACCATCACGGCGTTACTAGCATCCTTGA
ACAGCTGCTGCAACCCCTGGATATACATGTTTTTAGCGGCCATCTCCTGCAAGACTGTGT
CCAAAGCTTTCCATGCTGCCAAAGCGTGGTGCAGAAATTCACCAAGGACGACTCGGACAG
CATGAGCAGGAGGCAGACTTCTTACTCCAACAACCGAAGCCCCACAAACAGCACAGGGAT
GTGGAAGGACTCACCTAAATCGTCCAAGTCCATCAAATTCATCCCTGTTGCCACC

>Ictiodomys_tridecemlineatus

ATGCGTTTCTCCAGAGGCGCCGACGCGGGACCGCTGGGCAACTCCAGTTCGTGGTGGCC
TCTGGCTGCCGATAGTGCCAACCAGAGCCAGGAAGCAGATGCGCTTGGGGAAGGTGGTG
GTCCCCCGGGAGACCGGCGCAATGAGGAACTGGCCAAGCTGGAGATCGCGGTGCTGGC
GGTGACTTTCGTGGTGGCCGTGCTAGGCAACAGCAGCGTGCTTCTGGCGCTACACCGCAC
ACCGCGCAAGACGTCCCGCATGCACCTCTTCATCCGACACCTCAGCCTGGCCGACCTCGC
TGTCGCCTTCTTCCAGGTGTTACCGCAGCTGTGCTGGGACATAACCTACCGTTTCCGCGG
ACCCGACTGGCTGTGTCGCGTGGTGAAGCACCTGCAGGTGTTCGCCATGTTCGCGTCGG
CTTACATGCTGGTAGTAATGACCGCAGATCGCTACATTGCTGTGTGCCACCCGCTCAAGAC
TCTGCAGCAGCCGGCGCGCCGCTCGCGCTTCATGATCGCCGCCTCTTGGGTACTGAGCTT
CGTGCTGAGCACCCCACAGTACTTCATCTTCTCCATGATCGAGGTGAACAATGTTACCAAA
GCTCAAGACTGCTGGGCCACTTTCATCCAACCCTGGGGCACCCGAGCCTATGTGACCTGG
ATGACAAGCGGCATCTTCGTGGCACCCGTGGTCATCTTAGGTACCTGCTACGGCTTCATCT
GCTACCACATCTGGCGCAACATCCGCGGAAAGACCGCGTCGCGCCAGAGCAAGGGTGCA
AGCGGCGCGGAGGACGCCCTCCATAAGGGGATTCTGGTAGCGCCCTGTGTCAGCAGTGT
GAAGACCATTTCCCGCGCCAAGATTCGCACCGTAAAGATGACTTTTGTGATTGTGACGGCT
TACATCCTCTGCTGGGCTCCTTTCTTCATCATCCAGATGTGGTCGGTCTGGGATGAAAACT
TCATCTGGACCGATTCAGAAAACCCTTCCACCACCATCACGGCTTTACTGGCTTCTTTGAAT
AGTTGCTGCAATCCCTGGATATATATGTTTTTTAGTGGCCATCTCCTGCAAGACTGTGTCCA
GAGCTTTCCATGCTGCCACCACATGAAGCAAAAATTCAACAAAG---
ATACCGACAGTATGAGCAGAAGACAGACTTCTTATTCTAACAACCGAAGCCCAACAAATAG
TACCGGTACATGGAAGGACTCACCTAAATCTTCCAAGTCCATCAAATTTATTCCTGTTTCAA
CC

>Octodon_degus

ATGCGCTTCTCCCGCAACGCGGACGCGGGTCCGGCGGGCAACTCCAGCTCCTGGTGGGC
TCCGACCGCAGACGGTGCCAACAGCAGCGGCGAGGCAGATGTGCCGGGCGAAGGCGAC
TGCCAGCCCCGGGACGTGCGCAACGAGGAGCTGGCCAAGGTAGAGATCGCTGTGCTGGC
TGTGACTTTCGTGGTGGCGGTGCTGGGCAACAGCAGCGTGCTGCTAGCTCTGCACCGGA
CGCCTCGCAAGACGTCCCGCATGCACCTCTTCATCCGGCACCTCAGCCTGGCCGACTTGG
CGGTCGCCTTCTTCCAGGTGTTACCGCAGCTGTGCTGGGACATCACCTACCGCTTCCGCG
GCCCCGACTGGCTGTGCCGCGTGGTGAAGCACCTGCAGGTGTTCGCCATGTTCGCGTCG
GCCTACATGCTGGTCGTCATGACCGCGGACCGCTACATCGCCGTGTGCCACCCGCTCAAG
ACTCTGCAGCAGCCCGCGCGCCGCTCGCGCCTCATGATCGCCGCCTCTTGGGCGCTGAG
CTTCGCGCTGAGCACCCCGCAGTACTTCATCTTCTCCATGATCGAGGTGGACAACGTCAC
CAAAGCCCAAGACTGCTGGGCCACCTTCATCCAGCCCTGGGGGAGCCGCGCCTACGTCA
CCTGGATGACGAGCTGCGTCTTCGTGGCACCCGTGGTCATCTTAGGTACCTGCTACGGCT
TCATCTGCTATCACATCTGGCGCAATGTCCGCGGAAAAACCGCGTCGCGCCAGGGCAAGA
GCGCAGGGGGCTCAGGGAGCGCCTTCCACAAGGGGCTCTCGGACGCGCCCTTCGTCAGC
AGCGTGAAGACTATTTCCCGCGCCAAGATCCGCACGGTGAAAATGACTTTTGTCATTGTGA
CGGTTTACATCCTCTGTTGGGCGCCTTTCATCATCGTCCAGATGTGGTCCGTCTGGGATGA
GAAGTTCCTCTGGACCGATTCAGAAAACCCTTCTACCACCATCACTGCACTACTGGCTTCC
TTGAACAGCTGCTGCAACCCCTGGATATACATGTTTTTCAGTGGCCATCTCCTGCAAGACT
GTGTCAAAAGCTTCCCATGCTGCCAAACCATGAAGCAAAAATTCCACAAAGAAGATACTGA
CAGTATGAGCAGAAGACACACTTCTTACTCCAACAACCGAAGCCCAACAAACAGCACAGGT
ACATGGAAGGACTCACCTAAATCTTCCAAATCCATCAGATTCATTCCTGTTTCTACC

>Cavia_porcellus

ATGCGCTTCTCCGGAAACGCGGTCGCGGATCCAGCGGGCAACTCCAGCGCCTGGTGGGC
TCTGACTGCAGATGGTGCCAACAGTAGCCGCGAAGCGGATGTGCCCAGAGAAGGCGACT
GCCAGCCGCGGGACGTGCGCAATGAGGAGCTGGCCAAGGTGGAGATCGCTGTGCTGGCT
GTGACTTTCGTGGTAGCGGTGCTGGGCAATAGCAGCGTGCTGCTAGCTCTGCACCGCACG
CCACGCAAGACGTCCCGCATGCACCTCTTCATCCGGCACCTCAGCCTGGCCGACTTGGCT
GTCGCCTTCTTCCAGGTGTTGCCGCAGCTGTGCTGGGACGTCACCTACCGCTTCCGCGGG
CCCGACTGGCTGTGCCGCGTGGTGAAGCACCTGCAGGTGTTCGCCATGTTCGCGTCGGC
CTACATGCTGGTAGTCATGACCGCCGACCGCTACATCGCCGTGTGCCACCCGCTCAAGAC
TCTGCAGCAGCCCGCGCGCCGCTCGCGCCTCATGATCGCCGCCTCTTGGGTGCTGAGCT
TCGTGCTGAGCACGCCGCAGTACTTCATCTTCTCCATGATCGAGGTGGACAATGTCACCAA
GGCGCAGGACTGCTGGGCCACCTTCATCCAGCCCTGGGGCAGCCGCGCCTACGTCACCT
GGATGACCAGCTGCGTCTTCGTTGCACCCGTGGTCATCTTGGGTACCTGCTACGGCTTCA
TCTGCTACCACATCTGGCGCAACGTCCGCGGAAAGACCGCGTCGCGCCTGGGCGAAGGT
GCAGCGGGCTCAGGGGGCGCCTTCCATAAGGGCCTCTTGATTGCGCCCTTCGTCAGCAG
CGTGAAGACCATTTCCCGCGCCAAGATCCGCACCGTGAAAATGACTTTCGTGATTGTGACG
GTTTATATCCTCTGCTGGGCGCCTTTCATCATCGTCCAGATGTGGTCGGTCTGGGATGAGA
AGTTCGTCTGGACAGATTCAGAAAACCCTACTACCACCATCACTGCATTACTGGCTTCCTT
GAACAGTTGCTGCAACCCCTGGATATACATGTTTTTTAGTGGCCATCTCCTGCAAGACTGT
GTCAAAAGCTTCCCATGCTGCCAGAGTATGAAGCAAAAAATCAACAAAGAAGATACTGACA
GTATGAGCCGAAGACAGACTTCTTATTTCAACAATCGAAGCCCAACAAACAGCACAGGCAC
ATGGAAGGATTCACCTAAATCTTCCAAGTCCAGCAAATTCATTCCTGTTTCTACC

>Ochotona_princeps

ATGCGGTTTTCTGGAGGCTCCCACTTGGAACCCGCAGGTAACTTCAGCCCCTGGTGGCCC
TTGGGCGCCAGCGGTGCTAACAGCAGCCACGAGGCCGGAGAGCCCGGGAGAGGAGGCG
AGCCACAGGCGGACTTGCGCAACGAGGAGCTGGCCAAGCTGGAGATCGCGGTGCTGGCC
GTGACTTTCGTGGTGGCCGTGCTGGGCAACACCAGTGTGCTGCTGGCACTGCACCGCAC
GCCACGCAAGACGTCCCGCATGCACCTTTTCATTCGGCACCTAAGCCTTGCCGACCTGGC
CGTCGCCTTCTTCCAGGTGCTGCCGCAGTTGTGCTGGGACATCACCTACCGCTTCCGTGG
CCCGGACTGGCTGTGCCGCGTAGTGAAGCACCTGCAGGTGTTCGGGATGTTCGCGTCAG
CCTACATGCTGGTGGTCATGACCGCCGACCGCTACATCGCGGTGTGCCACCCGCTCAAGA
CGCTGCAGCAGCCGGCGCGCCGCTCGCGCCTCATGATCGCCGCCTCCTGGGTGCTGAGC
TTGGTGCTCAGCACACCCCAGTACTTCATCTTCTCCATGATCGAGGTGAACAATGTCACCA
AGGCCCAGGACTGCTGGGCCACCTTCATCCAGCCCTGGGGCTCCCGCGCCTACGTGACC
TGGATGACTAGCGGCATCTTCGTGGCACCCGTGGTCATCTTAGGTACCTGCTACGGCTTC
ATCTGTTACCACATTTGGAGCAACGTCCGCGGAAAGACCGCGTCGCGCCAGAGCAAGGGT
TCCGAGCGCGCGGCCGGCGCCTTCCACAAAGGGCTCCTGCTCACTCCCTGTGTCAGCAG

CGTGAAGAGCATCTCGCGTGCCAAGATCCGCACGGTGAAGATGACATTTGTGATCGTGAC
GGCGTACATCGTTTGTTGGGCGCCTTTCTTCATCATCCAAATGTGGTCAGTGTGGGATGAG
AATTTCAACTGGACCGATTCAGAAAACCCTTCCATCACCATCACTGCCCTACTGGCTTCTTT
GAATAGTTGCTGCAACCCTTGGATATACATGTTTTTTAGTGGCCATCTTCTGCAAGACTGTG
TCCAAAGCTTCCCGTGCTGCCAAAATATAAAGCAAAACTTCAACAAAGAAGACACTGACAG
CATGAGCAGAAGACATACTTCTTACTCTAATAACCGAAGCCCTACTAATAGTACAGGTACAT
GGAAGGACTCACCCAAGTCTTCCAAGTCCATCAAGTTCATTCCTGCTTCCACC

>Oryctolagus_cuniculus

ATGCGTTTCTCGGGAAGCTCCGACGTGGAGCCCGCGGGCAACTTCAGTCCCTGGTGGCC
CCTGAGAGCGGGCGGCGCCAACGGCAGCCTGGGGGGCGAGCCGCCGGGGGAAGGGGA
CGAGCCGCAGGGGGATGTGCGCAACGAAGAGCTGGCCAAGCTGGAGATCGCCGTGCTG
GCCGTGACTTTCGCGGTGGCGGTGCTGGGCAACATCAGCGTGCTGCTGGCGCTGCACCG
CACTCCGCGCAAGACGTCCCGCATGCACCTCTTCATTCGGCACCTCAGCCTGGCCGACCT
GGCCGTCGCCTTCTTTCAGGTGCTGCCGCAGCTGTGCTGGGACATCACCTACCGCTTCCG
CGGCCCCGACTGGCTGTGCCGCGTGGTGAAGCACCTGCAGGTGTTCGCGATGTTCGCGT
CTGCCTACATGCTGGTGGTCATGACCGCCGACCGCTACATCGCCGTGTGCCACCCGCTCA
AGACCCTGCAGCAGCCGGCGCGCCGCTCGCGCCTCATGATCGCCGCCTCCTGGGTGCTG
AGCTTCGTGCTGAGCACGCCGCAGTACTTCATCTTCTCCATGATCGAGGTGAACAATGTCA
CCAAGGCCCAGGACTGCTGGGCCACCTTCATCCAGCCCTGGGGGTCCCGCGCCTACGTG
ACCTGGATGACCAGCGGCATCTTCGTGGCACCCGTGGTCATCTTGGGTACCTGCTACGGC
TTCATCTGCTACCACATCTGGAGCAACGTCCGCGGGAAGACCGCGTCGCGCCAGAGCAAG
GGCTCCGAGGGCGCGGCCGGCGCCTTCCACAAGGGGCTGCTGCTCGCGCCCTGTGTCA
GCAGCGTGAAGACCATTTCCCGCGCCAAGATCCGCACGGTGAAGATGACCTTTGTCATCG
TGACGGCGTACATCGTCTGCTGGGCGCCTTTCTTTATCATCCAGATGTGGTCGGTGTGGG
ATGAGAATTTCAACTGGACCGATTCAGAAAACCCTTCCATCACCATCACTGCTCTGCTGGC
TTCACTGAACAGCTGCTGCAATCCCTGGATATACATGTTTTTCAGTGGCCATCTCCTGCAG
GACTGTCTCCAAAGCTTCCTGTGCTGCCAAAATATAAAGCAAAACTTCAACAAAGAAGAAAC
TGACAGCATGAGCAGAAGACAAACTTCTTACTCTAACAACCGAAGCCCTACCAACAGTACG
GGTACGTGGAAGGACTCACCGAAGTCGTCCAAGTCCATCAAAGTCCTTCCTGCTTCCACC

>Capra_hircus

ATGCGATTCTCCGGGAGCCCCAGCGCGGGGCCCGCGAGTAACTCCAGCCGGTGGTGGCC
TCTGGATGCGGGAGGTGCCAACACCAGCGGGGACTCGGAGGCGCTCGGGGAAGACGGC
GGCCCGCAGGCGGACACGCGCAACGAGGAGCTGGCCAAGCTGGAGATTGCTGTGCTGG
CCGTGATTTTCGTGGTGGCCGTGCTGGGTAACAGCAGTGTGCTGCTGGCGCTGCACCGCA
CGCCTCGCAAGACGTCCCGCATGCACCTCTTCATCCGCCACCTCAGCCTGGCCGACCTGG
CCGTCGCCTTCTTCCAGGTGCTGCCCCAGCTGGGCTGGGACATCACCTACCGTTTCCGCG
GACCCGACGGGCTGTGCCGCGTGGTGAAGCACATGCAGGTGTTCGCCATGTTCGCCTCG
GCCTACATGCTGGTGGTCATGACGGCCGATCGCTACATCGCCGTGTGCCACCCGCTGAAG
ACTCTGCAGCAGCCCGCGCGCCGCTCTCGCCTCATGATCGCCGCCGCCTGGGTGCTGAG
TTTCGTGCTGAGCACCCCGCAGTACTTCGTCTTCTCCGTGGTCGAGGTGAGCAACGTCAC
CAAGACCTACGACTGCTGGGCCAACTTCATCCAGCCCTGGGGTCTCCCGGCCTACGTGAC
CTGGATGACCGGCAGCGTGTTCGTGGCGCCCGTGGTCATCCTGGGCACCTGCTACGGTTT
CATCTGCTACCACATCTGGCGCAACGTCCGCGGAAAGACAGCGGGGCGCCAGGGCGCGG
GCGCCGAGGGCGCGGGAGACGCCTTGTACCGAGGAGTCCTGCACGCACGGTGTGTGAG
CAGCGTGAAGACCATTTCCCGCGCCAAGATCCGCACCGTGAAAATGACCTTCGTGATCGT
GACGGCCTACATCGTTTGCTGGGCGCCCTTCTTCATCATCCAGATGTGGTCTGCCTGGGA
TAAGAATTTCTCCTGGGTCGAGTCAGAAAACCCAGCCACCGCCATCCCTGCATTACTGGCT
TCCTTGAATAGTTGCTGCAACCCCTGGATATACATGTTTTTTAGTGGCCATCTCTTGCAAGA
CTGTGCCCAAAGTTTCCCATGCTGTCAAAATGTGAAACGAACGTTCACCAGAGAAGATTCT
AACAGTATGAGCCGAAGACAGACTTCCTTCACTAACAACCGAAGCCCCACCAACAGTATGG
GAACATGGAAAGACTCGCCTAAATCTTCCAAGTCCATCAGATTCATTCCTGTTTCAACC

>Capra_aegagrus

ATGCGATTCTCCGGGAGCCCCAGCGCGGAGGCCGCGAGTAACTCCAGGCGGTGGGGGC
CTCTGCATGCGGGAGGTGCCAACACCAGCGGGGACTCGGAGGCGCTCGGGGAAGACGG
CGGCCCGCAGGCGGACACGCGCAACGAGGAGCTGGCCAAGCTGGAGATTGCTGTGCTG
GCCGTGATTTTCGTGGTGGCCGTGCTGGGTAACAGCAGTGTGCTGCTGGCGCTGCACCG

CACGCCTCGCAAGACGTCCCGCATGCACCTCTTCATCCGCCACCTCAGCCTGGCCGACCT
GGCCGTCGCCTTCTTCCAGGTGCTGCCCCAGCTGGGCTGGGACATCACCTACCGTTTCCG
CGGACCCGACGGGCTGTGCCGCGTGGTGAAGCACATGCAGGTGTTCGCCATGTTCGCCT
CGGCCTACATGCTGGTGGTCATGACGGCCGATCGCTACATCGCCGTGTGCCACCCGCTGA
AGACTCTGCAGCAGCCCGCGCGCCGCTCTCGCCTCATGATCGCCGCCGCCTGGGTGCTG
AGTTTCGTGCTGAGCACCCCGCAGTACTTCGTCTTCTCCGTGGTCGAGGTGAGCAACGTC
ACCAAGACCTACGACTGCTGGGCCAACTTCATCCAGCCCTGGGGTCTCCCGGCCTACGTG
ACCTGGATGACCGGCAGCGTGTTCGTGGCGCCCGTGGTCATCCTGGGCACCTGCTACGG
TTTCATCTGCTACCACATCTGGCGCAACGTCCGCGGAAAGACAGCGGGGCGCCAGGGCG
CGGGGGCCGAGGGCGCGGGCGACGCCTTGTACCGAGGAGTCCTGCACGCACGGTGTGT
GAGCAGCGTGAAGACCATTTCCCGCGCCAAGATCCGCACCGTGAAAATGACCTTCGTGAT
CGTGACGGCCTACATCGTTTGCTGGGCGCCCTTCTTCATTAGT---
ATGCTCTCTCCTTGGGATAAGAATGTTTTCCATACAGAGTCAGAAAACCCAGCCACCGCCA
TCCCTGCATTACTGGCTTCCTTGAATAGTTGCTGCAACCCCTGGATATACATGTTTTTAGT
GGCCATCTCTTGCAAGACTGTGCCCAAAGTTTCCCATGCTGCCAAAACGTGAAACGAACGT
TCACCAAAGAAGATTCTAACAGTATGAGCCGAAGACAGACTTCCTTCACTAACAACCGAAG
CCCCACCAACAGTATGGGAACATGGAAAGACTCGCCTAAATCTTCCAAGTCCATCAAATTC
ATTCCTGTTTCAACC

>Ovis_aries

ATGCGATTCTCCGGGAGCCCCAGCGCGGGGCCCGCGAGTAACTCCAGCCGGTGGTGGCC
TCTGGACGCGGGAGGTGCCAACACCAGCGGGGACTCGCAGGCGCTCGGGGAAGACGGC
GGCCCGCAGGCGGACACGCGCAACGAGGAGCTGGCAAAGCTGGAGATTGCCGTGCTGG
CCGTGATTTTCGTGGTGGCCGTGCTGGGTAACAGCAGTGTGCTGCTGGCGCTGCACCGCA
CGCCTCGCAAGACGTCCCGCATGCACCTCTTCATCCGCCACCTCAGCCTGGCCGACCTGG
CCGTCGCCTTCTTCCAGGTGCTGCCCCAGCTGGGCTGGGACATCACCTACCGTTTCCGCG
GACCCGACGGGCTGTGCCGCGTGGTGAAGCACATGCAGGTGTTCGCCATGTTCGCCTCG
GCCTACATGCTGGTGGTCATGACGGCCGATCGCTACATCGCCGTGTGCCACCCGCTGAAG
ACTCTGCAGCAGCCCGCGCGCCGCTCTCGCCTCATGATCGCCGCCGCCTGGGTGCTGAG
TTTCGTGCTGAGCACCCCGCAGTACTTCGTCTTCTCCATGGTCGAGGTGAGCAACGTCAC
CAAGACCTACGACTGCTGGGCCAACTTCATCCATCCCTGGGGTCTCCCGGCCTACGTGAC
CTGGATGACCGGCAGCGTGTTCGTGGCGCCCGTGGTCATCCTGGGCACCTGCTACGGTTT
CATCTGCTACCACATCTGGCGCAAGGTCCGCGGAAAGACAGCGGGGCGCCAGGGCGCGG
GCGCCGAGGGCGCGGGAGGCGCCTTGTACCGAGGAGTCCTGCACGCGCGGTGTGTGAG
CAGCGTGAAGACCATTTCCCGCGCCAAGATCCGCACCGTGAAAATGACCTTCGTGATCGT
GACGGCCTACATCGTTTGCTGGGCGCCCTTCTTCATCATCCAGATGTGGTCTGCCTGGGA
TAAGAATTTCTCCTGGGTCGAGTCAGAAAACCCAGCCACCGCCATCCCTGCATTACTGGCT
TCCTTGAATAGTTGCTGCAACCCCTGGATATACATGTTTTTAGTGGCCATCTCTTGCAAGA
CTGTGCCCAAAGCTTCCCATGCTGCCAAAACGTGAAACGAACGTTCACCAGAGAAGGTTCT
GACAGTATGAGCCGAAGACAGACTTCCTTCACTAACAACCGAAGCCCCACCAACAGTATG
GGAACATGGAAAGACTCGCCTAAATCTTCCAAGTCCATCAAATTCATTCCTGTTTCAACC

>Ovis_orientalis

ATGCGATTCTCCGGGAGCCCCAGCGCGGGGCCCGCGAGTAACTCCAGCCGGTGGTGGCC
TCTGGACGCGGGAGGTGCCAACACCAGCGGGGACTCGCAGGCGCTCGGGGAAGACGGC
GGCCCGCAGGCGGACACGCGCAACGAGGAGCTGGCAAAGCTGGAGATTGCCGTGCTGG
CCGTGATTTTCGTGGTGGCCGTGCTGGGTAACAGCAGTGTGCTGCTGGCGCTGCACCGCA
CGCCTCGCAAGACGTCCCGCATGCACCTCTTCATCCGCCACCTCAGCCTGGCCGACCTGG
CCGTCGCCTTCTTCCAGGTGCTGCCCCAGCTGGGCTGGGACATCACCTACCGTTTCCGCG
GACCCGACGGGCTGTGCCGCGTGGTGAAGCACATGCAGGTGTTCGCCATGTTCGCCTCG
GCCTACATGCTGGTGGTCATGACGGCCGATCGCTACATCGCCGTGTGCCACCCGCTGAAG
ACTCTGCAGCAGCCCGCGCGCCGCTCTCGCCTCATGATCGCCGCCGCCTGGGTGCTGAG
TTTCGTGCTGAGCACCCCGCAGTACTTCGTCTTCTCCATGGTCGAGGTGAGCAACGTCAC
CAAGACCTACGACTGCTGGGCCAACTTCATCCATCCCTGGGGTCTCCCGGCCTACGTGAC
CTGGATGACCGGCAGCGTGTTCGTGGCGCCCGTGGTCATCCTGGGCACCTGCTACGGTTT
CATCTGCTACCACATCTGGCGCAAGGTCCGCGGAAAGACAGCGGGGCGCCAGGGCGCGG
GCGCCGAGGGCGCGGGAGGCGCCTTGTACCGAGGAGTCCTGCACGCGCGGTGTGTGAG
CAGCGTGAAGACCATTTCCCGCGCCAAGATCCGCACCGTGAAAATGACCTTCGTGATCGT
GACGGCCTACATCGTTTGCTGGGCGCCCTTCTTCATCATCCAGATGTGGTCTGCCTGGGA
TAAGAATTTCTCCTGGGTCGAGTCAGAAAACCCAGCCACCGCCATCCCTGCATTACTGGCT

TCCTTGAATAGTTGCTGCAACCCCTGGATATACATGTTTTTTAGTGGCCATCTCTTGCAAGA
CTGTGCCCAAAGCTTCCCATGCTGCCAAAACGTGAAACGAACGTTCACCAGAGAAGGTTCT
GACAGTATGAGCCGAAGACAGACTTCCTTCACTAACAACCGAAGCCCCACCAACAGTATG
GGAACATGGAAAGACTCGCCTAAATCTTCCAAGTCCATCAAATTCATTCCTGTTTCAACC

>Camelus_ferus

ATGCGTTTCTCCGGGAGCCCCGGCGCGGGGCCCGCTGGCAACTCCAGCCTGTGGTGGCC
TCTGGCCGCCAGCGGTGCCAACGCCAGCCGGGAAACGGAGGCGCTCGGAAAAGACGGC
GATCCGCAGAGGGACGCGCGCAACGAGGAGGTGGCCAAGCTGGAGATCGCTGTGCTAGC
CGTGATTTTCGTGGTGGCCGTGCTGGGTAACAGCAGTGTATTGCTGGCGCTGCACCGCAC
GCCTTCCAAAAAGTCCCGCATGCACCTCTTCATCCGCCACCTCAGCCTAGCAGACCTGGC
CGTCGCTTTCTTCCAGGTGCTGCCTCAGCTGTGCTGGGACATCACCTACCGTTTCCGTGG
ACCCGACGGGCTGTGCCGCGTGGTGAAGCACCTGCAGGTGTTCGCCATGTTCGCCTCGG
CTTACATGCTCGTGGTCATGACCGCCGACCGCTACATCGCTGTGTGCCACCCGCTGAAGA
CGCTGCAGCAGCCCGCGCGCCGCTCGCGCCTCATGATCGCCGCCGCCTGGGTGCTGAG
CTTCGTGCTGAGCACGCCGCAGTACTTCGTCTTCTCCGTGGTCGAGGTGAACAACGTCAC
CAAGGCCTACGACTGCTGGGCCAACTTCATCCAGCCCTGGGGGCTCCCCGCCTACGTGA
CCTGGATGACCGGCGGCATCTTCGTGGCACCCGTGGTCATCCTTGGTACTTGCTACGGCT
TCATCTGCTACCACATCTGGCGCAACGTCCGCGGAAAGACAGCAGCGCGCCAAGAGAAG
GGCGCCGGGGGCGCGGGTGGCACCTTCCACAAGGGGCTCCTGCTCGAGCCGTGTGTCA
GCAGTGTGAAGATCATTTCCCGCGCCAAGATCCGCACGGTGAAGATGACCTTCGTCATCG
TGACGGCTTACATCGTTTGCTGGGCGCCCTTTTTCATCATCCAGATGTGGTCTGTCTGGGA
TAAGAATTTGCTCTGGGTCGAGTCCGAAAACCCAGCCACCACCATCACTGCATTGCTGGCC
TCCTTGAATAGTTGCTGCAATCCCTGGATATACATGTTTTTTAGTGGCCATCTCCTGCAAGA
CTGTGTCCAGAGCTTCCCATGCTGCCAAAACATGAAGCAAACGTTCAACAAAGGAGATTCT
GACAGTATGAGCCGAAGACAGACTTCTTTCACTAACAACAGAAGCCCAACAAACAGTATGG
GCACATGGAAAGACTCGCCTAAATCTTCCAAGTCCATCAAATTCATTCCTGTTTCAACC

>Camelus_bactrianus

ATGCGTTTCTCCGGGAGCCCCGGCGCGGGGCCCGCTGGCCACCCCAGCCTGTGGTGGC
CTCTGGCCGCCAGCGGTGCCAACGCCAGCCGGGAAACGGAGGCGCTCGGAAAAGACGG
CGATCCGCAGAGGGACGCGCGCAACGAGGAGGTGGCCAAGCTGGAGATCGCTGTGCTAG
CCGTGATTTTCGTGGTGGCCGTGCTGGGTAACAGCAGTGTATTGCTGGCGCTGCACCGCA
CGCCTTCCAAAAAGTCCCGCATGCACCTCTTCATCCGCCACCTCAGCCTAGCAGACCTGG
CCGTCGCTTTCTTCCAGGTGCTGCCTCAGCTGTGCTGGGACATCACCTACCGTTTCCGTG
GACCCGACGGGCTGTGCCGCGTGGTGAAGCACCTGCAGGTGTTCGCCATGTTCGCCTCG
GCTTACATGCTCGTGGTCATGACCGCCGACCGCTACATCGCTGTGTGCCACCCGCTGAAG
ACGCTGCAGCAGCCCGCGCGCCGCTCGCGCCTCATGATCGCCGCCGCCTGGGTGCTGAG
CTTCGTGCTGAGCACGCCGCAGTACTTCGTCTTCTCCGTGGTCGAGGTGAACAACGTCAC
CAAGGCCTACGACTGCTGGGCCAACTTCATCCAGCCCTGGGGGCTCCCCGCCTACGTGA
CCTGGATGACCGGCGGCATCTTCGTGGCACCCGTGGTCATCCTTGGTACTTGCTACGGCT
TCATCTGCTACCACATCTGGCGCAACGTCCGCGGAAAGACAGCAGCGCGCCAGGAGAAG
GGCGCCAAGGGCGCGGGTGGCACCTTCCACAAGGGGCTCCTGCTTGAGCCGTGTGTCAG
CAGTGTGAAGATCATTTCCCGCGCCAAGATCCGCACGGTGAAGATGACCTTCGTCATCGT
GACGGCTTACATCGTTTGCTGGGCGCCCTTTTTCATCATCCAGATGTGGTCTGTCTGGGAT
AAGAATTTGCTCTGGGTCGAGTCCGAAAACCCAGCCACCACCATCACTGCATTGCTGGCCT
CCTTGAATAGTTGCTGCAATCCCTGGATATACATGTTTTTTAGTGGCCATCTCCTGCAAGAC
TGTGTCCAGAGCTTCCCATGCTGCCAAAACATGAAGCAAACGTTCAACAAAGGAGATTCTG
ACAGTATGAGCCGAAGACAGACTTCTTTCACTAACAACAGAAGCCCAACAAACAGTATGGG
CACATGGAAAGACTCGCCTAAATCTTCCAAGTCCATCAAATTCATTCCTGTTTCAACC

>Camelus_dromedarius

ATGCGTTTCTCCGGGAGCCCCGGCGCGGGGCCCGCTGGCAACTCCAGCCTGTGGTGGCC
TCTGGCCGCCAGCGATGCCAACGCCAGCCGGGAAACGGAGGCGCTCGGAAAAGACGGC
GATCCGCAGAGGGACGAGCGCAACGAGGAGGTGGCCAAGCTGGAGATCGCTGTGCTAGC
CGTGATTTTCGTGGTGGCCGTGCTGGGTAACAGCAGTGTATTGCTGGCGCTGCACCGCAC
GCCCTCCAAAAAGTCCCGCATGCACCTCTTCATCCGCCACCTCAGCCTAGCAGACCTGGC
CGTCGCTTTCTTCCAGGTGCTGCCTCAGCTGTGCTGGGACATCACCTACCGTTTCCGTGG
ACCCGACGGGCTGTGCCGCGTGGTGAAGCACCTGCAGGTGTTCGCCATGTTCGCCTCGG

CTTACATGCTCGTGGTCATGACCGCCGACCGCTACATCGCTGTGTGCCACCCGCTGAAGA
CGCTGCAGCAGCCCGCGCGCCGCTCGCGCCTCATGATCGCCGCCGCCTGGGTGCTGAG
CTTCGTGCTGAGCACGCCGCAGTACTTCGTCTTCTCCGTGGTCGAGGTGAACAACGTCAC
CAAGGCCTACGACTGCTGGGCCAACTTCATCCAGCCCTGGGGGCTCCCCGCCTACGTGA
CCTGGATGACCGGCGGCATCTTCGTGGCGCCCGTGGTCATCCTTGGTACTTGCTACGGCT
TCATCTGCTACCACATCTGGCGCAACGTCCGCGGAAAGACAGCAGCGCGCCAGGAGAAG
GGCGCCAAGGGCGCGGGTGGCACCTTCCACAAGGGGCTCCTGCTCGAGCCGTGTGTCAG
CAGTGTGAAGATCATTTCCCGCGCCAAGATCCGCACGGTGAAGATGACCTTCGTCATCGT
GACGGCTTACATCGTTTGCTGGGCGCCCTTTTTCATCATCCAGATGTGGTCTGTCTGGGAT
AAGAATTTGCTCTGGGTCGAGTCCGAAAACCCAGCCACCACCATCACTGCATTGCTGGCCT
CCTTGAATAGTTGCTGCAATCCCTGGATATACATGTTTTTTAGTGGCCATCTCCTGCAAGAC
TGTGTCCAGAGCTTCCCATGCTGCCAAAACATGAAGCAAACGTTCAACAAAGGAGATTCTG
ACAGTATGAGCCGAAGACAGACTTCTTTCACTAACAACAGAAGCCCGACAAACAGTATGGG
CACATGGAAAGACTCGCCTAAATCTTCCAAGTCCATCAAATTCATTCCTGTTTCAACC

>Vicugna_pacos

ATGCGTTTCTCCGGGAGCCCCGGCGCGGGGCCCGCTGGCAACTCCAGCCTGTGGTGGCC
TCTGGCCACCAGCGGTGCCAACGCCAGCCGGGAAACGGAGGCGCTCGGAAAAGACGGC
GATCCGCAGCGGGACGAGCGCAACGAGGAGGTGGCCAAGCTGGAGATCGCTGTGCTAGC
CGTGATTTTCGTGGTGGCCGTGCTGGGTAACAGCAGTGTATTGCTGGCGCTGCACCGCAC
GCCTTCCAAAAAGTCCCGCATGCACCTCTTCATCCGCCACCTCAGCCTGGCAGACCTGGC
CGTCGCTTTCTTCCAGGTGCTGCCTCAGCTGTGCTGGGACATCACCTACCGTTTCCGTGG
ACCCGACGGGCTGTGCCGCGTGGTGAAGCACCTGCAGGTGTTCGCCATGTTCGCCTCGG
CTTACATGCTCGTGGTCATGACCGCCGACCGCTACATCGCTGTGTGCCACCCGCTGAAGA
CGCTGCAGCAGCCCGCGCGCCGCTCGCGCCTCATGATCGCCGCCGCCTGGGTGCTGAG
CTTCGTGCTGAGCACGCCGCAGTACTTCGTCTTCTCCGTGGTCGAGGTGAACAACGTCAC
CAAGGCCTACGACTGCTGGGCCAACTTCATCCAGCCCTGGGGGCTCCCCGCCTACGTGA
CCTGGATGACTGGCGGCATCTTCGTGGCGCCCGTGGTCATCCTTGGTACTTGCTACGGCT
TCATCTGCTACCACATCTGGCGCAACGTCCGCGGAAAGACAACAGCGCGCCAGGAGAAG
GGCGCCAAGGGCGCGGATGGCACCTTCCACAAGGGGCTCCTGCTCGAGCCGTGTGTCAG
CAGTGTGAAGATCATTTCCCGCGCCAAGATCCGCACGGTGAAGATGACCTTCGTCATCGT
GACGGCTTACATCGTTTGCTGGGCGCCCTTTTTTATCATCCAGATGTGGTCTGTCTGGGAT
AAGAATTTGCCCTGGGTCGAGTCTGAAAACCCAGCCACCACCATCACTGCATTGCTGGCCT
CCTTGAATAGTTGCTGCAATCCCTGGATATACATGTTTTTTAGTGGCCATCTCCTGCAAGAC
TGTGTCCAGAGCTTCCCATGCTGCCAAAACATGAAGCAAACGTCCAACAAAGAAGATTCTG
ACAGTATGAGCCGAAGACAGACTTCTTTCACTAATAACAGAAGCCCAACAAACAGTATGGG
CACATGGAAAGACTCGCCTAAATCTTCCAAGTCCATCAAATTCATTCCTGTTTCAACC

>Bos_taurus

ATGCGATTCTCCGGGAGCCCCAGCGCGGAGCCCGCGAGCAACTCCAGCCGGTGGTGGCC
TCTGACTGCCGGAGGTGCCAACACCAGCGGGGACTCGGAGGCGCTCGGGGAAGACGGC
GGCCCACAGGCGGACACGCGCAACGAGGAGCTGGCCAAGCTAGAGATTGCCGTGCTGGC
CGTGATTTTCGTGGTGGCCGTGCTAGGTAACAGCAGTGTGCTGCTGGCGCTGCACCGCAC
GCCTCGCAAGACGTCCCGCATGCACCTCTTCATCCGCCACCTCAGCCTGGCCGACCTGGC
CGTCGCCTTCTTCCAGGTGCTGCCCCAGCTGGGCTGGGACATCACCTACCGTTTCCGCGG
ACCCGACGGGCTGTGCCGCGTGGTGAAGCACATGCAGGTGTTCGCCATGTTCGCCTCGG
CCTACATGCTGGTGGTCATGACCGCCGATCGCTACATCGCCGTGTGCCACCCGCTGAAGA
CGCTGCAGCAGCCCGCGCGCCGCTCGCGCCTCATGATCGCCGCCGCCTGGGTGCTGAGT
TTCGTGCTGAGCACCCCGCAGTACTTCGTCTTCTCCGTGGTCGAGGTGAGCAACGTCACC
AAGACCTACGACTGCTGGGCCAACTTCATCCAGCCCTGGGGTCTCCCGGCCTACGTGACC
TGGATGACCGGCAGCGTGTTCTTGGCGCCCATGGTCATCCTGGGCACCTGCTACGGTTTC
ATCTGCCACCACATCTGGGGCAACGTCCGTGGAAAGACAGCAGGGCGCCAGGGCACGGG
CGCCGAGGGCGCGGGCGGCGCCTTGCACCGAGGAGTCCTGCACGCACGGTGTGTTAGC
AGCGTGAAGACCATTTCCCGCGCCAAGATCCGCACCGTGAAAATGACCTTCGTGATCGTG
ACGGCCTACATCGTTTGCTGGGCACCCTTCTTCATCATCCAAATGTGGTCTGCCTGGGATA
AGAATTTCTCCTGGGTCGAGTCAGAAAACCCAGCCACCGCCATCCCTGCATTATTGGCTTC
CTTGAATAGTTGCTGCAACCCCTGGATATACATGTTTTTTAGTGGCCATCTCTTGCAAGACT
GTGCCCAAAGCTTCCCATGCTGCCAAAACGTGAAACGAACATTCACCAGAGAAAATTCTGA
CAGTATGAACCGAAGACCGACTTCCTTCACTAACACCAGAAGCCCCACCAACAGTATGGGA
ACATGGAAGGGCTCGCCTAAATCTTCCAAGTCCATCAAATTCATTCCTGTTTCAACC

>Bos_primigenius

ATGCGATTCCCCGCGAGCACCAGCGCGGAGCGATCTAGCACCTCCAGCCGGTGGTGGCC
TCTGACTGCCGGAGGTGCCAACCGTAGCGGGGACTCGGAGGCGCTCGGGAGCGGCGGC
GGCCCACAGGCGGACACGCGCAACGAGGAGCTGGCCAAGCTAGAGATTGCCGTGCTGGC
CGTGATTTTCGTGGTGGCCGTGCTAGGTAACAGCAGTGTGCTGCTGGCGCTGCACCGCAC
GCCTCGCAAGACGTCCCGCATGCACCTCTTCATCCGCCACCTCAGCCTGGCCGACCTGGC
CGTCGCCTTCTTCCAGGTGCTGCCCCAGCTGCGCGGGGACATCACCTACCGTTTCCGCGG
ACCCGACGGGCTGTGCCGCGTGGTGAAGCACATGCAGGTGTTCGCCATGTTCGCCTCGG
CCTACATGCTGGTGGTCATGACCGCCGATCGCTACATCGCCGTGTGCCACCCGCTGAAGA
CGCTGCAGCAGCCCGCGCGCCGCTCGCGCCTCATGATCGCCGCCGCCTGGGTGCTGAGT
TTCGTGCTGAGCACCCCGCAGTACTTCGTCTTCTCCGTGGTCGAGGTGAGCAACGTCACC
AAGACCTACGACTGCTGGGCCAACTTCATCCAGCCCTGGGGTCTCCCGGCCTACGTGACC
TGGATGACCGGCAGCGTGTTCTTGGCGCCCATGGTCATCCTGGGCACCTGCTACGGTTTC
ATCTGCCACCACATCTGGGGCAACGTCCGTGGAAAGACAGCAGGGCGCCAGGGCACGGG
CGCCGAGGGCGCGGGCGGCGCCTTGCACCGAGGAGTCCTGCACGCACGGTGTGTTAGC
AGCGTGAAGACCATTTCCCGCGCCAAGATCCGCACCGTGAAAATGACCTTCGTGATCGTG
ACGGCCTACATCGTTTGCTGGGCACCCTTCTTCATCATCCAAATGTGGTCTGCCTGGGATA
AGAATTTCTCCTGGGTAGAGTCAGAAAACCCAGCCACCGCCATCCCTGCATTATTGGCTTC
CTTGAATAGTTGCTGCAACCCCTGGATATACATGTTTTTTAGTGGCCATCTCTTGCAAGACT
GTGCCCAAAGCTTCCCATGCTGCCAAAACGTGAAACGAACATTCACCAGAGAAAATTCTGA
CAGTATGAACCGAAGACCGACTTCCTTCACTAACACCAGAAGCCCCACCAACAGTATGGGA
ACATGGAAGGGCTCGCCTAAATCTTCCAAGTCCATCAAATTCATTCCTGTTTCAACC

>Sus_scrofa_familiaris

ATGCGTTTCTCCGGGAACCCCGGGGCCGGGCCCGCGGGCAACTCTAGCCGGTGGTGGTC
TCTGGCCGCCGGAGATGCCAATACCAGTCGGGGAGCCGAGGCGCAGGGGAAAGACGGC
GACCCGCAGGCCGACAAGCGCAACGAAGAGCTGGCCAAACTGGAGATCGCTGTGCTGGC
TGTGATTTTCGTGGTGGCGGTGGTGGGTAACAGCAGTGTGTTGCTGGCGCTGCACCGCAC
GCCTCGCAAGACGTCCCGCATGCACCTCTTTATCCGCCACCTCAGCCTGGCCGACCTGGC
CGTCGCTTTCTTCCAGGTGCTGCCTCAGCTGTGCTGGGACATCACCTATCGTTTCCGCGG
ACCCGACGGGCTGTGCCGCGTGGTGAAGCACCTGCAGGTGTTCGGCATGTTCGCATCGG
CTTATATGCTGGTGGTCATGACCGCCGACCGATACATCGCTGTTTGCCATCCGCTGAAGAC
GCTGCAGCAGCCGGCGCGCCGCGCGCGCCTCATGATCGCCGCCGCCTGGGTGCTGAGC
TTCGTGCTGAGCATTCCGCAGTACTTCGTCTTCTCCATGGTCGAGGTGAGCAATGTCACCA
AGGCCTACGACTGCTGGGCCAACTTCATCCAGCCCTGGGGTCTCCCCGCCTACGTGACCT
GGATGACTGGCGGCATCTTCGTGGTGCCCGTGGTCATCCTGGGCACCTGTTACGGCTTCA
TCTGCTATCACATCTGGCGCAACGTCCGTGGAAAGACAGCGGCGCGCCAGGACATGGGG
GGGGAGAGCGCGGGTGGCGCCTTCCACCGGGGGCTCCTGCTCCAGCCGTGTGTCAGCA
GCGTGAAGACCATTTCTCGCGCCAAGATCCGCACGGTGAAGATGACCTTCGTGATCGTAA
CAGCTTACATCGTTTGCTGGGCGCCCTTCTTCATCATCCAGATGTGGTCTGTCTGGGATGA
GAAGTTCTCCTGGATTGACTCGGAAAACCCAACCACCGCCATCACTGCATTATTGGCTTCC
TTGAATAGCTGTTGCAATCCCTGGATATATATGTTTTTTAGTGGCCATCTCTTGCAAGACTG
TGTTCAAAACTTCCCATGCTGCCAAAACACGAAGCGAACATTCAACAAAGAAGATTCTGAC
AGTATGAGCCGAAGACAAACTTCTTTTACTAACAGCCGAAGCCCAACAAACAGTATGGGCC
CATGGAAGGACTCGCCTAAATCTTCCAAGTCTATTAAGTCCATTCCGATTTCAACC

>Sus_scrofa

ATGCGTTTCTCCGGGAACCCCGGGGCCGGGCCCGCGGGCAACTCTAGCCGGTGGTGGTC
TCTGGCCGCCGGAGATGCCAATACCAGTCGGGAAGCCGAGGCGCAGGGGAAAGACGGC
GACCCGCAGGCCGACAAGCGCAACGAAGAGCTGGCCAAACTGGAGATCGCTGTGCTGGC
TGTGATTTTCGTGGTGGCGGTGGTGGGTAACAGCAGTGTGTTGCTGGCGCTGCACCGCAC
GCCTCGCAAGACGTCCCGCATGCACCTCTTTATCCGCCACCTCAGCCTGGCCGACCTGGC
CGTCGCTTTCTTCCAGGTGCTGCCTCAGCTGTGCTGGGACATCACCTATCGTTTCCGCGG
ACCCGACGGGCTGTGCCGCGTGGTGAAGCACCTGCAGGTGTTCGGCATGTTCGCATCGG
CTTATATGCTGGTGGTCATGACCGCCGACCGATACATCGCTGTTTGCCATCCGCTGAAGAC
GCTGCAGCAGCCGGCGCGCCGCGCGCGCCTCATGATCGCCGCCGCCTGGGTGCTGAGC
TTCGTGCTGAGCATTCCGCAGTACTTCGTCTTCTCCATGGTCGAGGTGAGCAATGTCACCA
AGGCCTACGACTGCTGGGCCAACTTCATCCAGCCCTGGGGTCTCCCCGCCTACGTGACCT
GGATGACTGGCGGCATCTTCGTGGTGCCCGTGGTCATCCTGGGCACCTGTTACGGCTTCA

TCTGCTATCACATCTGGCGCAACGTCCGTGGAAAGACAGCGGCGCGCCAGGGCATGGGG
GGGGAGAGCGCGGGTGGCGCCTTCCACCGGGGGCTCCTGCTCCAGCCGTGTGTCAGCA
GCGTGAAGACCATTTCTCGCGCCAAGATCCGCACGGTGAAGATGACCTTCGTGATCGTAA
CAGCTTACATCGTTTGCTGGGCGCCCTTCTTCATCATCCAGATGTGGTCTGTCTGGGATGA
GAAGTTCTCCTGGACAGACTCGGAAAACCCAACCACCGCCATCACTGCATTATTGGCTTCC
TTGAATAGCTGTTGCAATCCCTGGATATATATGTTTTTTAGTGGCCATCTCCTGCAAGACTG
TGTTCAAAACTTCCCATGCTGCCAAAACACGAAGCGAACATTCAACAAAGAAGATTCTGAC
AGTATGAGCCGAAGACAAACTTCTTTTACTAACAGCCGAAGCCCAACAAACAGTATGGGCC
CATGGAAGGACTCGCCTAAATCTTCCAAGTCTATTAAGTCCATTCCGATTTCAACC

>Equus_caballus

ATGCGTTTCTCCGGAGGCCCTGGCGCCGGGTCCACGGGCAACTCAAGCCGCTGGTGGCC
TCTGGCCGCCGGCGGTGCCAACGCCAGCCGCGAATC-
GAAGCGCTCGGGGAAGAAGGCGGCCCAGCGAGGGACTTGCGCAACGAGGAGCTGGCCA
AGGTGGAGATCGCCGTGCTGGCCGTGACTTTCGTGGTGGCCGTACTGGGTAACAGCTGC
GTGTTGCTGGCACTGCATCGCACGCCTCGCAAGACGTCCCGCATGCACCTCTTCATCCGC
CACCTCAGCCTGGCCGACCTGGCCGTCGCTTTCTTCCAGGTGCTGCCGCAGCTGTGCTGG
GACATCACCTACCGTTTCCGCGGCCCCGACGGGCTGTGCCGCGTGGTGAAGCACCTGCA
GGTGTTCGGCATGTTCGCCTCGGCTTACATGCTGGTGGTCATGACCGCCGACCGCTACAT
CGCCGTGTGCCACCCGCTCAAGACGCTGCAGCAGCCGACGCGCCGCTCGCGCCTCATGA
TCGCGGCCGCCTGGGTGCTGAGCTTCGTGCTGAGCACGCCGCAGTACCTCGTCTTCTCCA
TGGTCGAGGTGAGCAACGTCACCAAGGCCTACGACTGCTGGGCCACCTTCATCCAGCCCT
GGGGACCCCGCGCCTACGTGACTTGGATGACAGCCAGCATCTTCGTGGCGCCCGTGGTC
ATCCTGGGCACCTGCTACGGCTTCATCTGCTACCACATCTGGCGCAACGTCCGCGGGAAG
ACGGCGTCGCGCCAGGACAAGGGCGCCGAGCGCGCGGGCGGCGCCTTCCGCAATGGGC
TCCTGCTCGAGCCCTGTGTCAGCAGCGTGAAGACCATCTCCCGAGCCAAGATCCGCACCG
TGAAGATGACTTTCGTGATTGTGACCGCTTACATCGTTTGCTGGATGCCCTTCTTCATCGTC
CAGATGTGGTCTGTCTGGGATGTGAAGTTCCACTGGATCGAGTCGGAAAACCCTGCCACC
ACCATCACGGCCTTGCTGGCTTCCTTGAATAGTTGCTGCAATCCCTGGATATACATGTTTTT
TAGCGGTCATCTCCTGCAGGACTGTGTCCAAAGCTTCCCATGCTGCCAAAACATGAAGCAA
ACGTTCAGCAAAGAAGATTCAGACAGTACGAGCAGAAGGCAGACTTCTTACACTAACAACC
GAAGCCCGACAAACAGCACAGGTCCGTGGAAGGACTCTCCTAAATCTTCCAAGATCATCAA
ATTCATTCCAGTTTCAACC

>Equus_assinus

ATGCGTTTCTCCGGAGGCCCTGGCGCCGGGTCCACGGGCAACTCAAGCCGCTGGTGGCC
TCTGGCCGCCGGCGGTGCCAACGCCAGCCGCGAATCGGAGGCGCTCGGGGAAGAAGGC
GGCCAGCAGAGGGACTTGCGCAACGAGGAGCTGGCCAAGGTGGAGATCGCCGTGCTGG
CCGTGACTTTCGTGGTGGCCGTACTGGGTAACAGCTGCGTGTTGCTGGCACTGCACCGCA
CGCCTCGCAAGACGTCCCGCATGCACCTCTTCATCCGCCACCTCAGCCTGGCCGACCTGG
CCGTCGCTTTCTTCCAGGTGCTGCCGCAGCTGTGCTGGGACATCACCTACCGTTTCCGCG
GCCCCGACGGGCTGTGCCGCGTGGTGAAGCACCTGCAGGTGTTCGGCATGTTCGCCTCG
GCTTACATGCTGGTGGTCATGACCGCCGACCGCTACATCGCCGTGTGCCACCCGCTCAAG
ACGCTGCAGCAGCCGACGCGCCGCTCGCGCCTCATGATCGCGGCCGCCTGGGTGCTGAG
CTTCGTGCTGAGCACGCCGCAGTACCTCGTCTTCTCCATGGTCGAGGTGAGCAACGTCAC
CAAGGCCTACGACTGCTGGGCCACCTTCATCCAGCCCTGGGGACCCCGCGCCTACGTGA
CTTGGATGACAGCCAGCATCTTCGTGGCGCCCGTGGTCATCCTGGGCACCTGCTACGGCT
TCATCTGCTACCACATCTGGCGCAACGTCCGCGGGAAGACGGCGTCGCGCCAGGACAAG
GGCGCCGAGCGCGCGGGCGGCGCCTTCCGCAATGGGCTCCTGCTCGAGCCCTGTGTCA
GCAGCGTGAAGACCATCTCCCGAGCCAAGATCCGCACCGTGAAGATGACTTTCGTGATTG
TGACCGCTTACATCGTTTGCTGGATGCCCTTCTTCATCGTCCAGATGTGGTCTGTCTGGGA
TGTGAAGTTCCACTGGATCGAGTCGGAAAACCCTGCCACCACCATCACGGCCTTGCTGGC
TTCCTTGAATAGTTGCTGCAATCCCTGGATATACATGTTTTTTAGCGGTCATCTCCTGCAGG
ACTGTGTCCAAAGCTTCCCATGCTGCCAAAACATGAAGCAAACGTTCAGCAAAGAAGATTC
AGACAGTACGAGCAGAAGGCAGACTTCTTACACTAACAACCGAAGCCCGACAAATAGCAC
AGGTCCGTGGAAGGACTCTCCTAAATCTTCCAAGATCATCAGATTCATTCCGGTTTCAACC

>Ancient_horse

ATGCGTTTCTCCGGAGGCCCTGGCGCCGGGTCCACGGGCAACTCAAGCCGCTGGTGGCC
TCTGGCCGCCGGCGGTGCCAACGCCAGCCGCGAATCGGAGGCGCTCGGGGAAGAAGGC
GGCCAGCAGAGGGACTTGCGCAACGAGGAGCTGGCCAAGGTGGAGATCGCCGTGCTGG
CCGTGACTTTCGTGGTGGCCGTACTGGGTAACAGCTGCGTGTTGCTGGCACTGCATCGCA
CGCCTCGCAAGACGTCCCGCATGCACCTCTTCATCCGCCACCTCAGCCTGGCCGACCTGG
CCGTCGCTTTCTTCCAGGTGCTGCCGCAGCTGTGCTGGGACATCACCTACCGTTTCCGCG
GCCCCGACGGGCTGTGCCGCGTGGTGAAGCACCTGCAGGTGTTCGGCATGTTCGCCTCG
GCTTACATGCTGGTGGTCATGACCGCCGACCGCTACATCGCCGTGTGCCACCCGCTCAAG
ACGCTGCAGCAGCCGACGCGCCGCTCGCGCCTCATGATCGCGGCCGCCTGGGTGCTGAT
CTTCCACCTTAGCACGCCGCAGTACCTCGTCTTCTCCATGGTCGAGGATCTCAACGTCACC
AAGGCCTACGACTGCTGGGCCACCTTCATCCAGCCCTGGGGACCCCGCGCCTACGTGAC
TTGGATGACAGCCAGCATCTTCGTGGCGAGATTGGTCATCCTGGGCACCTGCTACGGCTT
CATCTGCTACCAGATCTGGCGCAACGTC---GTCAACAC----
TCGCTCTCCGATCTGGGCGCCGAGCGCGCGGGCGGCGCCTTCCGCAATGGGCTCCTGCT
CGAGCCCTGTGTCAGCAGCGTGAAGACCATCTCCCGAGCCAAGATCCGCACCGTGAAGAT
GACTTTCGTGATTGTGACCGCTTACATCGTTTGCTGGATGCCCTTCTTTATCGTACAGATGT
GGTCTGTCTGGGATGTGAAGTTCCACTGGATCGGATCGGAAGA-------
AGGACGGTGCAGGCTTGCATGGCTTCCTTGAATAGTTGCTGCAATCCCTGGATATACATGT
TTTTTAGCGGTCATCTCCTGCAGGACTGTGTCCAAAGCTTCCCATGCTGCCAAAACATGAA
GCAAACGTTCAGCAAAGAAGATTCAGABAGTACGAGCAGAAGGCAGACTTCTTACACTAAC
AACCGAAGCCCGACAAACAGCACAGGTCCGTGGAAGGACTCTCCTAAATCTTCCAAGATC
ATCAGATTCATTCCAGTTTCAACC

>Equus_przawelskii

ATGCGTTTCTCCGGAGGCCCTGGCGCCGGGTCCACGGGCAACTCAAGCCGCTGGTGGCC
TCTGGCCGCCGGCGGTGCCAACGCCAGCCGCGAATCGGAGGCGCTCGGGGAAGAAGGC
GGCCAGCAGAGGGACTTGCGCAACGAGGAGCTGGCCAAGGTGGAGATCGCCCTGCTGGC
CGTGACTTTCGTGGTGGCCGTACTGGGTAACAGCTGCGTGTTGCTGGCACTGCATCGCAC
GCCTCGCAAGACGTCCCGCATGCACCTCTTCATCCGCCACCTCAGCCTGGCCGACCTGGC
CGTCGCTTTCTTCCAGGTGCTGCCGCAGCTGTGCTGGGACATCACCTACCGTTTCCGCGG
CCCCGACGGGCTGTGCCGCGTGGTGAAGCACCTGCAGGTGTTCGGCATGTTCGCCTCGG
CTTACATGCTGGTGGTCATGACCGCCGACCGCTACATCGCCGTGTGCCACCCGCTCAAGA
CGCTGCAGCAGCCGACGCGCCGCTCGCGCCTCATGATCGCGGCCGCCTGGGTGCTGAG
CTTCGTGCTGAGCACGCCGCAGTACCTCGTCTTCTCCATGGTCGAGGTGAGCAACGTCAC
CAAGGCCTACGACTGCTGGGCCACCTTCATCCAGCCCTGGGGACCCCGCGCCTACGTGA
CTTGGATGACAGCCAGCATCTTCGTGGCGCCCGTGGTCATCCTGGGCACCTGCTACGGCT
TCATCTGCTACCACATCTGGCGCAACGTCCGCGGGAAGACGGCGTCGCGCCAGGACAAG
GGCGCCGAGCGCGCGGGCGGCGCCTTCCGCAATGGGCTCCTGCTCGAGCCCTGTGTCA
GCAGCGTGAAGACCATCTCCCGAGCCAAGATCCGCACCGTGAAGATGACTTTCGTGATTG
TGACCGCTTACATCGTTTGCTGGATGCCCTTCTTCATCGTCCAGATGTGGTCTGTCTGGGA
TGTGAAGTTCCACTGGATCGAGTCGGAAAACCCTGCCACCACCATCACGGCCTTGCTGGC
TTCCTTGAATAGTTGCTGCAATCCCTGGATATACATGTTTTTTAGCGGTCATCTCCTGCAGG
ACTGTGTCCAAAGCTTCCCATGCTGCCAAAACATGAAGCAAACGTTCAGCAAAGAAGATTC
AGACAGTACGAGCAGAAGGCAGACTTCTTACACTAACAACCGAAGCCCGACAAACAGCAC
AGGTCCGTGGAAGGACTCTCCTAAATCTTCCAAGATCATCAGATTCATTCCAGTTTCAACC

>Felis_catus

ATGCGTTTTTCCGGAGGCCCCAACCAGGGGCCCGCGGACAACTCCAGCCAGGGGTGGCC
CCTGTTAGCCGGCGGTGTCAACGGCAGCCGGGCAGAGGAGGCGCTCGGGGACGGCGGT
AGCCCCCAGGGGGACGTGCGCAACGAGGAGCTGGCCAAGCTGGAGATCGCCGTGCTGG
CTGTGACTTTCGTGGTAGCCGTGCTAGGCAACAGCAGCGTGTTGGTGGCGCTGCACCGCA
CCCCTCGCAAGACGTCCCGCATGCACCTCTTCATCCGCCACCTCAGCCTGGCCGACCTGG
CCGTCGCTTTCTTCCAGGTGCTGCCGCAGATGTGCTGGGACATCACCTACCGTTTCCGCG
GGCCCGACGGGCTGTGCCGCGTGGTGAAGCACCTGCAGGTGTTCGGCATGTTCGTGTCG
CCCTACATGCTGGTGGTCATGACCGCCGACCGCTACATTGCGGTGTGCCACCCGCTGAAG
ACGCTGCAGCAACCGACGCGCCGCTCGCGCCTCATGATCGCTGCCGCCTGGGTGCTGAG
CTTCGTGCTTAGCACGCCGCAGTACTTCGTCTTCTCCATGGTCGAGGTGAACAACGTCACC
AAGGCCCGGGACTGCTGGGCCACCTTCATCCAGCCCTGGGGTCCCCGCGCCTATGTGAC
CTGGATGACGGGTGGCATCTTCGTGGCGCCCGTGGTCATCCTGGGTACCTGCTATGGCTT
CATCTGCTACCACATCTGGCGCAACGTCCGCGGAAAGACGGCTTTGCTCCCCGGAAAGGG

CGCCGAGGGCGCGAGTGGCGCTTTGCATAAGGGGTTCCTGCTCGCGCCGTGTATCAGCA
GCGTGAAGACCATTTCCCGCGCCAAGATTCGCACGGTGAAGATGACTTTTGTGATCGTGA
CCGTTTACATCGTTTGCTGGGCGCCCTTCTTCATCCTTCAGATGTGGTCTGTCTGGGATGA
GAAGTTTGTTTGGATCGAATCAGAAAACCCGGCCATCACCATCACTGCATTACTGGCTTCC
TTGAATAGTTGCTGTAATCCCTGGATATACATGTTTTTTAGCGGCCATCTCCTGCAAGACTG
TGTCCACAGCTTCCCATGCTGCCAGAACATGAATCAAACGTTCAACAAAGAAGATTCTGAC
AGTATGAGCAGAAGACAGACTTCTTACACTAACAACCGGAGCCCCACAAACAGTATGGGTA
CATGGAAGGACTCGCCTAAATCGTCCAAGTCCATCAAATTCATTCCTGTTTCAACC

>Felis_silvestris

ATGCGTTTCTCCGGAGGCCCCAACCAGGGGCCCGCGGACAACTCCAGCCAGGGGTGGCC
CCTGTTAGCCGGCGGTGTCAACGGCAGCCGGGCAGAGGAGGCGCTCGGGGACGGCGGC
AGCCCCCAGGGGGACGTGCGCAACGAGGAGCTGGCCAAGCTGGAGATCGCCGTGCTGG
CTGTGACTTTCGTGGTAGCCGKGSTAGGCAACAGCAGCGTGTTGGTGGCGCTGCACCGCA
CCCCTCGCAAGACGTCCCGCATGCACCTCTTCATCCGCCACCTCAGCCTGGCCGACCTGG
CCGTCGCTTTCTTCCAGGTGCTGCCGCAGATGTGCTGGGACATCACCTACCGTTTCCGCG
GGCCCGACGGGCTGTGCCGCGTGGTGAAGCACCTGCAGGTGTTCGGCATGTTCGTGTCG
CCCTACATGCTGGTGGTCATGACCGCCGACCGCTACATTGCGGTGTGCCACCCGCTGAAG
ACGCTGCAGCAACCGACGCGCCGCTCGCGCCTCATGATCGCTGCCGCCTGGGTGCTGAG
CTTCGTGCTTAGCACGCCGCAGTACTTCGTCTTCTCCATGGTCGAGGTGAACAACGTCACC
AAGGCCCGGGACTGCTGGGCCACCTTCATCCAGCCCTGGGGTCCCCGCGCCTATGTGAC
CTGGATGACGGGTGGCATCTTCGTGGCGCCCGTGGTCATCCTGGGTACCTGCTATGGCTT
CATCTGCTACCACATCTGGCGCAACGTCCGCGGAAAGACGGCTTTGCTCCCCGGAAAGGG
CGCCGAGGGCGCGAGTGGCGCTTTGCATAAGGGGTTCCTGCTCGCGCCGTGTATCAGCA
GCGTGAAGACCATTTCCCGCGCCAAGATTCGCACGGTGAAGATGACTTTTGTGATCGTGA
CCGTTTACATCGTTTGCTGGGCGCCCTTCTTCATCCTTCAGATGTGGTCTGTCTGGGATGA
GAAGTTTGTTTGTACAGAATCAGAAAACCCGGCCATCACCATCACTGCATTACTGGCTTCC
TTGAATAGTTGCTGTAATCCCTGGATATACATGTTTTTTAGCGGCCATCTCCTGCAAGACTG
TGTCCACAGCTTCCCATGCTGCCAGAACATGAATCAAACGTTCAACAAAGAAGATTCTGAC
AGTATGAGCAGAAGACAGACTTCTTACACTAACAACCGGAGCCCCACAAACAGTATGGGTA
CATGGAAGGACTCGCCTAAATCGTCCAAGTCCATCAAATTCATTCCTGTTTCAACC

>Ailuropoda_melanoleuca

ATGCGTATCTCCGGAGGCCCCGCCGCGGTGTCCGCGGACAACTCCAGCCAGTGGTGGCC
TCTGTCCGCCGGTGGTGCCAACGGTAGCGGGGAAGCGGAGGCGCTCGGGGAAGGCCGC
AGCCCACCGGGGGACGTGCGCGACGAGGAGCTGGCCAAGGCGGAGATCGCCGTGCTGG
CTGTGACTTTCGTGGTGGCCGTGCTAGGTAACAGCAGCGTGTTGGTGGCGCTGCACCGCA
CGCCTCGCAAGACGTCCCGCATGCACCTTTTCATCCGTCACCTCAGCCTAGCCGACCTGG
CTGTCGCTTTCTTCCAGGTGCTGCCGCAGATGTGCTGGGATATCACCTACCGTTTCCGCG
GACCCGACGTACTGTGCCGCGTGGTGAAGCATCTGCAGGTGTTTGGCATGTTCGTATCGC
CTTACATGCTGGTGGTCATGACCGCCGACCGCTACATTGCCGTGTGCCACCCGCTGAAGA
CGCTGCAACAGCCAACGCGCCGCTCGCGCCTCATGATCGCTGCTGCCTGGGTGCTGAGC
TTCGTGCTTAGCACGCCGCAGTACTTCGTCTTCTCCATGGTCGAGGTGGACAACGTCACCA
AGGCCGTTGACTGCTGGGCCACCTTCATTCAGCCCTGGGGTCCCCGCGCCTATGTGACTT
GGATGACGGGAGGCATCTTCGTGGCGCCGGTGGTCATCCTGGGCACCTGCTATGGCTTC
ATCTGCTACCACATCTGGCGCAATGTCCGCGGAAAGACGGCTTTGCGACCGGGGAAGTGC
GCGGAGGGCGCGCGTGGCGCCTTCCATAAGGGGTTCCTGCTGGCGCCGTGTATCAGCAG
CGTGAAAAACATTTCCCGCGCCAAGATCCGCACCGTGAAGATGACTTTTGTGATCGTGACA
GTTTACATCGTTTGCTGGGCGCCTTTCTTCATCCTCCAGATGTGGTCTGTCTGGGATGACA
AACTTGTCTGGGTCGAATCGGAAAACCCAGCTGTCACCATCACGGCATTACTGGCTTCCTT
GAACAGTTGCTGTAATCCCTGGATCTACATGTTTTTCAGTGGCCACCTCCTGCAGGACTGT
GTCCAGAGTTTCCCGTGCTGCCAAAGCATGAAGCAAACATTCAACAAAGAAGATTCTGACA
GTGTGAGCAGAAGACAGACTTCTTATACTAACAACCGAAGCCCGACCAACAGTATGGGTAC
ATGGAAGGACTCACCTAAATTTTCCAAGTCCGTCAAATTCATTCCTGTTTCCACC

>Mustela_putorius_furo

ATGCGTGTCTCCGGAGGTCCCGCCGCGGTGTCCGCGGACAACTCCAGCGAGTGGTGGCC
TCTGTCCGCGGGTGGTGCCAACCGTAGCGGAGAAGCGGAGGCGCCTGGGGGAGGCCGC
AGCCCTCCGGGGGACGTGCGCGACGAGGAGCTGGCCAAGGCGGAGATCGCCGTGCTGG

CTGTGACTTTCGTGGTGGCAGTGCTGGGTAACAGCAGTGTGTTGGTCGCGCTGCACCGCA
CGCCCCGCAAGACGTCCCGAATGCACCTCTTCATCCGTCACCTCAGTCTAGCCGACCTAG
CCGTCGCTTTCTTCCAGGTGCTGCCGCAGATGTGCTGGGATATCACCTACCGCTTCCGCG
GACCCGACGCCCTGTGCCGCGTGGTGAAGCATCTGCAGGTGTTCGGCATGTTCGTATCGC
CCTACATGCTGGTGGTTATGACTGCCGACCGCTACATCGCTGTGTGCCACCCGCTGAAAA
CGCTGCAACAGCCAACGCGCCGCTCGCGCCTCATGATCGCCGCTGCCTGGGTGCTGAGC
TTCGTGCTTAGCACGCCACAGTACTTCGTCTTCTCCATGGTCGAGGTGGACAACGTCACCA
AGGCCATTGACTGCTGGGCCACCTTCATCCAACCCTGGGGTCCCCGCGCCTATGTGACTT
GGATGACAGGTGGCATCTTCGTGGCGCCCGTGGTCATCCTCGGCACCTGCTATGGCTTCA
TCTGCTACCACATCTGGCGCAATGTCCGCGGAAAGACGGCTTTGCGCCCGGGAAAGGGC
GCGGAGGGCGCCAGTGGCGCCCTCCATAAAGGGTTCCTACTCGCGCCCTGTATCAGCAG
CGTGAAAACCATTTCCCGCGCGAAGATCCGCACCGTGAAGATGACTTTTGTGATCGTGACA
GTTTACATCGTTTGCTGGGCCCCCTTCTTCATCCTCCAGATGTGGTCTGTCTGGGATGACA
AACTTGTCTGGGTCGAATCGGAAAACCCAGCCGTCACCATCACGGCGTTACTAGCTTCCCT
GAATAGTTGCTGCAATCCTTGGATCTACATGTTTTTCAGTGGCCACCTCTTGCAAGACTGCA
TCCAGAGTTTCCCATGCTGCCAAAGCATGAAGCAAACATTGAACAAAGAAGATTCTGACAG
TGTGAGCAGAAGACAGACTTCTTACACAAACAACCGAAGCCCGACCAACAGTATGGTTACA
TGGAAGGACTCGCCCAAGTTTTCCAAGTCCGTCAAATTCATTCCTGTTTCCACC

>Canis_lupus

ATGCGCATCCCCGGGGGCCCCGGCGCCCCGTCCGCCGGCAACTCCAGCCGCTGGTGGC
CTGCGTCCGGCCGCGGAGCCAACGCGAGCGGGGACGCGGGGGCGCTGGCGGACGGCG
ACGGGCCCCCGGGGGACGCGCGCAACGAGGAGCTGGCCAAGCTGGAGATCGCCGTGCT
GGCCGTGACCTTCGTGGTGGCCGTGCTGGGCAACAGCAGCGTGCTGGTGGCGCTGCACC
GCACGCCCCGCAAGACGTCCCGCATGCACCTGTTCATCCGCCACCTGAGCCTGGCCGAC
CTGGCCGTCGCCTTCTTCCAGGTGCTGCCGCAGATGTGCTGGGACATCACCTACCGCTTC
CGCGGGCCCGACGGCCTGTGCCGCGTGGTGAAGCACCTGCAGGTGTTCGGCATGTTCGT
GTCGCCCTACATGCTGGTGGTCATGACCGCCGACCGCTACATCGCCGTGTGCCACCCGCT
GAAGACGCTGCAGCAGCCCACGCGCCGCTCGCGCCTCATGATCGCCGCCGCCTGGGTGC
TGAGCTTGGTGCTCAGCACGCCGCAGTACTTGGTCTTCTCCATGGTGGAGGTGAACAACG
TCACCAAGGCCAACGACTGCTGGGCCACCTTCATCCAGCCGTGGGGGCCCCGCGCCTAC
GTGACCTGGATGACGGCTGGCATCTTCGTGGCGCCCGTGGTGCTCCTGGCCACCTGCTA
CGGCTGCATCTGCTCGCACATCTGGCGCAGCGTCCGCGGCAGGACGGCCCTGCG-----------
-
CGCGGGGGCGGCGGCGGGCGCGGTCCCGAGGGGCCTCCTGCGGGCGCCCGGGGCCAG
CAGCGTGAAGACCATTTCCCGCGCCAAGATGCGCACCGTGAAGATGACTTTCGTGATCGT
GACCGTGTACATCGTTTGCTGGGCGCCCTTCTTCATCCTCCAGATGTGGTCCGTCTGGTCC
GCTTATGTTTTTCGTACAGAATCAGAAAACCCAGCCATCACCATCACCGCATTACTGGCTTC
CCTGAACAGTTGCTGCAATCCCTGGATCTACATGTTTTTCAGTGGCCATCTCCTGCAAGAC
TGTGTGCAGAGCTTCCCATGCTGCCAAAACATGAAGCAAACATTCAACAAAGTAGATTCTG
ACAGTGTGAGCAGAAGACAGACTTCCTATACTAACAACCGAAGCCCGACAAACAGTATGG
GCACATGGAAGGACTCCCCTAAAACTTCCAAGTCCGTCAAATTCATTCCTGTTTCAACC

>Canis_lupus_familiaris

ATGCGCATCCCCGGGGGCCCCGGCGCCCCGTCCGCCGGCAACTCCAGCCGCTGGTGGC
CTGCGTCCGGCCGCGGAGCCAACGCGAGCGGGGACGCGGGGGCGCTGGCGGACGGCG
GCGGGCCCCCGAGGGACGCGCGCAACGAGGAGCTGGCCAAGCTGGAGATCGCCGTGCT
GGCCGTGACCTTCGTGGTGGCCGTGCTGGGCAACAGCAGCGTGCTGGTGGCGCTGCACC
GCACGCCCCGCAAGACGTCCCGCATGCACCTGTTCATCCGCCACCTGAGCCTGGCCGAC
CTGGCCGTCGCCTTCTTCCAGGTGCTGCCGCAGATGTGCTGGGACATCACCTACCGCTTC
CGCGGGCCCGACGGCCTGTGCCGCGTGGTGAAGCACCTGCAGGTGTTCGGCATGTTCGT
GTCGCCCTACATGCTGGTGGTCATGACCGCCGACCGCTACATCGCCGTGTGCCACCCGCT
GAAGACGCTGCAGCAGCCCACGCGCCGCTCGCGCCTCATGATCGCCGCCGCCTGGGTGC
TGAGCTTGGTGCTCAGCACGCCGCAGTACTTGGTCTTCTCCATGGTGGAGGTGAACAACG
TCACCAAGGCCAACGACTGCTGGGCCACCTTCATCCAGCCGTGGGGGCCCCGCGCCTAC
GTGACCTGGATGACGGCTGGCATCTTCGTGGCGCCCGTGGTGCTCCTGGCCACCTGCTA
CGGCTGCATCTGCTCGCACATCTGGCGCAGCGTCCGCGGCAGGACGGCCCTGCG-----------
-
CGCGGGGGCGGCGGCGGGCGCGGTCCCGAGGGGCGTCCTGCGGGCGCCAGGGGCCAG
CAGCGTGAAGACCATTTCCCGCGCCAAGATGCGCACCGTGAAGATGACTTTCGTGATCGT

GACCGTGTACATCGTTTGCTGGGCGCCCTTCTTCATCCTCCAGATGTGGTCCGTCTGGGA
CGACGGGTTTGTTTGGATCGAATCAGAAAACCCAGCCATCACCATCACGGCATTACTGGCT
TCCCTGAACAGTTGCTGCAATCCCTGGATCTACATGTTTTTCAGTGGCCATCTCCTGCAAG
ACTGTGTGCAGAGCTTCCCATGCTGCCAAAACATGAAGCAAACATTCAACAAAGTAGATTC
TGACAGTGTGAGCAGAAGACAGACTTCCTATACTAACAACCGAAGCCCGACAAACAGTATG
GGCACATGGAAGGACTCCCCTAAAACTTCCAAGTCCGTCAAATTCATTCCTGTTTCAACC

4. Alignment of the mammalian species used in the *AVPR1b* analyses.

>Homo_sapiens

ATGGATTCTGGGCCTCTGTGGGATGCCAACCCCACCCCTCGGGGCACCCTCTCTGCCCCC
AATGCCACAACACCCTGGCTGGGCCGGGATGAGGAGCTGGCCAAGGTGGAGATCGGAGT
CCTGGCCACTGTCCTGGTGCTGGCGACCGGGGGCAACCTGGCTGTGCTGCTGACCCTGG
GCCAGCTGGGCCGCAAGCGCTCCCGCATGCACCTGTTCGTGCTGCACTTAGCCCTGACA
GACCTGGCCGTGGCGCTCTTCCAGGTGCTGCCACAGCTGCTGTGGGACATCACCTACCG
CTTCCAGGGCCCCGACCTCCTGTGCAGGGCCGTCAAGTACCTGCAGGTGCTCAGCATGTT
TGCCTCCACCTACATGCTGCTGGCCATGACGCTGGACCGCTACCTGGCTGTCTGTCACCC
CCTGCGCAGCCTCCAGCAGCCAGGCCAGTCCACCTACCTGCTCATCGCTGCTCCCTGGCT
GCTGGCCGCCATCTTCAGCCTCCCTCAAGTCTTCATTTTTCCCTGCGGGAGGTGATCCAG
GGCTCAGGGGTGCTGGACTGCTGGGCAGACTTCGGCTTCCCTTGGGGGCCACGGGCCTA
CCTCACCTGGACCACCCTGGCTATCTTCGTTCTGCCGGTGACCATGCTCACGGCCTGCTA
CAGCCTCATCTGCCATGAGATCTGTAAAAACCTAAAAGTCAAGACACAGGCCTGGCGGGT
GGGAGGAGGGGGCTGGAGGACTTGGGACAGGCCCTCACCTTCCACCTTAGCTGCCACCA
CTCGGGGGCTGCCATCTCGGGTCAGCAGCATCAACACCATCTCACGGGCCAAGATCCGAA
CAGTGAAGATGACCTTTGTCATCGTGCTGGCCTACATCGCTTGCTGGGCTCCCTTCTTCAG
TGTCCAGATGTGGTCCGTGTGGGACAAGAATGCCCCTGATGAAGATTCCACCAATGTGGC
TTTCACCATCTCTATGCTTTTGGGCAACCTCAACAGCTGCTGCAACCCCTGGATCTACATG
GGCTTCAACAGCCACCTGTTACCGCGGCCCCTGCGTCACCTTGCCTGCTGTGGGGGTCCC
CAGCCCAGGATGCGCCGGCGGCTCTCCGACGGCAGCCTCTCGAGCCGCCACACCACGCT
GCTGACCCGCTCCAGCTGCCCGGCCACCCTCAGCCTCAGCCTCAGCCTAACCCTCAGTG
GGAGGCCCAGGCCTGAAGAGTCACCAAGGGACTTGGAGCTGGCAGATGGGGAAGGCACC
GCTGAGACCATCATCTTT--

>Pan_troglodytes

ATGGATTCTGGGCCTCTGTGGGATGCCAACCCCACCCCTCGGGGCACCCTCTCTGCCCCC
AATGCCACAACACCCTGGCTGGGCCGGGATGAGGAGCTGGCCAAGGTGGAGATCGGAGT
CCTGGCCACTGTCCTGGTGCTGGCGACCGGGGGCAACCTGGCTGTGCTGCTGACCCTGG
GCCAGCTGGGCCGCAAGCGCTCCCGCATGCACCTGTTCGTGCTGCACCTAGCCCTGACA
GACCTGGCTGTGGCGCTCTTCCAGGTGCTGCCACAGCTGCTGTGGGACATCACCTACCGC
TTCCAGGGCCCCGACCTCCTGTGCAGGGCCGTCAAGTACCTGCAGGTGCTCAGCATGTTT
GCCTCCACCTACATGCTGCTGGCCATGACGCTGGACCGCTACCTGGCTGTCTGTCACCCC
CTGCGCAGCCGCCAGCAGCCAGGCCAGTCCACCTACCTGCTCATCGCTGCTCCCTGGCT
GCTGGCCGCCATCTTCAGCCTCCCTCAAGTTTTCATTTTTCCCTGCGGGAGGTGATCCAG
GGCTCAGGGGTGCTGGACTGCTGGGCAGACTTCCGCTTCCCTTGGGGGCCACGGGCGTA
CCTCACCTGGACCACCCTGGCTATCTTCGTTCTGCCGGTGACCATGCTCACGGCCTGCTA
CAGCCTCATCTGCCATGAGATCTGTAAAAACCTAAAAGTCAAGACACAGGCCTGGCGGGT
GGGAGGAGGGGTCTGGAGGACTTGGGACAGGCCCTCACCTTCCACCTCAGCTGCCACCA
CTCGGGGGCTGCCATCTCGGGTCAGCAGTATCAACACCATCTCACGGGCCAAGATCCGAA
CAGTGAAGATGACCTTTGTCATCGTGCTGGCCTACATCGCTTGCTGGGCTCCCTTCTTCAG
TGTCCAGATGTGGTCCGTGTGGGACAAGAATGCCCCTGATGAAGATTCCACCAATGTGGC
TTTCACCATCTCTATGCTTTTGGGCAACCTCAACAGCTGCTGCAACCCCTGGATCTACATG
GGCTTCAACAGCCACCTGTTACCGCGGCCCCTGCGTCACCTTGCCTGCTGTGGGGGTCCC
CAGCCCAGGATGCGCCGGCGGCTCTCCGACGGCAGCCTCTCGAGCCGCCACACCACGCT
GCTGACCCGCTCCAGCTGCCCGGCCACCCTCAGCCTCAGCCTCAGCCTAACCCTCAGTG
GGAGGCCCAGGCCTGAAGAGTCACCAAGGGACTTGGAGCTGGCGGATGGGGAAGGCAC
CGCTGAGACCATCATCTTT--

>Mus_musculus

ATGGATTCTGAGCCTTCTTGGACTGCTACCCCCTCCCCTGGGGGCACCCTGTTTGTCCCC
AATACCACCACACCCTGGCTGGGTAGGGATGAAGAGCTAGCCAAGGTGGAAATTGGTATC

CTAGCTACTGTCCTGGTTCTGGCCACAGGAGGCAACCTGGCTGTACTACTGATTCTGGGC
CTCCAGGGCCATAAGCGCTCCCGCATGCATCTGTTCGTGCTGCACTTGGCCCTGACCGAC
CTGGGCGTGGCGCTTTTCCAGGTACTGCCTCAGCTGCTCTGGGACATCACCTATCGCTTC
CAGGGCTCTGACCTCCTCTGCCGGGCTGTCAAATATCTACAGGTGCTCAGCATGTTTGCTT
CCACTTACATGCTGCTGGCCATGACGCTGGACCGGTACCTGGCTGTCTGTCACCCTCTTC
GAAGCCTCCAGCAGCCCAGCCAGTCCACCTACCCTCTTATTGCTGCTCCCTGGCTGCTGG
CTGCCATCCTCAGCCTCCCTCAAGTGTTCATTTTTTCGTTGCGAGAGGTGATCCAGGGCTC
AGGGGTGCTGGACTGCTGGGCAGATTTCTACTTTTCCTGGGGCCCGCGGGCCTACATCAC
CTGGACCACCATGGCCATCTTTGTGCTGCCCGTGGTGGTGCTCACAGCTTGCTATGGCCT
CATCTGCCACGAGATCTACAAGAACCTGAAAGTCAAGACACAGGCTGGCAGGGAGGAAAG
AAGGGGCTGGC---------
CCAAGTCCTCATCTTCTGCCGCTGCTGCAGCCACTAGGGGGCTGCCGTCCCGGGTCAGCA
GCATCAGTACCATCTCCAGGGCAAAGATCCGAACCGTGAAGATGACCTTTGTCATTGTGCT
GGCCTACATTGCTTGCTGGGCACCTTTCTTCAGTGTCCAGATGTGGTCTGTGTGGGACGA
GAATGCCCCTAATGAAGATTCTACCAATGTGGCTTTCACCATCTCGATGCTTCTGGGCAAC
CTCAGCAGCTGCTGCAACCCCTGGATCTATATGGGCTTCAACAGCCACCTGTTGCCACGTT
CCCTGAGTCACCGCGCCTGCTGCAGGGGTTCCAAGCCCCGAGTGCACAGGCAGCTCTCC
AACAGCAGCCTCGCTAGCCGCCGCACAACACTGCTGACCCACACCTGTGGTCCATCTACT
CTCCGTCTTAGCCTTAA------
CCTCCATGCAAAGCCTAAGCCTGCGGGCTCGCTGAAGGATTTGGAGCAGGTGGATGGAGA
AGCCACTATGGAGACCAGCATCTCT--

>Peromyscus_maniculatus

ATGGAGTCTGAGCCTTCTTGGACTGCCACTCCCTCTCCTGGGGGCACTCTGTCTGTCCCC
AATGCTACCACACCCTGGCTGGGCAGGGATGAAGAGCTAGCCAAGGTGGAGATTGGGATC
CTAGCTACTGTCCTGGTTCTGGCCACAGGGGGCAACCTGGCTGTGCTGTTGGCACTGGGC
TTCCAGGGCCGCAAGCGTTCCCGCATGCACCTGTTCGTGCTGCACTTGGCCCTGACGGAT
CTGGGCGTGGCGCTTTTCCAGGTACTGCCTCAGCTGCTCTGGGATATCACCTACCGCTTC
CAGGGCTCTGACACCCTTTGCCGGCTTGTCAAGTACCTGCAGGTGCTCAGCATGTTCGCT
TCCACTTACATGCTGCTGGCCATGACGCTGGACCGGTACCTGGCTGTCTGTCACCCCCTT
CGTAGCCTCCAGCAGCCCAGCCAGTCCACCTACCCTCTCATTGCGGCTCCCTGGCTGCTG
GCTGCCATCCTCAGCCTTCCTCAGATTTTCATTTTTCTCTGCGAGAGGTCATCCAGGGCT
CGGGGGTCCTGGACTGCTGGGCAGAGTTCTACTTTTCTTGGGGGCCACGGGCCTACATCA
CCTGGACCACTGTGGCCATCTTTGTGCTCCCCGTGGTTGTGCTCACAGCCTGTTACAGCCT
CATCTGCTGTGAGATCTACAAGAACCTGAAAGTCAAGACACAGGCTGGCAGAGAGGAAAA
AAGTGGCTGGAGGACTTGGGACAAGTCTTCATCTCCGGCCCCTGCTGATGCCACTAGAGG
GCTGCCATCCCGGGTCAGCAGCATCAGCACCATCTCCAGAGCAAAGATCCGAACTGTGAA
GATGACCTTTGTGATTGTGCTGGCCTACATCGCCTGCTGGGCACCCTTCTTCAGTGTCCAG
ATGTGGTCTGTGTGGGACGAGAACGCCCCCAATGAAGATTCCACCAACGTGGCTTTCACC
ATCTCAATGCTTTTGGGCAACCTCAGCAGCTGCTGCAACCCCTGGATCTACATGGCCTTCA
ACAGCCACCTGCTGCCACGTTCCCTGAGTCGCCGGGTCTGCTGCAGGGGTTCCCAGCGC
CGGGTGCACAGGCAACTCTCCGATAGCAGCCTGGCTAGCCGCCGCACGACGCTGCTGAC
CCACTCCAGCGGTCCGTCCACCCTCCGTCTCAGCCTTCA------
CCTCCAGGCAAAGCCCAGGCCTGCAGAGCCACAGAAGGATCTAGAGCAGGGGGATGGAG
AAGCCGCCGTGGAGACCAGCATCTTT--

>Rattus_norvegicus

ATGAATTCTGAGCCTTCTTGGACTGCCACCCCCTCCCCTGGGGGCACCCTGCCTGTCCCC
AATGCTACCACACCGTGGCTAGGTAGGGATGAAGAGCTAGCCAAGGTGGAGATCGGTATC
CTAGCTACTGTCCTGGTTCTGGCCACAGGAGGCAACCTGGCTGTACTTCTGACTCTGGGC
CGCCATGGCCACAAGCGCTCCCGCATGCATCTGTTCGTGCTGCACTTGGCCCTGACCGAC
CTGGGCGTGGCGCTTTTCCAGGTACTGCCTCAGCTGCTCTGGGACATCACCTACCGCTTC
CAGGGCTCTGACCTCCTCTGCCGGGCTGTCAAGTATCTACAGGTGCTCAGCATGTTTGCTT
CCACTTACATGCTGCTGGCCATGACACTGGACCGGTACCTGGCTGTCTGTCACCCTCTTC
GAAGCCTCCGGCAGCCCAGCCAGTCTACCTACCCTCTCATTGCTGCTCCCTGGTTGCTGG
CTGCCATCCTCAGCCTCCCTCAAGTTTTCATTTTTTCATTGCGAGAGGTGATCCAGGGCTC
AGGGGTGCTGGACTGCTGGGCAGATTTCTACTTTTCCTGGGGCCCGCGGGCCTACATCAC
CTGGACCACCATGGCTATCTTTGTGCTGCCGGTGGCGGTGCTCACAGCTTGCTACGGCCT
CATCTGCCACGAGATCTACAAGAACCTGAAAGTCAAGACCCAGGCTGGCAGGGAGGAGAG
GAGGGGCTGGCGGACTTGGGATAAGTCCTCATCTT---------

CTGCAGCCACGAGGGGGCTACCTTCCCGGGTCAGCAGCATCAGTACCATCTCCAGGGCA
AAGATCCGAACCGTGAAGATGACCTTTGTCATCGTGCTGGCCTACATTGCTTGCTGGGCAC
CTTTCTTCAGTGTCCAGATGTGGTCTGTGTGGGATGAGAATGCCCCCAATGAAGATTCTAC
CAATGTGGCTTTCACCATCTCCATGCTTCTGGGCAACCTCAGCAGCTGCTGCAACCCCTGG
ATCTACATGGGCTTCAACAGTCGCCTGTTGCCACGTTCCTTGAGCCACCATGCCTGCTGCA
CGGGCTCTAAGCCCCAAGTGCACAGGCAACTCTCCACCAGCAGCCTCACTAGCCGTCGCA
CTACGCTGCTGACCCATGCCTGTGGTTCCCCTACTCTCCGACTCAGCCTTAA------
CCTCCGTGCAAAGCCCAGACCTGCAGGCTCACTGAAGGATTTAGAGCAGGTGGACGGGG
AAGCCACTATGGAGACCAGCATCTTT--

>Mesocricetus_auratus

ATGGATTCTGAGCCTTTCTGGATTGTCACTCCCTCTCCCGGGAGCACCCCGTCTGTCCCCA
ATGCCACCACGCCCTGGCTGGGCAGGGATGAAGAGCTAGCCAAGGTGGAGATCGGCATC
CTAGCTACTGTCCTGGTTCTGGCCACGGGGGGCAACCTGGCTGTACTCCTGACGCTGGG
GTGCCAGGGTCACAAGCGTTCCCGCATGCATCTGTTTGTGCTGCACTTGGCCCTGACTGA
CCTGGGCGTGGCGCTTTTCCAGGTGCTGCCTCAGCTGCTCTGGGATATCACCTACCGCTT
CCAGGGCTCTGACACCCTTTGCCGGGCCGTCAAGTATCTGCAGGTGCTCAGCATGTTTGC
TTCCACTTACATGCTGCTGGCCATGACACTGGACCGATACCTGGCTGTCTGCCACCCCCTT
CGCAGCCTCCAGCAGCCCAGCCAGGCCACCTACCCTCTCATTGCTGCTCCCTGGCTGCTG
GCTGCCGTCTTCAGCCTCCCTCAAGTTTTCATTTTTTCTTTGCGAGAGGTGATCCAGGGCT
CAGGGGTGCTGGACTGCTGGGCAGATTTCTACTTTTCTTGGGGGCCGCGGGCCTACATCA
CCTGGACCACCATGGCCATCTTTGTGCTACCTGTGGCGGTGCTCACAGCCTGTTACAGTCT
CATCTGCCGCGAGATCTACAAGAACCTTAAAGTGAAGACACAGGCTGGCAGGGAGCAAAG
AAGGGGCTGGAGGACTTGGGACAAGTC------
TTCTGCCCCTGCTGCAGCCCCTAGAGGTCTGCCATCCAGGGTCAGCAGCATCAGCACCAT
CTCCAGGGCAAAGATCCGAACTGTGAAGATGACCTTTGTCATTGTGCTGGCCTACATCGCT
TGCTGGGCACCCTTCTTCAGTGTCCAGATGTGGTCTGTGTGGGATGAGCATGCCCCTAAC
GAAGATTCCACCAATGTGGCTTTTACCATCTCAATGCTTTTGGGCAACCTCAGCAGCTGCT
GCAACCCCTGGATCTACATGGGCTTCAACAGCCACCTGCTGCCACGTTCCCTGAGTCACC
GCATCTGCTGCAGGGGTTCCCAGCCCCGAGTGCACAGGCAACTCTCCAGCAGCAGCCTT
GCTAGCCGCCGCACGACACTGCTGGCCCACTCCAGTGGTCCATCTACCCTCCGTCTCAGC
CTTAA------
CCTCCACTCAAAGCCCAGGCCTGCAGAGTCCCTGAAGGATTTAGAGCAGGGGGATGGAGA
AGCCTCCGTGGAGACCAGCATCTCT--

>Microtus_ochrogaster

ATGGATTCTGAATCTTCTTGGACTGCCACTCCCTCCCCTGGGGGCACTCTGTCTGTCCCCA
ATGCTACCATACCCTGGCTGGGCAGGGATGAAGAGCTAGCCAAAGTGGAGATTGGTATCC
TAGCTACTGTCCTGGTTCTGGCCACAGGGGGCAACCTGGCTGTACTGCTGATGCTGGGCT
GCCAGGGCCGCAAGCGTTCCCGCATGCACCTGTTTGTGCTGCACTTGGCCCTGACTGACC
TGGGAGTGGCAATTTTCCAGGTACTGCCTCAGTTGCTCTGGGATATTACCTACCGCTTCCA
GGGCTCTGACCTCCTCTGCCGGGCCGTCAAGTATCTGCAGGTGCTCAGCATGTTTGCTTC
CACTTACATGCTGCTGGCCATGACGCTGGACCGGTACCTGGCTGTCTGTCACCCCCTTCG
CAGCCTCCAGCAACCCAGCCATTCCACCTACCCTCTCATTGCTGCCCCCTGGCTGCTGGC
TGCCATCCTCAGCCTCCCTCAAATTTTCATTTTTCTCTGCGAGAGGTGATCCAGGGCTCA
GGGGTGTTGGACTGCTGGGCAGATTTCTACTTTTCTTGGGGGCCACGGGCCTACATCACC
TGGACCACCATGGCCATCTTTGTGCTACCTGTGGCCATGCTCACAGCTTGTTACAGCCTCA
TTTGCTGTGAGATCTACAAGAACCTTAAAGTAAAGACACAGGCTGGCAGGGAGGAAAAAAA
GGGCTGGAGGACTAGGGACAGGTCCTCATCTTCTTCCCCTGCTGTGGCCACTAGAAGGCT
GCCGTCACGGGTCAGCAGCATCAGCACCATCTCCAGGGCAAAGATCCGAACTGTGAAGAT
GACCTTTGTCATCGTGCTGGCCTACATCGCTTGCTGGGCACCCTTCTTCAGTGTCCAGATG
TGGTCTGTGTGGGATGAGGATGCCCCTAATGAAGATTCCACCAATGTGGCTTTCACCATCT
CCATGCTTCTGGGCAACCTCAGCAGCTGCTGCAACCCCTGGATCTACATGGGCTTCAACA
GCCACCTGCTGCCACGTTCCCTGAGTCACCGTGTCTGCTGCAGGGGTTCCCAGCCCCGG
GTGCACAGGCAACTCTCCAACAGCAGCCTTGCTAGCCGCCGCACGACACTACTGACCCAC
TCCGGTGGTCCCTCCACCCTCCGTCTCAGCCTTAA------
CCTCCATGCGAAGCCCAGGCCTGCCGAGTCACAGAAGGGTTTAGAGCAGAGGGATGGAG
AAGCCATCGTGGAGACCAGCATCTTT--

>Ictiodomys_tridecemlineatus

ATGGATTTGGAGCCTTCCTGGACTGCCATACCCAACCCTGGGGGCACCCTTTCTGCCCCC
AATGCCACAACACCCTGGCTGGGCCGGGATGAGGAGCTGGCCAAGGTGGAGATTGGCAT
CCTGGCTACCGTCCTGGTGCTGGCCACAGGGGGCAACCTGGCTGTGCTGCTGACCCTGG
GCCAGCCAGGCCGCAAGCGCTCCCGCATGCACCTGTTTGTGCTGCACCTGGCCCTGACT
GACCTGGGTGTGGCACTCTTCCAGGTGCTGCCCCAGCTGCTCTGGGACATCACCTACCGC
TTCCAGGGTCCTGACCTCCTCTGCCGGGCTGTCAAGTACCTGCAGGTGCTCAGCATGTTT
GCCTCCACCTACATGCTGCTGGCCATGACAGTGGACCGATACCTGGCCGTCTGTCATCCT
CTGCGCAGCCTCCAGCAGCCCAGCCAGTCCACTTACCCTCTCATCGCGGCTCCCTGGCTG
CTGGCTGCCATCCTTAGCCTCCCCCAAGTCTTCATTTTCTCTTTGCGGGAGGTGATCCAGG
GCACAGGGGTGCTGGACTGCTGGGCTGATTTCCGCTTCTCCTGGGGGCCACGGGCCTAC
ATCACCTGGACTACTCTGGCCATCTTTGTCCTGCCTGTGGCCATACTTACAGCCTGCTATA
GCCTCATTTGCCATGAGATCTGTAAGAACCTAAAAGTCAAGACACAGGCTGGGAGGGTGG
AAGCAGGGGGCTGGAGGACTTGGAACAAGCCTTCACCTTCTGCCCCAGCTGCGGCCAAG
CGGGGGCTACCATCTCGGGTCAGCAGCATCAGCACCATCTCTCGGGCCAAGATCCGAAC
GGTGAAGATGACCTTCATCATCGTGCTGGCCTACATCGCTTGCTGGGCACCCTTCTTCAGT
GTCCAGATGTGGTCTGTGTGGGATGAGGATGCCCCTGATGAAGATTCCACCAACGTGGCC
TTTACCATCTCCATGCTTTTGGGCAACATCAGCAGCTGCTGCAACCCCTGGATCTACATGG
GCTTCAATAGTCACCTGTTACCACGCCCCCTGCGTCATCTTGCCTGTTGTGGGGGAGGCC
GGCCCCGCATGCGCAGACAGCTCTCCAGCAGCAGTCTCTCCAGCCGCCGCACCACCCTG
CTGACCCGCTCCAGCTGCCCACCCACCCTCAGCCTCAGCCTCAG------------
TTCGAGGCCAGGGCCGGCAGAGTCACTGAAGGATTTAGAGCAGGCAGACGGGGAAGCCA
CCACTGAGACCAGCATCTTT--

>Cavia_porcellus

ATGGATTCTGAGCCTTCTTGGGCTGCCATCCCTAGCCCACAGGGCACCTTCTCTGCCCTCA
ATGCCACCACACCCTGGCTCGGCCGGGATGAAGAGCTGGCCAAAGTGGAAATTGGCATAC
TGGCCACTGTTCTGGTGCTGGCCTCAGGGGGCAACCTGGCCATGCTGCTGATCCTGGGC
CTGCTGGGCAGAAAGCGCTCACGCATGCACCTGTTCATGCTGCACCTGGCACTGACTGAC
CTGGGTGTGGCGCTCTTCCAGGTGCTACCTCAGCTGCTCTGGGACATCACCTACCGTTTC
CAGGGCCCTGACCTCCTCTGCCGGGCCATCAAATACTTGCAAGTGCTCAGCATGTTTGCC
TCCACCTACATGCTCCTGGCCATGACCCTGGACCGATACTTGGCCGTCTGCCACCCCCTG
CGCAACCTCCAGCAGCCCAGCCAGTCTACCTACCTGCTCATCGCTGCTCCGTGGCTGCTG
GCTGCCGTCCTCAGCCTTCCTCAGGTCTTCATTTTTCCTTGCAGGAAGTGATCCAGGGCT
CGGGGGTGCTCGACTGCTGGGCTGACTTCCGCTTTCCCTGGGGGCCACAGGCCTACATC
ACCTGGACTACCATGGCCATGTTTGTCCTGCCTGTGGCCACACTCACAGCTTTCTACAGCC
TTATCTGCCATGAGATCTGTAAAAACCTGAAAGTCAAGACAAAGGCCAGGAGAGTAGAAAG
AAGTGATTGGAGGACTTGGGACAAGCTCTTACCTTCTGCCCCAGCTGCAGCTACTCAGGG
GCTGCCATCCCATGTCAGCAGCATCAGCACCATCTCTCGGGCCAAGATCCGAACCGTGAA
GATGACTTTTGTCATTGTGCTGACCTACATCGCTTGCTGGGCACCCTTCTTTAGTGTCCAG
ATGTGGTCTGTGTGGGATGAGAATGCCCCGGATGAAGATTCCACCAACGTGGCTTTCACC
ATCACCATGCTTCTGGGCAACCTCAGCAGCTGCTGTAACCCCTGGATCTACATGGGGTTCA
ACAGCCACCTGTTGCCATGGTCCTTGCATCAGCTGGCCTGCTGCAGAAGGGCCCGGCCC
CGGCTGCACAGAGAGCTCTCCAATGGCAGTCTGTCCAGCCGCCGCACCACACTGCTGACC
CGCTCCAGCTGCCCACCCAATTTCAGCCTCAGTCTCAG------
CCTGGGCGGGAAGCCAGGGCCTGCAGGATCACTGAAGGACTTAGAACAGGAAGAGGGTG
AAGCCACCACTGAGACCAGCATGCTT--

>Octodon_degus

ATGGATTCTGAGCCTTCTTGGGCTACCATCCCTACCCCACAGGGCACCCTCTCCACCCTCA
ATGCCACCACACCCTGGCTTGGCCGAGATGAAGAGCTGGCCAAGGTGGAAATTGGCATCC
TGGCTGCTGTCCTGGTGCTGGCCTCAGCGGGCAACCTAACTGTGCTGCTGATCGTGGGCC
TGCTGGGTCGAAAGCGCTCCCGCATGCACCTGTTCATGCTGCACCTGGCACTGACTGACC
TGGGAGTGGCACTCTTCCAGGTGCTGCCCCAGCTGCTCTGGGACATCACCTACCGCTTCC
AGGGCCCCGACCTCCTCTGCCGGGCCATCAAATACCTGCAGGTGCTCAGCATGTTTGCCT
CCACCTACATGCTGCTGGCCATGACCCTGGACCGATACCTGGCCGTCTGTCACCCGCTGC
GCAGCCTCCAGCAGCCCAGCCAGTCCGCCTACCCACTCATTGCTGTTCCGTGGCTGCTAG
CCGCCGTCCTCAGCCTCCCTCAAGTCTTTATTTTTTCCTTGCGGGAGGTAATCCAGGGCTC
AGGAGTGCTGGATTGCTGGGCTGACTTCTACTTCCCCTGGGGGCCACGGGCCTACATCAC
CTGGACTACCATGGCCATCTTTGTCCTGCCTGTGGCCATGCTCACAGCCTGCTACAGCCTT
ATCTGCCATGAGATCTGTAAGAACCTAAAAGTCAAGACAAAGGCCAGGAGGGTGGAAAGA

AGTGGCTGGAGGACTTGGGACACACCCTCAGCTTCAGGCCCAGCTGCAGCCACTCAGGG
GCTGCCGGCCCAAGTCAGCAGCACCAGCACCATCTCTCGGGCCAAGATTCGTACAGTGAA
GATGACCTTTGTCATTGTGCTGGCCTACATCGCTTGCTGGGCGCCCTTCTTCAGTGTCCAG
ATGTGGTCTGTGTGGGACAGGAATGCCCCAGATGAAGATTCCACGAATGTGGCTTTCACC
ATCTCCATGCTTCTGGGCAACCTCAGCAGCTGCTGCAACCCCTGGATCTACATGGGTTTCA
ATAGCCAACTGCTGCCGCGGTCCCTGCGCAGCCTGACCTGCTGCAGAGGGGCCCGGCCC
CAGCTGCACCGACAGCTCTCCAATGGCAGCCTATCCAGCCGCCGTACCACGCTGTTCACC
CGCTCCAGCTGCCCACCCAGCCTGAGCCTCAGCCTTGG------------
CAGGAGGCTGGGGCCTGTGGAGTCACTGAAGGACTTAGAGCAGGAAGATGGCGAAGCCA
CCACTGAGACCAGCACCCTT--

>Chinchilla_lanigera

ATGGATTCTGAGCCTTCTTGGGCTGCCATGCCTACCCCACAAGGCACCCTCTCTGCCCTCA
ATGCCACCACGCCCTGGCTTGGCCGGGATGAAGAGCTGGCCAAGGTGGAAATTGGCATC
CTGGCCATTGTCCTGGTGCTGGCCTCAGGTGGCAACTTGGCCGTGCTGCTGATCCTGGGC
CTGCTGGGCCAAAAGCGCTCCCGCATGCACCTGTTCATGCTGCACCTGGCACTGACCGAC
CTGGGTGTGGCACTCTTCCAGGTGTTACCCCAGCTGCTCTGGGACATCACCTACCGGTTC
CAGGGCCCCGACCTCCTCTGCCGAGCCATCAAATACCTGCAAGTGCTCAGCATGTTTGCC
TCCACCTACATGCTGCTGGCCATGACCTTGGACCGATACCTGGCCGTCTGTCACCCCCTG
CGCAGCCTCCAGCAGCCCAGCCAGTCTACCTACCCGCTCATTGCTGCTCCTTGGCTGCTG
GCTGCCATCCTCAGCCTCCCTCAGGTCTTCATTTTTTCCTTGCGGGAGGTGATCCAGGGCT
CGGGGGTGCTGGACTGCTGGGCTGACTTCCGCTTCCCCTGGGGACCACGGGCCTACATC
ACCTGGACTACTATGGCCATCTTTGTCCTGCCTGTGGCCGTGCTCACAGCCTGCTATAGCC
TTATCTGCCATGAAATCTGTAAGAACCTAAAAGTCAAGACAAAGGCCAGGAGGGTGGAGAG
AAGTAGCTGGAGGACTTGGGACAAGCCCTTACCCTCTGCCCCAGCTGCAGCCACTCAGGG
GCTGCCATCCCGGGTCAGCAGCATCAGCACCATCTCTCGGGCCAAGATCCGTACAGTGAA
GATGACCTTCGTCATTGTGCTGGCCTACATTGCTTGCTGGGCACCCTTCTTCAGTGTCCAG
ATGTGGTCTGTGTGGGACAAGAATGCCCCAGATGAAGATTCCACCAACATGGCTTTCACCA
TTTCCATGCTTCTGGGAAACCTCAGCAGCTGCTGCAACCCCTGGATCTACATGGGTTTCAA
CAGCCACCTGCTGCCGCGGTCCCTGCGCCACCTGGCCTGCTGCAGAGGGGCCCGGCCC
CAGGTGCACAGACAGCTCTCCAATGGCAGCCTGTCCAGCCGCCACACCACACTGCTGACT
CGCTCCAGCTGCCCGCCCAGCCTCAGCCTCGGCCCAGG------------
CAGGAGACCAGGGCCTGAGGGGTTTCTGAAGGACCTAGAGCAGGAAGATGGTGAAGCCA
CTACTGAGACCAGCACCCTT--

>Marmota_marmota

ATGGATTTGGAGCCTTCCTGGACTGCCATACCCAACCCTGGGGGCACCCTTTTTGCCCCC
AATGCCACAACACCCTGGCTGGGCCGGGATGAGGAGCTGGCCAAGGTGGAGATTGGCAT
ACTGGCTACCGTCCTGGTGCTGGCCACAGGGAGCAACCTGGCTGTGCTGCTGACCCTGG
GCCAGCCAGGTCGCAAGCGCTCCCGCATGCACCTGTTTGTGCTGCACCTGGCCCTGACTG
ACCTGGGTGTGGCACTCTTCCAGGTGCTGCCCCAGCTGCTCTGGGACATCACCTACCGCT
TCCAGGGTCCTGACCTCCTCTGCCGGGCTGTCAAGTACCTGCAGGTGCTCAGCATGTTTG
CCTCCACCTACATGCTGCTGGCCATGACAGTGGACCGATACCTGGCCGTCTGTCATCCCC
TACGCAGCCTCCAGCAGCCCAGCCAGTCCACTTACCCACTCATCGCGGCTCCCTGGTTGC
TGGCTGCCATCCTTAGCCTCCCTCAAGTCTTCATATTTTCTTTGCGGGAGGTGATCCAGGG
CACAGGGGTGCTGGACTGCTGGGCTGATTTCCGCTTCTCCTGGGGGCCACGGGCCTACA
TCACCTGGACTACTCTGGCCATCTTTGTCCTGCCTGTGGCCATACTTACAGCCTGCTACAG
CCTCATCTGCCATGAGATCTGTAAGAACCTAAAAGTCAAGACACAGGCTGGGAGGGTGGA
AGCAGGGGGCTGGAGGACTTGGGACAAGCCTTCACCTTCTGCCCCAGCTGCGGCCAAGC
GGGGGCTACCATCTCGGGTCAGCAGCATCAGCACCATCTCTCGGGCCAAGATCCGAACG
GTGAAGATGACCTTCATCATCGTGCTGGCCTACATCGCTTGCTGGGCACCCTTCTTCAGTG
TCCAGATGTGGTCTGTGTGGGATGAGGATGCCCCTGATGAAGATTCCACCAACGTGGCCT
TTACCATCTCCATGCTTTTGGGCAACATCAGCAGCTGCTGCAACCCCTGGATCTACATGGG
CTTCAATAGCCACCTGTTACCACGCCCCCTGCGTCATCTTGCCTGTAGTGGGGGAGCCCG
GCCCCGCATGCGTAGACAGCTCTCCAGCAGCAGTCTCTCCAGCCGCCGCACCACCCTGCT
GACCCGCTCCAGCTGCCCACCCACCCTCAGCCTCAGCCTCAG------------
TTCGAGGCCAGGGCCGGCAGAGTCACTGAAGGATTTAGAACAGGCAGACGGGGAAGCCA
CCACTGAGACCAGCATCTTT--

>Ochotona_princeps

ATGGTTTCAGGACCTCCTTGGGGTACAAGTCCCACCCCAGAAAGTACCCTCTCTGCCCCC
AATGCCACCACACCTTGGCTGGGCCGGGATGAAGAGCTGGCCAAAGTGGAGATTGGAGT
CTTGGCCACTGTCCTGGTGCTGGCCACTGGGGGCAACCTGGCCATGCTGCTGATCCTGG
GCCGGCCTGGCTGCAAGCGCTCACGCATGCACCTGTTTGTGCTGCATTTGGCCCTGACTG
ACCTGGGTGTGGCACTCTTCCAGGTGCTGCCCCAGCTGCTCTGGGACATCACCTACCGTT
TCCAGGGCCCCGACTTCCTCTGCCGGACTGTCAAGTACCTGCAGGTGCTCAGCATGTTCG
CCTCCACCTACATGCTGCTGGCCATGACGCTGGACCGCTACCTGGCTGTCTGTCACCCCC
TACGCAGCTTCCGGCAGCCCAGCCAGTCCACCCACCCACTCATCGCTGTTCCCTGGTTGC
TCGCCGCTGTCCTCAGTCTCCCTCAAGTCTTCATTTTTTCCTTGCGAGAGGTGATCCAGGG
CTCCGGGGTGCTGGACTGCTGGGCAGACTTCCGCTTCCCCTGGGGCCTGCGGGCCTACA
TCACTTGGACTACCCTGGCCATCTTCATCCTGCCTGTGGCCATGCTCACAGCGTGCTATGG
CCTCATCTGCCATGAGATCTGTAAGAACCTAAAAGTCAAGACACAGGCCTACAGGGCAGAA
GGAAGGGACTGGAGGGCCTGGGATGGGACCCCACCCTCTGCTCCCCCTGCAGCCGCCTT
GGGGCTGCCCTCCCGCGTGAGCAGTGTCAGTACCATCTCCCGGGCCAAGATGAGGACGG
TGAAGATGACCTCCGTCATCGTGCTGGCCTACATCGCTTGCTGGGCGCCCTTCTTCAGCG
TCCAGATGTGGTCTGTGTGGGATGAAAACGCCCCTGACGAAGATTCCACCAATGTGGCCT
TCACCATCTCCATGCTCCTGGGCAACCTCAGCAGCTGCTGCAACCCCTGGATCTACATGG
GCTTCAACAGGCACCTGCTGCCGCGCACCCTGCAGCACCTCGCCTGCTGCAGACCCCCA
CGGCCCCTGCTGCGCCGGCAGCTGTCTCCCAGCAGCCTCTCTAGCCGCCGCACCACACT
GCTGACCCACACCAGCCACACTCCTGCCCTCAGCTTCAGCCTGGG------------
TAGGAGCCCAAGGCCTGGAGAGTCACCCAAGGACTCGGAGCAGCTGGATGGGGAAGCTG
CCACTGAGACGAGCATCTTC--

>Oryctolagus_cuniculus

ATGGATCCCGGGCCTCCCTGGGTTGCCAGTCCCACTCCTGGGGGCACCCTCTCTGCCCC
CAATGCCACCACACCTTGGCTGGGCCGGGATGAGGAGCTGGCCAAGGTGGAGATTGGAG
TCCTGGCCGCTGTCCTCGTGCTGGCCACAGGGGGCAACCTGGCGGTGCTGCTGATTCTG
GGCCGGCCCGGCCGCAAGCGCTCGCGCATGCACCTGTTTGTGCTGCACCTGGCCCTGAC
CGACCTGGGGGTGGCGCTCTTCCAGGTGCTGCCCCAGCTGCTCTGGGACATCACCTATC
GCTTCCAGGGCCCCGACCTCCTCTGCCGGGCCGTCAAGTACCTGCAGGTGCTTAGCATGT
TTGCCTCCACCTACATGCTGCTGGCCATGACACTGGACCGATACCTGGCCATCTGCCACC
CCCTGCGCAGCCTCCGGCAGCCCAGCCAGTCCACCCATCCACTCATCGCCGCTCCCTGG
CTGCTCGCTGCCATCCTCAGCCTCCCTCAAGTCTTCATTTTTTCTTTGCGGGAGGTGATCC
AGGGCTCGGGGGTGCTGGACTGCTGGGCGGATTTTCGCTTCCCCTGGGGTCCACGGGCT
TACATCACCTGGACCACCCTGGCCATCTTCATCTTGCCCATGGCCATGCTCACAGCCTGCT
ACAGCCTCATCTGCCACGAGATCTGTAAGAACCTGAAGGTCAAGACGCAGGCCGGGAGG
GGAGAAGGCGGGGGCTGGAGAGCTTGGGACAGGCCT---------------
GCTGCAGCTGCTCTGGGGCTGCCCTCCCGGGTGAGCAGCGTCAGCACCATCTCGCGAGC
CAAGATGCGGACAGTGAAGATGACCTTCGTCATTGTGCTGGCCTACATCGCTTGCTGGGC
ACCCTTCTTCAGTATGCAGATGTGGTCTGTGTGGGATGACGACGCCCCTGATGAAGATTCC
ACCAACGTGGCCTTCACCATCTCCATGCTTCTGGGGAACCTCAGCAGCTGCTGCAACCCC
TGGATCTACATGGGCTTCAACAGCCACCTGCTGCCGCGGCCCCTGCGTCACCTGGCCTGC
TGCAGGGCCCCGCGGCCCCGGATGCGCCGACAGCTGTCCAACAGCAGCCTCTCCAGCCG
CCGCACCACGCTGCTGACCCGCTCCAGCTGCCCGCCCACCCTCACGCTCAGCCTCAG-----
-
CCTGGGCAGGAGCCCCGGGCCCCAAGAGTCACCCAAGGACTCCAAGCAGGTGGATGGG
GAAGCTGCCACCGAGACCAGTGTCTTT--

>Capra_hircus

ATGGATTCCAGGCCTCCTTGGACGGCTGCTCCTACCTCGGGGAGCACCCTCGCTGCCGC
GAACGCCACCACACCTTGGCTGGGCCGGGATGAGGAGCTGGCCAAGGTGGAGATCGGCG
TCCTGGCCACTGTCCTGGCGCTGGCGACAGGGGGCAACCTGACGGTGCTTCTGACGGTG
GGACAGCCGGTCCGCAAGCGCTCCCGCATGCAGGTGTTCGTGCTGCATCTGGCTCTGAC
CGACCTGGGCGTGGCGCTCTTCCAGGTGCTGCCCCAGCTGCTGTGGGACATCACCTACC
GCTTCCGGGGCCCCGACCCGCTCTGCCGGGCCGTCAAGTACCTGCAGGTGCTGAGCATG
TTTGCTTCCACCTACATGCTGCTGGCCATGACGCTGGACCGCTACCTGGCCGTCTGTCAC
CCCCTGCGCAGCCTCCAGCAGCCCAGCCGGTCCACCTACCCGCTCATCGCAGCCCCCTG
GCTGCTGGCGGCGGTCCTCAGCCTCCCTCAAGTCTTCATTTTTTCCGTACGAGAGGTGATC
CAGGGCTCTGGAGTGCTGGACTGCTGGGCAGACTTCCGCTTCCCTTGGGGGCCACGGGC
CTACATCACCTGGACCACCCTAGCCATCTTCATCCTGCCAGTGGCCATGCTCACAGCCTGC

TACAGCCTCATCTGCCACGAAATCTGCAAGAACCTCAAAGTCAAGACGGAGGCTGGGCAG
GCCAAAGGAGGGAGCTGGGGCCCTGCGAACAGGCCGTCTGCTCGTGGCCCGGTGGCAG
CCACGCGGGGGCTGCCATCCCGGGTTAGCAGCGTCAGCACCATCTCGCGGGCCAAGATC
CGAACTGTGAAGATGACCTTCGTCATTGTGCTGGCCTATATCGCCTGCTGGGCGCCTTTCT
TCAGTGTCCAGATGTGGTCTGTGTGGGATGAGAATGCCCCCGATGAAGATTCGACCAACG
TGGCTTTCACCATCTCCATGCTTTTGGGCAACCTCAGCAGCTGCTGCAACCCCTGGATCTA
CATGGGCTTCAACAGCCACCTGTGGTGGCGTGCCCTGCGCCGTCTGGCCTGCTGCAGAG
GCGCTGGGCCCAGGATGCGCAGGCGGCTCTCCAATGGCAGTCTTTCCAGCCGCCATGCC
ACCCTGCTGACCCGCTCGAGTGGCCTGCCTGCCCGCGGCCTCAGCCCCGG------
ACTCAGCAGGAAACCAGGGCCCAGAGACTCCCTGAGGGGCGCAGAGCAGGTGGATGGCG
ATGCTGCCACTGAGACCAGCATCTTT--

>Capra_aegagrus

ATGGATTCCAGGCCTCCTTGGACGGCTGCTCCTACCTCGGGGAGCACCCTCGCTGCCGC
GAACGCCACCACACCCTGGCTGGGCCGGGATGAGGAGCTGGCCAAGGTGGAGATCGGC
GTCCTGGCCACTGTCCTGGCGCTGGCGACAGGGGGCAACCTGACGGTGCTTCTGACGGT
GGGACAGCCGGTCCGCAAGCGCTCCCGCATGCAGGTGTTCGTGCTGCATCTGGCTCTGA
CCGACCTGGGCGTGGCGCTCTTCCAGGTGCTGCCCCAGCTGCTGTGGGACATCACCTAC
CGCTTCCAGGGCCCGGACCCGCTCTGCCGGGCCGTCAAGTACCTGCAGGTGCTGAGCAT
GTTTGCTTCCACCTACATGCTGCTGGCCATGACGCTGGACCGCTACCTGGCCGTCTGTCC
CCCCCTGCGCAGCCTCCAGCAGCCCAGCCGGTCCACCTACCCGCTCATCGCAGCCCCCT
GGCTGCTGGCGGCGGTCCTCAGCCTCCCTCAAGTCTTCATTTTTTCCGTACGAGAGGTGA
TCCAGGGCTCTGGAGTGCTGGACTGCTGGGCAGACTTCCGCTTCCCTTGGGGGCCACGG
GCCTACATCACCTGGACCACCCTAGCCATCTTCATCCTGCCAGTGGCCATGCTCACAGCCT
GCTACAGCCTCATCTGCCACGAAATCTGCAAGAACCTCAAAGTCAAGACGGAGGCTGGGC
AGGCCGAAGGAGGGAGCTGGGGCCCTGCGAACAGGCCGTCTGCTCGTGGCCCGGTGGC
AGCCACGCGGGGGCTGCCATCCCGGGTTAGCAGCGTCAGCACCATCTCGCGGGCCAAGA
TCCGAACTGTGAAGATGACCTTCGTCATTGTGCTGGCATATATCGCCTGCTGGGCGCCTTT
CTTCAGTGTCCAGATGTGGTCTGTGTGGGATGAGAATGCCCCCGATGAAGGCTCGACCAA
CGTGGCTTTCACCATCTCCATGCTTTTGGGCAACCTCAGCAGCTGCTGCAACCCCTGGATC
TACATGGGCTTCAACAGCCACCTGTGGTGGCGTGCCCTGCGCCGTCTGGCCTGCTGCAGA
GGCGCTGGGCCCAGGATGCGCAGGCGGCTCTCCAATGGCAGTCTTTCCAGCCGCCATGC
CACCCTGCTGACCCGCTCGAGTGGCCTGCCTGCCCGCGGCCTCAGCCCCGG------
ACTCAGCAGGAAACCAGGGCCCAGAGACTCCCTGAGGGGCGCAGAGCAGGTGGATGGCG
ATGCTGCCACTGAGACCAGCATCTTT--

>Ovis_aries

ATGGATTCCAGGCCTCCGTGGACGGCTGCTCCTACCTCGGGGAGCACCGTCGCTGCTGC
GAACGCCACCACACCCTGGCTGGGCCGGGATGAGGAGCTGGCCAGGGTGGAGATCGGC
GTCCTGGCCACTGTCCTGGCGCTGGCGACAGGGGGCAACCTGACGGTGCTTCTGACGGT
GGGACAGCCGGTCCGCAAGCGCTCCCGCATGCAGGTGTTCGTGCTGCATCTGGCTCTGA
CCGACCTGGGCGTGGCGCTCTTCCAGGTGCTGCCCCAGCTGCTGTGGGACATCACCTAC
CGCTTCCGGGGCCCGGACCCGCTCTGCCGGGCCGTCAAGTACCTGCAGGTGCTGAGCAT
GTTTGCTTCCACCTACATGCTGCTGGCCATGACGCTGGACCGCTACCTGGCCGTCTGTCA
CCCCCTGCGCAGCCTCCAGCAGCCCAGCCGGTCCACCTACCCGCTCATCGCAGCCCCCT
GGCTGCTGGCGGCGGTCCTCAGCCTCCCTCAAGTCTTCATTTTTTCCCTACGAGAGGTGAT
CCAGGGCTCTGGAGTGTTGGACTGCTGGGCAGACTTCCGCTTCCCTTGGGGGCCACGGG
CCTACATCACCTGGACCACCCTAGCCATCTTCATCCTGCCAGTGGCCATGCTCACAGCCTG
CTACAGCCTCATCTGCCACGAAATCTGCAAGAACCTCAAAGTCAAGACGGAGGCTGGGCA
GGCCGAAGGAGGGAGCTGG-
GCCCTGCGAACAGGCCATCTGCTCTCGTCCCGGTGGCAGCCACGAGGGGGCTGCCGTCC
CGGGTTAGCAGCGTCAGCACCATCTCGCGGGCCAAGATCCGAACGGTGAAGATGACCTTC
GTCATTGTGCTGGCCTATATCGCCTGCTGGGCGCCTTTCTTCAGTGTCCAGATGTGGTCTG
TGTGGGATGAGAATGCCCCCGATGAAGATTCGACCAATGTGGCTTTCACCATCTCCATGCT
TTTGGGCAACCTCAGCAGCTGCTGCAACCCCTGGATCTACATGGGCTTCAACAGCCACCT
GTGGTGGCGTGCCCTGCGCCGTCTGGCCTGCTGCAGAGGCGCGGGGCCCAGGATGCGC
AGGCGGCTCTCCAACGGCAGTCTGTCCAGCCGCCATGCCACCCTGCTGACCCGCTCGAG
TGGCCTGCCTGCCCGCGGCCTCAGCCCCGG------
ACTCAGCAGGAAGCCAGGGCCCAGAGACTCCCTGCGGGGCACAGAGCAGGTGGATGGTG
ATGCTGCCACTGAGACCAGCATCTTT--

>Ovis_orientalis

ATGGATTCCAGGCCTCCTTGGACGGCTGCTCCTACCTCGGGGAGCACCGTCGCTGCTGC
GAACGCCACCACACCCTGGCTGGGCCGGGATGAGGAGCTGGCCAGGGTGGAGATCGGC
GTCCTGGCCACTGTCCTGGCGCTGGCGACAGGGGGCAACCTGACGGTGCTTCTGACGGT
GGGACAGCCGGTCCGCAAGCGCTCCCGCATGCAGGTGTTCGTGCTGCATCTGGCTCTGA
CCGACCTGGGCGTGGCGCTCTTCCAGGTGCTGCCCCAGCTGCTGTGGGACATCACCTAC
CGCTTCCGGGGCCCGGACCCGCTCTGCCGGGCCGTCAAGTACCTGCAGGTGCTGAGCAT
GTTTGCTTCCACCTACATGCTGCTGGCCATGACGCTGGACCGCTACCTGGCCGTCTGTCA
CCCCCTGCGCAGCCTCCAGCAGCCCAGCCGGTCCACCTACCCGCTCATCGCAGCCCCCT
GGCTGCTGGCGGCGGTCCTCAGCCTCCCTCAAGTCTTCATTTTTTCCCTACGAGAGGTGAT
CCAGGGCTCTGGAGTGTTGGACTGCTGGGCAGACTTCCGCTTCCCTTGGGGGCCACGGG
CCTACATCACCTGGACCACCCTAGCCATCTTCATCCTGCCAGTGGCCATGCTCACAGCCTG
CTACAGCCTCATCTGCCACGAAATCTGCAAGAACCTCAAAGTCAAGACGGAGGCTGGGCA
GGCCGAAGGAGGGAGCTGG-
GCCCTGCGAACAGGCCATCTGCTCTCGGCCCGGTGGCAGCCACGAGGGGGCTGCCGTCC
CGGGTTAGCAGCGTCAGCACCATCTCGCGGGCCAAGATCCGAACGGTGAAGATGACCTTC
GTCATTGTGCTGGCCTATATCGCCTGCTGGGCGCCTTTCTTCAGTGTCCAGATGTGGTCTG
TGTGGGATGAGAATGCCCCCGATGAAGATTCGACCAATGTGGCTTTCACCATCTCCATGCT
TTTGGGCAACCTCAGCAGCTGCTGCAACCCCTGGATCTACATGGGCTTCAACAGCCACCT
GTGGTGGCGTGCCCTGCGCCGTCTGGCCTGCTGCAGAGGCGCGGGGCCCAGGATGCGC
AGGCGGCTCTCCAACGGCAGTCTGTCCAGCCGCCATGCCACCCTGCTGACCCGCTCGAG
TGGCCTGCCTGCCCGCGGCCTCAGCCCCGG------
ACTCAGCAGGAAGCCAGGGCCCAGAGACTCCCTGCGGGGCACAGAGCAGGTGGATGGTG
ATGCTGCCACTGAGACCAGCATCTTT--

>Bos_taurus

ATGGATTCCAGGCCTCCGTGGACGGCTGCTCCTACCCCGGGGAGCACCCTCTCTGCTGC
CAACGCCACCACACCCTGGCTGGGCCGGGATGAGGAGCTGGCCAAGGTGGAGATCGGC
GTCCTGGCCACTGTCCTGGCGCTGGCGACAGGGGGCAACCTGACGGTGCTCCTGACGGT
GGGGCAGCCGGTCCGCAAGCGCTCCCGCATGCAGGTGTTCGTGCTGCACCTGGCCCTGA
CCGACCTGGGCGTGGCGCTCTTCCAGGTGCTGCCCCAGCTGCTGTGGGACATCACCTAC
CGCTTCCGGGGCCCCGACCCGCTCTGCCGGGCCGTCAAGTACCTGCAGGTGCTGAGCAT
GTTTGCCTCCACCTACATGCTGCTGGCCATGACGCTGGACCGCTACCTGGCCGTCTGTCA
CCCCCTGCGCAGCCTCCAGCAGCCCAGCCGGTCCACCTACCCGCTCATCGCAGCGCCCT
GGCTTCTGGCGGCAGTCCTCAGCCTCCCTCAAGTCTTCATTTTTTCCATACGAGAGGTGAT
CCAGGGCTCTGGAGTGCTGGACTGCTGGGCAGACTTCCGCTTCCCTTGGGGGCCACGGG
CCTACATCACCTGGACCACCCTGGCCATCTTCATCCTGCCAGTGGCCATGCTCACAGCCT
GCTATGGCCTCATCTGCCACGAGATCTGCAGGAACCTCAAAGTCAAGACGGAGGCTGGGC
AGGCTGAAGGAGGGAGCTGGGGCACTGGGAACAGGCCCTCCGCTCGTGGGCCGGTGGC
AGCCCCGCGGGGGCTGCCGTCCCGGGTTAGCAGCGTCAGCGCCATCTCACGGGCCAAGA
TCCGAACTGTGAAGATGACCTTCGTCATTGTGCTGGCCTATATCGCCTGCTGGGCGCCTTT
CTTCAGTGTCCAGATGTGGTCTGTGTGGGATGAGAATGCCCCCGATGAAGATTCGACCAAT
GTGGCTTTCACCATCTCCATGCTTTTGGGCAACCTCAGCAGCTGCTGCAACCCCTGGATCT
ACATGGGCTTCAACAGCCACCTGTGGTTGCATGCCCTGCGCCGTCTGGCCTGCTGCGGAG
GCCCTGGGCCCAGGATGCGCAGGCGGCTCTCCAACGGCAGTCTGTCCAGCCGCCATGCC
ACCCTGCTGACCCGCTCCAGTGGCCCGCCTGCCCGCGGCCTCAGCCCCGG------
ACTCAGCAGGAGGCCAGGGCCCAAAGACTCCCTGCGGGGCGCAGAGCAGGTGGAGGGC
GATGCTGCCACTGAGACCAGCATCTTT--

>Bos_primigenius

ATGGATTCCAGGCCTCCGTGGACGGCTGCTCCTAC-
CCGGGGAGCACCCTCTCTGCTGCCAACGCCACCACGCCCTGGCTGGGCCGGGATGAGGA
GCTGGCCAAGGTGGAGATCGGAAGAGCGGCCACTGTCCTGGCGCTGGCGATCGGCGG---
TCTGAGCG-
ACACCGGGCGGTGGGGCAGCCGGTCCGCAAGCGCTCCCGCATGCAGGTGTTCGTGCTGC
ACCTGGCCCTGACCGACCTGGGCGTGGCGCTCTTCCAGGTGCTGCCCCAGCTGCTGTGG
GACATCACCTACCGCTTCCGGGGCCC-GACCCGCTCTGCCGG-
CCGTCAAGTACCTGCAGGTGGTGGGCATGTCG--CTCCACCAAC---
CTGCTGGCCATGGGCTATGACCGCTACGTGGCCATCTGCCACCCCCTGCGCAGCCTCCA

GCAGCACAGCCGGTCC-
CCTACCCGCTCATCGCAGCGCCCTGGCTTCTGGCGGCAGTCCTCAGCCTCCCTCAAGTCT
TCATTTTTTCCATACCAGAGGTGATCCAGGGCTCTGGAGTGCTGGACTGCTGGGCAGACTT
CCGCTTCCCTTGGGGGCCACGGGCCTACATCACCTGGACCACCCTGGCCATCTTCATCCT
GCCAGTGGCCATGCTCACAGCCTGCTATGGCCTCATCTGCCACGAGATCTGCAGGAACCT
CAAAGTCAAGACGGAGGCTGGGCAGGCTGAAGGAGGGAGCTGGGGCACTGGGAACAGG
CCCTCCGCTCGTGGCCGGGTGGCAGGC-
CGCGGGGGCTGCCGTCCCGGGTTAGCAGCGTCAGCGCCATCTCACGGGCCAAGATCCG-
ACTGTGAAGATGACCTTCGTTATTGTGCTGGCCTATATCGCCTGCTGGGCGCCTTTCTTCA
GTGTCCAGATGTGGTCCTTGTG---------
CTTCTCCCTTGCAGATTCGACCAATGTGGCTTTCACCATCTCCATGCTTTTGGGCAACCTCA
GCAGCTGCTGCAACCCCTGGATCTACATGGGCTTCAACAGCCACCTGTGGTTGCATGCCC
TGCTCCGTCTGG-CTGCTG-
GGAGGCCCTGGGCCCAGGATGCGCAGGCGGCTCTCCAACGGCAGTC-GTCCGGC--
TCATGCCACCCTGCTGACCCGCTCCGGTCAGCCGCCTGCCCGCGACCTCAGCCCCG-------
TACGGGTCGGAGGCCAGGGCCCAAAGACTCCCTGCGGGGCGCAGAGCAGGTGGAGGGC
GATGCTGCCACTGAGACCAGCATCTTT--

>Bos_mutus

ATGGATTCCAGGCCTCCGTGGACGGCTGCTCCTACCCCGGGGAGCACCCTCTCTGCTGC
CAACGCCACCACACCCTGGCTGGGCCGGGATGAGGAGCTGGCCAAGGTGGAGATCGGC
GTCCTGGCCACTGTCCTGGCGCTGGCGACAGGGGGCAACCTGACGGTGCTCCTGACGGT
GGGGCAGCCGGTCCGCAAGCGCTCCCGCATGCAGGTGTTCGTGCTGCACCTGGCCCTGA
CCGACCTGGGCGTGGCGCTCTTCCAGGTGCTGCCCCAGCTGCTGTGGGACATCACCTAC
CGCTTCCGGGGCCCCGACCCGCTCTGCCGGGCCGTCAAGTACCTGCAGGTGCTGAGCAT
GTTTGCCTCCACCTACATGCTGCTGGCCATGACGCTGGACCGCTACCTGGCCGTCTGTCA
CCCCCTGCGCAGCCTCCAGCAGCCCAGCCGGTCCACCTACCCGCTCATCGCAGCGCCCT
GGCTTCTGGCGGCAGTCCTCAGCCTCCCTCAAGTCTTCATTTTTTCCGTACGAGAGGTGAT
CCAGGGCTCTGGAGTGCTGGACTGCTGGGCAGACTTCCGCTTCCCTTGGGGCCCACGGG
CCTACATCACCTGGACCACCCTGGCCATCTTCATCCTGCCAGTGGCCATGCTCACAGCCT
GCTACGGCCTCATCTGCCACGAGATCTGCAGGAACCTCAAAGTCAAGACGGAGGCTGGGC
AGGCCGAAGGAGGGAGCTGGGGCACTGGGAACAGGCCCTCCACTCGTGGCCCGGTGGC
AGCCCCGCGGGGGCTGCCGTCCCGGGTTAGCAGTGTCAGTGCCATCTCACGGGCCAAGA
TCCGAACTGTGAAGATGACCTTCGTCATTGTGCTGGCCTATATCGCCTGCTGGGCGCCTTT
CTTCAGTGTCCAGATGTGGTCTGTGTGGGATGAGAATGCCCCCGATGAAGATTCGACCAAT
GTGGCTTTCACCATCTCCATGCTTTTGGGCAACCTCAGCAGCTGCTGCAACCCCTGGATCT
ACATGGGCTTCAACAGCCACCTGTGGTTGCATGCCCTGCGCCGTCTGGCCTGCTGCGGAG
GCCCTGGGCCCAGGATGCGCAGGCGGCTCTCCAACGGCAGTCTGTCCAGCCGCCATGCC
ACCCTGCTGACCCGCTCCAGTGGCCCGCCTGCCCGCGGCCTCAGCCCCGG------
ACTCAGCAGGAGGCCAGGGCCCAAAGACTCCCTGCGGGGCGCAGAGCAGGTGGAGGGC
GATGCTGCCACTGAGACCAGCATCTTT--

>Bubalus_bubalis

ATGGATTCCAGGCCTCCGTGGACGGCTGCTCCTACCCCGGGGAGCACCCATTTTACCACC
AACGCCACCACACCCTGGCTGGGCCGGGATGAGGAGCTGGCCAAGGTGGAGATCGGCGT
CCTGGCCACTGTCCTGGCGCTGGCGACAGGGGGCAACCTGACGGTGCTCCTGACGGTGG
GGCAGCCGGTCCGCAAGCGCTCCCGCATGCAGGTGTTCGTGCTGCACCTGGCCCTGACC
GACCTGGGCGTGGCGCTCTTCCAGGTGCTGCCCCAGCTGCTGTGGGACATCACCTACCG
CTTCCGGGGCCCCGACCCGCTCTGCAGGGCCGTCAAGTACCTGCAGGTGCTGAGCATGT
TTGCCTCCACCTACATGCTGCTGGCCATGACGCTGGACCGCTACCTGGCCGTCTGTCACC
CGCTGCGCAGCCTCCAGCAGCCCAGCCGGTCCACCTACCCGCTCATCGCAGCGCCCTGG
CTTCTGGCGGCGGTCCTCAGCCTCCCTCAAGTCTTCATTTTTTCCATACGAGAGGTGATCC
AGGGCTCTGGAGTGCTGGACTGCTGGGCAGACTTCCGCTTCCCTTGGGGGCCACGGGCC
TACATCACCTGGACCACCCTGGCCATCTTCATCCTGCCAGTGGCCATGCTCACAGCCTGCT
ACGGCCTCATCTGCCACGAAATCTGCAAGAACCTCAAAGTCAAGACGGAGGCTGGGCAGG
CCGAAGGAGGGAGCTGGGGCACCGGGAACAGGCCCTCTGCTCATGGCCGCGTGGCAGC
CGCGCGGGGGCTGCCGTCCCGGGTTAGCAGTGTCAGCGCCATCTCACGGGCCAAGATCC
GAACTGTGAAGATGACCTTCGTCATTGTGCTGGCCTATATCGCCTGCTGGGCGCCTTTCTT
CAGTGTCCAGATGTGGTCTGTGTGGGATGAGAATGCCCCTGATGAAGATTCTACCAACGT
GGCTTTCACCATCTCCATGCTTTTGGGCAACCTCAGCAGCTGCTGCAACCCCTGGATCTAC

ATGGGCTTCAACAGCCACCTGTGGCTGCATGCCCTGCGCCATCTGGCCTGCTGCCGAGGT
GCTGGGCCCAGGATGCGCAGGCGGTTCTCCAATGGCAGTCTGTCCAGCCGCCATGCCAC
CCTGCTGACCCGCTCCAGTGGCCCGCTTGCCCGTGGCCTCAGCCCCGG------
AATCAGCAGGAGGCCAGGGCCCAAAGACTCCCTGCGGGGCGCAGAGCAGGTGGAGGGC
GATGCTGCCACTGAGACCAGCATCTTT--

>Camelus_dromdarius

ATGGATTCTGGACCTCCTTGGACCGCCAGCCCCACACGCAGGGGCACCCTCTCTGCCCCC
AATGCCACCACACCCTGGCTGGGCCGGGATGAGGAGCTGGCCAAGGTGGAGATCGGAGT
TCTGGCCACTATCCTGGTGCTGGCCACCGGGGGCAACCTGACTGTGCTGCTGACCGTGG
GACAGCCAGCCCGGAAGCGCTCCCGCATGCACCTGTTTGTGCTGCACCTCGCCCTGACTG
ACCTGGGCGTGGCGCTCTTCCAAGTGCTGCCCCAGCTGCTGTGGGACATCACCTACCGCT
TTCAGGGCCCCGACCCCCTCTGCAGGGCCATCAAGTACCTGCAGGTGCTCAGCATGTTCG
CCTCCACCTACATGCTGCTGGCCATGACCCTGGACCGCTACCTGGCCGTTTGTCACCCAC
TGCGCAGCCTCCAGCAGCCCAGCCAGTCCACCTACCCACTCATCGCTGCTCCCTGGCTGC
TGGCTGCCGTCCTCAGCCTCCCTCAAGTCTTCATTTTTTCTTTACGAGAGGTGATCCAGGG
CTCCGGAGTACTGGACTGCTGGGCAGACTTTCGCTTCCCTTGGGGACCACGGGTCTACAT
CACCTGGACCACTCTGGCCATCTTCATCCTACCTGTGGCCATGCTCACGGCCTGCTACAG
CCTCATCTGCCACGAAATCTGTAAGAATCTAAAAGTCAAGACACAGGCCCAGAGGGTAGAA
GGAGGTGGCTGGAGGACTTGGAAGAGATCCTCACCTTCTGTCCCAGCCACAGCCACGCG
GGGGCTGCCATCTCGGGTCAGCAGCGTTAGCACCATCTCACGGGCCAAGATCCGAACTGT
GAAGATGACCTTCGTCATTGTGCTGGCTTACATCGCCTGCTGGGCACCCTTCTTCAGTGTC
CAGATGTGGACTGTGTGGGATGAAAATGCTCCCGATGAAGATTCAACCGATGTGGCTTTCA
CCATCTCCATGCTTTTGGGCAACCTCAGCAGCTGCTGCAACCCCTGGATCTACATGGGCTT
CAACAGCCACGTGTGGCCGCGCCCCCTGCGCCGCCTGGCCTGCTGCGGGGGTCCCCGG
CCCCGGATGCGCCGGCAGCTCTCCAATGGCAGCCTGTCCAGCCGCCGCGCCGCGCTGCT
GACCCGTTCCAGTGGCCCGCCCGCCCTCAGCCTCAGCTCCAG------
ACTCAGCGGGAGGCCAGGGCCCGAAGAGTCACTGAAGGACTCAGAGCAGGTGGTTGGGA
ATGTCACCACTGAGATCGGCATCTTT--

>Camelus_ferus

ATGGATTCTGGGCCTCCTTGGACCGCCAGCCCCACACGCAGGGGCACCCTCTCTGCCCC
CAATGCCACCACACCCTGGCTGGGCCGGGATGAGGAGCTGGCCAAGGTGGAGATCGGAG
TCCTGGCCACTATCCTGGTGCTGGCCACCGGGGGCAACCTGACTGTGCTGCTGACCGTG
GGACAGCCAGCCCGGAAGCGCTCCCGCATGCACCTGTTTGTGCTGCACCTCGCCCTGACT
GACCTGGGCGTGGCGCTCTTCCAAGTGCTGCCCCAGCTGCTGTGGGACATCACCTACCG
CTTTCAGGGCCCCGACCCCCTCTGCAGGGCCATCAAGTACCTGCAGGTGCTCAGCATGTT
CGCCTCCACCTACATGCTGCTGGCCATGACCCTGGACCGCTACCTGGCCGTTTGTCACCC
ACTGCGCAGCCTCCAGCAGCCCAGCCAGTCCACCTACCCACTCATCGCTGCTCCCTGGCT
GCTGGCTGCCGTCCTCAGCCTCCCTCAAGTCTTCATTTTTTCTTTACGAGAGGTGATCCAG
GGCTCCGGAGTACTGGACTGCTGGGCAGACTTTCGCTTCCCTTGGGGACCACGGGTCTAC
ATCACCTGGACCACTCTGGCCATCTTCATCCTACCTGTGGCCATGCTCACGGCCTGCTACA
GCCTCATCTGCCACGAAATCTGTAAGAATCTAAAAGTCAAGACACAGGCCCAGAGGGTAGA
AGGAGGTGGCTGGAGGACTTGGAAGAGATCCTCACCTTCTGTCCCAGCCGCAGCCACGC
GGGGGCTGCCATCTCGGGTCAGCAGCGTTAGCACCATCTCACGGGCCAAGATCCGAACT
GTGAAGATGACCTTCGTCATTGTGCTGGCTTACATCGCCTGCTGGGCACCCTTCTTCAGTG
TCCAGATGTGGACTGTGTGGGATGAAAATGCTCCCGATGAAGATTCAACCGATGTGGCTTT
CACCATCTCCATGCTTTTGGGCAACCTCAGCAGCTGCTGCAACCCCTGGATCTACATGGG
CTTCAACAGCCACGTGTGGCCGCGCCCCCTGCGCCGCCTGGCCTGCTGTGGGGGTCCCC
GGCCCCAGATGCGCCGGCAGCTCTCCAATGTCAGCCTGTCCAGCCGCCGCGCCGCGCTG
CTGACCCGTTCCAGTGGCCCGCCCGCCCTCAGCCTCAGCTCCAG------
ACTCAGCGGGAGGCCAGGGCCCGAAGAGTCACTGAAGGACTCAGAGCAGGTGGTTGGGA
ATGTCACCACTGAGATCGGCATCTTT--

>Camelus_bactrianus

ATGGATTCTGGGCCTCCTTGGACCGCCAGCCCCACACGCAGGGGCACCCTCTCTGCCCC
CAATGCCACCACACCCTGGCTGGGCCGGGATGAGGAGCTGGCCAAGGTGGAGATCGGAG
TCCTGGCCACTATCCTGGTGCTGGCCACCGGGGGCAACCTGACTGTGCTGCTGACCGTG
GGACAGCCAGCCCGGAAGCGCTCCCGCATGCACCTGTTTGTGCTGCACCTCGCCCTGACT

GACCTGGGCGTGGCCCTCTTCCAAGTGCTGCCCCAGCTGCTGTGGGACATCACCTACCGC
TTTCAGGGCCCCGACCCCCTCTGCAGGGCCATCAAGTACCTGCAGGTGCTCAGCATGTTC
GCCTCCACCTACATGCTGCTGGCCATGACCCTGGACCGCTACCTGGCCGTTTGTCACCCA
CTGCGCAGCCTCCAGCAGCCCAGCCAGTCCACCTACCCACTCATCGCTGCTCCCTGGCTG
CTGGCTGCCGTCCTCAGCCTCCCTCAAGTCTTCATTTTTCTTTACGAGAGGTGATCCAGG
GCTCCGGAGTACTGGACTGCTGGGCAGACTTTCGCTTCCCTTGGGGACCACGGGTCTACA
TCACCTGGACCACTCTGGCCATCTTCATCCTACCTGTGGCCATGCTCACGGCCTGCTACAG
CCTCATCTGCCACGAAATCTGTAAGAATCTAAAAGTCAAGACACAGGCCCAGAGGGTAGAA
GGAGGTGGCTGGAGGACTTGGAAGAGATCCTCACCTTCTGTCCCAGCCGCAGCCACGCG
GGGGCTGCCATCTCGGGTCAGCAGCGTTAGCACCATCTCACGGGCCAAGATCCGAACTGT
GAAGATGACCTTCGTCATTGTGCTGGCTTACATCGCCTGCTGGGCACCCTTCTTCAGTGTC
CAGATGTGGACTGTGTGGGATGAAAATGCTCCCGATGAAGATTCAACCGATGTGGCTTTCA
CCATCTCCATGCTTTTGGGCAACCTCAGCAGCTGCTGCAACCCCTGGATCTACATGGGCTT
CAACATCCACGTGTGGCCGCGCCCCCTGCGCCGCCTGGCCTGCTGTGGGGGTCCCCGGC
CCCGGATGCGCCGGCAGCTCTCCAATGGCAGCCTGTCCAGCCGCCGCGCCGCGCTGCTG
ACCCGTTCCAGTGGCCCGCCCGCCCTCAGCCTCAGCTCCAG------
ACTCAGCGGGAGGCCAGGGCCCGAAGAGTCACTGAAGGACTCAGAGCAGGTGGTTGGGA
ATGTCACCACTGAGATCGGCATCTTT--

>Vicugna_pacos

ATGGATTCTGGGCCTCCTTGGACCGCCAGCCCCACACGCAGGGGCACCCTCTCTGCCCTC
AATGCCACCACACCCTGGCTGGGCCGGGATGAGGAGCTGGCCAAGGTGGAGATCGGAGT
CCTGGCCACTATCCTGGTGCTGGCCACCGGGGGCAACCTGACTGTGCTGCTGACCGTGG
GACAGCCAGCCCAGAAGCGCTCCCGCATGCACCTGTTTGTGCTGCACCTCGCCCTGACCG
ACCTGGGCGTGGCGCTCTTCCAAGTGCTGCCCCAGCTGCTGTGGGACATCACCTACCGCT
TTCAGGGCCCCGACCCCCTCTGCAGGGCCATCAAGTACCTGCAGGTGCTCAGCATGTTCG
CCTCCACCTACATGCTGCTGGCCATGACCCTGGACCGCTACCTGGCCGTTTGTCACCCAC
TGCGCAGCCTCCAGCAGCCCAACCAGTCCACCTACCCACTCATCGCTGCTCCCTGGCTGC
TGGCCGCCATCCTCAGCATCCCTCAAGTCTTCATTTTTCTTTACGAGAGGTGATCCAGGG
CTCCGGAGTACTGGACTGCTGGGCAGACTTTCGCTTCCCTTGGGGACCACGGGTCTACAT
CACCTGGACCACTCTGGCCATCTTCATCCTACCTGTGGCCATGCTCACGGCCTGCTACAG
CCTCATCTGCCACGAAATCTGTAAGAATCTAAAAGTCAAGACACAGGCCCAGAGGGTAGAA
GGAGGTGGCTGGAGGACTTGGAAGAGATCCTCACCTTCTGACCCAGCCGCAGCCACATG
GGGGCTGCCATCTCGGGTCAGCAGCGTTAGCACCATCTCACGGGCCAAGATCCGAACTGT
GAAGATGACCTTCATCATTGTGCTGGCTTACATCGCCTGCTGGGCACCCTTCTTCAGTGTC
CAGATGTGGACTGTGTGGGATGAAAATGCTCCTGATGAAGATTCAACCGATGTGGCTTTCA
CCATCTCCATGCTTTTGGGCAACCTTAGCAGCTGCTGCAACCCCTGGATCTACATGGGCTT
CAACAGCCACCTGTGGCCACGCCCCCTGCGCCGCCTGGCCTGCTGCGGGGGTCCCCGG
CCCCGGATGCGCCGGCAGCTCTCCAATGGCAGCCTGTCCAGCCGCCGCGCCACGCTGCT
GACCCGTTCCAGTGGCCCGCCCGCCCTCAGCCTCAGCTCCAG------
ACTCAGCGGGAGGCCTGGGCCCGTAGAGTCACTGAAGGACTCGGAGCAGGTGGTTGGGG
ATGTCACCACTGAGATTGGCATCTTT--

>Sus_scrofa_familiaris

ATGGATTCGGGACCTCCTTGGTTGGCCAACCCCACCCCTGGAGGCACTCTCTCTGTCCCC
AATGCCACCACACCCTGGCTGGGCCGGGATGAGGAACTGGCCAAGGTGGAGATTGGAGT
GTTGGCCACTGTCCTGGTGCTGGCGACAGGGGGCAACCTGACTGTGCTGCTGACCTTGG
GACAGCCAGGCCGCAAGCGCTCCCGCATGCACCTGTTTGTGCTGCACCTGGCCCTGACG
GACCTGGGCGTGGCGCTCTTCCAGGTGCTGCCCCAGCTGCTGTGGGACATCACCTACCG
CTTCCAGGGCCCTGACCCTCTCTGCCGGGCCGTTAAGTACCTACAGGTGCTCAGCATGTT
CGCCTCCACTTACATGCTGCTGGCCATGACACTGGACCGCTACCTGGCCGTTTGTCACCC
TCTGCGCAGCCTCCAGCAGCCCAACCGGTCCACCTACCCACTCATCGCAGCTCCCTGGCT
GCTGGCCGCCCTCCTCAGCCTCCCTCAAGTCTTCATTTTTCTTTGCGAGAGGTGATCCAG
GGCTCCGGCGTGCTGGACTGCTGGGCAGACTTCCGCTTCCCTTGGGGGCCACGGGCCTA
CATCACCTGGACCACCCTGGCCATCTTCATCCTGCCTGTGGCCACGCTCACGGCCTGCTA
CAGCCTCATCTGCCACGAAATCTGCAAGAACCTAAAAGTCAAGACTCAGGCCTGGAAGGT
GGAAGGAGGGGGTCGGAGGACTTGGAACGAACCCTTACCTTTGGCCCAGGCTGCCGCCA
TGCGGGGGCTGCCCTCCCGGGTCAGCAGCATCAGTACCATCTCACGGGCCAAGATCCGA
ACTGTGAAGATGACCTTTGTCATTGTGCTGGCCTACATCGCCTGCTGGGCACCTTTCTTCA
GTGTCCAGATGTGGTCTGTATGGGATGAGAATGCCCCTGACGAAGATTCAACGAATGTGG

CTTTCACCATCTCCATGCTTTTGGGCAACCTCAGCAGCTGCTGCAACCCCTGGATCTACCT
GGGCTTCAACAGCCACCTGTGGCCACGCCCCCTGCGCCATCCTGTCTGCTGCCGAGGCC
CCGGGCCCAGTGTGCGCAGGCAACTCTCCAGCAGCAGCCTGTCCAGCCGCCGCACCACC
CTGCTGACCCGCTCCAGCGGCGCCCCCAACCCCAGCCTCAGCCCCAG------
ACTCAGCGGGAGGCTGGAGCCCCAAGACTCACTGAAGGACTCGGAGCCGGTGGACAGGG
ATGGCACCACCGAGACCGGCATCTTT--

>Sus_scrofa

ATGGATTCGGGACCTCCTTGGTTGGCCAACCCCACCCCTGGAGGCACTCTCTCTGTCCCC
AATGCCACCACACCCTGGCTGGGCCGGGATGAGGAACTGGCCAAGGTGGAGATTGGAGT
GTTGGCCACTGTCCTGGTGCTGGCGACAGGGGGCAACCTGACTGTGCTGCTGACCTTGG
GACAGCCAGGCCGCAAGCGCTCCCGCATGCACCTGTTTGTGCTGCACCTGGCCCTGACG
GACCTGGGCGTGGCGCTCTTCCAGGTGCTGCCCCAGCTGCTGTGGGACATCACCTACCG
CTTCCAGGGCCCTGMCCCTCTCTGCCGGGCCGTTAAGTACCTACAGGTGCTCAGCATGTT
CGCCTCCACTTACATGCTGCTGGCCATGACACTGGACCGCTACCTGGCCGTTTGTCACCC
TCTGCGCAGCCTCCAGCAGCCCAACCGGTCCACCTACCCACTCATCGCAGCTCCCTGGCT
GCTGGCCGCCCTCCTCAGCCTCCCTCAAGTCTTCATTTTTCTTTGCGAGAGGTGATCCAG
GGCTCCGGCGTGCTGGACTGCTGGGCAGACTTCCGCTTCCCTTGGGGGCCACGGGCCTA
CATCACCTGGACCACCCTGGCCATCTTCATCCTGCCTGTGGCCACGCTCACGGCCTGCTA
CAGCCTCATCTGCCACGAAATCTGCAAGAACCTAAAAGTCAAGACTCAGGCCTGGAAGGT
GGAAGGAGGGGGTCGGAGGACTTGGAACGAACCCTTACCTTTGGCCCAGGCTGCCGCCA
TGCGGGGGCTGCCCTCCCGGGTCAGCAGCATCAGTACCATCTCACGGGCCAAGATCCGA
ACTGTGAAGATGACCTTTGTCATTGTGCTGGCCTACATCGCCTGCTGGGCACCTTTCTTCA
GTGTCCAGATGTGGTCTGTATGGGATGAGAATGCCCCTGACGAAGGCAAGTCTAATGTGG
CTTTCACCATCTCCATGCTTTTGGGCAACCTCAGCAGCTGCTGCAACCCCTGGATCTACCT
GGGCTTCAACAGCCACCTGTGGCCACGCCCCCTGCGCCATCCTGTCTGCTGCCGAGGCC
CCGGGCCCAGTGTGCGCAGGCAACTCTCCAGCAGCAGCCTGTCCAGCCGCCGCACCACC
CTGCTGACCCGCTCCAGCGGCGCCCCCAACCCCAGCCTCAGCCCCAG------
ACTCAGCGGGAGGCTGGAGCCCCAAGACTCACTGAAGGACTCGGAGCCGGTGGACAGGG
ATGGCACCACCGAGACCGGCATCTTT--

>Equus_caballus

ATGGATTCTGGGACTCCTTGGGCTGCCAACCCCACTCCCTGGGGCACCCTCTCTGCTCCC
AATGCCACAACACCCTGGCTGGGCCGGGATGAGGAGCTGGCCAAGGTGGAGATAGGAGT
CCTGGCCACTGTCCTGGTACTGGCCACAGGGGGCAACCTGACTGTGCTGCTGATGCTGG
GGCGGCTGGGCCGCAAGCTCTCGCGCATGCACCTGTTTGTGCTGCACCTGGCCCTCACT
GACCTGGGGGTGGCGCTCTTCCAGGTGCTGCCCCAGCTGCTGTGGGACATCACCTACCG
TTTCTGGGGCCCCGACCTCCTTTGCCGGGCCATCAAGTACCTGCAGGTGCTGAGCATGTT
TGCCTCCACCTACATGCTGCTGGCCATGACGCTGGACCGCTACCTGGCTGTTTGTCACCC
ACTGCGCAGCCTCCGGCAGCCCAGCCAGTCCGCCTACCCACTCATTGCCGCTCCTTGGCT
GCTAGCCGCCATCCTCAGCCTCCCTCAAGTCTTCATTTTCTCTTTGCGGGAGGTGATCCAG
GGCACCGGGGTGCTGGACTGCTGGGCAGACTTCCGCTTCCCTTGGGGGCCACGGGCCTA
CATTACCTGGACCACCCTGGCCATCTTCATCCTGCCCGTGGCCATGCTCACGACCTGCTA
CAGCCTCATCTGCCACGAGATCTGTAAGAACCTAAAAGGCAAGACGCGGGCCTGGAGGGT
AGAAAGAGGGGGCTGGAGGAATTGGGACAGGCCTGCACCATCTGCCCCGGCTGCGGCCA
CGCAGGGGCTGCCATCCCGCGTCAGCAGCATCAGCACCATTTCAAGGGCCAAGATCCGAA
CCGTGAAGATGACCTTCATCATTGTGCTGGCCTATATCGCCTGCTGGGCACCCTTCTTCAG
CGTCCAGATGTGGTCTGTGTGGGACGAGAACGCGCCTGATGAAGATTCAACCAATGTGGC
CTTCACCATCTCCATGCTTTTGGGCAACCTCAGCAGCTGCTGCAACCCCTGGATCTACATG
GGCTTCAACAGCCACCTGTGGCCGCGCCCCCTGCGCCATCTAGCCTGCTGTGGGGGCCC
CCGGCCCGGGATGCGCAGGCAGCTCTCCAACGGCAGCCTCTCCAGTCGCCGCACCACGC
TGCTGACCCGCTCCAGCGGCCCGCCCACCCTCGGCCTCAGCCCCAA------
ACTCCGTGGGAGGCCTGGGCCCCAAGAGTCACTGAAGGGCTCAGAGCCGGTGGATGGGG
AAGCCATCACTGAGACCGGCATCTTT--

>Ancient_horse

ATGGATTCTGGGACTCCTTGGGCTGACGACGCTCTTCCGATCTGCACCCTCTCTGCTCCCA
ATGCCACAACACCCTGGCTGGGCCGGGATGAGGAGCTGGCCAAGGTAGAGATAGGAGTC
CTGGCAATCGGAAGAGCACACGC-

ACAGGGGGCAACCTGACTGTGCTGCTGATGTTGGGGCGGCTGGGCCGCAAGCTCTCGCG
CATGCACCTGTTTGTGCTGCACCTGGCCCTCACTGACCTGGGGGTGGCGCTCTTCCAGGT
GCTGCCCCAGCTGCTGTGGGACATCACCTACCGTTTCTGGGGCCCCGACCTCCTTTGCCG
GGCCATCAAGTACCTGCAGGTGCTGAGCATGTTTGCCTCCACCTACATGCTGCTGGCCAT
GACGCTGGACCGCTACCTGGCTGTTTGTCACCCACTGCGCAGCCTCCGGCAGCCCAGCC
AGTCAGCCTACCCACTCATTGCTACACTCTTTCCCTACACGACGCTCTTCCGATCTTCTCAA
GTCTTCATTTTCTCTTTGCGGGAGGTGATCCAGGGCACCGGGGTGCTGGACTGCTGGGCA
GACTTCCGCTTCCCTTGGGGGCCACGGGCCTACATTACCTGGACCACCTTGGCCATCTTC
ATCCTGCCCGTGGCCATGCTCACGACCTGCAGACGTGTGCTCTTCCGATCTATCTGTAAGA
ACCTAAAAGGCAAGACGCGGGCCTGGAGGGTAGAAAGAGGGGGCTGGAGGAATTGGGAC
AGGCCTGCACATCGTGAGAGCGTCGTGGTAGCGCAGGGGCTGCCATCCCGCGTCAGCAG
CATCAGCACCATTTCAAGGGCCAAGATCCGAACCGTGAAGATGACCTTCATCATTGTGCTG
GCCTATATCGCCTGCTGGGCACCCAGATCGGACGTCCAGATGTGGTCTGTGTGGGACGAG
AACGCGCCTGATGAAGGCTCATGTGATGTGGCCTTCACCATCTCCATGCTTTTGGGCAACC
TCAGCAGCTGCTGCAACCCCTGGATCTACATGGGCCTCCG-
AGACTCATGTGTTTGCCTCCCCTACGCTCTTCCGATCTCTGTGGGGGCCCCCGGCCCGGG
ATGCGCAGGCAGCTCTCCAACGGCAGCCTAGATCGGAAGAGCACCACGT--
CTGACACGCTCTTACCGATCTCCCACCCTCGGCCTCAGCCCCAA------
ACTCCGTGGGAGGCCTGGGCCCCAAGAGTCACTGAAGGGCTCAGAGCCGGTGGATGGGG
AAGCAGATCGGAAGAGCAGCATCTTT--

>Felis_catus

ATGGATTCTGGGCCTGCCTGGGCTGCTAACCCCACTCCCGGGGGTACCTTCTCCGTCCCC
AATGCCACCACACCCTGGATGGGCCGGGATGAGGAGCTGGCCAAGGTGGAGATCGGAGT
CCTGGCCGCGGTCCTGGTGCTGGCGACAGGGGGCAATCTGACTGTGCTGCTGACCCTGG
GACAGCCAGGCCGCAAGCGCTCCCGCATGCACCTGTTTGTTCTGCACCTGGCCGTGACTG
ACCTGGGTGTGGCCCTGTTTCAGGTGCTGCCCCAGCTGCTGTGGGACATCACCTACCGTT
TCCAGGGCCCTGACCTCCTCTGCCGGGCCGTCAAGTATCTGCAGGTGCTCAGCATGTTCG
CCTCCACCTACATGCTACTGGCCATGACGCTGGACCGCTACCTGGCTGTTTGTCACCCCC
TGCGCAGCCTCCAGCAGCCCAGCCAGTCCACTTACCCTCTCATCGCCGCTCCCTGGCTGC
TGGCTGCCATCCTCAGCCTGCCTCAGATCTTCATCTTTTCTTTGCGGGAGGTGTTCCACGG
CACGGGGGTGCTGGACTGCTGGGCAGACTTCCGCTTCCCTTGGGGGCCTCGGGTCTATA
TCACCTGGACCACCCTGGCCATCTTTGTCCTGCCTGTGGTCATGCTCACGGCCTGCTACA
GCCTCATCTACCACGAGATCTGTAAGAACCTCAAAGTCAAGACGCAGGCCCGCAAGGTGG
AAGGAAGGGGCTGGAGGACTTGGGACAGGATGTCGCCTTCTGTCCCAGCTGCCGCCACA
CGGGGCCTGCCATCCCGGGTCAGCAGCATCAGTACCATCTCGAGGGCTAAGATCCGAAC
CGTGAAGATGACTTTCGTCATTGTGCTGGCCTACATTGCCTGCTGGGCACCCTTCTTCAGC
GTCCAGATGTGGTCCGTGTGGGACAAAGATGCCCCCGACGAAGATTCAACCAACGTGGCT
TTCACCATCTCCATGCTCTTGGGCAACCTCAGCAGCTGCTGCAACCCCTGGGTCTCCATG
GGTTTCAACAGCCACCTGCGGCCATGGCCGCTGTGCCACCCGGACTGCTGCGGGGGTCC
CGGGCCCCGGCCACGCCGGCAACTCTCCAGCCTCAGCCTCTCCAGCCGCCACACCACAC
TGCTGACCTGCTCCAGCGGCCTGCCCGCCCTCACCCTCAGCCCCAG------
ACTCGGTGGGGGCCCTGGGACCGAAGGGTCACTGAAGGACTCTGCGCAGGTGGATGGG
GAAGCCTCCACAGAGACCGGTGTCTTT--

>Felis_silvstris

ATGGATTCTGGGCCTGCCTGGGCTGCTAACCCCACTCCCGGGGGTACCTTCTCCGTCCCC
AATGCCACCACACCCTGGATGGGCCGGGATGAGGAGCTGGCCAAGGTGGAGATCGGAGT
CCTGGCCGCGGTCCTGGTGCTGGCGACAGGGGGCAATCTGACTGTGCTGCTGACCCTGG
GACAGCCAGGCCGCAAGCGCTCCCGCATGCACCTGTTTGTTCTGCACCTGGCCGTGACTG
ACCTGGGTGTGGCCCTGTTCCAGGTGCTGCCCCAGCTGCTCTGGGACATCACCTTCCGCT
TCCACGGGCCCGACCTGCTGTGCCGCCTGGTCAAGTACCTGCAGGTGGTGGGCATGTTC
GCCTCCACCTACATGCTACTGGCCATGACGCTGGACCGCTACCTGGCTGTTTGTCACCCC
CTGCGCAGCCTCCAGCAGCCCAGCCAGTCCACTTACCCTCTCATCGCCGCTCCCTGGCTG
CTGGCTGCCATCCTCAGCCTGCCTCAGATCTTCATCTTTTCTTTGCGGGAGGTGTTCCACG
GCACGGGGGTGCTGGACTGCTGGGCAGACTTCCGCTTCCCTTGGGGGCCTCGGGTCTAT
ATCACCTGGACCACCCTGGCCATCTTTGTCCTGCCTGTGGTCATGCTCATGGCCTGCTACA
GCCTCATCTACCACGAGATCTGTAAGAACCTCAAAGTCAAGACGCAGGCCCGCAAGGTGG
AAGGAAGGGGCTGGAGGACTTGGGACAGGATGTCGCCTTCTGTCCCAGCTGCCGCCACA
CGGGGCCTGCCATCCCGGGTCAGCAGCATCAGTACCATCTCGAGGGCTAAGATCCGAAC

CGTGAAGATGACTTTCGTCATTGTGCTGGCCTACATTGCCTGCTGGGCACCCTTCTTCAGC
GTCCAGATGTGGTCCGTGTGGGACAAAGATGCCCCCGACGAAGATTCAACCAACGTGGCT
TTCACCATCTCCATGCTCTTGGGCAACCTCAGCAGCTGCTGCAACCCCTGGGTCTCCATG
GGTTTCAACAGCCACCTGCGGCCATGGCCGCTGTGCCACCCGGACTGCTGCGGGGGTCC
CCGGCCCCGGCCACGCCGGCAACTCTCCAGCCTCAGCCTCTCCAGCCGCCACACCACAC
TGCTGACCTGCTCCAGCGGCCTGCCCGCCCTCACCCTCAGCCCCAG------
ACTCGGTGGGGGCCCTGGGACCGAAGGGTCACTGAAGGACTCTGCGCAGGTGGATGGG
GAAGCCTCCACAGAGACCGGTGTCTTT--

>Odobenus_rosmarus

ATGGATTCTGGTCCTGCCTGGGCTGCCAACCCCACTCCCCGGGGCACCTTGTCTGCCCCC
AATGCCACCACACCCTGGCTGGGCCGGGATGAGGAGCTGGCCAAGGTGGAGATCGTTGT
CCTGGCCACCGTCCTGGTGCTGGCGACAGGGGGCAATCTGACTGTGCTGCTGACCCTGG
GACAGCCAAACCGCAAGCGCTCCCGCATGCACCTGTTTGTTCTGCACCTAGCCCTGACTG
ACCTGGGTGTGGCGCTATTCCAGGTGCTGCCCCAGCTGCTGTGGGACATCACCTACCGCT
TCCAGGGCCCTGACCTCCTCTGCCGGGCCATCAAGTATCTGCAGGTGCTCAGCATGTTCG
CCTCCACCTACATGCTGCTGGCCATGACGCTGGACCGCTACCTGGCTGTTTGTCACCCCT
TGCGCAGCCTCCAGCAGCCCAGCCAGTCCACCTACCCGCTCATCGCTGCTCCCTGGCTGC
TGGCTGCCATCCTCAGCCTGCCTCAAGTCTTCATCTTTTCTTTGCGGGAGGTGATCCAGGG
CACTGGGGTGCTGGACTGCTGGGCAGACTTCCGCGTCCCTTGGGGGCCTCGGGTCTATA
TCACCTGGACCACCCTGGCCATCTTTGTCCTGCCTGTGGCCATGCTCACGGCCTGCTATA
GCCTCATCTACCATGAGATCTGTAAGAACCTAAAAATCAAGACACAGGCCCGGAAGGTGGA
AGGAAGGGGCTGCAGGACTTGGGACAGGACCTCGCCTTCTGGCCCAGCTGCAGCTACAC
GGGGGCTGCCATCCCGAGTCAGCAGCATCAGCGCCATCTCCAGGGCCAAGATCCAAACC
GTGAAAATGACTTTTGTCATCGTGCTGGCCTACGTCGCCTGCTGGGCACCCTTCTTCAGCG
TCCAGATGTGGTCTGTGTGGGACAAGAATGCCCCTGATGAAGATTCAACCAACGTGGCTTT
CACCATCTCCATGCTCCTGGGCAACCTCAGCAGCTGTTGCAACCCCTGGGTTTACATGGG
CTTCAACAGCCACCTGCGGCCGTGGCCCCTGCGCCGCCTGGCCTGCTGCAGGGGACCCC
GGCCC------
CTCCCGCAGCTCTCCGGCGGCAGCCCCTCCAGCCGCCGCACCACGCTGCTGACCCGCTC
CAGCGGCCTGCCCACCCTCACGCTCAGCCCCAG------
ACTCAGTGGGGGCCATGGGCCCGAACGCTCACTGAAGGACTCAGCGCAGGTGGACGGCG
AAGCCTCCACTGAGACCGTCGCCTTT--

>Mustela_putorius_furo

ATGGATTCTGGGCCTGCCTGGGCTGCCAACCCCACTCCCCAGGGCACCTTGTCTGCCCCC
AATGCCACCACACCCTGGCTGGGCCGGGATGAGGAGCTGGCCAAGGCGGAGATCGGAGT
CCTGGCCACCGTCCTGGTGCTGGCGACAGGGGGCAATCTGACCGTGCTGCTGACCCTGG
GACAGCGAAGCCGCAAGCGCTCCCGCATGCACCTGTTTGTTCTGCACCTGGCCCTGACCG
ACCTGGGCGTGGCGCTCTTCCAGGTGCTGCCCCAGCTGCTGTGGGACATCACCTACCGCT
TCCAGGGCCCCGACCTCCTCTGCCGGGCCGTCAAGTACCTGCAGGTGCTCAGCATGTTTG
CCTCCACCTACATGCTGCTGGCCATGACGCTGGACCGCTACCTGGCGGTTTGTCACCCAC
TGCGCAGCCTCCAGCAGCCCAGCCAGTCCGCTTACCCACTCATCGCTGCCCCCTGGCTGC
TGGCTGCCGTCTTCAGCCTGCCTCAAGTCTTCATCTTTTCTCTGCGAGAGGTGATCCAGGG
CACCGGGGTGCTGGACTGCTGGGCACACTTCCACGTCCCTTGGGGGCCTCGCGTCTACA
TCACCTGGACCACCCTGGCCATCTTTGTCCTGCCCGTGGCCATGCTCACGGCCTGCTACA
GCCTCATCTATCGTGAGATCTGTAAGAACCTCAAAGTCAAGACACAGGCCCGGAAGGTGG
AAGGAAGGGGCTGGAGGACGTGGGACAGGACCTCACCTTCTGGCCCAGCTGTGGCCACA
CGGGGGCTGCCATCCCGAGTCAGCAGCATCAACACCATCTCCAGGGCCAAGATCCAAACC
GTGAAAATGACTTTTGTCATCGTGCTGGCATACATCGCCTGCTGGGCGCCCTTCTTCAGCG
TCCAGATGTGGTCCGTGTGGGACAAGAATGCCCCTGATGAAGATTCAACCAATGTGGCCTT
CACCATCTCCATGCTCCTGGGCAACCTGAGCAGCTGTTGCAACCCCTGGGTCTACATGGG
TTTCAACAGCCACCTGCGACCGCGCCCCCTGCGCCGCCTGGCCTGCTGTGGA---
CCCCGGCCCCGGCCCTGCCCGCAGCTCTCCAGCGACAGCCCCTCGGGCCGCCACACCA
CGCTGCTGACCTGCTCCAGCGGCCTGCCCACCCTCATCCTCAGCCCCAG------
GCTCCGTGGGGGCCCCGGGTCCGAAGGCTCCCGGAAGGGCTCAGTGCAGGTGCATGGC
AAAGCCTCCACTGAGACCATCGCCTTT--

>Canis_lupus_familiaris

ATGACTTCTGGGCCTACCTGGGCTGCCAGCTCCACTCCCGGGGGCGCCTTCTCTGCCCCC
AATGCCACCACACCCTGGCTGGGCCGGGATGAGGAGCTGGCCAAGGTGGAGATCGGAGT
CCTGGCCACCGTCCTGGTGCTGGCGACAGGGGGCAATCTGACTGTGCTGCTGACCCTGG
GACAGCCAAGCCGCAAGCGCTCCCGCGTGCACCTGTTTGTTCTGCACCTAGCCCTCACTG
ACCTGGGCGTGGCACTATTCCAGGTGCTGCCCCAGCTGCTGTGGGACGTCACCTACCGCT
TCCAGGGCCCTGACCTCCTCTGCCGGGCTGTCAAGTATCTCCAGGTGCTCAGCATGTTTG
CCTCCACCTACATGCTGCTGGCCATGACGCTGGACCGCTACCTGGCTGTTTGTCACCCTC
TGCGCAGCCTCCAGCAACCCAGTCAGTCCACCTACCCGTTCATCGCCGCTCCCTGGGTGC
TGGCTGCCGTCCTCAGCCTGCCTCAAGTCTTCATCTTTTCTTTGCGGGAAGTGATCGAGGG
CACCGGGGTGCTGGACTGCTGGGCAGACTTCCGCTTCCCTTGGGGGCCTCGGGTCTACA
TCACCTGGACCACCCTGGCCATCTTTATCCTGCCCGTGGCCATGCTCACAGCCTGCTACA
GCCTCATCTACCATGAGATCTGTAAGAACCTAAAAGTCAAGACGCAGGCTCGGAAGGTGG
AAGGAAGAGGCTGGAGGGCCTGGGACAGGACCTTGCCTTCTGGCCCAGCCGCAGCCACT
CGGGGGCTGCCGTCCCGAGTCAGCAGCATCAGCACCATCTCCAGGGCCAAGATCCAAAC
TGTGAAAATGACTTTTGTCATTGTGTTGGCCTACATCGCCTGCTGGGCACCCTTCTTCAGC
GTCCAGATGTGGTCCGTGTGGGACAAGAACGCTCCTGATGAAGATTCAACCAATGTGGCT
TTCACCATCTCCATGCTCTTGGGCAACCTCAGCAGCTGTTGCAACCCTTGGATCTATATGG
GCTTCAACAGCCACCTGCGCCCGCGCCCGCTGCGCCGCCTGCCCTGCTGCTGGGG------
GCCCCAGGCGCGCCCCCAGCTCTCCAGCCACAGCCCCTCGAGCCGCCGCACCACGCTGC
TGACCCGCTCTAGCGGCCTGCCCCCCCTCACCCTCAGCCCCAG------
CCTCAGTGGGGGCCCCGGGCCTGAAGGCTCACCCAAAGACTCAGCGCAGGTGGACGCAG
AGGCCTCCACAGAGACCGTCGCCTTT--

>Canis_lupus

ATGACTTCTGGGCCTACCTGGGCTGCCAGCTCCACTCCCGGGGGCGCCTTCTCTGCCCCC
AATGCCACCACACCCTGGCTGGGCCGGGATGAGGAGCTGGCCAAGGTGGAGATCGGAGT
CCTGGCCACCGTCCTGGTGCTGGCGACAGGGGGCAATCTGACTGTGCTGCTGACCCTGG
GACAGCCAAGCCGCAAGCGCTCCCGCGTGCACCTGTTTGTTCTGCACCTAGCCCTCACTG
ACCTGGGCGTGGCACTATTCCAGGTGCTGCCCCAGCTGCTGTGGGACGTCACCTACCGCT
TCCAGGGCCCTGACCTCCTCTGCCGGGCTGTCAAGTATCTCCAGGTGCTCAGCATGTTTG
CCTCCACCTACATGCTGCTGGCCATGACGCTGGACCGCTACCTGGCTGTTTGTCACCCTC
TGCGCAGCCTCCAGCAACCCAGTCAGTCCACCTACCCGTTCATCGCCGCTCCCTGGGTGC
TGGCTGCCGTCCTCAGCCTGCCTCAAGTCTTCATCTTTTCTTTGCGGGAAGTGATCGAGGG
CACCGGGGTGCTGGACTGCTGGGCAGACTTCCGCTTCCCTTGGGGGCCTCGGGTCTACA
TCACCTGGACCACCCTGGCCATCTTTATCCTGCCCGTGGCCATGCTCACAGCCTGCTACA
GCCTCATCTACCATGAGATCTGTAAGAACCTAAAAGTCAAGACGCAGGCTCGGAAGGTGG
AAGGAAGAGGCTGGAGGGCCTGGGACAGGACCTTGCCTTCTGGCCCAGCCGCAGCCACT
CGGGGGCTGCCGTGCCGAGTCAGCAGCATCAGCACCATCTCCAGGGCCAAGATCCAAAC
TGTGAAAATGACTTTTGTCATTGTGTTGGCCTACATCGCCTGCTGGGCACCCTTCTTCAGC
GTCCAGATGTGGTCCGTGTGGGACAAGAACGCTCCTGATGAAGATTCAACCAATGTGGCT
TTCACCATCTCCATGCTCTTGGGCAACCTCAGCAGCTGTTGCAACCCTTGGATCTATATGG
GCTTCAACAGCCACCTGCGCCCGCGCCCGCTGCGCCACCTGCCCTGCTGCTGGGG------
GCCCCAGGCGCGCCCCCAGCTCTCCAGCCACAGCCCCTCGAGCCGCCGCACCACGCTGC
TGACCCGCTCTAGCGGCCTGCCCCCCCTCACCCTCAGCCCCAG------
CCTCAGTGGGGGCCCCGGGCCTGAAGGCTCACCCAAAGACTCAGCGCAGGTGGACGCAG
AGGCCTCCACAGAGACCGTCGCCTTT--

5. Alignment of the mammalian species used in the *AVPR2* analyses.

>Homo_sapiens

ATGCTCATGGCGTCCACCACTTCCGCTGTGCCTGGGCATCCCTCTCTGCCCAGCCTGCCC
AGCAACAGCAGCCAGGAGAGGCCACTGGACACCCGGGACCCGCTGCTAGCCCGGGCGG
AGCTGGCGCTGCTCTCCATAGTCTTTGTGGCTGTGGCCCTGAGCAATGGCCTGGTGCTGG
CGGCCCTAGCTCGGCGGGGCCGGCGGGGCCACTGGGCACCCATACACGTCTTCATTGGC
CACTTGTGCCTGGCCGACCTGGCCGTGGCTCTGTTCCAAGTGCTGCCCCAGCTGGCCTG
GAAGGCCACCGACCGCTTCCGTGGGCCAGATGCCCTGTGTCGGGCCGTGAAGTATCTGC
AGATGGTGGGCATGTATGCCTCCTCCTACATGATCCTGGCCATGACGCTGGACCGCCACC
GTGCCATCTGCCGTCCCATGCTGGCGTACCGCCATGGAAGTGGGGCTCACTGGAACCGG
CCGGTGCTAGTGGCTTGGGCCTTCTCGCTCCTTCTCAGCCTGCCCCAGCTCTTCATCTTCG
CCCAGCGCAACGTGGAAGGTGGCAGCGGGGTCACTGACTGCTGGGCCTGCTTTGCGGAG

CCCTGGGGCCGTCGCACCTATGTCACCTGGATTGCCCTGATGGTGTTCGTGGCACCTACC
CTGGGTATCGCCGCCTGCCAGGTGCTCATCTTCCGGGAGATTCATGCCAGTCTGGTGCCA
GGGCCATCAGAGAGGCCTGGGGGGCGCCGCAGGGGACGCCGGACAGGCAGCCCCGGT
GAGGGAGCCCACGTGTCAGCAGCTGTGGCCAAGACTGTGAGGATGACGCTAGTGATTGT
GGTCGTCTATGTGCTGTGCTGGGCACCCTTCTTCCTGGTGCAGCTGTGGGCCGCGTGGGA
CCCGGAGGCACCTCTGGAAGGGGCGCCCTTTGTGCTACTCATGTTGCTGGCCAGCCTCAA
CAGCTGCACCAACCCCTGGATCTATGCATCTTTCAGCAGCAGCGTGTCCTCAGAGCTGCG
AAGCTTGCTCTGCTGTGCCCGGGGACGCACCCCACCCAGCCTGGGTCCCCAAGATGAGT
CCTGCACCACCGCCAGCTCCTCCCTGGCCAAGGACACTTCATCG

>Pan_troglodytes

ATGCTCATGGCGTCCACCACTTCCGCTGTGCCTGGGCATCCCTCTCTGCCCAGCCTGCCC
AGCAACAGCAGCCAGGAGAGGCCACTGGACACCCGGGACCCGCTGCTAGCCCGGGCGG
AGCTGGCGCTGCTCTCCATAGTCTTTGTGGCTGTGGCCCTGAGCAATGGCCTGGTGCTGG
CGGCCCTAGCTCGGCGGGGCCGGCGGGGCCACTGGGCACCCATACACGTCTTCATTGGC
CACTTGTGCCTGGCCGACCTGGCCGTGGCTCTGTTCCAAGTGCTGCCCCAGCTGGCCTG
GAAGGCCACCGACCGCTTCCGTGGGCCAGATGCCCTGTGTCGGGCCGTGAAGTATCTGC
AGATGGTGGGCATGTATGCCTCCTCCTACATGATCCTGGCCATGACGCTGGACCGCCACC
GTGCCATCTGCCGTCCCATGCTGGCGTACCGCCATGGAAGTGGGGCTCACTGGAACCGG
CCAGTGCTAGTGGCTTGGGCCTTCTCGCTCCTTCTCAGCCTGCCCCAGCTCTTCATCTTCG
CCCAGCGCAACGTGGAAAGTGGCAGCGGGGTCACTGACTGCTGGGCCTGCTTTGCGGAG
CCCTGGGGCCGTCGCACCTATGTCACCTGGATTGCCCTGATGGTGTTCGTGGCACCTACC
CTGGGTATCGCCGCCTGCCAGGTGCTCATCTTCCGGGAGATTCATGCCAGTCTGGTGCCA
GGGCCATCAGAGAGGCCTGGGGGGCGCCGCAGGGGACGCCGGACAGGCAGCCCCGGT
GAGGGAGCCCACGTGTCAGCAGCTGTGGCCAAGACTGTGAGGATGACGCTAGTGATTGT
GGTCGTCTATGTGCTGTGCTGGGCACCCTTCTTCCTGGTGCAGCTGTGGGCCGCGTGGGA
CCCGGAGGCACCTCTGGAAGGGGCGCCCTTCGTGCTGCTCATGTTGCTGGCCAGCCTCA
ACAGCTGCACCAACCCCTGGATCTATGCATCTTTCAGCAGCAGTGTCTCCTCAGAGCTGCG
AAGCTTGCTCTGCTGTGCCCGGGGACGCACCCCACCCAGCCTGGGTCCCCAAGATGAGT
CCTGCACCACCGCCAGCTCCTCCCTGGCCAAGGACACTTCATCG

>Mus_musculus

ATGATCCTGGTGTCTACCACGTCTGCAGTGCCTGGGGCCCTTTCGTCCCCTAGCTCTCCC
AGCAACAGCAGCCAGGAGGAGCTACTGGATGACCGAGACCCGCTGTTAGTCCGGGCTGA
ACTGGCCCTGCTATCTACAATTTTTGTGGCTGTGGCCTTGAGCAATGGCCTAGTGCTTGGG
GCCCTAATACGACGGGGTCGGCGTGGACGCTGGGCACCCATGCACGTCTTCATCAGTCAT
TTGTGCCTAGCTGACCTGGCTGTGGCTCTGTTTCAAGTGCTGCCCCAGCTGGCTTGGGAT
GCCACCGACCGCTTCCATGGCCCTGATGCCTTGTGTCGGGCCGTCAAGTACCTGCAGATG
GTGGGCATGTATGCCTCTTCCTACATGATCCTGGCCATGACACTAGACCGCCATCGCGCC
ATCTGCCGCCCTATGCTGGCATACCGCCATGGAGGTGGGGCTCGCTGGAACAGGCCAGT
GCTGGTGGCCTGGGCCTTCTCACTCCTTCTCAGCCTGCCTCAGCTCTTCATCTTTGCTCAA
CGTGATGTGGGAAATGGCAGTGGGGTATTTGATTGCTGGGCCCGATTTGCAGAGCCATGG
GGCCTTCGTGCCTATGTCACCTGGATCGCCTTGATGGTGTTTGTGGCACCTGCCCTAGGC
ATTGCTGCCTGCCAGGTTCTTATCTTCCGGGAGATACATGCCAGTCTGGTGCCAGGGCCA
TCTGAAAGGGCAGGGAGGCGCCGCAGAGGACACCGGACAGGAAGTCCCAGCGAGGGAG
CCCATGTATCAGCAGCCATGGCCAAGACCGTGAGGATGACACTGGTGATTGTGATTGTCTA
CGTGCTGTGCTGGGCACCCTTCTTCCTTGTGCAGCTGTGGGCAGCGTGGGATCCAGAAGC
TCCTCTGGAAAGACCCCCCTTTGTGTTGCTCATGCTGCTGGCTAGCCTTAACAGCTGTACC
AACCCCTGGATCTATGCTTCCTTCAGTAGCAGTGTCTCCTCGGAGTTGCGTAGCCTGCTTT
GCTGTGCTCAGAGGCACACCACACACAGCCTGGGTCCTCAAGATGAGTCCTGTGCCACAG
CCAGCTCCTCTCTGATGAAGGATACACCCTCC

>Peromyscus_maniculatus

ATGCTCCTCGTGTCCACCATATCTGCTGTGCCTGGGCCTCTTTCGCCCCCTAGCTCTCCCA
GCAACAGCAGCCAGGAGGAGCTACTGGATGACCGAGACCCACTGCTAGTCCGGGCTGAA
CTGGCCCTGCTTTCTACTGTCTTTGTGGCTGTGGCCTTGAGCAATGGCTTGGTGCTGGGA
GCCCTAATACGACGGGGCCGGCGTGGACGCTGGGCACCCATGCATGTCTTCATCAGTCAT
CTGTGCCTAGCTGACCTGGCTGTGGCTCTGTTTCAAGTGCTGCCCCAGCTGGCCTGGGAT
GCCACTGACCGCTTCCATGGTCCTGATGCCCTGTGTCGGGCCGTCAAGTACCTGCAGATG

GTGGGCATGTATGCCTCCTCTTATATGATCCTGGCCATGACGCTAGACCGCCACCGCGCC
ATCTGCCGCCCTATGCTGGCATATCGCCATGGAGGTGGAGCTCGCTGGAACCGGCCAGT
GCTGGTGGCCTGGGCCTTCTCACTCCTTCTCAGCCTGCCTCAGCTCTTCATTTTTGCTCAA
CGAGATGTGGGAAACGGCAGTGGAGTGTTTGATTGCTGGGCCCGATTTGCAGAGCCCTGG
GGCCTTCGTGCCTACGTCACCTGGATTGCCTTGATGGTGTTTATAGCACCTGCCCTGGGC
ATTGCTGCCTGCCAGGTTCTTATCTTCCGAGAGATTCATGCCAGTCTGGTGCCAGGGCCAT
CTGAGAGAGCAGGGAGGCGCCGCAGAGGGCACCGGACAGGCAGTCCCATCGAGGGAGC
CCATGTGTCAGCAGCCATGGCCAAGACTGTGAGGATGACACTGGTGATTGTGATTGTCTAC
GTGCTGTGCTGGGCACCCTTCTTCCTTGTGCAGCTGTGGGCAGCGTGGGACCCAGAGGC
TCCACTGGAAAGACCTCCCTTTGTGCTGCTCATGCTGCTGGCTAGCCTTAACAGCTGTACC
AACCCCTGGATCTATGCTTCCTTCAGTAGCAGCGTCTCCTCAGAGCTACGTAGCCTGCTTT
GCTGTGCTCAGAGGCACATCACACCCAGCCTGGGACATCAAGATGAGTCCTGTGCCACGG
CCAGCTCTTCTCTGACCAAGGATACACCCTTC

>Mesocricetus_auratus

ATGCTTCTTGTGTCCACCATATCCGCTGTGCCTGGGCCTCTTTCACCTCCTAGCTCTTCCA
GCAACAGCAGCCAGGAGGAGCTACTGGATGACCGAGATCCACTGCTAGTCCGGGCTGAA
CTGGCCTTGCTTTCTACCGTCTTTGTGGCTGTGGCCTTGAGCAACGGCTTGGTGCTGGGA
GCCCTAATACGACGGAGCCGCCGTGGACGCTGGGCACCCATGCACGTCTTCATCAGTCAT
TTGTGCCTAGCTGACCTGGCTGTGGCTCTGTTTCAAGTGCTACCCCAGCTGGCTTGGGAT
GCCACTGACCGCTTCCATGGCCCTGATGCCCTGTGTCGAGCTGTCAAGTACCTGCAGATG
GTAGGCATGTATGCCTCCTCCTATATGATCCTGGCCATGACGCTAGACCGCCACCGTGCC
ATCTGCCGTCCTATGCTGGCATACCGCCATGGAGGTGGGGCTCGCTGGAACCGGCCAGT
GCTGATGGCCTGGGCCTTCTCACTCCTTCTCAGCCTGCCTCAGCTCTTCATTTTTGCTCAA
CGAGATGTGGGAAATGGCAGTGGAGTATTTGATTGCTGGGCCCGATTTGCAGAGCCCTGG
GGCCTTCGTGCCTACGTCACCTGGATTGCCTTGATGGTATTTGTAGCACCTGCCCTGGGTA
TTGCTGCCTGCCAGGTTCTTATCTTCCGAGAGATTCATGCCAGTCTAGTGCCAGGGCCATC
TGAGCGGGCAGGGAGACGCCGCAGAGGGCACCGGACAAGCAGTCCCACTGAAGGAGCC
CATGTGTCAGCAGCCATGGCCAAGACTGTGAGGATGACACTGGTGATTGTGATTGTCTAC
GTACTGTGCTGGGCACCCTTCTTCCTTGTGCAGCTGTGGGCAGCGTGGGACCCAGAGGCT
CCTCTGGAAAGGCCTCCCTTTGTGCTGCTCATGCTGCTGGCTAGTCTTAACAGCTGTACCA
ACCCCTGGATCTATGCTTCCTTCAGTAGCAGCGTCTCTTCAGAGCTGCGCAGCCTGCTTTG
CTGTGCTCAGAGGCACATCACACCCAGCCTGGGGCCTCAAGATGAGTCCTGTGCCACGGC
CAGCTCCTCTCTGACCAAGGATACTCCTTCC

>Ictiodomys_tridecemlineatus

ATGCTGCTGGCCTCCACCACCTCCGCTGTGCCTGGGCCCCTCTCTCCACTTAGCCCGCCA
AGCAACGGCAGCCGGGAGGAGTCATTGGACACCAGGGACCCATTGCTGGCCCGGGCAGA
ACTGGCCCTACTTTCCACAGTCTTTGTGGCTGTGGCCTTGAGCAATGGCTTGGTACTGGG
GGCCCTAGTACGACGTGGCCGGCGCGGCCGCTGGGCACCCATGCACGTCTTCATTGGCC
ACTTGTGCCTAGCAGACCTGGCTGTGGCTCTATTTCAAGTGCTGCCCCAGCTGGCCTGGG
ATGCCACCGACCGCTTCCGTGGGCCTGATGCCCTGTGTCGGGCCGTCAAGTACCTGCAGA
TGGTGGGCATGTATGCTTCCTCCTACATGATCCTGGCCATGACGCTAGACCGCCACCGCG
CCATCTGCCGCCCCATGCTGGCATACCGCCATGGAGGTGGGGCTCGCTGGAACCGGCCA
GTGCTGGTGGCCTGGGCCTTCTCGCTTCTTCTCAGCCTGCCTCAGCTATTCATCTTTGCCC
AGCGTGATGTGGGAGATGGCAGCGGGGTCTTCGACTGCTGGGCCCGCTTTGCAGAGCCC
TGGGGCCTTCGTGCCTATGTCACCTGGATTGCCCTGATGGTGTTTGTGGCACCTGCCCTG
GGTATTGCTGCTTGCCAGGTTCTCATCTTCCGGGAGATTCATGCCAGTCTGGTGCCAGGG
CCATCAGAAAGGGCTGGGGGGCGCCACAGAAGGCACCGGACAGGCAGTCCCAGAGAGG
GAGCCCGGGTGTCAGCAGCCATGGCCAAGACTGTGAGGATGACACTGGTGATTGTGATCG
TCTACGTGTTATGCTGGGCACCCTTCTTCCTTGTGCAGCTGTGGGCAGCATGGGACCCAG
AGGCACCTCTGGAAGGGCCCCCCTTCGTGCTGCTCATGTTGCTGGCCAGCCTCAACAGCT
GTACCAACCCCTGGATCTATGCTTCCTTCAGCAGCAGCGTCTCCTCAGAGCTGCGCAGCTT
GCTCTGCTGTGCTCGGGTACAGGCCCCACCCAGCCTGGGGCCCCAAGACGAGTCCTGTG
CCACTGCCAGCTCCTCCCTGGCCAAGGATACTTTCTCC

>Cavia_porcellus

ATGCTCTCTGCGACCACCACCTCTGTGGCACCCAGGCCCCTCTC---------
CCTGCCCAGCAACAGCAGCCAGGAACAGCCCCTAGATGCCCGGGACCCACTGCTTGCGC

GGGCCGAGCTGGCCCTGCTCTCCACCGTCTTTGTGGCTGTGGCTGTGGGCAATGGCCTG
GTTCTGGGGGCCCTGGTGAGGCAAGGCCGGCACGGACGCTGGGCACCCATGCACGTCTT
CATCGGCCACTTGTGCCTGGCTGACCTGGTGGTGGCTCTGTTTCAAGTGCTGCCCCAGCT
GGCCTGGGATGCCACCGACCGCTTCCACGGGCCTGACGCCCTGTGCCGGGCCGTCAAGT
ACCTGCAGATGGTGGGCATGTACGCCTCCTCCTACATGATCCTGGCCATGACGCTGGACC
GGCACCGTGCCATCTGCCGCCCCATGCTGGCCTACCGCCACGGGGGCGGGGCTCGCTG
GAACCGGCCAGTGCTGGTGGCCTGGGCCTTCTCGTTGCTTCTTAGCCTGCCACAGCTCTT
CATCTTTGCCCGGCGTGACGTGGGGGATGGCAGTGGGGTCTTCGACTGCTGGGCACGCT
TTGCAGAGCCCTGGGGACTCCGTGCCTATGTCACCTGGATCACACTGATGGTGTTTGTGG
CGCCAGCCCTGGGCATCGCCGCCTGCCAAGTTCTCATCTTCCGAGAGATGCATGCCAGCC
TGGTGCCTGCTCCGTCCAAGAGG------
GGGTGCCGCAGAGGGCGCCAGCCAGGCAGTGCCAGAGAAGGCGCACGCGTGTCAGCGG
CCATGGCCAAGACCGTGAGGATGACGCTGGTGATCGTCATCGTCTACGTGCTGTGCTGGG
CGCCCTTCTTCCTCGTGCAGCTGTGGGCAGCATGGGACCCCCAGGCGCCTAGGGAAAGA
CCCCCCTTCGTGCTGCTCATGCTACTTGCAAGCCTCAACAGCTGCACCAACCCCTGGATCT
ACGCGTCCTTCAGCAGCAGCGTGTCATCGGAGCTGCGCGGCCTGCTCTGCTGCGTGAGG
GGCCGCACCCCATCCGGCCTGGGGCCCCAGGATGAGTCCTGCACCACTGCCAGCTCTTA
CCTGGCCAGGGAAGCTTTCTCC

>Marmota_marmota

ATGCTGCTGGCCTCCACCACCTCCGCTGTGTCTGGGCCCCTCTCTCCACTTAGCCCGCCA
AGCAACGGCAGCCGGGAGGAGTCATTGGACACCAGGGACCCATTGCTAGCCCGGGCAGA
ACTGGCCCTACTTTCCACAGTCTTTGTGGCTGTGGCCTTGAGCAATGGCTTGGTACTGGG
GGCCCTAGTACGACGTGGCCGGCGCGGCCGCTGGGCACCCATGCACGTCTTCATTGGCC
ACTTGTGCCTAGCAGACCTGGCTGTGGCTCTATTTCAAGTGCTGCCCCAGCTAGCCTGGG
ATGCCACCGACCGCTTCCGTGGGCCTGATGCCCTGTGTCGGGCCGTCAAGTACCTGCAGA
TGGTGGGCATGTATGCTTCCTCCTACATGATCCTGGCCATGACGCTAGACCGCCACCGCG
CCATCTGCCGCCCCATGCTGGCATACCGCCATGGAGGTGGGGCTCGCTGGAACCGGCCA
GTGCTGGTGGCCTGGGCCTTCTCGCTTCTTCTCAGCCTGCCTCAGCTCTTCATCTTTGCCC
AGCGTGATGTGGGAGATGGCAGCGGGGTCTTTGACTGCTGGGCCCGCTTTGCAGAGCCC
TGGGGCCTTCGTGCCTATGTCACCTGGATTGCCCTGATGGTGTTTGTGGCACCTGCCCTG
GGTATTGCTGCTTGCCAGGTTCTCATCTTCCGGGAGATTCATGCCAGTCTGGTGCCAGGG
CCATCGGAAAGGGCTGGGGGGCGCCACAGAAGGCACCGGACAGGCAGTCCCAGAGAGG
GAGCCCGGGTGTCAGCAGCCATGGCCAAGACTGTGAGGATGACACTGGTGATTGTGATCG
TCTACGTGCTATGCTGGGCACCCTTCTTCCTTGTGCAGCTATGGGCAGCATGGGACCCAG
AGGCACCTCTGGAAGGGCCCCCCTTCGTGCTGCTCATGTTGCTGGCCAGCCTCAACAGCT
GTACCAACCCCTGGATCTATGCCTCCTTCAGCAGCAGCGTCTCCTCAGAGCTGCGCAGCT
TGCTCTGCTGTGTTCGGGTGCAGGCCCCACCCAGCCTGGGGCCCCAAGACGAGTCCTGT
GCCACTGCCAGCTCCTCCCTGGCCAAGGATACTTTCTCC

>Ochotona_princeps

ATGGTCATGGCGTCTACCACTTCAGCCTTGCCAGGACCACTCTCTCCACCCAGCCCACCC
AGCAACAGCAGCCAGAAGGAGCCGCTAGGCCCCCGTGACCCGGTGCTGGTTCGTGCAGA
ACTGGCCCTGCTCTCCACTGTCTTTGTGGCCGTGGCCCTGAGCAATGGCCTAGTGCTGGG
GGCCCTTGCCCGGCGTGGCCGGCGTGGCCGTTGGGCACCCATGCACGTCTTCATCGGCC
ACTTGTGCCTGGCCGACCTGGCCGTGGCGCTGTTCCAGGTGTTGCCACAGCTGGCCTGG
GACGCTAGCGACCGCTTCCACGGGCCTGACGCCTTGTGCCGGGCCGTCAAGTACCTGCA
GATGGTGGGCATGTACGCCTCCCCCTACATGATCCTGGCCATGACGCTGGACCGCCACCG
GGCCATCTGCCGCCCCATGCTGGCGCACCGCCAGGGAGGTGGCGCTCACTGGAACCGG
CCCGTGCTGGTGGCTTGGGCTTTCTCGCTCCTTCTCAGCCTGCCCCAGCTCTTCATCTTCG
CCCAGCGAGACGTGGGTGATGGCAGTGGGGTCTTCGACTGTTGGGCCCAGTTCGTGGAG
CCCTGGGGCCTGCGTGCTTATGTCACCTGGATCGCCTTGATGGTGTTTGTGGCACCTGCC
CTGGGTATCGCTGCCTGCCAGGTGCTCATCTTTCGGGAGATTCATGCCAGCCTGGTACCA
GGATCATCGGAGAGAGCTGGGGGACGGCGTGTAGGGCGCCGCACAGGTGACCCCCATG
AGGGAGCCCGTGTGTCAGCAGCCATGGCCAAGACCGTGCGGATGACGCTCGTGATTGTC
ATTGTCTACGTGTTGTGCTGGGCACCTTTCTTCCTGGTGCAGCTGTGGTCAGTGTGGGACA
CCGAGGCGCCTCGGGAAGGGCCCCCGTTCGTGCTGCTCATGCTGCTGGCCAGCCTCAAC
AGCTGCACCAACCCCTGGATCTATGCCTCCTTCAGCAGCAGTGTGTCCTCGGAGCTGCGC
AACTGGCTCTGCTGTGCTAGGAGGCGTGCTTCTCCCAGCCTGGGGCCCCAAGACGAGTC
CTGTGCCACGGCCAGCTCCTCACTGGCCAAGGACACTGCCTCC

>Oryctolagus_cuniculus

GTGCTCATGGCGTCCACCGCTTCAGCTGCACCCAGGCCCCTCTCTCCACCCAGCCCGCCC
GGCAACGGCAGCCAGAAGGAGCCGGTGGGCGCCCGGAACCCGGTGCTAGTCCGCGCAG
AACTGGCCCTGCTCTCCACCGTCTTCGTGGCCGTGGCCCTGAGCAACGGCCTGGTGCTG
GGGGCCCTGGCACGGCGGGGCCGGCGGGGCCGCTGGGCGCCCATGCACGTCTTCATCG
GCCACTTGTGCCTGGCTGACCTGGCCGTGGCACTGTTCCAAGTGCTGCCCCAGTTGGCCT
GGGATGCTACCGACCGCTTCCACGGGCCCGATGCCTTATGTCGGGCCGTCAAGTACCTGC
AGATGGTGGGCATGTATGCCTCCTCCTACATGATCCTGGCCATGACGCTGGACCGCCACC
GTGCCATCTGCCGCCCCATGCTGGCATACCGCCATGGAGGGGGGGCACACTGGAACCGG
CCAGTGCTGGTGGCCTGGGCCTTCTCCCTGCTCCTCAGCCTGCCCCAGCTCTTCATCTTC
GCCCAGCGCGACGTGGGAGACGGCAGCGGGGTGTTTGACTGCTGGGCCCAGTTTGTGGA
GCCCTGGGGCCTTCGTGCCTATGTTACCTGGATCGCCCTGATGGTGTTTGTGGCACCCGC
CCTGGGTATCGCCGCCTGTCAGGTGCTCATCTTCCGAGAGATTCACGCCAGTCTGGTGCC
AGGGCCATCCGAGAGGGCGGGGGGCCGCCGTGGAGGGCGCCGGACAGGTGGGCCCAG
CGAGGGAGCCCGGGTGTCGGCAGCCGTGGCCAAGACCGTGAGGATGACGCTTGTGATTG
TCATTGTCTACGTGTTGTGCTGGGCACCCTTCTTCCTTGTTCAGCTGTGGTCTGTGTGGGA
CCCGGAGGCGCCTCGGGAAGGTCCCCCCTTCGTGCTGCTCATGTTGCTGGCCAGCCTCA
ACAGCTGCACCAACCCCTGGATCTACGCCTCCTTCAGCAGCAGTGTCTCGTCGGAGCTGC
GCAGCTGGCTCTGCTGTGCTCGGAGGCACACTCTGCGCAGCCTGGGGCCCCAAGACGAG
TCCTGCGCCACCGCCAACTCCTCGCTGGCCAGGGACACGGCGTCC

>Capra_hircus

ATGTTCATGGCATCCACCACCTCAGCTGTGCCCCGGCACCTCTCTCAACCTACCCCAGCA
GGCAACGGCAGCGAAGGGGAGCTGTGGACTGCCCGGGACCCACTGCTAGCCCAGGCAG
AGCTGGCCCTGCTCTCCACCGTCTTCGTGGCTGTGGCCCTGAGCAACGGCCTGGTTTTGG
GGGCCCTGGTGCGGCGGGGCCGGCGGGGCCGCTGGGCACCCATGCACGTCTTCATCGG
CCACCTGTGCCTGGCCGACCTGGCCGTAGCTCTGTTCCAAGTGCTGCCCCAGCTGGCCTG
GGACGCCACCGACCGCTTCCGTGGGCCTGATGCCCTGTGCCGGGCAGTCAAGTACCTGC
AGATGGTGGGCATGTATGCCTCCTCCTACATGATCCTGGCCATGACACTGGACCGCCACC
GTGCCATCTGCCGCCCCATGCTGGCACACCGCCACGCGGGTGGCACTCACTGGAACCGG
CCAGTGCTGCTGGCCTGGGCCTTCTCGCTGCTCCTCAGCCTGCCCCAGCTCTTCATCTTT
GCCCAGCGTGACGTGG---
ATGGCAGCGGGGTCCTTGACTGCTGGGCCCGCTTTGCTGAGCCCTGGGGCCTCCGTGCC
TACGTCACCTGGATCGCCCTAATGGTGTTTGTGGCTCCTGCCCTGGGTATCGCTGCCTGT
CAGGTGCTCATCTTCCGGGAGATTCATGCCAGCCTGGGGCCAGGGCCGGTGCCGAGGGC
CGGCGGGCCCCGCCGAGGGTGCCGGCCGGGCGGCCCTGCCGAGGGAGCCCGGGTGTC
GGCGGCCGTGGCCAAGACCGTGAGGATGACGCTGGTGATCGTCATAGTGTACGTGCTGT
GCTGGGCGCCCTTCTTCCTTGTGCAGCTGTGGGCCGCGTGGGACCCAGAGGCACCGCGG
GAAGGGCCTCCCTTCGTGTTGCTCATGCTGCTGGCCAGCCTCAACAGCTGTACCAACCCC
TGGATCTACGCCTCCTTCAGCAGCAGCGTCTCCTCCGAGCTGCGAAGCCTGCTCTGCTGC
ACCCGGAGGCGCGCCCCGCCCAGCCCGGGGCCCCAAGAGGAGTCATGCGCCACAGCCA
GCTCCTTTCTGGCCAAGGACACCCCCTCC

>Capra_aegagrus

ATGCCCTCCGCCCCCACCTCCCCAGCTGTGCCCCGGCACCTCTCTCAACCTACCCCAGCA
GGCAACGGCAGCGAAGGGGAGCTGTGGACTGCCCGGGACCCACTGCTAGCCCAGGCAG
AGCTGGCCCTGCTCTCCACCGTCTTCGTGGCTGTGGCCCTGAGCAACGGCCTGGTTTTGG
GGGCCCTGGTGCGGCGGGGCCGGCGGGGCCGCTGGGCACCCATGCACGTCTTCATCGG
CCACCTGTGCCTGGCCGACCTGGCCGTAGCTCTGTTCCAAGTGCTGCCCCAGCTGGCCTG
GGACGCCACCGACCGCTTCCGTGGGCCTGATGCCCTGTGCCGGGCAGTCAAGTACCTGC
AGATGGTGGGCATGTATGCCTCCTCCTACATGATCCTGGCCATGACACTGGACCGCCACC
GTGCCATCTGCCGCCCCATGCTGGCACACCGCCACGCGGGTGGCACTCACTGGAACCGG
CCAGTGCTGCTGGCCTGGGCCTTCTCGCTGCTCCTCAGCCTGCCCCAGCTCTTCATCTTT
GCCCAGCGTGACGTGG---
ATGGCAGCGGGGTCCTTGACTGCTGGGCCCGCTTTGCTGAGCCCTGGGGCCTCCGTGCC
TACGTCACCTGGATCGCCCTAATGGTGTTTGTGGCTCCTGCCCTGGGTATCGCTGCCTGT
CAGGTGCTCATCTTCCGGGAGATTCATGCCAGCCTGGGGCCAGGGCCGGTGCCGAGGGC
CGGCGGGCCCCGCCGAGGGTGCCGGCCGGGCGGCCCTGCCGAGGGAGCCCGGGTGTC
GGCGGCCGTGGCCAAGACCGTGAGGATGACGCTGGTGATCGTCATAGTGTACGTGCTGT

GCTGGGCGCCCTTCTTCCTTGTGCAGCTGTGGGCCCCCTCAGCCCCAGCCCCGCCCCAC
ACAGGGCCTCCCTTCGTGTTGCTCATGCTGCTGGCCAGCCTCAACAGCTGTACCAACCCC
TGGATCTACGCCTCCTTCAGCAGCAGCGTCTCCTCCGAGCTGCGAAGCCTGCTCTGCTGC
ACCCGGAGGCGCGCCCCGCCCAGCCCGGGGCCCCAAGAGGAGTCATGCGCCACAGCCA
GCTCCTTTCTGGCCAAGGACACCCCCTCC

>Ovis_aries

ATGTTCATGGCATCCACCACCTCAGCTGTGCCCCGGCACCTCTCTCAACCTACCCCAGCA
GGCAACGGCAGCGAAGGGGAGCTGTGGACTGCCCGGGACCCGCTGCTAGCTCAGGCAG
AGCTGGCCCTGCTCTCCACCGTCTTCGTGGCCGTGGCCCTGAGCAACGGCCTGGTTTTGG
GGGCCCTGGTGCGGCGGGGCCGGCGGGGCCGCTGGGCACCCATGCACGTCTTCATCGG
CCACCTGTGCCTGGCTGACCTGGCCGTAGCTCTGTTCCAAGTGCTGCCCCAGCTGGCCTG
GGACGCCACCGACCGCTTCCGTGGGCCCGATGCCCTGTGCCGGGCAGTCAAGTACCTGC
AGATGGTGGGCATGTATGCCTCCTCCTACATGATCCTGGCCATGACGCTGGACCGCCACC
GTGCCATCTGCCACCCCATGCTGGCACACCGCCACGCGGGTGGCACTCACTGGAACCGG
CCGGTGCTGCTGGCCTGGGCCTTCTCGCTGCTCCTCAGCCTGCCCCAGCTCTTCATCTTT
GCCCAGCGTGACGTGG---
ATGGCAGCGGGGTCCTTGACTGCTGGGCCCGCTTTGCTGAGCCCTGGGGCCTCCGTGCC
TACGTCACCTGGATCGCCCTGATGGTGTTTGTGGCTCCTGCCCTGGGTATCGCTGCCTGT
CACGTGCTCATCTTCCGGGAGATTCATGCCAGCCTGGGGCCAGGGCCGGTGCCGAGGGC
CGGCGGACCCCGCCGAGAGTGCCGGCCGGGCGGTCCTGCTGA-
GGAGCCCGGGTGTCGGCGGCCGTGGCCAAGACCGTGAGGATGACGCTGGTGATCGTCAT
AGTGTACGTGCTGTGCTGGGCGCCCTTCTTCCTTGTGCAGCTGTGGGCCGCGTGGGACC
CAGAGGCACCGCGGGAAGGGCCTCCCTTCGTGTTGCTCATGCTGCTGGCCAGCCTCAAC
AGCTGTACCAACCCCTGGATCTACGCCTCCTTCAGCAGCAGCGTCTCCTCCGAGCTGCGA
AGCCTGCTCTGCTGCACCCGGAGGCGCGCCCCACCCAGCCGGGGGCCCCAAGAGGAGT
CATGCGCCACAGCCAGCTCCTTTCTGGCCAAGGACACCCCCTCC

>Ovis_orientalis

ATGTTCTGCCCCCCCCTCTGCCCAGCTGTGCCCCGGCACCTCTCTCAACCTACCCCAGCA
GGCAACGGCAGCGAAGGGGAGCTGTGGACTGCCCGGGACCCGCTGCTAGCTCAGGCAG
AGCTGGCCCTGCTCTCCACCGTCTTCGTGGCCGTGGCCCTGAGCAACGGCCTGGTTTTGG
GGGCCCTGGTGCGGCGGGGCCGGCGGGGCCGCTGGGCACCCATGCACGTCTTCATCGG
CCACCTGTGCCTGGCTGACCTGGCCGTAGCTCTGTTCCAAGTGCTGCCCCAGCTGGCCTG
GGACGCCACCGACCGCTTCCGTGGGCCCGATGCCCTGTGCCGGGCAGTCAAGTACCTGC
AGATGGTGGGCATGTATGCCTCCTCCTACATGATCCTGGCCATGACGCTGGACCGCCACC
GTGCCATCTGCCGCCCCATGCTGGCACACCGCCACGCGGGTGGCACTCACTGGAACCGG
CCGGTGCTGCTGGCCTGGGCCTTCTCGCTGCTCCTCAGCCTGCCCCAGCTCTTCATCTTT
GCCCAGCGTGACGTGG---
ATGGCAGCGGGGTCCTTGACTGCTGGGCCCGCTTTGCTGAGCCCTGGGGCCTCCGTGCC
TACGTCACCTGGATCGCCCTGATGGTGTTTGTGGCTCCTGCCCTGGGTATCGCTGCCTGT
CAGGTGCTCATCTTCCGGGAGATTCATGCCAGCCTGGGGCCAGGGCCGGTGCCGAGGGC
CGGCGGACCCCGCCGAGAGTGCCGGCCGGGCGGTCCTGCTGAGGGAGCCCGGGTGTCG
GCGGCCGTGGCCAAGACCGTGAGGATGACGCTGGTGATCGTCATAGTGTACGTGCTGTG
CTGGGCGCCCTTCTTCCTTGTGCAGCTGTGGGCCGCGTGGGACCCAGAGGCACCGCGGG
AAGGTGTGCCCTTCGTGTTGCTCATGCTGCTGGCCAGCCTCAACAGCTGTACCAACCCCT
GGATCTACGCCTCCTTCAGCAGCAGCGTCTCCTCCGAGCTGCGAAGCCTGCTCTGCTGCA
CCCGGAGGCGCGCCCCACCCAGCCGGGGGCCCCAAGAGGAGTCATGCGCCACAGCCAG
CTCCTTTCTGGCCAAGGACACCCCCTCC

>Bos_taurus

ATGTTCATGGCATCCACCACCTCAGCTGTGCCCTGGCACCTCTCTCAACCTACCCCAGCAG
GCAACGGCAGTGAAGGGGAGCTGTTGACTGCCCGGGACCCGCTGCTAGCCCAGGCAGAG
CTGGCCCTGCTCTCCACCGTCTTCGTGGCCGTGGCCCTGAGCAACGGCCTGGTTTTGGG
GGCCCTGGTGCGGCGGGGCCGGCGGGGCCGCTGGGCACCGATGCACGTCTTCATCGGC
CACCTGTGCCTGGCTGACCTGGCTGTAGCTCTGTTCCAAGTGCTGCCCCAGCTGGCCTGG
GACGCCACTGACCGCTTCCGTGGGCCCGATGCCCTGTGCCGGGCAGTCAAGTACCTGCA
GATGGTGGGCATGTATGCCTCCTCCTACATGATCCTGGCCATGACGCTGGACCGCCACCG
TGCCATCTGCCGCCCCATGCTGGCACACCGCCATGGGGGTGGCACTCATTGGAACCGGC

CGGTGCTGCTGGCCTGGGCCTTCTCGCTGCTCTTCAGCCTGCCCCAGCTCTTCATCTTTG
CCCAGCGTGACGTGG---
ATGGTAGCGGGGTCCTTGACTGCTGGGCCCGCTTTGCCGAGCCCTGGGGCCTCCGCGCC
TATGTCACCTGGATTGCCCTGATGGTGTTTGTGGCTCCTGCCCTGGGTATTGCTGCCTGTC
AGGTGCTCATCTTCCGGGAGATTCATGCCAGCCTGGGGCCGGGACCAGTGCCGAGGGCC
GGCGGGCCCCGCCGAGGGTGCCGGCCGGGCAGCCCTGCCGAGGGAGCCCGGGTGTCG
GCGGCCGTGGCCAAGACTGTGAAGATGACGCTGGTGATCGTCATAGTGTACGTGCTGTGC
TGGGCGCCCTTCTTCCTTGTGCAGCTGTGGGCCGCGTGGGACCCGGAGGCACCACGGGA
AGGGCCTCCCTTCGTGTTGCTCATGCTGCTGGCCAGCCTCAACAGCTGTACCAACCCCTG
GATCTACGCCTCCTTCAGCAGCAGCATCTCCTCCGAGCTGCGAAGCCTGCTCTGCTGCAC
TTGGAGGCGCGCCCCGCCCAGCCCGGGGCCCCAAGAAGAGTCATGCGCCACAGCCAGCT
CCTTCTTGGCCAAGGACACCCCCTCC

>Bos_primigenius

ATGTTCTCACCAGCCACCACCACAGCTGTGCCCTGGCACCTCTCTCAACCTAGAACATTCA
GTCACCGAAGCTCTGAGGAGCTGTTGACTGCCCGGGACCCGCTGTTAGCCCAGGCAGAG
CTGGCCCTGCTCTCCACCGTCTTCGTGGCCGTGCCCCTGA-CGACTGCCGGCCGC-
GGGGGCCCTGGTGCGGCGGGGCCGGCGGGGCCGCTGGGCACCGATGCACGTCTTCATC
GGCCACCTGTGCCTGGCTGACCTGGCTGTAGCTCTGTTCCAAGTGCTGCCCCAGCTGGCC
TGGGACGCCACTGACCGCCGCCGTGGTCCCGATGCCCTGTGCCGGGCAGTCAAGTACCT
GCAGGTTGTGGGCATGTATGCGTCCTCCTACATGATCCTGGCCATGACGCTGGACCGCCA
CCGCGCCATCTGCCGCCCCATGCCGGCACACCGCCACGGGGGTGGCCCTCATTGCACCC
AGCCGGTGCTGCTGGCCTGGGCCACCGAGCTGCTCTTCAGCCTGCCCCAGCTCTTCATCT
TTGCCCAGCGTGACGTGG------------
AGCTCTGTGACTGCTGGGCCCGCTTTGCCGAGCCCTGGGGCCT----------------------------------
-------------------
TATTGCTGCCTGTCAGGTGCTCATCTTCCGGGAGATTCATGCCAGCCGGATGCCAGATCC
AGTGCCGAGGGCCGGAAAGCGCCGCCGGCCCCGCCGGCCCCGCCGCCCCGCCATCGGC
GCCCGGGTGTCGGCGGCCGTGGCCACCCTGGTGAAGATGACGCTGGTGATCGTCATAGT
GTACGTGCTGTGCTGGGCGCCCTTCTTCCTTGTGGAGCTGTGGGCCGC---
GGGCCTCGATGCGCACCCGGGAGGGCCTCCCTTCGTGTTGCTCATGCTGCTGGCCAGCC
TCAACAGCTGCTCCAACCCCTGGATCTACGCCTCCTTCAGCAGCAGCATCTCCTCCGAGCT
GCGAAGCCTGCTCTGCTGCACTTGGAGGCGCGCCCCGCCCAGCCCGGGGCCCCAAGAA
GGGTCATGCGCCACAGCCAGCTCCTTCTTGGCCAAGGACACCTCCTCC

>Camelus_dromedarius

ATGCTCATGGCATCCACCGCCTCAGCTGTGCCCTGGCCCCTCTCTCAGCCTAGCCCACCC
AGCAACAGCAGCAAGAAGGAGCCGCTGGACACCCGGGACCCGCTGCTAGTCCAGGCAGA
GCTGGCCCTGCTCTCCACGGTCTTCGTGGCCGTGGCCCTGAGCAACGGCTTGGTGCTAG
GGGCTCTGGTGCGCCGGGGCCGGCGGGGCCGCTGGGCACCCATGCATGTCTTCATCGG
CCACTTGTGCCTGGCTGACCTGACCGTGGCTCTGTTCCAAGTGCTGCCCCAGCTGGCCTG
GGATGCCACCGACCGCTTCCGTGGGCCTGACGCCCTGTGCCGGGCTGTCAAGTACCTGC
AGATGGTAGGCATGTATGCCTCCTCCTACATGATCCTGGCCATGACTCTGGACCGCCACC
GCGCCATCTGCCGCCCCATGCTGGCATACCGCCATGGAGGTGGAGCTCACTGGAACCGG
CCAGTGCTGGTGGCCTGGGCCTTCTCGCTTCTTCTCAGCCTGCCCCAACTCTTCATCTTTG
CCCAGCGCGACGTGGGCGATGGCAGCGGGGTCCTCGACTGCTGGGCCCGCTTTGCCGA
GCCCTGGGGCCTCCGTGCCTACGTCACCTGGATTGCCCTAGTGGTGTTTGTGGCACCTGC
ACTGGGCATCACCGCCTGCCAGGTGCTCATCTTCCGGGAGATTCATGCCAGCCTGGTGCC
AGGGCCAGCAGAGAGGGCTGGTGGGCACCGTGGAGGGCGCCGGACTGGCAGTCCCACC
GAGGGAGCCCGGGTGTCGGCCGCCATGGCCAAGACCGTGAGGATGACCCTGGTGATCGT
GATCGTGTACGTGCTGTGCTGGGCGCCCTTCTTCCTTGTGCAGCTGTGGGCAGCGTGGGA
CCCAGAGGCACCTCAAGAAGGGCCCCCCTTCGTGCTGCTCATGCTGCTGGCCAGTCTCAA
CAGCTGTACCAATCCCTGGATCTACGCCTCCTTCAGCAGCAGCGTCTCCTCAGAGCTGCG
GAGTCTGCTCTGCTGCGCCTGCAGGCGCACCCGGCCCAGCCTGGGGCCCCAGGATGTGT
CCTGCGCCACTGCCAGCTCCTTCCTGCCCAAAGACACCTCCTCC

>Camelus_bactrianus

ATGCTCATGGCATCCACCGCCTCAGCTGTGCCCTGGCCCCTCTCTCAGCCTAGCCCACCC
AGCAACAGCAGCAAGAAGGAGCCGCTGGACACCCGGGACCCGCTGCTAGTCCAGGCAGA

GCTGGCCCTGCTCTCCACGGTCTTCGTGGCCGTGGCCCTGAGCAACGGCTTGGTGCTAG
GGGCTCTGGTGCGCCGGGGCCGGCGGGGCCGCTGGGCACCCATGCATGTCTTCATCGG
CCACTTGTGCCTGGCTGACCTGACCGTGGCTCTGTTCCAAGTGCTGCCCCAGCTGGCCTG
GGATGCCACCGACCGCTTCCGTGGGCCTGACGCCCTGTGCCGGGCTGTCAAGTACCTGC
AGATGGTAGGCATGTATGCCTCCTCCTACATGATCCTGGCCATGACTCTGGACCGCCACC
GCGCCATCTGCCGCCCCATGCTGGCATACCGGCATGGAGGTGGAGCTCACTGGAACCGG
CCAGTGCTGGTGGCCTGGGCCTTCTCGCTTCTTCTCAGCCTGCCCCAACTCTTCATCTTTG
CCCAGCGCGACGTGGGCGATGGCAGCGGGGTCCTCGACTGCTGGGCCCGCTTTGCCGA
GCCCTGGGGCCTCCGTGCCTACGTCACCTGGATTGCCCTAGTGGTGTTTGTGGCACCTGC
ACTGGGCATCACCGCCTGCCAGGTGCTCATCTTCCGGGAGATTCATGCCAGCCTGGTGCC
AGGGCCAGCAGAGAGGGCTGGTGGGCACCGTGGAGGGCGCCGGACTGGCAGTCCCACC
GAGGGAGCCCGGGTGTCGGCCGCCATGGCCAAGACCGTGAGGATGACCCTGGTGATCGT
GATCGTGTACGTGCTGTGCTGGGCGCCCTTCTTCCTTGTGCAGCTGTGGGCAGCGTGGGA
CCCAGATGCACCTCAAGAAGGGCCCCCCTTCGTGCTGCTCATGCTGCTGGCCAGTCTCAA
CAGCTGTACCAATCCCTGGATCTACGCCTCCTTCAGCAGCAGCGTCTCCTCGGAGCTGCG
GAGTCTGCTCTGCTGCGCCTGCAAGCGCACCCGGCCCAGCCTGGGGCCCCAGGATGTGT
CCTGCGCCACTGCCAGCTCCTTCCTGCCCAAAGACACCTCCTCC

>Camelus_ferus

ATGTTCTTGGCCCTCTCCCCCCCAGCTGTGCCCTGGCCCCTCTCTCAGCCTAGCCCACCC
AGCAACAGCAGCAAGAAGGAGCCGCTGGACACCCGGGACCCGCTGCTAGTCCAGGCAGA
GCTGGCCCTGCTCTCCACGGTCTTCGTGGCCGTGGCCCTGAGCAACGGCTTGGTGCTAG
GGGCTCTGGTGCGCCGGGGCCGGCGGGGCCGCTGGGCACCCATGCATGTCTTCATCGG
CCACTTGTGCCTGGCTGACCTGACCGTGGCTCTGTTCCAAGTGCTGCCCCAGCTGGCCTG
GGATGCCACCGACCGCTTCCGTGGGCCTGACGCCCTGTGCCGGGCTGTCAAGTACCTGC
AGATGGTAGGCATGTATGCCTCCTCCTACATGATCCTGGCCATGACTCTGGACCGCCACC
GCGCCATCTGCCGCCCCATGCTGGCATACCGCCATGGAGGTGGAGCTCACTGGAACCGG
CCAGTGCTGGTGGCCTGGGCCTTCTCGCTTCTTCTCAGCCTGCCCCAACTCTTCATCTTTG
CCCAGCGCGACGTGGGCGATGGCAGCGGGGTCCTCGACTGCTGGGCCCGCTTTGCCGA
GCCCTGGGGCCTCCGTGCCTACGTCACCTGGATTGCCCTAGTGGTGTTTGTGGCACCTGC
ACTGGGCATCACCGCCTGCCAGGTGCTCATCTTCCGGGAGATTCATGCCAGCCTGGTGCC
AGGGCCAGCAGAGAGGGCTGGTGGGCACCGTGGAGGGCGCCGGACTGGCAGTCCCACC
GAGGGAGCCCGGGTGTCGGCCGCCATGGCCAAGACCGTGAGGATGACCCTGGTGATCGT
GATCGTGTACGTGCTGTGCTGGGCGCCCTTCTTCCTTGTGCAGCTGTGGGCAGCGTGGGA
CCCAGAGGCACCTCAAGAAGGGCCCCCCTTCGTGCTGCTCATGCTGCTGGCCAGTCTCAA
CAGCTGTACCAATCCCTGGATCTACGCCTCCTTCAGCAGCAGCGTCTCCTCGGAGCTGCG
GAGTCTGCTCTGCTGCGCCTGCAGGCGCACCCGGCCCAGCCTGGGGCCCCAGGATGTGT
CCTGCGCCACTGCCAGCTCCTTCCTGCCCAAAGACACCTCCTCC

>Vicugna_pacos

ATGCTCATGGCATCCACCGCCTCAGCTGTGCCCTGGCCCCTCTCTCAGCCTAGCCCACCC
AGCAACAGCAGCAAGAAGGAGCCGCTGGACACCCGGGACCCGCTGCTAGTCCAGGCAGA
GCTGGCCCTGCTCTCCACGGTCTTCGTGGCCGTGGCCCTGAGCAATGGCTTGGTGCTAG
GGGCTCTGGTGCGCCGGGGCCGGCGGGGCCGCTGGGCACCCATGCATGTCTTCATCGG
CCACTTGTGCCTGGCTGACCTGACCGTGGCTCTGTTCCAAGTGCTGCCCCAGCTGGCCTG
GGATGCCACCGACCGCTTCCGTGGGCCTGACGCCCTGTGCCGGGCTGTCAAGTACCTGC
AGATGGTAGGCATGTATGCCTCCTCCTACATGATCCTGGCCATGACTCTGGACCGCCACC
GCGCCATCTGCCGCCCCATGCTGGCATACCGCCATGGAGGTGGAGCTCACTGGAACCGG
CCAGTGCTGGTGGCCTGGGCCTTCTCGCTTCTTCTCAGCCTGCCCCAACTCTTCATCTTTG
CCCAGCGCGACGTGGGTGATGGCAGCGGGGTCCTCGACTGCTGGGCCCGCTTTGCCGAG
CCCTGGGGCCTCCGTGCCTACGTCACCTGGATTGCCCTAGTGGTGTTTGTGGCACCTGCG
CTGGGCATCACCGCCTGCCAGGTGCTCATCTTCCGGGAGATTCATGCCAGCCTGGTGCCA
GGGCCAGCAGAGAGGGCTGGTGGGCACCGCGGAGGGCGCCGGACTGGCAGTCCCACCG
AGGGAGCCCGGGTGTCGGCCGCCATGGCCAAGACCGTGAGGATGACCCTGGTGATCGTG
ATCGTGTACGTGCTGTGCTGGGCGCCCTTCTTCCTTGTGCAGCTGTGGGCAGCGTGGGAC
CCAGAGGCACCTCAAGAAGGGCCCCCCTTCGTGCTGCTCATGCTGCTGGCCAGTCTCAAC
AGCTGTACCAATCCCTGGATCTACGCCTCCTTCAGCAGCAGCGTCTCCTCGGAGCTGCGG
AGTCTGCTCTGCTGTGCCTGCAGGCGCACCCCGCCCAGCCCGGGGCCCCGGGATGAGTC
CTGCGCCACTGCCAGCTCCTTCCTGCCCAAAGACACCTCCTCC

>Sus_scrofa_familiaris

GTGCCCGCTGCAGCCACCACCTCGGCTGTGCCCCGGGCCCTCTCGTGGCCCGCCGCACC
CGGCAACGGCAGCGAGAGGGAGCCGCTGGACGACCGGGACCCGCTGCTGGCCCGGGTG
GAGCTGGCCCTGCTGTCCACGGTCTTTGTGGCCGTGGCCCTGAGCAATGGCTTGGTCCTG
GGGGCCCTGGTGCGGCGGGGCCGGCGGGGCCGCTGGGCGCCCATGCACGTCTTCATCG
GCCACTTGTGTCTGGCCGACCTGGCCGTGGCTCTGTTCCAAGTGCTGCCCCAGCTGGCCT
GGGATGCCACCTACCGCTTCCGTGGGCCTGATGCCCTGTGCCGGGCGGTCAAGTACCTG
CAGATGGTAGGCATGTACGCCTCCTCCTACATGATCCTGGCCATGACGCTGGACCGCCAC
CGCGCCATCTGCCGCCCCATGCTGGCATACCGCCACGGAGGTGGAGCTCGCTGGAACCG
GCCGGTGCTGGTGGCCTGGGCCTTCTCGCTGCTTCTCAGCCTGCCCCAGCTCTTCATCTT
TGCCCAGCGCGACGTGGGAGACGGCAGCGGGGTCCTCGACTGCTGGGCCAGCTTTGCC
GAGCCCTGGGGCCTGCGTGCCTACGTCACGTGGATCGCCCTGATGGTGTTTGTGGCGCC
TGCCTTGGGCATCGCCGCCTGCCAGGTGCTCATCTTCCGGGAGATTCACACCAGCCTGGT
CCCGGGGCCGGCGGAGAGGGCTGGGGGGCACCGCGGGGGGCGCCGGGCGGGCAGCC
CCCGCGAGGGAGCCCGGGTGTCGGCGGCCATGGCTAAGACGGCGAGGATGACGCTGGT
GATCGTGGCTGTGTACGTGCTGTGCTGGGCGCCCTTCTTCCTCGTGCAGCTCTGGTCCGT
GTGGGACCCGAAGGCGCCTCGGGAAGGGCCCCCTTTTGTGCTGCTCATGCTGCTGGCCA
GCCTCAACAGCTGTACCAACCCCTGGATCTACGCCTCCTTCAGCAGCAGCATCTCCTCGG
AGCTGCGGAGCCTGCTCTGCTGCCCCCGGAGGCGCACCCCGCCCAGCCTCAGGCCCCAA
GAGGAGTCCTGCGCCACCGCCAGCTCCTTCTCGGCCAGGGACACCTCGTCC

>Sus_scrofa

ATGCCCAGAGC---
CACCACCTCGGGTGTGCCCCGGGCCCTCTCGTGGCCCGCCGCACCCGGCAACGGCAGC
GAGAGGGAGCCGCTGGACGACCGGGACCCGCTGCTGGCCCGGGTGGAGCTGGCCCTGC
TGTCCACGGTCTTTGTGGCCGTGGCCCTGAGCAATGGCTTGGTCCTGGGGGCCCTGGTG
CGGCGGGGCCGGCGGGGGCGCTGGGCGCCCATGCACGTCTTCATCGGCCACTTGTGTCT
GGCCGACCTGGCCGTGGCTCTGTTCCAAGTGCTGCCCCAGCTGGCCTGGGATGCCACCT
ACCGCTTCCGTGGGCCTGATGCCCTGTGCCGGGCGGTCAAGTACCTGCAGATGGTAGGC
ATGTACGCCTCCTCCTACATGATCCTGGCCATGACGCTGGACCGCCACCGCGCCATCTGC
CGCCCCATGCTGGCATACCGCCACGGAGGTGGAGCTCGCTGGAACCGGCCGGTGCTGGT
GGCCTGGGCCTTCTCGCTGCTTCTCAGCCTGCCCCAGCTCTTCATCTTTGCCCAGCGCGA
CGTGGGAGATGGCAGCGGGGTCCTCGACTGCTGGGCCCGCTTTGCCGAGCCCTGGGGC
CTGCGTGCCTACGTCACGTGGATCGCCCTGATGGTGTTTGTGGCGCCTGCCTTGGGCATC
GCCGCCTGCCAGGTGCTCATCTTCCGGGAGATTCACACCAGCCTGGTCCCGGGGCCGGC
GGAGAGGGCTGGGGGGCCCCGCGGGGGGCGCCGGGCGGGCAGCCCCCGCGAGGGAG
CCCGGGTGTCGGCGGCCATGGCTAAGACGGCGAGGATGACGCTGGTGATCGTGGCTGTG
TACGTGCTGTGCTGGGCGCCCTTCTTCCTCGTGCAGCTCTGGTCCGTGTGGGACCCGAAG
GCGCCTCGGGAAGGGCCCCCTTTTGTGCTGCTCATGCTGCTGGCCAGCCTCAACAGCTGT
ACCAACCCCTGGATCTACGCCTCCTTCAGCAGCAGCATCTCCTCGGAGCTGCGGAGCCTG
CTCTGCTGCCCCCGGAGGCGCACCCCGCCCAGCCTCAGGCCCCAAGAGGAGTCCTGCGC
CACCGCCAGCTCCTTCTCGGCCAGGGACACCTCGTCC

>Equus_caballus

ATGCTCAGAACGCGCACCACCTCCGCTGTGCTCCAGCCCCCCTCTCGACCCACCCCACCT
GGCAACAGCAGCGAGGAGGAGGCACTGGACACCCGGGATCCGCTGCTAGCCCAGGCAG
AGCTGGCCCTGCTCTCCACGGTCTTTGTGGCTGTGGCCCTGAGCAATGGCTTGGTGCTGG
GAGCTCTAGTGCGGCGGGGCCGGCGGGGCCACTGGGCACCCATGCACGTCTTCATCGTC
CACTTATGCTTGGCTGACCTGGCCGTGGCTCTGTTCCAAGTGCTGCCCCAGCTGGCCTGG
GATGCCACCGATCGCTTCCGTGGGCCTGATGCCCTGTGCCGGGCAGTCAAGTACCTGCA
GATGGTGGGCATGTATGCCTCCTCCTACATGATCCTGGCCATGACGCTGGACCGTCACCG
CGCCATCTGCCACCCCATGCTGGCATACCGCCACGGAGGTGGAGCTCGCTGGAACCGGC
CTGTACTGGTGGCCTGGGCCTTCTCGCTTCTTCTCAGCCTGCCCCAGCTCTTCATCTTCGC
CCAGCGTGACGTGGGAGACGGCAGCGGGGTCTTCGACTGTTGGGCCCACTTTGCCGAGC
CCTGGGGCCTCCGTGCCTATGTCACTTGGATCGCCCTAATGGTGTTTGTGGCACCAGCCC
TGGGCATCACCGCCTGCCAGGTGCTCATCTTCCGGGAGATTCATGCGAGCCTGGTGCCAG
GGCCGGCAGAGAGGGCTGGGGGGCACCGCGGAGGGCACCGGAGAGGCAGTCCCAGAG
AGGGAGCCCGGGTGTCAGCAGCCGTGGCCAAGACCGTGAGGATGACGCTGGTGATTGTG
ATTGTCTATGTGCTGTGCTGGGCGCCCTTCTTCCTCGTGCAGCTGTGGGCAGCGTGGGAC

CCAGAGGCACCTCGGGAAGGGCCCCCTTTCGTGCTGCTCATGTTGCTAGCCAGCCTCAAC
AGCTGCACCAACCCCTGGATCTACGCCTCCTTCAGTAGCAGCGTCTCCTCAGAGCTGCGC
AGCCTGCTCTGCTGGGCCCGGAGGCGTGCCCCACCCAGCCCAGGGCCCCAAGATGAGTC
CTGTGCCACTGCCAGCTCCTTCCTGGCCAAGGACACTTCCTCC

>Ancient_horse

ATGCTCAGAACGCGCACCTGCCCAGCTGTGCTCCAGCCCCCCTCTCGACCCACCCCACCT
GGCAACAGTCTTCCGATCG---
CACTGGACACCCGGGATCCGCTGCTAGCCCAGGCAGAGCTGGCCCTGCTCTCCACGGTC
TTTGTGGCTGTGGCCCTGAGCAATGGC---
GATCGGAGAGCTCTAGTGCGGCGGGGCCGGCGGGGCCACTGGGCACCCATGCACGTCTT
CATCGTCCACTTATGCTTGGCTGACCTGGCCGTGGCTCTGTTCCAAGTGCTGCCCCAGT--
GACTGGAGTTCAGACGTGTGCTTCCGATCTCCTGATGCCCTGTGCCGGGCAGTCAAGTAC
CTGCAGATGGTGGGCATGTATGCCTCCTCCTACATGAT----------
AGATCGGAAGAGCGTCGTGTTGTCTGCCACCCCATGCTGGCATACCGCCACGGAGGTGG
AGCTCGCTGGAACCGGCCTGTACTGGTGGCCTGGGCCTTCTCGCTTCTTCTCAGCCTGCC
CCAGCTCTTCATCTTCGCCCAGCGTGACGTGGGAGACGGCAGCGGGGTCTTCGACTGTTG
GGCCCACTTTGCCGAGCCCTGGGGCCTCCGTGCCTATGTCACTTGGATCGCCCTAATGGT
GTTTGTGGCACCAGCCCTGGGCATCACGGCCTGCCAGGTGCTCATCTTACGAGATATTCA
T------
CTAGTGACTGGAGTTGCAGACGTGTGCTCTTCCGATCGCGGAGGGCACCGGAGAGGCAG
TCCCAGAGAGGGAGCCCGGGTGTCAGCAGCCGTGGCCAAGACCGTGAGGATGACGCTGG
TGATTGTGATTGTCTATGTGCTGTGCTGGGCGCCCTTCTTCCTCGTGCAGCTGTGGGCAG
CGTGGGACCCCTTTTCCACTCCTACAGGGCCCCCTTTCGTGCTGCTCATGTTGCTAGCCA
GCCTCAACAGCTGCACCAACCCCTGGATCTACGCCTCCTTCAGTAGCAGCGTCTCCTCAG
AGCTGCGCAGACTGCTCTGCTGGGCCCGGAGGCGTGCCCCACCCAGCCCAGGGCCCCAA
GATGAGTCCTGTGCCACTGCCAGCTCCTTCCTGGCCAAGGACACTTCCTCC

>Felis_catus

ATGCCCTCTTCCTCCACCCCCCCAGCCGTGCCCCGGCCCCTCTCTCGACCTGTTCCACCT
GGCAACGGCAGCAAGGAGGAGCTGTCGGATACCCGGGACCCACTGCTGGCCCAGGCGG
AATTGGCCCTGCTCTCCACAGTCTTTGTGGCCGTGGCCCTGAGCAATGGCTTGGTGCTGA
GCGCCCTAGTGCGCCGGGGCCGGCGGGGTCGCTGGGCGCCCATGCACGTCTTCATTGG
CCACTTGTGCCTGGCCGACCTGGCCGTGGCTCTGTTCCAAGTACTGCCCCAGCTGGCATG
GGATGCCACCGACCGCTTCCGTGGGCCCGATGCCCTGTGCCGGGCAGTCAAGTACCTGC
AGATGGTGGGCATGTACGCCTCCTCCTACATGATCCTGGCCATGACGCTGGATCGCCACC
GCGCCATCTGCCGCCCCATGCTGGCATACCGCCACGGAGGCGGAGCCCGCTGGAACCG
GCCGGTGCTGGTGGCCTGGGCCTTCTCGCTCATTCTCAGCCTGCCCCAGCTCTTCATCTT
CGCCCAGCGTGACGTGGGAGACGGTACTGGGGTCCTTGACTGCTGGGCCCGCTTTGCTG
AGCCCTGGGGCCTCCGAGCCTATGTCACCTGGATCGCCTTAATGGTGTTTGTGGCGCCTG
CCCTGGGCATCGCTGCCTGCCAGGTGCTCATCTTCCGGGAGATTCACGCCAGCCTGGTGC
CGGGGCCGGCAGAGAGGGCCAGGGGCTGCCGTGGGGGGCGCCGGACAGGCAGTCCCA
GTGAGGGGGCCCGGGTGTCAGCAGCCATGGCCAAGACCGTGAGGATGACGCTGGTGATC
GTGATTGTGTACGTGCTGTGCTGGGCGCCCTTCTTCCTCGTGCAGCTGTGGTCGGCGTGG
GACCCTGAGGCGCCCCGGGAAGGGCCCCCCTTCGTGCTGCTGATGTTGCTGGCCAGCCT
CAACAGCTGTACCAACCCCTGGATCTATGCCGCCTTCAGCAGCAGCGTCTCCTCTGAGCT
GCGCAGCCTGCTCTGCTGGGCCCGGAAACGGGACCCCCCCAGCCCGGGGCCCCCCGAG
GAGTCCTGCGCCACCGGCAGCTCCTTCCTGGCCAAGGACACTTCCTCC

>Felis_silvestris

ATG-----
GGCATCCAGCACGTCTGCCGTGCCCCGGCCCCTCTCTCGACCTGTTCCACCTGGCAACG
GCAGCAAGGAGGAGCTGTCGGATACCCGGGACCCACTGCTGGCCCAGGCGGAATTGGCC
CTGCTCTCCACAGTCTTTGTGGCCGTGGCCCTGAGCAATGGCTTGGTGCTGAGCGCCCTA
GTGCGCCGGGGCCGGCGGGGTCGCTGGGCGCCCATGCACGTCTTCATTGGCCACTTGTG
CCTGGCCGACCTGGCCGTGGCTCTGTTCCAAGTACTGCCCCAGCTGGCATGGGATGCCA
CCGACCGCTTCCGTGGGCCCGATGCCCTGTGCCGGGCAGTCAAGTACCTGCAGATGGTG
GGCATGTACGCCTCCTCCTACATGATCCTGGCCATGACGCTGGATCGCCACCGCGCCATC
TGCCGCCCCATGCTGGCATACCGCCACGGAGGCGGAGCCCGCTGGAACCGGCCGGTGC

TGGTGGCCTGGGCCTTCTCGCTCATTCTCAGCCTGCCCCAGCTCTTCATCTTCGCCCAGC
GTGACGTGGGAGACGGTACTGGGGTCCTTGACTGCTGGGCCCGCTTTGCTGAGCCCTGG
GGCCTCCGAGCCTATGTCACCTGGATCGCCTTAATGGTGTTTGTGGCGCCTGCCCTGGGC
ATCGCTGCCTGCCAGGTGCTCATCTTCCGGGAGATTCACGCCAGCCTGGTGCCGGGGCC
GGCAGAGAGGGCCAGGGGCTGCCGTGGGGGGCGCCGGACAGGCAGGCCCAGTGAGGG
GGCCCGGGTGTCAGCAGCCATGGCCAAGACCGTGAGGATGACGCTGGTGATCGTGATTG
TGTACGTGCTGTGCTGGGCGCCCTTCTTCCTCGTGCAGCTGGGGTCGGCGCGGGACCCT
TCGGCACCCCCA-
CAGGGCCCCCCTTCGTGCTGCTGATGTTGCTGGCCAGCCTCAACAGCTGTACCAACCCCT
GGATCTATGCCGCCTTCAGCAGCAGCGTCTCCTCTGAGCTGCGCAGCCTGCTCTGCTGGG
CCCGGAAACGGCCCCCCCCCGCCCGGGGCCCCCCGAGGAGGCGTGCGCCACTGGCAG
CTCCTTCCTGGCCAAGGACACTTCCTCC

>Ailuropoda_melanoleuca

ACGCTCATGGCATCCACCACCTCTGCTGTGCCCTGGACCCTCTCTCCACCTATCCCACCTG
GCAACAGCAGC---
AAGGAGCAGCTGGACGCCCGGGACCCGCGGCTCGTCCAGGCGGAGCTGGCCCTGCTCT
CCACGGTCTTTGTGGCTGTGGCCCTGAGCAATGGCTTGGTGCTGGGGGTCCTAGCACGC
CGGGGCCGGCGGGGTCGCTGGGCACCCATGCATGTCTTCATTGGCCACTTGTGCCTGGC
CGACCTGGCCGTGGCTCTGTTCCAAGTACTGCCCCAGCTGGCCTGGGATGCCACGGACC
GCTTCCGTGGGCCTGATGCCCTGTGCCGGGCAGTCAAGTACCTGCAGATGGTGGGCATG
TATGCCTCCTCCTACATGATCCTGGCCATGACACTGGACCGCCACCGCGCCATCTGCCGC
CCCATGCTGGCATACCGCCACGGAGGTGGAGCTCACTGGAACCGGCCGGTGCTGGTGGC
CTGGGCCTTCTCGCTCATTCTCAGCCTGCCCCAGCTTTTCATCTTTGCCCAGCGTGACGTG
GGAAATGGCAGTGGGGTCCTCGACTGCTGGGCCCACTTTGCTGAGCCCTGGGGCCTCAG
AGCCTATGTCACCTGGATCGCCCTAATGGTGTTTGTGGCACCTGCCCTGGGCATCGCTGC
CTGCCAGGTGCTCATCTTCCGAGAGCTTCATGCCAGCCTGGCGCCAGGGCCAGCAGACA
GGGCTGGGGGTTGCCGTGGAGGGCACCGGACAGGCAGTCCCAGTGAGGGGGCCCGGGT
GTCAGCAGCCATGGCCAAGACCGTGAGGATGACACTGGTGATCGTGATTGTCTACGTGTT
GTGCTGGGCACCTTTCTTCCTCGTGCAGCTGTGGGCAGCGTGGGACCCGCAGGCGCCCC
TGGAAGAGGCCCCGTTCGTGCTGCTGATGCTGCTGGCCAGCCTCAACAGCTGCACCAACC
CCTGGATCTACGCCTTCTTCAGCAGCAGCGTCTCCTCCGAGCTGCGCAGCCTGCTCTGCG
GGGCCCACGGTCGGGCCCCACCCAGCCTGGGGCCCCAAGACGAGTCCTGTGCCACCGC
CAGCTCCTTCCTGGCCAAGGACACTTCCTCC

>Mustela_putorius_furo

ATGCCCACGGCGTCCACCGCCTCTGCTGCGCCCTGGACCCTCTCTGCCCCTGTGCCACCT
GGCAACGGCAGC---
GAGGGGCTGCTGGACACGCGGAACCTGCTGCTCGCCCAGGCGGAGCTTGCCCTGCTCTC
CACAGTCTTTGTGGCTGTGGCCCTGAGCAACGGCTTGGTGCTGGGGGTCCTAGCACGCC
GGGGCCGGCGGGGTCGCTGGGCACCCATGCATGTCTTCATTGGCCACTTGTGCCTGGCC
GACCTGGCTGTGGCTCTGTTCCAAGTACTGCCCCAGCTGGTATGGGATGCCACAGACCGC
TTCCGTGGGCCAGACGCCCTGTGCCGGGCCGTCAAGTACCTGCAGATGGTGGGCATGTA
CGCCTCGTCCTACATGATCCTGGCCATGACGCTGGACCGCCACCGTGCCATCTGCCGCCC
CATGTTGGCCTACCGCCACGGAGGTGGGACTCACTGGAACCGGCCGGTGCTGGTGGCCT
GGGCCTTCTCGCTCATTCTCAGCCTGCCCCAGCTTTTCATCTTTGCCCAGCGTGACGTGG
GTAACGGCAGTGGGGTCCATGACTGCTGGGCCCACTTTGTGGAGCCCTGGGGTCTCCGA
GCCTATGTCACCTGGATCGCTCTGATGGTGTTCGTGGCCCCGGCCCTGGGCATCGCTGCC
TGCCAGGTGCTCATCTTCCGGGAGATTCATGCGAGCCTGCTGCTGGGGCCAGCAGAGAG
GGCCGGGGCTTGCCGGGGAGGGCACCGGACAGGCAGTCCCAGCGAGGGGGCCCGGGT
GTCAGCGGCCATGGCCAAGACCGTGCGGATGACGCTGGTGATTGTGATCGTGTACGTGCT
ATGCTGGGCGCCTTTCTTCCTCGTGCAGCTGTGGGCGGCGTGGGACCCGCAGGCGCCGG
TGGAAGAGGCCCCCTTCGTGCTGCTGATGTTGCTGGCCAGCCTGAACAGCTGCACCAACC
CCTGGATCTACGCCTTCTTCAGCAGCAGCGTCTCCTCGGAGCTGCGGAGCCTGCTCTGCG
GGGCCCGAAGTCGGGCCCCACCCAGCTCGGGCCCCCAGGAGGAGTCCTGCGCCACTGC
CAGCTCCTTCCTGGCCAAGGACACTTCCTCC

>Canis_lupus

ATGCTCCTGGCATCCACCAGCCCAGCTGTGCCCCGGACCCTCTCTCCACCTACTCCAGCT
GGCAACGGCAGC---
AGGGAGCTGCTGGACACCCGGGACCCGCTGCTTGTCCAGGCCGAGCTAGCCCTGCTCTC
CACGGTCTTCGTGGCTGTGGCCCTGAGCAACGGCCTGGTGCTGGGGGCCCTGGCGCGCC
GGGTCCGGCGGGGTCGCTGGGCACCCATGCACGTCTTCATTGGCCACTTGTGCCTGGCC
GACCTGGCTGTGGCTCTGTTCCAAGTACTGCCCCAGCTGGCGTGGGATGCCACGGACCG
CTTCCGTGGGCCTGATGCCCTGTGCCGGGCAGTCAAGTACCTGCAGATGGTGGGCATGTA
TGCCTCCTCCTACATGATCCTGGCCATGACGCTGGACCGCCACCGCGCCATCTGCCGCCC
CATGCTGGCATACCGCCACGGAGGTGGAGCTCGCTGGAACCGGCCGGTGCTGGTGGCCT
GGGCCTTCTCGCTCATTCTCAGCCTGCCCCAGCTCTTCATCTTTGCCCAGCGTGACGTGG
GAAATGGCAGTGGGGTCCTTGACTGCTGGGCCCACTTCGCTGAGCCCTGGGGCCTCCGA
GCCTATGTCACCTGGATCGCCCTAATGGTCTTTGTGGCACCTGCCCTGGGCATCGCGGCC
TGCCAGGTGCTCATCTTCCGGGAGATTCACTCCAGCCTGGTGCCGGGGCCAGCAGAGAG
GGCTGGGGGGTGCCGTGGAGGGCACCGGACCGGCAGCCCCAGCGAGGGGGCCCGGGT
GTCCGCAGCCATGGCCAAGACGGTGAGGATGACGCTGGTCATTGTGATCGTCTACGTGTT
GTGCTGGGCGCCCTTCTTCCTCGTGCAGCTGTGGGCAGCGTGGGACCCGCAGGCGCCCC
TGGAAGGGGCCCCCTTCGTGCTGCTTATGTTGCTGGCCAGCCTCAACAGCTGCACCAACC
CCTGGATCTACGCCTTCTTCAGCGGCACCGTCTCCTCCGAGCTGCGCAGCCTGTTCTGCT
GGGCCCGGAGTCGGGCCCCACCCAGCCTGGGGCCCCAAGAGGAGTCCTGCGCCACGGC
CAGCTCCTTCCTGGCCAAGGACACTTCCTCC

>Canis_lupus_familiaris

ATGCTCCTGGCATCCACCACCTCGGCTGTGCCCCGGACCCTCTCTCCACCTACTCCAGCT
GGCAACGGCAGC---
AGGGAGCTGCTGGACACCCGGGACCCGCTGCTTGTCCAGGCCGAGCTAGCCCTGCTCTC
CACGGTCTTCGTGGCTGTGGCCCTGAGCAACGGCCTGGTGCTGGGGGCCCTGGCGCGCC
GGGTCCGGCGGGGTCGCTGGGCACCCATGCACGTCTTCATTGGCCACTTGTGCCTGGCC
GACCTGGCTGTGGCTCTGTTCCAAGTACTGCCCCAGCTGGCGTGGGATGCCACGGACCG
CTTCCGTGGGCCTGATGCCCTGTGCCGGGCAGTCAAGTACCTGCAGATGGTGGGCATGTA
TGCCTCCTCCTACATGATCCTGGCCATGACGCTGGACCGCCACCGCGCCATCTGCCGCCC
CATGCTGGCATACCGCCACGGAGGTGGAGCTCGCTGGAACCGGCCGGTGCTGGTGGCCT
GGGCCTTCTCGCTCATTCTCAGCCTGCCCCAGCTCTTCATCTTTGCCCAGCGTGACGTGG
GAAATGGCAGTGGGGTCCTTGACTGCTGGGCCCACTTCGCTGAGCCCTGGGGCCTCCGA
GCCTATGTCACCTGGATCGCCCTAATGGTCTTTGTGGCACCTGCCCTGGGCATCGCGGCC
TGCCAGGTGCTCATCTTCCGGGAGATTCACTCCAGCCTGGTGCCGGGGCCAGCAGAGAG
GGCTGGGGGGTGCCGTGGAGGGCACCGGACCGGCAGCCCCAGCGAGGGGGCCCGGGT
GTCCGCAGCCATGGCCAAGACGGTGAGGATGACGCTGGTCATTGTGATCGTCTACGTGTT
GTGCTGGGCGCCCTTCTTCCTCGTGCAGCTGTGGGCAGCGTGGGACCCGCAGGCGCCCC
TGGAAGGGGCCCCCTTCGTGCTGCTTATGTTGCTGGCCAGCCTCAACAGCTGCACCAACC
CCTGGATCTACGCCTTCTTCAGCAGCAGCGTCTCCTCCGAGCTGCGCAGCCTGTTCTGCT
GGGCCCGGAGTCGGGCCCCACCCAGCCTGGGGCCCCAAGAGGAGTCCTGCGCCACGGC
CAGCTCCTTCCTGGCCAAGGACACTTCCTCC

6. Alignment of the mammalian species used in the *OXTR* analyses.

>Homo_sapiens

ATGGAGGGCGCGCTCGCAGCCAACTGGAGCGCCGAGGCAGCCAACGCCAGCGCCGCGC
CGCCGGGGGCCGAGGGCAACCGCACCGCCGGACCCCCGCGGCGCAACGAGGCCCTGG
CGCGCGTGGAGGTGGCGGTGCTGTGTCTCATCCTGCTCCTGGCGCTGAGCGGGAACGCG
TGTGTGCTGCTGGCGCTGCGCACCACACGCCAGAAGCACTCGCGCCTCTTCTTCTTCATG
AAGCACCTAAGCATCGCCGACCTGGTGGTGGCAGTGTTTCAGGTGCTGCCGCAGTTGCTG
TGGGACATCACCTTCCGCTTCTACGGGCCCGACCTGCTGTGCCGCCTGGTCAAGTACTTG
CAGGTGGTGGGCATGTTCGCCTCCACCTACCTGCTGCTGCTCATGTCCCTGGACCGCTGC
CTGGCCATCTGCCAGCCGCTGCGCTCGCTGCGCCGCCGCACCGACCGCCTGGCAGTGCT
CGCCACGTGGCTCGGCTGCCTGGTGGCCAGCGCGCCGCAGGTGCACATCTTCTCTCTGC
GCGAGGTGGCTGACGGCGTCTTCGACTGCTGGGCCGTCTTCATCCAGCCCTGGGGACCC
AAGGCCTACATCACATGGATCACGCTAGCTGTCTACATCGTGCCGGTCATCGTGCTCGCT
GCCTGCTACGGCCTTATCAGCTTCAAGATCTGGCAGAACTTGCGGCTCAAGACCGCTGCA
GCGGCGGCGGCCGAGGCGCCAGAGGGCGCGGCGGCTGGCGATGGGGGGCGCGTGGCC
CTGGCGCGTGTCAGCAGCGTCAAGCTCATCTCCAAGGCCAAGATCCGCACGGTCAAGATG

ACTTTCATCATCGTGCTGGCCTTCATCGTGTGCTGGACGCCTTTCTTCTTCGTGCAGATGT
GGAGCGTCTGGGATGCCAACGCGCCCAAGGAAGCCTCGGCCTTCATCATCGTCATGCTCC
TGGCCAGCCTCAACAGCTGCTGCAACCCCTGGATCTACATGCTGTTCACGGGCCACCTCT
TCCACGAACTCGTGCAGCGCTTCCTGTGCTGCTCCGCCAGCTACCTGAAGGGCAGACGCC
TGGGAGAGACGAGTGCCAGCAAAAAGAGCAACTCGTCCTCCTTTGTCCTGAGCCATCGCA
GCTCCAGCCAGAGGAGCTGCTCCCAGCCATCCACGGCG---

>Pan_troglodytes

ATGGAGGGCGCGCTCGCAGCCAACTGGAGCGCCGAGGCAGTCAACGCCAGCGCCGCGC
CGCCGGGGGCCGAGGGCAACCGCACCGCCGGACCCCCGCGGCGCAACGAGGCCCTGG
CGCGCGTGGAGGTGGCGGTGCTGTGTCTCATCCTGTTCCTGGCGCTGAGCGGGAATGCG
TGTGTGCTGCTGGCGCTGCGCACCACACGCCAGAAGCACTCGCGCCTCTTCTTCTTCATG
AAGCACCTAAGCATCGCCGACCTGGTGGTGGCAGTGTTCCAGGTGCTGCCGCAGTTGCTG
TGGGACATCACCTTCCGCTTCTACGGGCCCGACCTGCTGTGCCGCCTGGTCAAGTACTTG
CAGGTGGTGGGCATGTTCGCCTCCACCTACCTGCTGCTGCTCATGTCCCTGGACCGCTGC
CTGGCGATCTGCCAGCCGCTGCGCTCGCTGCGCCGCCGCACCGACCGCCTGGCAGTGCT
CGCCACGTGGCTCGGCTGCCTGGTGGCCAGCGCGCCGCAGGTGCACATCTTCTCTCTGC
GCGAGGTGGCTGACGGCGTCTTCGACTGCTGGGCCGTCTTCATCCAGCCCTGGGGACCC
AAGGCCTACATCACGTGGATCACGCTAGCTGTCTACATCGTGCCGGTCATCGTGCTCGCT
GCCTGCTACGGCCTTATCAGCTTCAAGATCTGGCAGAACTTGAGGCTCAAGACCGCTGCA
GCGGCGGCGGCCGAGGCGCCAGAGGGCGCGGCGGCTGGCGATGGGGGGCGCGTGGCC
CTGGCGCGTGTCAGCAGCGTCAAGCTCATCTCCAAGGCCAAGATCCGCACGGTCAAGATG
ACTTTCATCATCGTGCTGGCCTTCATCGTGTGCTGGACGCCTTTCTTCTTCGTGCAGATGT
GGAGCGTCTGGGATGCCAACGCGCCCAAGGAAGCCTCGGCCTTCATCATCGTCATGCTCC
TGGCCAGCCTCAACAGCTGCTGCAACCCCTGGATCTACATGCTGTTCACGGGCCACCTCT
TCCACGAACTCGTGCAGCGCTTCCTGTGCTGCTCCGCCAGCTACCTGAGGGGCAAACGCC
TGGGAGAGACGAGTGCCAGCAAAAAGAGCAACTCGTCCTCCTTTGTCCTGAGCCATCGCA
GCTCCAGCCAGAGGAGCTGCTCCCAGCCATCCACGGCG---

>Mus_musculus

ATGGAGGGCACGCCCGCAGCCAACTGGAGTATCGAGTTGGACCTCGGGAGTGGAGTGCC
ACCAGGGGCGGAGGGTAACCTCACGGCCGGGCCGCCACGACGCAACGAGGCCCTGGCG
CGCGTGGAGGTGGCGGTCCTGTGTCTCATACTGTTCCTGGCTCTGAGTGGCAACGCGTGC
GTGCTGCTGGCGCTGCGTACGACGCGCCACAAGCACTCGCGCCTCTTCTTTTTCATGAAG
CACCTGAGCATCGCCGACCTGGTGGTGGCCGTGTTCCAGGTTCTCCCGCAGCTGCTGTG
GGACATCACCTTCCGCTTCTACGGGCCCGACCTGCTGTGTCGTCTGGTCAAATACTTGCA
GGTGGTGGGCATGTTCGCCTCCACCTACCTGCTGTTGCTGATGTCGCTCGACCGCTGCCT
GGCCATCTGCCAGCCGCTGCGCTCACTGCGCCGCCGAACCGACCGCCTGGCGGTGCTGG
CGACGTGGCTCGGCTGCCTGGTGGCCAGCGTGCCGCAGGTGCACATTTTCTCGCTGCGC
GAAGTGGCGGACGGCGTCTTCGATTGCTGGGCGGTCTTCATCCAGCCCTGGGGACCCAA
GGCCTACGTCACGTGGATCACGCTCGCCGTCTACATTGTACCGGTCATCGTGCTGGCCGC
CTGCTATGGTCTCATCAGCTTCAAGATCTGGCAGAATCTGCGACTCAAGACGGCAGCCGC
GGCGGCAGCCGAGGGGAGTGA------
CGCAGCCGGTGGAGCTGGCCGTGCGGCGTTGGCACGGGTCAGTAGTGTCAAGCTTATCT
CCAAGGCCAAAATCCGCACAGTGAAGATGACCTTCATCATTGTTCTGGCCTTCATCGTGTG
CTGGACGCCTTTCTTCTTCGTGCAGATGTGGAGCGTCTGGGACGTCAATGCGCCCAAAGA
AGCTTCTGCCTTCATCATTGCCATGCTCTTGGCCAGCCTCAACAGCTGCTGCAACCCATGG
ATCTACATGCTCTTCACGGGCCATCTCTTCCACGAACTCGTGCAGCGCTTCCTCTGCTGCT
CTGCTCGGTACCTGAAGGGCAGCCGGCCTGGAGAGACGAGCATTAGCAAGAAAAGCAACT
CCTCCACCTTCGTCCTGAGTCGTCGCAGCTCGAGTCAGAGGAGCTGTTCTCAACCATCCT
CGGCA---

>Rattus_norvegicus

ATGGAGGGCACGCCAGCAGCCAACTGGAGTGTCGAGTTGGACCTCGGGAGTGGAGTGCC
ACCAGGGGAGGAGGGCAACCGCACGGCCGGGCCACCACAACGCAACGAGGCCCTGGCG
CGCGTGGAGGTGGCGGTCCTGTGCCTCATTCTGTTCCTGGCTCTGAGTGGCAACGCGTGT
GTGCTGCTGGCGCTGCGCACCACGCGCCACAAGCACTCGCGCCTCTTCTTTTTCATGAAG
CACCTGAGCATCGCTGACCTGGTGGTGGCTGTGTTTCAGGTGCTTCCGCAGCTGCTGTGG
GACATCACCTTCCGCTTCTATGGGCCCGACCTGCTGTGTCGTCTGGTCAAATACTTGCAGG

TGGTGGGCATGTTCGCCTCCACCTACCTGCTGCTGCTGATGTCGCTCGACCGCTGTCTGG
CCATCTGCCAGCCGCTGCGCTCCCTGCGCCGCCGAACCGACCGCCTGGCGGTGCTGGG
GACGTGGCTTGGCTGCCTGGTGGCCAGTGCTCCGCAGGTGCACATTTTCTCGCTGCGCGA
AGTGGCGGACGGAGTCTTCGATTGCTGGGCGGTCTTCATCCAACCCTGGGGACCCAAGG
CCTACGTCACATGGATCACGCTCGCCGTCTACATTGTACCGGTCATCGTGCTGGCCGCCT
GCTACGGCCTCATCAGCTTCAAGATCTGGCAGAATCTGAGACTCAAGACGGCAGCGGCGG
CGGCGGCCGAGGGGAATGA------
CGCGGCGGGTGGAGCTGGGCGTGCGGCGTTGGCACGGGTCAGTAGTGTCAAGCTTATCT
CCAAGGCCAAAATCCGCACGGTGAAGATGACCTTCATCATCGTACTGGCCTTCATCGTGTG
CTGGACACCTTTCTTCTTCGTGCAGATGTGGAGCGTTTGGGACGTCAATGCGCCCAAGGA
AGCTTCTGCCTTCATCATTGCCATGCTCTTGGCCAGCCTCAACAGCTGCTGCAACCCGTGG
ATCTACATGCTCTTCACAGGTCACCTCTTCCACGAACTCGTGCAGCGCTTCTTCTGCTGCT
CTGCTCGTTACCTGAAGGGCAGCCGGCCCGGCGAGACGAGTGTCAGCAAGAAGAGCAAC
TCATCCACCTTTGTCCTGAGCCGTCGCAGCTCAAGCCAGAGGAGCTGCTCTCAGCCATCTT
CAGCA---

>Peromyscus_maniculatus

ATGGAGGGCATGCCCGCAGCCAACTGGAGCATCGAGTTGGACCTCGGGAGTGGAGTGCC
GCCTGGGGTGGAGGGGAACCTCACGGCCGGGCCGCCGCGGCGCAACGAGGCCCTGGC
GCGCGTGGAGGTGGCGGTGCTGTGCCTCATTCTGTTCCTGGCGCTGAGCGGCAACGCGT
GCGTGCTGCTGGCGCTGCGCACCACGCGCCACAAGCACTCGCGCCTCTTCTTTTTCATGA
AGCACCTGAGCATCGCCGACCTGGTGGTGGCTGTGTTCCAGGTGCTCCCGCAGCTGCTGT
GGGACATCACCTTCCGCTTCTATGGGCCCGACCTGCTGTGTCGTCTGGTCAAATACTTGCA
GGTGGTGGGCATGTTCGCCTCCACCTACCTGCTGCTGCTCATGTCGCTCGACCGCTGCCT
GGCCATCTGCCAGCCGCTGCGCTCGCTGCGCCGCCGAACCGACCGCCTGGCAGTACTGG
CGACGTGGCTCGGCTGCCTGGTGGCCAGCGCGCCGCAGGTGCACATTTTCTCGCTGCGC
GAAGTGGCGGACGGCGTCTTTGACTGCTGGGCTGAGTTCATCCAGCCCTGGGGACCCAA
GGCTTACGTCACGTGGATCACGCTCGCCGTCTACATTGTGCCTGTCATCGTGCTGGCCGC
CTGCTATGGCCTCATCAGCTTCAAGATCTGGCAGAACTTGCGACTCAAGACGGCAGCGGC
TGCGGAGGCCCAGGGGACTGAAGGATCTGCAGCCGGTGGAGCTGGGCGGGCGGCGCTG
GCTCGGGTCAGTAGCGTCAAGCTCATCTCCAAGGCGAAGATCCGCACAGTGAAGATGACC
TTTATCATCGTACTGGCCTTCATCGTGTGCTGGACGCCTTTCTTCTTCGTGCAGATGTGGA
GCGTCTGGGACGTCAATGCGCCCAAGGAAGCTTCTGCCTTCATCATCGCCATGCTCTTGG
CCAGCCTCAACAGCTGCTGCAACCCTTGGATCTACATGCTCTTCACGGGCCACCTCTTCCA
CGAACTTGTGCAACGCTTCCTCTGCTGCTCTGCCCGTTACCTGAAGGGCAGCCGGCCTGG
AGAGACAAGCGTCAGCAAGAAGAGCAACTCATCTACCTTTGTCCTGAGTCGCCGCAGCTC
CAGCCAGAGGAGCTGCTCTCAACCATCTTCAGCG---

>Ictiodomys_tridecemlineatus

ATGGAGGGCGCTTTCTCAGCCAACTGGAGCGCCGAGGCGGTCAATGGGAGCGCCGCACC
TCCTGGGGCCGAAGACAACTGCACTGCCGGACCTCCGCGGCGCAACGAGGCACTGGCAC
GCGTGGAGGTGGCAGTACTGTGCCTCATTCTCTTCCTGGCACTGAGCGGCAACGCGTGCG
TGCTGCTGGCACTGCGCACCACGCGCCACAAGCACTCACGCCTCTTTTTCTTCATGAAACA
CCTGAGTATCGCTGACTTGGTGGTAGCTGTGTTCCAGGTGCTGCCGCAGCTACTGTGGGA
TATCACCTTCCGTTTCTATGGGCCCGACCTGTTGTGCCGTCTAGTCAAGTACCTGCAGGTG
GTGGGGATGTTCGCCTCCACCTACCTGCTGCTGCTCATGTCGCTTGACCGCTGCCTGGCC
ATCTGCCAGCCGCTGCGATCGCTGCGCCGGCGTACCGACCGCCTGGCGGTGCTTGCCAC
GTGGCTTGGCTGCCTAGTGGCCAGCGCCCCGCAGGTGCACATCTTCTCGCTGCGCGAGG
TGGCTGAAGGCGTCTTTGATTGCTGGGCGGTCTTCATCCAGCCCTGGGGACCCAAGGCCT
ACGTCACGTGGATCACACTCGCCGTCTACATCGTGCCAGTCATCGTACTCGCTGCCTGTTA
CGGCCTCATCAGCTTCAAGATCTGGCAGAACTTGAGGCTCAAGACGGCTGCAGCAGCTCA
GGCTCAGGGACCCGAGGGTTCAGCAGCGGGTAGTGAGGGTCGTGCGGCCCTGGCACGA
GTCAGCAGTGTCAAGCTCATCTCCAAAGCCAAGATCCGCACGGTCAAGATGACCTTCATCA
TCGTGCTGGCCTTCATCGTATGCTGGACACCATTCTTCTTCGTGCAGATGTGGAGCGTCTG
GGATACTAATGCACCCAAGGAAGCCTCGGCCTTCATCATTGCCATGCTCCTGGCCAGCCT
CAACAGCTGCTGCAACCCCTGGATCTACATGCTCTTCACGGGCCACCTCTTCCATGAACTG
GTACAGCGCTTCCTCTGCTGCTCCTCCAGCCATCTGAAGGGTAGCCGCCCAGGAGAGACT
AGCGTCAGCAAAAAGAGCAACTCCTCCACATTTGTCTTGAGCCGACGCAGCTCCAGCCAG
AGGAGCTGCTCCCAGCCATCAACAGCG---

>Cavia_porcellus

ATGGCGGGCGCGCTAGCCGCCAACTGGAGCGCGGACGCAGCCAACGCGAGCGCCGTCC
TTCCAGGGTCCGAGCGCAACTCCACGGCCGGACCGCCGCAGCGCAACGAGGCCCTGGC
GCGCGTGGAGGTGGCCGTGCTGTGCGTCATCCTCTTCCTGGCGCTGAGCGGCAACGCGT
GTGTGCTCCTGGCGCTGCGCACCACGCGCCACAAGCACTCGCGCCTCTTCTTCTTCATGA
AGCACCTGAGCATCGCCGACCTGGTGGTGGCCGTGTTCCAGGTGCTACCACAGCTGCTGT
GGGACATCACCTTCCGCTTCTACGGGCCCGATCTGCTATGCCGTCTGGTCAAGTACCTGC
AGGTGGTGGGCATGTTCGCCTCCACCTACCTGCTGCTGCTCATGTCCCTGGACCGCTGCC
TGGCCATCTGCCAGCCGCTGCGCTCGCTGCGCCGCCGCACTGACCGCTTGGCTGTGCTC
GCCACGTGGCTGGGCTGCCTAGTGGCCAGCGTGCCGCAGGTGCACATCTTCTCCCTGCG
CGAGGTGGCCGAGGGTGTCTTTGACTGCTGGGCCGTCTTTATCCAGCCCTGGGGGCCCA
AGGCCTATGTCACGTGGATCACTCTGGCCGTCTACATTGTGCCAGTCATCGTGCTGGCTG
CCTGCTACGGCCTCATCAGCTTCAAGATCTGGCAGAACCTGAAGCTTAAGACAGCGGCCG
AGGCTGCAGCCCAGGGGCCGGAGGACTCGGCCACTGCCGGCACAGAGCGAGCGGCGCT
GGCCAGAGTCAGCAACGTCAAGCTGATCTCCAAGGCCAAGATCCGCACGGTCAAGATGAC
CTTCATCGTCGTGCTGGCCTTCATTGTATGCTGGACGCCCTTCTTCTTCGTGCAGATGTGG
AGCGTGTGGGATGCAGATGCGCCCAAGGAAGCCTCGGCCTTCATCATTGCCATGCTCCTG
GCCAGCCTCAACAGCTGCTGCAACCCCTGGATCTACATGCTCTTCACTGGCCACCTCTTCC
ACGAACTTGTGCAGCGCTTCCTGTGCTGCTACCCCCGCCACCTGCGGGGCAGCTGGCCG
GGAGAGACAAGCATCAGCAGAAAGAGCAACTCGTCAACCTTCGTGCTGAGCCAGCGCAGC
TCCAGCCAGAGGAGCTGCTCCCAGCCATCTACGGCG---

>Octodon_degus

ATGGAAGGCGCGCTTGCGGTCAACTGGAGCGCCGACGCAGCCAACGGGAGCACCGAGTC
CCCGGGCTCCGAGAGCAACAGCACGGCCGGACCCCCGAGGCGCAACGAGGCCCTGGCG
CGCGTGGAGGTGGCGGTGCTGTGCCTCATCCTCTTCCTGGCGCTGAGCGGCAACGCGTG
CGTGCTCCTGGCGCTGCGCACCACGCGCCACAAGCATTCTCGCCTCTTCTTCTTCATGAA
GCACCTGAGCATCGCCGACCTGGTGGTGGCCGTGTTCCAGGTGCTGCCGCAACTGCTGT
GGGACATCACCTTCCGTTTCTACGGGCCTGACCTGTTGTGCCGCCTCGTCAAGTACTTGCA
GGTGGTAGGCATGTTCGCCTCCACCTACCTGCTGCTGCTCATGTCGCTGGACCGCTGCCT
GGCCATCTGCCAGCCTCTGCGCTCGCTGCGCCGCCGCACTGACCGCCTGGCTGTGCTTG
CCACGTGGCTGGGCTGTCTGGTGGCCAGCGTGCCGCAGGTGCACATCTTCTCCCTGCGC
GAGGTGGCCGAGGGCGTTTTTGACTGCTGGGCAGTCTTCATCCAGCCCTGGGGGCCCAA
GGCCTACGTCACATGGATCACACTGTCTGTCTACATTGTGCCGGTGATCGTGCTGGCCGC
CTGCTATGGCCTCATCAGCTTCAAGATCTGGCAGAATCTGCGGCTCAAGACGGCCGCTGC
GGCCGCCGCCCAGGGGCCCGAGGACTCCACCGTGACCGGTGCAGGGCGAGCAACGCTG
GCGAGAGTCAGCAACGTCAAGCTTATCTCCAAGGCCAAGATCCGCACGGTCAAGATGACC
TTCATCATCGTGCTGGCCTTCATCGTGTGCTGGACACCCTTCTTCTTCGTGCAGATGTGGA
GTGTGTGGGATGCAGATGCGCCCAAAGAAGCCTCAGCCTTCATCATCGCCATGCTGCTGG
CCAGCCTCAACAGCTGCTGCAACCCCTGGATCTACATGCTCTTCACCGGCCACCTCTTCCA
CGAACTCGTGCAGCGCTTCCTCTGCTGCTCCGCCAGCCACCTGAGGGGCAGCCGGCCAG
GAGAGACCAGCGTTAGCAAAAAGAGCAACTCCTCGACTTTCGTCCTAAGCCAGCGCAGCT
CCAGCCAGAGGAGCTGTTCCCAGCCGTCCACAGCG---

>Chinchilla_lanigera

ATGGAGGGCGCGCTTGCCTGCAACTGGAGCGCGGACGCGGCCAACGCGAGCGCCGCGC
CTCCGGACCACGAGCGCAACTGCACTGCCGGACCGCCGCGGCGCAACGAGGCCCTGGC
GCGCGTGGAGGTGGCCGTGCTGTGCCTCATCCTCTTCCTGGCCCTGAGCGGCAACGCGT
GCGTGCTCCTGGCGCTGCGCACCACGCGCCACAAGCACTCGCGCCTCTTCTTCTTCATGA
AGCACCTGAGCATCGCCGACCTGGTGGTGGCCGTGTTCCAGGTGCTGCCGCAGCTTCTGT
GGGACATCACCTTCCGATTCTACGGGCCTGATCTGCTGTGCCGCCTGGTCAAGTACTTGC
AGGTGGTGGGCATGTTCGCCTCCACCTACCTGCTGCTGCTCATGTCCCTGGACCGCTGCC
TGGCCATCTGCCAGCCGCTGCGCTCGCTGCGCCGCCGCACTGACCGTCTGGCCGTGCTC
GCCACGTGGCTGGGCTGCCTGGTGGCCAGCGTGCCTCAGGTGCACATCTTTTCCCTGCG
CGAGGTGGCCGAGGGCGTCTTTGACTGCTGGGCGGTCTTCATCCAGCCCTGGGGGCCCA
AGGCCTACGTCACATGGATCACGTTGGCTGTCTACATTGTGCCGGTCATCGTGCTGGCCG
CCTGCTATGGGCTCATCAGCTTCAAGATCTGGCAGAATCTGCGGCTCAAGACGGCAGCCG
CGGCTGCAGCCCAGGGGCCCGAGGACTCAGCTGTGGCCGGCACAGGGCGAGCGGCCCT
GGCCAGAGTCAGTAACGTCAAGCTCATCTCCAAGGCCAAGATCCGCACGGTCAAGATGAC

CTTCATCGTCGTGCTGGCCTTCATCGTGTGCTGGACACCCTTCTTCTTTGTGCAGATGTGG
AGCGTGTGGGATGCAGATGCGCCTAAGGAAGCCTCAGCCTTCATCATCGCCATGCTCCTG
GCCAGCCTCAATAGCTGCTGCAACCCCTGGATCTACATGCTCTTCACTGGCCACCTCTTCC
ATGAACTCATGCAGCGCTTCCTCTGCTGCTCCTCCAGCTCCTTGAGGAGCAGCCGGCCGG
GGGAGACTAGCATCAGCAAAAAGAGCAACTCATCCACCTTTGTCTTGAGCCAGCGCAGCT
CCAGCCAGAGGAGCTGCTCCCAGCCGTCCACAGCG---

>Heterocephalus_glaber

ATGGAGGGCGCGCTCATCGCCAACTGGAGCGCAGACGCGGCTAACGAGAGCGCCGCGC
CTGGGGACCCAGAGCGCAACTGCACTGCCCAACCGCCGCGGCGCAACGAGGCCCTGGC
GCGCGTGGAGGTGGCGGTGCTGTGCCTCATCCTCTTCCTGGCGCTGAGCGGCAACGCGT
GCGTGCTCCTGGCGCTGCGCACCACGCGCCACAAGCACTCGCGTCTCTTCTTCTTCATGA
AGCACCTGAGCATCGCCGACCTGGTGGTGGCTGTGTTCCAGGTGCTGCCGCAGCTGCTG
TGGGACATCACCTTCCGCTTCTACGGCCGGACCTGCTGTGCCGCCTGGTCAAGTATCTA
CAGGTGGTGGGCATGTTCGCCTCCACCTACCTGCTGCTGCTCATGTCCCTGGACCGCTGC
CTGGCCATCTGCCGGCCGCTGCGCTCGCTGCGCCGCCGCACCGACCGCCTGGCCGTGCT
CGGCACGTGGCTGGGCTGCCTGGTGGCCAGCGCGCCGCAAGTGCACATCTTCTCCCTGC
GCGAGGTGGCCGAGGGCGTCTTCGACTGCTGGGCTGTCTTCATCCAGCCCTGGGGGCCC
AAGGCCTACGTCACGTGGATCACACTGTCCGTCTATATTGTGCCGGTCATCGTGCTGGCC
GCCTGCTACAGCCTCATCAGCTTCAAGATCTGGCAGAACCTGCGGCTCAAGACGGCTGCG
GAGGCCGCAGCCCAAGGGCCCGGGGACTCCGACGTGGGCGGCGCAGGGCGTGTGGCG
CTGGCGCGAGTCAGCAGCGTCAAGCTCATCTCCAAGGCCAAGATCCGCACCGTCAAGATG
ACCTTCATCATCGTGCTGGCCTTCATCGTGTGCTGGACGCCCTTCTTCTTCGTGCAGATGT
GGAGCGTATGGGACGCGGATGCGCCCAAGGAAGCCTCGGCCTTCATCATTGCCATGCTG
CTGGCCAGCCTCAACAGCTGCTGCAACCCCTGGATCTACATGCTTTTCACCGGCCACCTCT
TCCACGAACTCGTGCAGCGCTTCCTCTGCTGCTCCCCCAGCTCCCTGAAAGGTAGCCGTT
CAAGAGAGACGAGTGTCAGCAAAAAGAGCAACTCATCCACCTTTGCCTTGAGTCAGCGCA
GCTCCAGCCAGAGGAGCTGCTCCCAGCCGTCCACAGCG---

>Ochotona_princeps

ATGGAGGGCATTCTGGCAGCCAACTGGAGCGCCGAGGCGGC------
AAGCACGGGACCACCGGGGACCGAGGGCAACCGCACGGCGGGGCCACCGCGGCGCAAC
GAGGCCTTGGCGCGCGTGGAGGTGGCCGTGCTGTGCCTCATCCTCTTCCTGGCACTAAGT
GGCAACGCGTGTGTGCTGCTGGCGCTGCGCACCACGCGCCACAAACACTCACGCCTCTTT
TTCTTCATGAAGCACCTAAGCATTGCAGACCTGGTGGTGGCGGTGTTCCAGGTGCTGCCG
CAGCTGCTGTGGGACATCACCTTCCGTTTCTACGGGCCTGATTTGCTGTGCCGCCTGGTC
AAGTACCTGCAGGTGGTCGGCATGTTCGCCTCCACCTATATGCTGCTGCTGATGTCGCTG
GACCGCTGCCTGGCCATCTGCCGGCCACTGCGCGCGCTGCGCCGCCGCACCGACCGCC
TAGCTGTGCTCGCCACGTGGCTCGGCTGCCTGGTGGCCAGCGCCCCGCAGGTGCACATC
TTCTCACTGCGCGAGGTGGCGGAGGGCGTCTTCGACTGCTGGGCCGTCTTCATCCAGCC
CTGGGGTCCCAAGGCCTACGTCACATGGATCACGCTGGCCGTGTACATCGTGCCGGTCAT
CGTGCTGGCTGCCTGCTACGGCCTCATCAGCTTCAAGATCTGGCAGAACCTGCGCCTTAA
GACGCTGGCGGCCGCGGCGGCCCAGGAGCCCGAGGGGGCGGTGGCGG---
GCGCAGGGCGCGCGGCTCTGGCGCGGGTCAGCAGCGTCAAGCTCATCTCCAAGGCCAAG
ATCCGCACGGTCAAGATGACCTTCATCATCGTACTTGCCTTCATTGTGTGCTGGACGCCCT
TCTTCTTCGTGCAGATGTGGAGCGTCTGGGACGCGGACGCGCCCAAGGAAGCCTCGGCC
TTCATCATCGCCATGCTCCTGGCCAGCCTCAACAGCTGCTGCAACCCCTGGATCTACCTGC
TGTTCACTGGCCATCTCTTCCATGAACTGGTGCAGCGCTTCCTGTGCTGCTCACAAGGCTA
TGTGACAAGCGGCCGGCAGGCCGAGACAAGCGCCAGCAAGAAGAGCAACTC---
CACCTTCGTGCTCAGCCGCCGCAGCTCCAGCCAGCAGAGTGGCTCGCAGCCCACCACAG
TG---

>Oryctolagus_cuniculus

ATGGAGGGCGCGCTGGTGGCCAACTGGAGCACGGAGGCGGTGGGCGCGAGTGCGGTGT
CTCCGGGGACTACGGAGAACGGCACGGTCGGGCCGCCGCAGCGCAACGAGGCCCTAGC
GCGCGTGGAGGTGGCCGTGCTGTGCCTCATCCTCTTCCTGGCGCTGAGCGGGAACGTGT
GCGTGCTGCTGGCGCTGCGCACCACGCGCCACAAGCACTCGCGCCTCTTCTTCTTCATGA
AGCACCTGAGCATTGCCGACCTGGTGGTGGCCGTGTTCCAGGTGTTGCCGCAGCTGCTGT
GGGACATCACCTTCCGCTTCTACGGGCCCGACCTGCTGTGCCGCCTCGTCAAGTACCTGC

AGGTGGTGGGCATGTTTGCCTCCACCTACATGCTGCTGCTCATGTCGCTGGACCGCTGCC
TGGCCATCTGCCGGCCGCTGCGCACGCTGCGCCGCCGCACCGACCGCCTCGCGGTGCT
CGCCACGTGGCTCGGCTGCCTGGTGGCCAGCGCGCCGCAGGTGCACATCTTCTCGCTGC
GCGAGGTGGCCGAGGGCGTCTTTGACTGCTGGGCGGTCTTCATCCAGCCCTGGGGGCCC
AAGGCCTACGTCACGTGGATCACGCTCGCCGTCTACATCGTGCCCGTCATCGTGCTGGCC
GCCTGCTACGGCCTCATCAGCTTCAAGATCTGGCAGAACCTGCGGCTCAAGACGCTGGCG
GCGGCGGCGGCCGCGGGGCCCGAGGGCGCGGCGGCGGGCGGCGCAGAGCGCGCAGC
CCTGGCGCGGGTCAGCAGCGTGAAGCTCATTTCCAAGGCCAAGATCCGCACGGTCAAGAT
GACCTTCATCATCGTGCTGGCCTTCATCGTGTGCTGGACGCCATTCTTTTTCGTGCAGATG
TGGAGCGTCTGGGATGCTGACGCGCCCAAGGAAGCCTCGGCCTTCATCATCGCCATGCTC
CTGGCCAGCCTCAACAGCTGCTGCAACCCCTGGATCTACCTGCTCTTCACCGGGCACCTC
TTCCAGGAACTGGTGCAGCGCTTCCTCTGCTGCTCCCCTGGCTACCGGAGAGGGGGCCG
GCAGGGCGAGACCAGCGTCAGCAAGAAGAGCAACTCCTCCACCTTCGTGCTGAGCCGGC
GCAGCTCCAGCCAGCGGAGCTGCTCCCAGCCCACCACTGTG---

>Ovis_orientalis

ATGGAGGGCGCGTTTGCGGCAAACTGGAGCGCGGAGGCGGTCAACGGGAGCGCGGCGC
CTCCGGGAACCGAGGGCAATCGCACTGCCGGGCCGCCACAGCGCAACGAGGCCCTGGC
GCGGGTGGAGGTGGCCGTGCTGTCCCTCATCCTGTTCCTGGCGCTGAGCGGCAACGCGT
GCGTGCTGCTGGCGCTGCGCACCACGCGCCACAAGCACTCGCGCCTCTTCTTCTTCATGA
AGCACCTGAGCATAGCCGACCTGGCGGTGGCGGTGTTCCAGGTGCTGCCGCAGCTTCTG
TGGGACATCACGTTCCGTTTCTACGGGCCCGACCTGCTGTGCCGCCTCGTCAAGTACCTG
CAGGTGGTGGGCATGTTCGCGTCCACCTACCTGCTGCTGCTCATGTCGCTCGACCGCTGC
CTGGCCATCTGCCAGCCGCTGCGCTCGCTGCGCCGCCGCACCGACCGCCTGGCGGTACT
CGCCACCTGGCTCGGCTGCCTGGTGGCCAGCGCGCCGCAGGTGCACATCTTCTCGCTGC
GCGAGGTGGCCGACGGCGTCTTCGACTGCTGGGCCGTTTTCATTCAGCCCTGGGGGCCC
AAGGCCTACATCACGTGGATCACGCTCGCCGTCTACATTGTGCCGGTCATCGTGCTTGCC
GCCTGCTACGGCCTTATCAGCTTCAAGATCTGGCAGAACTTACGGCTCAAGACGGAGGCG
GCGGCGGCAGCCGCGGGGGCTGAGGGCGCGGCGGCAGACTGCGCGGGGCGCGCGGCT
CTGGCCCGCGTCAGCAACGTCAAGCTCATCTCTAAGGCTAAGATCCGCACGGTCAAGATG
ACCTTCATCGTCGTGCTGGCCTTCATCGTGTGCTGGACGCCATTCTTTTTCGTGCAGATGT
GGAGTGTCTGGGATGCCGATGCGCCCAAGGAAGCCTCGGCTTTCATCATCGCCATGCTTC
TGGCCAGCCTCAACAGCTGCTGCAACCCCTGGATCTACATGCTCTTCACGGGCCACCTCT
TCCAAGACCTTGTGCAGCGCTTCCTCTGCTGCTCATTCCGCCGCCTGAAAGGCAGCCAGC
CTGGGGAGACGAGCGTCAGCAAAAAGATCCATTCGTACACCTTTGTCCTGAGCCGGCACA
GCTCCAGCCAGAGAAGCTGCTCGC-----------------

>Ovis_aries

ATGGAGGGCGCGTTTGCGGCAAACTGGAGCGCGGAGGCGGTCAACGGGAGCGCGGCGC
CTCCGGGAACCGAGGGCAATCGCACTGCCGGGCCGCCACAGCGCAACGAGGCCCTGGC
GCGGGTGGAGGTGGCCGTGCTGTCCCTCATCCTGTTCCTGGCGCTGAGCGGCAACGCGT
GCGTGCTGCTGGCGCTGCGCACCACGCGCCACAAGCACTCGCGCCTCTTCTTCTTCATGA
AGCACCTGAGCATAGCCGACCTGGCGGTGGCGGTGTTCCAGGTGCTGCCGCAGCTTCTG
TGGGACATCACGTTCCGTTTCTACGGGCCCGACCTGCTGTGCCGCCTCGTCAAGTACCTG
CAGGTGGTGGGCATGTTCGCGTCCACCTACCTGCTGCTGCTCATGTCGCTCGACCGCTGC
CTGGCCATCTGCCAGCCGCTGCGCTCGCTGCGCCGCCGCACCGACCGCCTGGCGGTACT
CGCCACCTGGCTCGGCTGCCTGGTGGCCAGCGCGCCGCAGGTGCACATCTTCTCGCTGC
GCGAGGTGGCCGACGGCGTCTTCGACTGCTGGGCCGTTTTCATTCAGCCCTGGGGGCCC
AAGGCCTACATCACGTGGATCACGCTCGCCGTCTACATTGTGCCGGTCATCGTGCTTGCC
GCCTGCTACGGCCTTATCAGCTTCAAGATCTGGCAGAACTTACGGCTCAAGACGGAGGCG
GCGGAGGCTGCCGCGGGGGCTGAGGGCGCGGCGGCAGACTGCGCGGGGCGCGCGGCT
CTGGCCCGCGTCAGCAACGTCAAGCTCATCTCTAAGGCTAAGATCCGCACGGTCAAGATG
ACCTTCATCGTCGTGCTGGCCTTCATCGTGTGCTGGACGCCATTCTTTTTCGTGCAGATGT
GGAGTGTCTGGGATGCCGATGCGCCCAAGGAAGCCTCGGCTTTCATCATCGCCATGCTTC
TGGCCAGCCTCAACAGCTGCTGCAACCCCTGGATCTACATGCTCTTCACGGGCCACCTCT
TCCAAGACCTTGTGCAGCGCTTCCTCTGCTGCTCATTCCGCCGCCTGAAAGGCAGCCAGC
CTGGGGAGACGAGCGTCAGCAAAAAGATCCATTCGTACACCTTTGTCCTGAGCCGGCACA
GCTCCAGCCAGAGAAGCTGCTCGCAGCCATCCACGGTG---

>Bos_taurus

ATGGAGGGTGCGTTTGCGGCTAACTGGAGCGCTGAGGCGGTCAACGGGAGCGCGGCGC
CGCCGGGAACCGAGGGCAATCGCACTGCCGGGCCGCCACAGCGCAACGAGGCCCTGGC
GCGGGTGGAGGTGGCCGTGCTGTGCCTCATCCTGTTCCTGGCGCTGAGCGGCAACGCGT
GCGTGCTGCTAGCGCTGCGCACCACGCGCCACAAGCACTCGCGCCTCTTCTTCTTCATGA
AGCACCTGAGCATAGCCGACCTGGTAGTGGCGGTGTTCCAGGTGCTGCCGCAGCTTCTGT
GGGACATCACGTTCCGCTTCTACGGGCCCGACCTGCTGTGCCGCCTCGTCAAGTACCTGC
AGGTTGTGGGCATGTTCGCGTCCACCTACCTGCTGCTGCTCATGTCGCTCGACCGCTGCC
TGGCCATCTGCCAGCCGCTGCGCTCGCTGAGCCGCCGCACCGACCGCCTGGCGGTACTC
GTCACATGGCTCGGCTGCCTGGTGGCCAGCGCGCCGCAGGTGCACATCTTCTCGCTGCG
CGAGGTGGCCGACGGTGTCTTCGACTGCTGGGCCGTTTTCATTCAACCCTGGGGGCCCAA
GGCCTACATCACGTGGATCACGCTCGCCGTCTACATTGTGCCCGTCATCGTCCTTGCCAC
CTGCTATGGCCTTATCAGCTTCAAGATCTGGCAGAATTTACGGCTCAAGACGGCGGCGGC
GGAGGCTGCCGCGGGGGCTGAGGGCGAGGCGGCAGACTGGGCGGGGCGCGCGATTCT
GGCCCGCGTCAGCAACGTCAAGCTCATCTCTAAGGCCAAGATCCGCACGGTCAAGATGAC
CTTCATCGTCGTGCTGGCCTTCATCGTGTGCTGGACGCCATTCTTTTTCGTGCAGATGTGG
AGTGTCTGGGATGCCGATGCGCCCAAGGAAGCCTCACCTTTCATCATCGCCATGCTCCTG
GCCAGCCTCAACAGCTGCTGCAACCCCTGGATCTACATGCTCTTCACGGGCCACCTCTTC
CAAGAACTTGTGCAGCGCTTCCTCTGCTGCTCATTCCGCCGCCTGAAAGGCAGCCGGCCT
GGGGAGACAAGCGTCAGCAAAAAGAGCAACTCGTCTACCTTTGTCCTGAGCCAGTACAGC
TCCAGCCAGAGAAGATGCTCGCAGCCATCCACGCTG---

>Bos_primigenius

ATGGAGGGTGCGTTTGCGGCTAACTGGAGCGCTGAGGCGGTCAACGGGAGCGCGGCGC
CGCCGGGAACCGAGGGCAATCGCACTGCTGGGACGCCACAGCGCAACGAGGCCCTGGC
GCGGGTGGAGGTGGCCGTGCTGTGCTTTATCCTGTTTCTGGCGCTGAGCGGCAACGCGT
GCGTGCTGCTAGCGCTGCGCACCACGCGCCACAAGCACTCGCGCCTCTTCTTCTTCATGA
AGCACCTGAGCATAGCCGACCTGGTAGTGGCGGTGTTCCAGGTGCTGCCGCAGCTTCTGT
GGGACATCACGTTCCGCTTCTACGGGCCCGACCTGCTGTGCCGCCTCGTCAAGTACCTGC
AGGTTGTGGGCATGTTCGCGTCCACCTACCTGCTGCTGCTCATGTCGCTCGACCGCTGCC
TGGCCATCTGCCAGCCGCTGCGCTCGCTGAGCCGCCGCACCGACCGCCTGGCGGTACTC
GTCACATGGCTCGGCTGCCTGGGGGCC------
CCGCAGGGTCACATCTTCTCGCTGCGCGAGGTGGCCGACGGTGTCTTCGACTGCTGGGC
CGTTTTCATTCAACCCTGGGGGCCCAAGGCCTACATCACGTGGATCACGCTCGCCGTCTA
CATTGTGCCCGTCATCGTCCTTGCCACCTGCTATGGCCTTATCAGCTTCAAGATCTGGCAG
AATTTACGGCTCAAGACGGCGGCGGCGGAGGCTGCCGCGGGGGCTGAGGGCGAGGCGG
CAGACTGGACGGGGCGCGCGATTCTGGCCCGCGTCAGCAACGTCAAGCTCATCTCTAAG
GCCAAGATCCGCACGGTCAAGATGACCTTCATCGTCGTGCTGGCCTTCATCGTGTGCTGG
ACGCCATTCTTTTTCGTGCAGATGTGGAGTGTCTGGGATGCCGATGCGCCCAAGGAAGCC
TCACCTTTCATCATCGCCATGCTCCTGGCCAGCCTCAACAGCTGCTGCAACCCCTGGATCT
ACATGCTCTTCACGGGCCACCTCTTCCAAGAACTTGTGCAGCGCTTCCTCTGCTGCTCATT
CCGCCGCCTGAAAGGCAGCCGGCCTGGGGAGACAAGCGTCAGCAAAAAGAGCAACTCGT
CTACCTTTGTCCTGAGCCAGTACAGCTCCAGCCAGAGAAGATGCTCGCAGCCATCCACGC
TG---

>Bubalus_bubalis

ATGGAGGGTGCGTTTGCGGCAAACTGGAGCGCTGAGGAGGTCAACGGGAGCGCGGCGC
CGCCGGGAACCGAGGGCAATCGCACTGCCGGGCCGCCACAGCGCAACGAGGCCCTGGC
GCGGGTGGAGGTGGCCGTGCTGTGCCTCATCCTGTTCCTGGCGCTGAGCGGCAACGCGT
GCGTGCTGCTGGCGCTGCGCACCACGCGCCACAAGCACTCGCGCCTCTTCTTCTTCATGA
AGCACCTGAGCATAGCCGACCTGGTGGTGGCGGTGTTCCAGGTGCTGCCGCAGCTTCTGT
GGGACATCACGTTCCGCTTCTACGGGCCCGACCTGCTGTGCCGCCTCGTCAAGTACCTGC
AGGTGGTGGGCATGTTCGCGTCCACCTACCTGCTGCTGCTCATGTCGCTCGACCGCTGCC
TGGCCATCTGCCAGCCGCTGCGCTCGCTGAGCCGCCGCACCGACCGCCTGGCGGTACTC
GTCACATGGCTCGGCTGCCTGGTGGCCAGCGCGCCTCAGGTGCACATCTTCTCGCTGCG
CGAGGTGGCCGACGGTGTCTTCGACTGCTGGGCCGTTTTCATTCAACCCTGGGGGCCCAA
AGCCTACATCACGTGGATCACGCTCGTCGTCTACATTGTGCCCGTCATCGTGCTTGCCACC
TGCTATGGCCTTATCAGCTTCAAGATCTGGCAGAATTTACGGCTCAAGACGGCGGCGGCG
GAGGCTGCCGCGGGGGCTGAGGGCGCGGCGGCAGACTGGGCGGGGCGCGCGGCTCTG
GCCCGCGTCAGCAACGTCAAGCTCATCTCTAAGGCCAAGATCCGCACGGTCAAGATGACC
TTCATCGTCGTGCTGGCCTTCATCGTGTGCTGGACGCCATTCTTTTTCGTGCAGATGTGGA

GTGTCTGGGATGCCGATGCGCCCAAGGAAGCCTCGCCTTTCATCATCGCCATGCTCCTGG
CCAGCCTCAACAGCTGCTGCAACCCCTGGATCTACATGCTCTTCACGGGCCACCTCTTCCA
AGAACTTGTGCAGCGCTTCCTCTGCTGCTCATTCCGCCGCCTGAAAGGCAGCCGGCCTGG
GGAGACAAGCGTCAGCAAAAAGAGCAACTCGTCCACCTTTGTCCTGAGCCAGTACAGCTC
CGGCCAGAGAAGATGCTCGCAGCCATCCATGCTG---

>Bos_mutus

ATGGAGGGTGCGTTTGCGGCTAACTGGAGCGCTGAGGCGGTCAACGGGAGCGCGGCGC
CGCCGGGAACCGAGGGCAATCGCACTGCCGGGCCGCCACAGCGCAACGAGGCCCTGGC
GCGGGTGGAGGTGGCCGTGCTGTGCCTCATCCTGTTCCTGGCGCTGAGCGGCAACGCGT
GCGTGCTGCTAGCGCTGCGCACCACGCGCCACAAGCACTCGCGCCTCTTCTTCTTCATGA
AGCACCTGAGCATAGCCGACCTGGTGGTGGCGGTGTTCCAGGTGCTGCCGCAGCTTCTGT
GGGACATCACGTTCCGCTTCTACGGGCCCGACCTGCTGTGCCGCCTCGTCAAGTACCTGC
AGGTTGTGGGCATGTTCGCGTCCACCTACCTGCTGCTGCTCATGTCGCTCGACCGCTGCC
TGGCCATCTGCCAGCCGCTGCGCTCGCTGAGCCGCCGCACCGACCGCCTGGCGGTACTC
GTCACATGGCTCGGCTGCCTGGTGGCCAGCGCGCCGCAGGTGCACATCTTCTCGCTGCG
CGAGGTGGCCGACGGTGTCTTCGACTGCTGGGCCGTTTTCATTCAACCCTGGGGGCCCAA
GGCCTACATCACGTGGATCACGCTCGCCGTCTACATTGTGCCCGTCATCGTGCTTGCCAC
CTGCTATGGCCTTATCAGCTTCAAGATCTGGCAGAATTTACGGCTCAAGACGGCGGCGGC
GGAGGCTGCCGCGGGGGCTGAGGGCGAGGCGGCAGACTGGGCTGGGCGCGCGATTCTG
ACCCGCGTCAGCAACGTCAAGCTCATCTCTAAGGCCAAGATCCGCACGGTCAAGATGACC
TTCATCGTCGTGCTGGCCTTCATCGTGTGCTGGACGCCATTCTTTTTCGTGCAGATGTGGA
GTGTCTGGGATGCCGATGCGCCCAAGGAAGCCTCACCTTTCATCATCGCCATGCTCCTGG
CCAGCCTCAACAGCTGCTGCAACCCCTGGATCTACATGCTCTTCACGGGCCACCTCTTCCA
AGAACTTGTGCAGCGCTTCCTCTGCTGCTCATTCCGCCGCCTGAAAGGCAGCCGGCCTGG
GGAGACAAGCGTCAGCAAAAAGAGCAACTCGTCCACCTTTGTCCTGAGCCAGTACAGCTC
CAGCCAGAGAAGATGCTCGCAGCCATCCACGCTG---

>Sus_scrofa_familiaris

ATGGAGGGGGTGCTTGCAGCCAACTGGAGCGCCGAGGCGGTCAACTCGAGCGCGGCGC
CGCCAGAGGCCGAGGGCAACCGCACCGCCGGGCCGCCGCAGCGCAACGAGGCCCTGGC
GCGTGTGGAGGTGGCGGTGTTGTGCCTCATTCTCTTCCTAGCTCTGAGCGGCAATGCGTG
CGTGCTGCTGGCGCTGCGCACCACGCGCCACAAGCATTCGCGCCTCTTCTTCTTCATGAA
ACACCTGAGCATAGCCGACCTGGTGGTGGCGGTGTTCCAGGTGCTGCCTCAGCTACTGTG
GGATATCACCTTCCGCTTCTACGGACCCGACTTGCTGTGCCGCCTCGTCAAGTACCTCCA
GGTGGTGGGCATGTTCGCTTCCACTTACCTGCTGCTGCTCATGTCGCTCGACCGCTGTCT
GGCCATCTGCCAGCCGTTGCGCGCGCTGCGCCGCCGCGCGGATCGTCTGGCAGTGCTAG
CCACATGGCTGGGCTGCCTGGTGGCCAGCGCGCCGCAGGTGCACATCTTCTCGCTGCGC
GAGGTGGCCGACGGCGTCTTCGATTGCTGGGCCGTCTTCATCCAGCCCTGGGGGCCCAA
GGCCTACATCACGTGGATCACACTTGCGGTCTACATCGTGCCGGTCATCGTGCTCGCCGC
CTGCTACGGCCTTATCAGCTTCAAGATCTGGCAGAACTTGCGGCTCAAGACTGCGGCGGC
GGAGGCGATCGCGGGAACCGAGGGCGCGGCGGCGGGCAGCCGGGGCCGAGCGGCTCT
GGCCCGCGTCAGCAGCGTCAAGCTCATCTCCAAGGCCAAGATCCGCACCGTCAAGATGAC
CTTCATCATTGTGCTGGCCTTCATCGTATGCTGGACGCCATTCTTTTTCGTGCAGATGTGG
AGCGTCTGGGACGCCGATGCGCCCAAGGAAGCCTCAGCTTTCATCATTGCAATGCTCCTG
GCCAGCCTCAACAGCTGCTGCAACCCCTGGATCTACATGCTCTTCACAGGCCACCTCTTCC
ATGAACTTGTGCAGCGCTTCCTTTGCTGCTCTTCCAGCCACCTGAAGACCAGCCGGCCCG
GGGAGACGAGTGTCAGCAAAAAGAGCAACTCGTCCACCTTTGTCTTGAGCCAGCACAGCT
CCAGCCAGAAGAGCTGCTCG------------------

>Sus_scrofa

ATGGAGGGGGTGCTTGCAGCCAACTGGAGCGCCGAGGCGGTCAACTCGAGCGCGGCGC
CGCCAGAGGCCGAGGGCAACCGCACCGCCGGGCCGCCGCAGCGCAACGAGGCCCTGGC
GCGTGTGGAGGTGGCGGTGTTGTGCCTCATTCTCTTCCTAGCTCTGAGCGGCAATGCGTG
CGTGCTGCTGGCGCTGCGCACCACGCGCCACAAGCATTCGCGCCTCTTCTTCTTCATGAA
ACACCTGAGCATAGCCGACCTGGTGGTGGCGGTGTTCCAGGTGCTGCCTCAGCTACTGTG
GGATATCACCTTCCGCTTCTACGGACCCGACTTGCTGTGCCGCCTCGTCAAGTACCTCCA
GGTGGTGGGCATGTTCGCTTCCACTTACCTGCTGCTGCTCATGTCGCTCGACCGCTGTCT
GGCCATCTGCCAGCCGTTGCGCGCGCTGCGCCGCCGCGCGGATCGTCTGGCAGTGCTAG

CCACATGGCTGGGCTGCCTGGTGGCCAGCGCGCCGCAGGTGCACATCTTCTCGCTGCGC
GAGGTGGCCGACGGCGTCTTCGATTGCTGGGCCGTCTTCATCCAGCCCTGGGGGCCCAA
GGCCTACATCACGTGGATCACACTTGCGGTCTACATCGTGCCGGTCATCGTGCTCGCCGC
CTGCTACGGCCTTATCAGCTTCAAGATCTGGCAGAACTTGCGGCTCAAGACTGCGGCGGC
GGAGGCGATCGCGGGAACCGAGGGCGCGGCGGCGGGCAGCCGGGGCCGAGCGGCTCT
GGCCCGCGTCAGCAGCGTCAAGCTCATCTCCAAGGCCAAGATCCGCACCGTCAAGATGAC
CTTCATCATTGTGCTGGCCTTCATCGTATGCTGGACGCCATTCTTTTTCGTGCAGATGTGG
AGCGTCTGGGACGCCGATGCGCCCAAGGAAGGCAGGGCGGTCGGGAAACCAAGGCTCCT
GGCCAGCCTCAACAGCTGCTGCAACCCCTGGATCTACATGCTCTTCACAGGCCACCTCTT
CCATGAACTTGTGCAGCGCTTCCTTTGCTGCTCTTCCAGCCACCTGAAGACCAGCCGGCC
CGGGGAGACGAGTGTCAGCAAAAAGAGCAACTCGTCCACCTTTGTCTTGAGCCAGCACAG
CTCCAGCCAGAAGAGCTGCTCG------------------

>Felis_catus

ATGGAGGGCGCGCTTGCGGCCAACTGGAGCGCGGAGGCGGTCAATGGCAGCGAGGCGC
CGCCGGGAGCCGAGGGCAACCGCACCGCCGGGCCCCCGCAGCGCAACGAGGCCCTGGC
GCGCGTGGAGGTGGCCGTGCTGTGCCTCATTCTCTTCCTGGCGCTGAGCGGCAACGCGT
GCGTGCTACTGGCGCTGCGCACCACGCGCCACAAGCACTCGCGCCTTTTCTTTTTCATGA
AGCACCTGAGTATAGCCGACCTGGTGGTGGCAGTGTTCCAGGTGCTGCCGCAACTGCTCT
GGGACATCACTTTCCGCTTCTACGGGCCCGACCTGCTGTGCCGCCTGGTCAAGTACCTGC
AGGTGGTGGGCATGTTCGCCTCCACCTACTTGCTGCTGCTCATGTCTCTCGACCGCTGCC
TGGCCATCTGCCAGCCGCTGCGGGCGCTGCGTCGCCGCGCGGACCGCCTGGCGGTGCT
CGCCACGTGGCTGGGGTGCCTGGTGGCCAGCGCGCCCCAAGTGCACATCTTCTCGCTGC
GCGAGGTGGCCGACGGCGTCTTCGACTGCTGGGCCGTCTTCATCCAGCCCTGGGGGCCC
AAGGCCTACATCACGTGGATCACGCTCGCGGTCTATATCGTGCCTGTCATCGTGCTCGCC
GTCTGCTACGGCCTCATCAGCTTCAAGATCTGGCAGAACTTGCGGCTCAAGACCGCGGCG
GCGGCGGCG---
GAGGGGCAGGAGGGCGCGGCGGCGGGCAGCGCTGGGCGCGCGGCTCTGGCTCGCGTC
AGCAGCGTGAAGCTCATCTCCAAGGCCAAGATCCGCACCGTCAAGATGACCTTCATTATCG
TGCTGGCCTTCATCGTGTGCTGGACGCCATTCTTCTTCGTTCAGATGTGGAGCGTCTGGGA
CGCCAATGCGCCCAAGGAAGCCTCGGCCTTCATCATAGCCATGCTCCTGGCCAGCCTCAA
CAGCTGCTGCAACCCTTGGATCTACATGCTCTTCACTGGCCACCTCTTCCACGAACTTGTA
CAGCGCTTCCTCTGCTGCTCCTCCAGCTACCTGAAGGGCAGCCGGCCGGGAGAGACCAG
CGTCAGCAAAAAGAGCAACTCCTCCACCTTCGTCCTGAGCCGCCACAGCTCCAGCCAGAG
GAGCTCCTCGCAGCCATCCACGGTG---

>Felis_silvestris

ATGGAGGGCGCGCTTGCGGCCAACTGGAGCGCGGAGGCGGTCAATGGCAGCGAGGCGC
CGCCGGGAGCCGAGGGCAACCGCACCGCCGGGCCCCCGCAGCGCAACGAGGCCCTGGC
GCGCGTGGAGGTGGCCGTGCTGTGCCTCATTCTCTTCCTGGCGCTGAGCGGCAACGCGT
GCGTGCTACTGGCGCTGCGCACCACGCGCCACAAGCACTCGCGCCTTTTCTTTTTCATGA
AGCACCTGAGTATAGCCGACCTGGTGGTGGCAGTGTTCCAGGTGCTGCCGCAACTGCTCT
GGGACATCACTTTCCGCTTCTACGGGCCCGACCTGCTGTGCCGCCTGGTCAAGTACCTGC
AGGTGGTGGGCATGTTCGCCTCCACCTACTTGCTGCTGCTCATGTCTCTCGACCGCTGCC
TGGCCATCTGCCAGCCGCTGCGGGCGCTGCGTCGCCGCGCGGACCGCCTGGCGGTGCT
CGCCACGTGGCTGGGGTGCCTGGTGGCCAGCGCGCCCCAAGTGCACATCTTCTCGCTGC
GCGAGGTGGCCGACGGCGTCTTCGACTGCTGGGCCGTCTTCATCCAGCCCTGGGGGCCC
AAGGCCTACATCACGTGGATCACGCTCGCGGTCTATATCGTGCCTGTCATCGTGCTCGCC
GTCTGCTACGGCCCCATCAGCTTCAAGATCTGGCAGAACTTGCGGCTCAAGACCGCGGCG
GCGGCGGCG---
GAGGGGCAGGAGGGCGCGGCGGCGGGCAGCGCTGGGCGCGCGGCTCTGGCTCGCGTC
AGCAGCGTGAAGCTCATCTCCAAGGCCAAGATCCGCACCGTCAAGATGACCTTCATTATCG
TGCTGGCCTTCATCGTGTGCTGGACGCCATTCTTCTTCGTTCAGATGTGGAGCGTCTGGGA
CGCCAATGCGCCCAAGGAAGGTAGGGCCTTCATCATAGCCATGCTCCTGGCCAGCCTCAA
CAGCTGCTGCAACCCTTGGATCTACATGCTCTTCACTGGCCACCTCTTCCACGAACTTGTA
CAGCGCTTCCTCTGCTGCTCCTCCAGCTACCTGAAGGGCAGCCGGCCGGGAGAGACCAG
CGTCAGCAAAAAGAGCAACTCCTCCACCTTCGTCCTGAGCCGCCACAGCTCCAGCCAGAG
GAGCTCCTCGCAGCCATCCACGGTG---

>Odobenus_rosmarus

ATGGAGGGCGCGCTGGTGGCCAACTGGACCGCGGAGGCGGTCAACGGCAGCGAGGCGC
CTCCGGAAGCCGAGGGCAACCGCACCGCCGGGCCCCCGCAGCGCAACGAGGCCCTGGC
GCGCGTGGAGGTGGCCGTGCTGTGCCTCATCCTCTTCCTGGCGCTGAGCGGCAACGCGT
GCGTGCTCCTGGCGCTGCGCACCACGCGCCACAAGCACTCGCGCCTCTTCTTCTTCATGA
AGCACCTGAGCATAGCCGACCTGGTGGTGGCAGTGTTCCAGGTGCTGCCGCAACTTCTGT
GGGACATCACCTTCCGCTTCTACGGGCCCGACCTGCTGTGCCGCCTGGTCAAGTACCTGC
AGGTGGTGGGCATGTTCGCCTCCACCTACCTGCTGCTGCTCATGTCTCTCGACCGCTGCC
TGGCCATCTGCCAGCCCCTGCGGGCGCTGCGGCGCCGCGCGGACCGCCTGGCCGTGCT
CGCCACGTGGCTGGGCTGCCTGGTGGCCAGCGCGCCGCAAGTGCACATCTTCTCGCTGC
GCGAGGTGGCCGACGGCGTCTTCGACTGCTGGGCCGTCTTCATCCAGCCGTGGGGGCCC
AAGGCCTACATCACGTGGATCACACTCGCGGTCTACATCGTGCCTGTCATCGTGCTCGCC
GCCTGCTACGGCCTCATCAGCTTCAAGATCTGGCAGAACTTGCGACTCAAGACGGCGGCG
GCGGCGGCG------------
GCGGCTGCGGCTGCAGAGGGGCCGGAGGGCGCGGCTCTGGCACGGGTCAGCAGCGTCA
AGCTCATCTCCAAGGCCAAGATCCGCACGGTTAAGATGACCTTCATTATCGTGCTGGCCTT
CATCGTGTGCTGGACGCCATTCTTCTTCGTGCAGATGTGGAGCGTCTGGGACGCCAATGC
GCCCAAGGAAGCCTCGGCTTTCATCATAGCCATGCTCCTGGCCAGCCTCAACAGCTGCTG
CAACCCTTGGATCTACATGCTCTTCACCGGCCACCTCTTCCACGAACTTGTACAACGCTTC
CTCTGCTGCTCCTCCGGCTTCCTGAAGGGCAACCGGCCGGGAGAGACCAGCGTCAGCAA
AAAGAGCAACTCATCTACCTTTGTCCTGAGCTGCCACAGCTCCAGCCAGAGGAGCTGCTC
GCAGCCATCCACAGTG---

>Mustela_putorius_furo

ATGGAGGGCTCTCTGGTGGCCAACTGGAGCGCCGAGGCGGTCAACGGCAGCGAGGCGC
CTCCGGAAGCCGAGGGCAACCGCACCGCCGGGCCCCCGCAGCGCAACGAGGCCCTGGC
GCGCGTGGAGGTGGCGGTGCTGTGCCTCATTCTCTTCCTGGCGCTGAGCGGCAACGCTT
GCGTGCTACTAGCGCTGCGCACCACGCGCCACAAACACTCGCGCCTCTTCTTCTTCATGA
AGCACCTGAGCATAGCCGACCTGGTGGTGGCCGTGTTCCAGGTGCTGCCGCAACTGCTGT
GGGACATCACCTTCCGCTTCTACGGGCCCGACCTGCTGTGCCGCCTGGTCAAGTACCTGC
AGGTGGTGGGCATGTTCGCCTCCACCTACCTGCTACTGCTCATGTCTCTGGACCGCTGCC
TGGCCATCTGCCAGCCGCTGCGGGCGCTGCGTCGCCGGGCGGACCGCCTGGCGGTGCT
CGCCACGTGGCTGGGCTGCCTGGTGGCTAGCGCGCCGCAAGTGCACATCTTCTCGCTGC
GCGAGGTGGCGGACGGCGTCTTCGACTGCTGGGCCGTCTTCATCCAGCCCTGGGGGCCC
AAGGCCTACATCACGTGGATCACACTCGCCGTCTACATCGTGCCTGTCATCGTGCTCGCC
GCCTGCTACGGCCTCATCAGCTTCAAGATCTGGCAGAACTTGAGACTCAAGACGGCGGCG
GCGGAGGCTGCCGAGGGGTCGGAGGGCCGGGCGGCGGGCAGCGCGGGGAGCGCGGCT
CTGGCACGCGTCAGCAGCGTCAAGCTCATCTCCAAGGCCAAGATCCGCACGGTTAAGATG
ACCTTCATTATCGTGCTGGCCTTCATCGTGTGCTGGACGCCATTCTTCTTCGTGCAGATGT
GGAGCGTCTGGGATGCCAACGCGCCCAAGGAAGCCTCAGCTTTCATCATAGCCATGCTCC
TGGCCAGCCTCAACAGCTGCTGCAACCCGTGGATCTACCTGCTCTTCACGGGCCATCTCT
TCCATGAACTGGTACAGCGCTTCCTCTGCTGCTCCTCCAGCTACCTGAAGGGCAACCGTC
CAGGAGAGACCAGCGTCAGCAAAAAGAGCAACTCCTCCACTTTTGTCCTGAGCCGCCACA
GCTCCAGCCAGAGGAGCTGCTCACAGCCATCCACAGTG---

>Canis_lupus

ATGGAGCGCGCGCTGGCGGCCAACTGGAGCGCGGAGGCGGGCAACGGCAGCGAGGCG
GCCCCGGCGGCGCAGGGCAACCGCACGGCCGGGCCCCCGCAGCGCAACGAGGCCCTG
GCGCGCGTGGAGGTGGCCGTGCTGTGCCTCATCCTCTTCCTGGCGCTGAGCGGCAACGC
GTGCGTGCTCCTGGCGCTGCGCACCACGCGCCACAAGCACTCGCGCCTCTTCTTCTTCAT
GAAGCACCTGAGCATAGCCGACCTGGTGGTGGCGGTGTTCCAGGTGCTGCCGCAGCTGC
TGTGGGACATCACCTTCCGCTTCTACGGGCCCGACCTGCTGTGCCGCCTGGTCAAGTACC
TGCAGGTGGTGGGCATGTTCGCCTCCACCTACCTGCTGCTGCTCATGTCCCTGGACCGCT
GCCTGGCCATCTGCCAGCCGCTGAGGGCGCTGCGCCGCCGCGCGGACCGCCTGGCCGT
GCTCGCCACGTGGCTGGGCTGCCTGGTGGCCAGCGCGCCGCAGGTGCACATCTTCTCGC
TGCGCGAGGTGGCCGACGGCGTCTTCGACTGCTGGGCCGTCTTCATCCAGCCCTGGGGG
CCCAAGGCCTACGTCACGTGGATCACGCTCTCCGTCTACATCGTGCCCGTCATCGTGCTC
GCCGCCTGCTACGGCCTCATCAGCTTCAAAATTTGGCAGAACCTGCGACTCAAGACGGCG
GCGGCGGCGGCCGCGGAGGGGCGGGAGG---------------
GCGCGGGGCGCGCGGCCCTGGCTCGGGTCAGCAGCGTCAAGCTCATCTCCAAGGCCAA
GATCCGCACCGTGAAGATGACCTTCATTATCGTGCTGGCCTTCATCGTGTGCTGGACGCC

GTTCTTCTTCGTGCAGATGTGGAGCGTCTGGGACGCCGATGAGAAAGAGGAAGCAGGAGC
TTTCAGCATGGTAATGCTCCTCCCCACCCCCACCTGCTGTTGGAGCCCTGCTTTAGAGAGG
CTCTGCAGGGACCACACCCACGGCAGGTTTGGACCA-
GCCCCCTATGTTCCTTCACAAGCTTCCTAGAGGAGA---
AGCTTTTGAATACCAGCGTAAACAAACAAACAAACAAATCCACCTTTGTCCTGAGCCACCAC
ACTTCCAGCCAGAGGAGCGCTTCACTTCCTTCCACAGTG---

>Canis_lupus_familiaris

ATGGAGCGCGCGCTGGCGGCCAACTGGAGCGCGGAGGCGGGCAACGGCAGCGA
GGCGGCCCCGGCGGCGCAGGGCAACCGCACGGCCGGGCCCCCGCAGCGCAACGAGGC
CCTGGCGCGCGTGGAGGTGGCCGTGCTGTGCCTCATCCTCTTCCTGGCGCTGAGCGGCA
ACGCGTGCGTGCTCCTGGCGCTGCGCACCACGCGCCACAAGCACTCGCGCCTCTTCTTCT
TCATGAAGCACCTGAGCATAGCCGACCTGGTGGTGGCGGTGTTCCAGGTGCTGCCGCAG
CTGCTGTGGGACATCACCTTCCGCTTCTACGGGCCCGACCTGCTGTGCCGCCTGGTCAAG
TACCTGCAGGTGGTGGGCATGTTCGCCTCCACCTACCTGCTGCTGCTCATGTCCCTGGAC
CGCTGCCTGGCCATCTGCCAGCCGCTGAGGGCGCTGCGCCGCCGCGCGGACCGCCTGG
CCGTGCTCGCCACGTGGCTGGGCTGCCTGGTGGCCAGCGCGCCGCAGGTGCACATCTTC
TCGCTGCGCGAGGTGGCCGACGGCGTCTTCGACTGCTGGGCCGTCTTCATCCAGCCCTG
GGGGCCCAAGGCCTACGTCACGTGGATCACGCTCTCCGTCTACATCGTGCCCGTCATCGT
GCTCGCCGCCTGCTACGGCCTCATCAGCTTCAAAATTTGGCAGAACCTGCGACTCAAGAC
GGCGGCGGCGGCGGCCGCGGAGGGGCGGGAGG--------------
GCGCGGGGCGCGCGGCCCTGGCTCGGGTCAGCAGCGTCAAGCTCATCTCCAAGGCCAA
GATCCGCACCGTGAAGATGACCTTCATAATCGTGCTGGCCTTCATCGTGTGCTGGACGCC
GTTCTTCTTCGTGCAGATGTGGAGCGTCTGGGACGCCGATGCGCCCAAGGAAGCCTCGG
CTTTCATCATAGCCATGCTCCTGGCCAGCCTCAACAGCTGCTGTAACCCTTGGATCTACAT
GCTCTTCACGGGCCACCTCTTCCATGAACTTGTACAGCGCTTCCTCTGCTGCTCCTCCAGC
TACCTGAAGGGGAACCGTCCAGGGGAGACCAGCGTCAGCAAAAAGAGCAACTCATCCACC
TTTGTCCTGAGCCACCACAGCTCCAGCCAGAGGAGCTCCTCGCAGCCATCCACAGTG---
