# Supplementary material for: Oxytocin and arginine vasopressin systems in the domestication process
Source: Genet Mol Biol. 2018 Mar 26;41(1 Suppl 1):235–42. doi: 10.1590/1678-4685-GMB-2017-0069 (PMC5913714; doi:10.1590/1678-4685-GMB-2017-0069)
Supplement: Supplementary file 5 [file 1415-4757-GMB-41-01-2017-0069-s002.pdf]

Supplementary Material to "Oxytocin and Arginine Vasopressin Systems in the Domestication Process"

Table S2 - Estimated parameters under different codon substitution models for OXT and AVP system genes*

	Model	*dN/dS*	Estimated parameters	ℓ	*P value*
	M1a: neutral	0.191	p0= 0.90797, (p1= 0.09203)	-4235.879435	
			(ω0= 0.10897), (ω1= 1.00000)		p=1
	M2a: selection	0.191	p0=0.90797, p1=0.06179, (p2=0.03023)	-4235.879435	
AVP			(ω0= 0.10897), (ω1= 1.00000), ω2= 1.00000		
	M7: β	0.1375	p= 0.96361, q= 5.90598	-4202.397596	
					p=0.3755284668671542
	M8: β & ω		p0 = 0.99438, (p1= 0.00562), p= 0.99933	-4201.418175	
			q= 6.36430, ω=1.68047		
	M1a: neutral	0.1297	p0= 0.94069, (p1=0.05931)	-2814.136256	
			(ω0= 0.07484), (ω1= 1.00000)		p=1
	M2a: selection	0.1297	p0= 0.94069, p1= 0.03464, (p2= 0.02467)	-2814.136256	
OXT			(ω0= 0.07484), (ω1= 1.00000), ω2= 1.00000		
	M7: β	0.1078	p= 0.62254, q= 4.98970	-2792.538098	
					p=0.111690123136207
	M8: β & ω	0.1201	p0= 0.96325, (p1= 0.03675), p= 0.83505	-2790.346071	
			q= 8.54946, ω= 1.00000		

	Model	*dN/dS*	Estimated parameters	ℓ	*P value*
AVPR1A	M1a: neutral	0.2328	p0= 0.81564, (p1= 0.18436) (ω0= 0.05939), (ω1= 1.00000)	-9372.53479	p=1
	M2a: selection	0.2328	p0=0.81564, p1= 0.08385, (p2= 0.10051) (ω0=0.05939), (ω1= 1.00000), ω2= 1.00000	-9372.53479	
	M7: β	0.1588	p= 0.23572, q= 1.22402	-9294.294221	p=0.2353604758888066
	M8: β & ω	0.1574	p0= 0.96125, (p1= 0.03875), p= 0.27485 q= 1.88627, ω=1.00428	-9292.847584	
AVPR1B	M1a: neutral	0.2876	p0= 0.78293, p1= 0.21707 ω0= 0.09013, ω1= 1.00000	-11528.4793	p=1
	M2a: selection	0.2883	p0= 0.78188, p1= 0.10650, p2= 0.11162 ω0= 0.08978, ω1= 1.00000, ω2= 1.00000	-11528.1628	
	M7: β	0.213	p= 0.39619, q= 1.44314	-11481.31444	p=0.001986092775002057
	M8: β & ω	0.2271	p0= 0.96900, (p1 = 0.03100), p= 0.43006 q= 1.81490, ω=1.42987	-11475.09285	
AVPR2	M1a: neutral	0.2232	p0= 0.83215, p1= 0.16785 ω0= 0.06656, ω1= 1.00000	-8507.043425	p=1
	M2a: selection	0.2232	p0=0.83215, p1= 0.07801, p2= 0.08984	-8507.043424	

	Model	*dN/dS*	Estimated parameters	ℓ	*P value*
			ω0= 0.06656, ω1= 1.00000, ω2= 1.00000		
	M7: β	0.1976	p= 0.26163, q= 1.05030	-8502.12558	p=3.279968252556387e-00
	M8: β & ω	0.2062	p0= 0.94656, (p1= 0.05344), p= 0.38118	-8484.892733	
			q= 2.40174, ω= 1.49853		
OXTR	M1a: neutral	0.1256	p0= 0.90805, p1=0.09195	-7579.55727	
			ω0= 0.03704, ω1= 1.00000		p=1
	M2a: selection	0.1256	p0= 0.90805, p1= 0.05432, p2= 0.03763	-7579.55727	
			ω0= 0.03704, ω1= 1.00000, ω2= 1.00000		
	M7: β	0.0745	p = 0.20501, q= 2.38379	-7496.04886	p=1
	M8: β & ω	0.0745	p0= 0.99999, (p1= 0.00001), p= 0.20500	-7496.04935	
			q= 2.38372, ω= 1.00000		

* p_0 = proportion of sites where ω< 1; p_1 = proportion of sites where ω = 1 and p_2 = proportion of sites where ω> 1 (selection models only); ω_0< 1 (negative selection), ω_1 = 1 (neutral selection) and ω_2 >1 (positive selection);Likelihood ratio tests were performed between neutral models (M1a- Nearly Neutral, and M7 - Beta) and models that identify positive selection and/or relaxationof functional constraints (M2a – Selection and M8 – Beta+ Selection); In the comparisons, M1a vs M2a has 2 degrees of freedom (df=2), and M7 vs M8 too (df=2). Parentheses indicate fixed parameters.
