# Supplementary material for: Oxytocin and arginine vasopressin systems in the domestication process
Source: Genet Mol Biol. 2018 Mar 26;41(1 Suppl 1):235–42. doi: 10.1590/1678-4685-GMB-2017-0069 (PMC5913714; doi:10.1590/1678-4685-GMB-2017-0069)
Supplement: Supplementary file 7 [file 1415-4757-GMB-41-01-2017-0069-s004.pdf]

Supplementary Material to "Oxytocin and Arginine Vasopressin Systems in the Domestication Process"

Table S4 - Results of the Clade D analysis

Genes	site class	M1a			CLADE D				M1a x CLADE D
		w0	*w1*	*l*	w0	w1	w2	*l*	p value
AVP	proportion	0.93094	0.06906		0.12641	0.33970	0.53389		p<0.00001
Wild	branch type 0:	0.09073	1.00000	4713.27436	0.00000	0.26984	0.06705	-4176.453125	
Domestic	branch type 1:				0.00000	0.26984	0.01924		
OXT	proportion	0.93395	0.06605		0.44041	0.07607	0.48352		p<0.00001
Wild	branch type 0:	0.06275	1.00000	3191.355008	0.01044	0.54518	0.12443	-3149.107899	
Domestic	branch type 1:				0.01044	0.54518	0.08374		
AVPR1A	proportion	0.77893	0.22107		0.31565	0.13724	0.54711		p<0.00001
Wild	branch type 0:	0.0532	1.00000	-12248.66491	0.19907	0.76449	0.00347	-12077.75279	
Domestic	branch type 1:				0.19907	0.76449	0.00259		
AVPR1B	proportion	0.77022	0.22978		0.47187	0.18804	0.34010		p<0.00001
Wild	branch type 0:	0.07827	1.00000	-14524.93717	0.01616	0.78460	0.20646	-14418.02824	
Domestic	branch type 1:				0.01616	0.78460	0.16162		
AVPR2	proportion	0.81749	0.18251		0.47616	0.15213	0.37171		p<0.00001
Wild	branch type 0:	0.05722	1.00000	-10401.12772	0.00649	0.88746	0.17147	-10345.71596	
Domestic	branch type 1:				0.00649	0.88746	0.11429		
OXTR	proportion	0.90248	0.09752		0.61235	0.10596	0.28170		p<0.00001
Wild	branch type 0:	0.03297	1.00000	-8985.83008	0.00158	0.42724	0.09584	-8845.11211	
Domestic	branch type 1:				0.00158	0.42724	0.06669		

[1] p0 = proportion of sites where $\omega < 1$; p1 = proportion of sites where $\omega = 1$ and p2 = proportion of sites where $\omega > 1$ (selection models only); $\omega_0 < 1$ (negative selection), $\omega_1 = 1$ (neutral selection) and $\omega_2 > 1$ (positive selection). A Likelihood ratio test was performed with 3 degrees of freedom.
