# Supplementary material for: Oxytocin and arginine vasopressin systems in the domestication process
Source: Genet Mol Biol. 2018 Mar 26;41(1 Suppl 1):235–42. doi: 10.1590/1678-4685-GMB-2017-0069 (PMC5913714; doi:10.1590/1678-4685-GMB-2017-0069)
Supplement: Figure S5 [file 1415-4757-GMB-41-01-2017-0069-s010.pdf]

Supplementary Material to "Oxytocin and Arginine Vasopressin Systems in the Domestication Process"

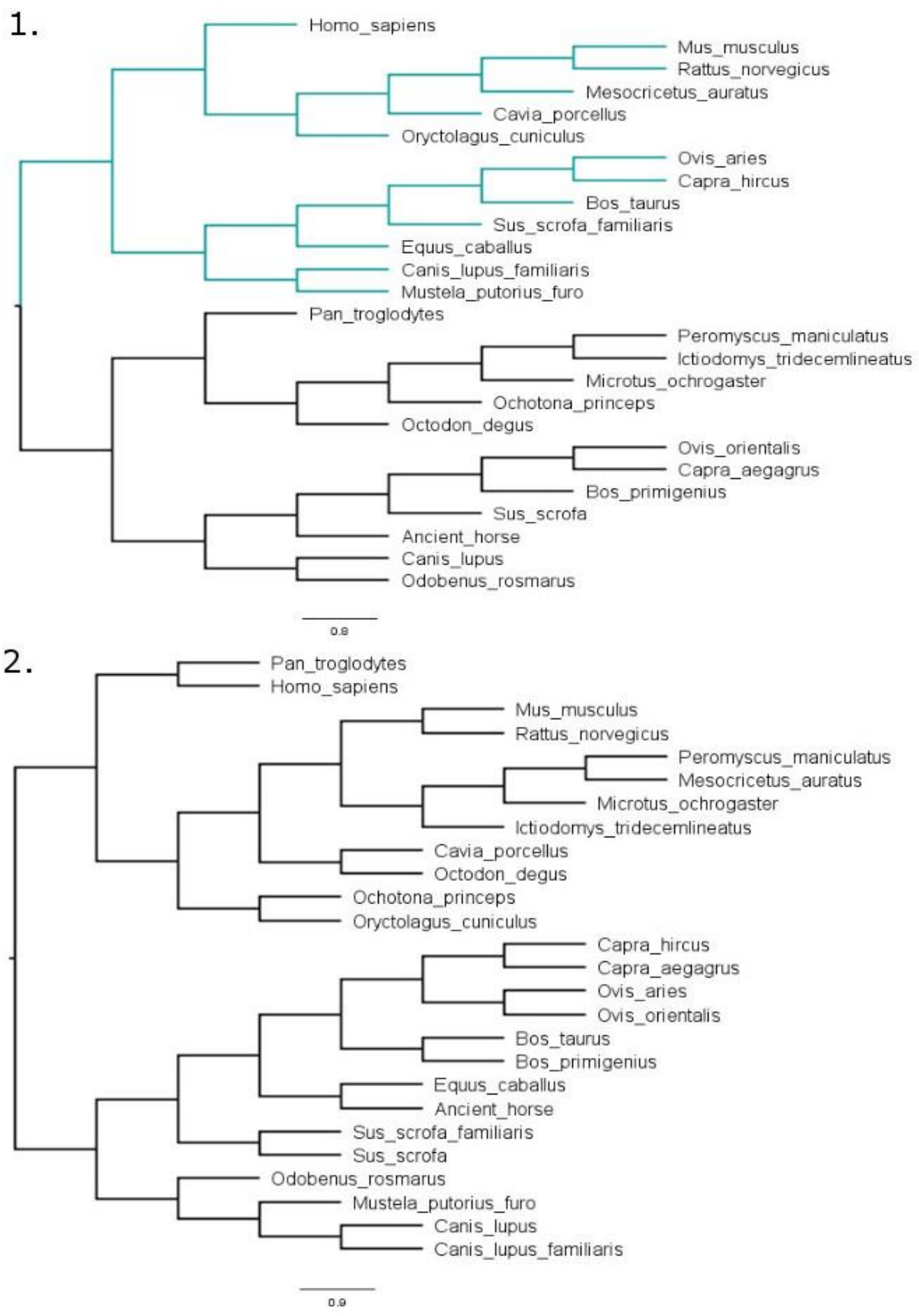

Figure S5 - Phylogenetic trees of 26 species of mammals used in *OXT* analyses, 1: mirrored tree, 2: phylogenetic tree.
